# A Randomized Open label Phase-II Clinical Trial with or without Infusion of Plasma from Subjects after Convalescence of SARS-CoV-2 Infection in High-Risk Patients with Confirmed Severe SARS-CoV-2 Disease (RECOVER): A structured summary of a study protocol for a randomised controlled trial

**DOI:** 10.1186/s13063-020-04735-y

**Published:** 2020-10-06

**Authors:** Maike Janssen, Ulrike Schäkel, Carine Djuka Fokou, Johannes Krisam, Jacek Stermann, Katharina Kriegsmann, Isabella Haberbosch, Jan Philipp Novotny, Stefan Weber, Maria Vehreschild, Martin Bornhäuser, Lars Bullinger, Michael Schmitt, Tobias Liebregts, Peter Dreger, Hanns-Martin Lorenz, Anna Plaszczyca, Ralf Bartenschlager, Barbara Müller, Hans-Georg Kräusslich, Niels Halama, Dirk Jäger, Richard F. Schlenk, Albrecht Leo, Stefan Meuer, Markus A. Weigand, Johann Motsch, Uta Merle, Claudia M. Denkinger, Carsten Müller-Tidow

**Affiliations:** 1 Department of Internal Medicine V, Heidelberg University Hospital, Heidelberg, Germany; 2 NCT-Trial Center, National Center of Tumor Diseases, Heidelberg University Hospital and German Cancer Research Center, Heidelberg, Germany; 3 Institute of Medical Biometry and Informatics, University of Heidelberg, Heidelberg, Germany; 4 Division of Tropical Medicine, Department of Infectious Diseases, Heidelberg University Hospital, Heidelberg, Germany; 5 Division of Infectious Diseases, University Hospital Frankfurt, Frankfurt, Germany; 6 University Hospital Dresden, Dresden, Germany; 7 Charité University Medicine, Berlin, Germany; 8 Department of Infectious Diseases, Molecular Virology, Heidelberg University Hospital, Heidelberg, Germany; 9 Department of Infectious Diseases, Virology, Heidelberg University Hospital, Heidelberg, Germany; 10 Medical Oncology, National Center for Tumor Diseases (NCT), Heidelberg, Germany; 11 Institute for Clinical Transfusion Medicine and Cell Therapy Heidelberg, Heidelberg, Germany; 12 Department of Anaesthesiology, Heidelberg University Hospital, Heidelberg, Germany; 13 Department of Internal Medicine IV, Heidelberg University Hospital, Heidelberg, Germany

**Keywords:** COVID-19, Randomised controlled trial, protocol, convalescent plasma, early application, high-risk patients with severe disease

## Abstract

**Objectives:**

Primary objectives

• To assess the time from randomisation until an improvement within 84 days defined as two points on a seven point ordinal scale or live discharge from the hospital in high-risk patients (group 1 to group 4) with SARS-CoV-2 infection requiring hospital admission by infusion of plasma from subjects after convalescence of SARS-CoV-2 infection or standard of care.

Secondary objectives

• To assess overall survival, and the overall survival rate at 28 56 and 84 days.

• To assess SARS-CoV-2 viral clearance and load as well as antibody titres.

• To assess the percentage of patients that required mechanical ventilation.

• To assess time from randomisation until discharge.

**Trial design:**

Randomised, open-label, multicenter phase II trial, designed to assess the clinical outcome of SARS-CoV-2 disease in high-risk patients (group 1 to group 4) following treatment with anti-SARS-CoV-2 convalescent plasma or standard of care.

**Participants:**

High-risk patients >18 years of age hospitalized with SARS-CoV-2 infection in 10-15 university medical centres will be included. High-risk is defined as SARS-CoV-2 positive infection with Oxygen saturation at ≤ 94% at ambient air with additional risk features as categorised in 4 groups:

• Group 1, pre-existing or concurrent hematological malignancy and/or active cancer therapy (incl. chemotherapy, radiotherapy, surgery) within the last 24 months or less.

• Group 2, chronic immunosuppression not meeting the criteria of group 1.

• Group 3, age ≥ 50 - 75 years meeting neither the criteria of group 1 nor group 2 and at least one of these criteria:

Lymphopenia < 0.8 x G/l

and/or

D-dimer > 1μg/mL.

• Group 4, age ≥ 75 years meeting neither the criteria of group 1 nor group 2.

Observation time for all patients is expected to be at least 3 months after entry into the study. Patients receive convalescent plasma for two days (day 1 and day 2) or standard of care. For patients in the standard arm, cross over is allowed from day 10 in case of not improving or worsening clinical condition. Nose/throat swabs for determination of viral load are collected at day 0 and day 1 (before first CP administration) and subsequently at day 2, 3, 5, 7, 10, 14, 28 or until discharge. Serum for SARS-Cov-2 diagnostic is collected at baseline and subsequently at day 3, 7, 14 and once during the follow-up period (between day 35 and day 84).

There is a regular *follow-up* of 3 months. All discharged patients are followed by regular phone calls. All visits, time points and study assessments are summarized in the Trial Schedule (see full protocol Table 1).

All participating trial sites will be supplied with study specific visit worksheets that list all assessments and procedures to be completed at each visit. All findings including clinical and laboratory data are documented by the investigator or an authorized member of the study team in the patient's medical record and in the electronic case report forms (eCRFs).

**Intervention and comparator:**

This trial will analyze the effects of convalescent plasma from recovered subjects with SARS-CoV-2 antibodies in high-risk patients with SARS-CoV-2 infection. Patients at high risk for a poor outcome due to underlying disease, age or condition as listed above are eligible for enrollment. In addition, eligible patients have a confirmed SARS-CoV-2 infection and O_2_ saturation ≤ 94% while breathing ambient air. Patients are randomised to receive (experimental arm) or not receive (standard arm) convalescent plasma in two bags (238 - 337 ml plasma each) from different donors (day 1, day 2). A cross over from the standard arm into the experimental arm is possible after day 10 in case of not improving or worsening clinical condition.

**Main outcomes:**

**Primary endpoints:**

The main purpose of the study is to assess the time from randomisation until an improvement within 84 days defined as two points on a seven-point ordinal scale or live discharge from the hospital in high-risk patients (group 1 to group 4) with SARS-CoV-2 infection requiring hospital admission by infusion of plasma from subjects after convalescence of a SARS-CoV-2 infection or standard of care.

**Secondary endpoints:**

• Overall survival, defined as the time from randomisation until death from any cause 28-day, 56-day and 84-day overall survival rates.

• SARS-CoV-2 viral clearance and load as well as antibody titres.

• Requirement mechanical ventilation at any time during hospital stay (yes/no).

• Time until discharge from randomisation.

• Viral load, changes in antibody titers and cytokine profiles are analysed in an exploratory manner using paired non-parametric tests (before – after treatment).

**Randomisation:**

Upon confirmation of eligibility (patients must meet all inclusion criteria and must not meet exclusion criteria described in section 5.3 and 5.4 of the full protocol), the clinical site must contact a centralized internet randomization system (https://randomizer.at/). Patients are randomized using block randomisation to one of the two arms, experimental arm or standard arm, in a 1:1 ratio considering a stratification according to the 4 risk groups (see Participants).

**Blinding (masking):**

The study is open-label, no blinding will be performed.

**Numbers to be randomised (sample size):**

A total number of 174 patients is required for the entire trial, n=87 per group.

**Trial Status:**

Protocol version 1.2 dated 09/07/2020.

A recruitment period of approximately 9 months and an overall study duration of approximately 12 months is anticipated. Recruitment of patients starts in the third quarter of 2020.

The study duration of an individual patient is planned to be 3 months.

After finishing all study-relevant procedures, therapy, and follow-up period, the patient is followed in terms of routine care and treated if necessary.

**Table:** 

Total trial duration:	18 months
Duration of the clinical phase:	12 months
First patient first visit (FPFV):	3^rd^ Quarter 2020
Last patient first visit (LPFV):	2^nd^ Quarter 2021
Last patient last visit (LPLV):	3^rd^ Quarter 2021
Trial Report completed:	4^th^ Quarter 2021

**Trial registration:**

EudraCT Number: **2020-001632-10**, https://www.clinicaltrialsregister.eu/ctr-search/trial/2020-001632-10/DE, registered on 04/04/2020.

**Full protocol:**

The full protocol is attached as an additional file, accessible from the Trials website (Additional file 1). In the interest in expediting dissemination of this material, the familiar formatting has been eliminated; this Letter serves as a summary of the key elements of the full protocol.

The study protocol has been reported in accordance with the Standard Protocol Items: Recommendations for Clinical Interventional Trials (SPIRIT) guidelines (Additional file 2).

The eCRF is attached (Additional file 3).

..

## Supplementary information

**Additional file 1.**

**Additional file 2.**

**Additional file 3.**

## Data Availability

After the trial has been completed and published, it is planned to make trial data available for re- and meta-analyses. An appropriate repository is defined at the end of the trial.

