# Supplementary material for: A Randomized Open label Phase-II Clinical Trial with or without Infusion of Plasma from Subjects after Convalescence of SARS-CoV-2 Infection in High-Risk Patients with Confirmed Severe SARS-CoV-2 Disease (RECOVER): A structured summary of a study protocol for a randomised controlled trial
Source: Trials. 2020 Oct 6;21:828. doi: 10.1186/s13063-020-04735-y (PMC7538058; doi:10.1186/s13063-020-04735-y)
Supplement: Supplementary file 2 — Additional file 2. [file 13063_2020_4735_MOESM2_ESM.docx]

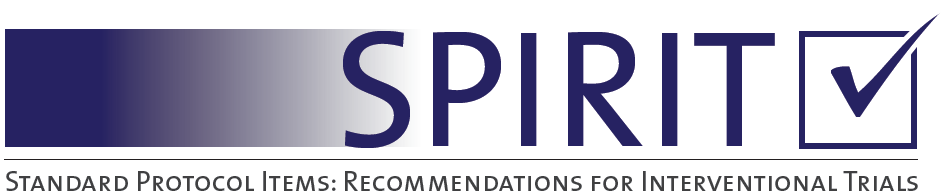


SPIRIT 2013 Checklist: Recommended items to address in a clinical trial protocol and related documents*

| Section/item | | ItemNo | | Description |  |
| --- | --- | --- | --- | --- | --- |
| **Administrative information** | | | | |  |
| Title | | 1 | | **A Randomized Open label Phase-II Clinical Trial with or without Infusion of Plasma from Subjects after Convalescence of SARS-CoV-2 Infection in High-Risk Patients with Confirmed Severe SARS-CoV-2 Disease**  **RECOVER** (Trial acronym) |  |
| Trial registration | | 2a | | **EudraCT Number: 2020-001632-10** |  |
|  |  | 2b | | Not applicable |  |
| Protocol version | | 3 | | Version 1.2. dates 09/07/2020 |  |
| Funding | | 4 | | The trial is co-financed by the BMBF program on emergency research funding for COVID-19 and the funds of Heidelberg University Hospital |  |
| Roles and responsibilities | | 5a | | **Coordinating Investigator**  Prof. Dr. Carsten Müller-Tidow, Internal Medicine V, University Hospital Heidelberg  **Co-Coordinating investigators**  Prof. Dr. Uta Merle, Internal Medicine IV, HD  Prof. Dr. Markus Weigand, Anesthesiology, HD  Dr. Claudia Denkinger, Tropic Medicine, HD  Prof. Dr. Hans-Georg Kräusslich, Infectious Diseases, Virology, HD  Prof. Dr. Maria Vehreschild, Div. Infectious Dis, University Hospital Frankfurt  Prof. Dr. Lars Bullinger, Hematology/Oncology, Charite, Berlin  **Project Management**  Dr. med Ulrike Schäkel, Internal Medicine V  Dr. Carine Djuika Fokou, NCT Trial Center  **Scientific Coordination**  Prof. Dr. Richard F. Schlenk, NCT Trial Center  Prof. Dr. Michael Schmitt, Internal Medicine V  **Biostatistician**  Dr. Johannes Krisam  Institute of Medical Biometry and Informatics, HD |  |
|  |  | 5b | | Ruprecht-Karls-University Heidelberg, Medical Faculty  represented in law by Heidelberg University Hospital  and its Commercial Managing Director Katrin Erk  Im Neuenheimer Feld 672  69120 Heidelberg  Germany |  |
|  | | 5c | | The legal sponsor of the trial is the Ruprecht-Karls-University Heidelberg, Medical Faculty, represented in law by Heidelberg University Hospital, Germany, and its commercial director Katrin Erk. The trial is investigator-initiated, therefore, the main investigator has obtained all rights and duties from the legal sponsor except contract management. The legal sponsor itself does not have a role in collection, management, analysis, and interpretation of data; writing of the report; and the decision to submit the report for publication. |  |
|  | | 5d | | **Trial Coordination** NCT Trial Center Im Neuenheimer Feld 130/3 69120 Heidelberg, Germany  Phone: +49 (0)6221 56 6522 Fax: +49 (0)6221 56 5863  E-Mail: [studienzentrale@nct-heidelberg.de](mailto:studienzentrale@nct-heidelberg.de)  **Data Management**  Christina Klose and Jacek Stermann  Institute of Medical Biometry and Informatics (IMBI) Im Neuenheimer Feld 130/3 69120 Heidelberg, Germany  Phone: +49 (0)6221 56- 37235 Fax: +49 (0)6221 56-4195 E-Mail: stermann@imbi.uni-heidelberg.de  **Clinical Monitoring and Pharmacovigilance**  Coordination Centre for Clinical Trials (KKS)  Heidelberg University Hospital  Im Neuenheimer Feld 130/3 69120Heidelberg, Germany  Phone:+49 (0)6221 56-34507 Fax: +49 (0)6221 56-33508 E-Mail: V-KKS.SAE@med.uni-heidelberg.de  [Karsten.thelen@med.uni-heidelberg.de](mailto:Karsten.thelen@med.uni-heidelberg.de) |  |
| Introduction | |  | |  |  |
| Background and rationale | | 6a | | The SARS-COV-2 infection primarily manifests as mild to moderate respiratory illness in most affected patients. However, older people, and patients with concomitant diseases such as chronic respiratory diseases, cardiovascular diseases, cancers, and/or chronic immunosuppression are at high risk to develop severe respiratory distress ^1-3^. Current control strategies of SARS-CoV-2 disease are limited to prevention, case monitoring and supportive care. A validated vaccine or curative treatment is not available yet, resulting in disease widespread and increasing mortality ^4^. Outbreaks have overwhelmed the health care systems in several regions of the world. Patients requiring mechanical ventilation were not intubated due to shortages in ventilators, material and staff ^5^. Accordingly, early effective treatment is urgently required to avoid the need for mechanical ventilation.  Although the therapeutic effect of COVID-19 CP is not well known, previous reports suggest that neutralizing antibodies present in the sera may suppress viremia, when transferred to SARS-CoV-2-infected patients – leading to an improvement of symptoms, a shorter duration of hospitalisation and reduced mortality compare to patients not treated with CP ^7,8,9^. Furthermore, infusion of CP at an early stage of other coronavirus infections (H1N1 and SARS) has been associated with high effectiveness ^6,8,10,11^. Similar findings provide a meta-analysis of CP treatment during the Spanish influenza ^10^. Results of a recent study investigating 20 patients treated with CP suggests that in critically ill patients with Sars-Cov-2 infections early administration of CP may improve survival^12^.  In contrast, CP administered later in the course of COVID-19 (median of 21.5 days after diagnosis) has limited impact^13^. This is likely due to the fact that viral loads peak in the first week for most viral infections, and deterioration in the subsequent clinical course of illness is mostly attributable to the inflammatory reaction in response to the virus, which leads to destruction of lung tissue and acute respiratory distress syndrome.  Thus, we anticipate that early CP treatment of high-risk COVID-19 patients results in a favorable outcome in high-risk patient populations.  Moreover, other than in vaccination, passive antibody therapy provides an immediate protection. This is of particular importance in an infectious outbreak context, especially for high-risk patients who might not develop a prompt and adequate immune response due to underlying disease or concomitant therapy.  Therapy with plasma is considered to be save as a recent meta-analyses of >5000 people in the U.S. highlighted^14^. |  |
|  | | 6b | | Not applicable. |  |
| Objectives | | 7 | | Primary objectives   - To assess the time from randomisation until an improvement within 84 days defined as two points on a seven point ordinal scale or live discharge from the hospital in high-risk patients with SARS-CoV-2 infection requiring hospital admission by infusion of plasma from subjects after convalescence of SARS-CoV-2 infection or standard of care.   Secondary objectives   - To assess overall survival, and the overall survival rate at 28, 56 and 84 days. - To assess SARS-CoV-2 viral clearance and load as well as antibody titres. - To assess percentage of patients that required mechanical ventilation. - To assess time from randomisation until discharge |  |
| Trial design | | 8 | | Randomised, open-label, multicentre phase II trial, designed to assess the clinical outcome of SARS-CoV-2 disease in high-risk patients following treatment with anti-SARS-CoV-2 convalescent plasma or standard of care. |  |
| Methods: Participants, interventions, and outcomes | | | | |  |
| Study setting | | 9 | | Multi-centre trial conducted in several German university medical centres (10 - 15). A list of participating centres is not completed yet updates can be obtained on the NCT study centre website. |  |
| Eligibility criteria | | 10 | | \| **Inclusion Criteria**   1. PCR confirmed SARS-CoV-2 infection in a respiratory tract   sample.   1. Oxygen saturation (SaO_2_) of 94% or less while breathing   ambient air or a ratio of the partial pressure of oxygen (PaO_2_) to  the fraction of inspired oxygen (FiO_2_) of less than 300 mm Hg.   1. High risk due to either pre-existing or concurrent hematological   malignancy and/or active cancer therapy (incl. chemotherapy,  radiotherapy, surgery) within the last 24 months or less.  (group 1)  and/or  chronic immunosuppression not meeting the criteria of group 1  (group 2)  and/or Age ≥ 50 - 75 years meeting neither the criteria of group 1  nor group 2 (group 3) and at least one of these criteria: Lymphopenia < 0.8 x G/l and/or D-dimer > 1µg/mL  and/or Age ≥ 75 years meeting neither the criteria of group 1 nor  2 (group 4)   1. Blood hemoglobin concentration ≥ 10g/dl. 2. Provision of written informed consent. 3. Patient is able to understand and comply with the protocol for   the duration of the study, including treatment and scheduled  visits and examinations.   1. Male or female patient aged ≥ 18 years. 2. Postmenopausal or evidence of non-childbearing status.   For women of childbearing potential: negative urine or serum  pregnancy test within 14 days prior to study treatment. \| \| --- \| \| **Exclusion Criteria**   1. Dementia, psychiatric or cognitive illness or recreational 2. drug/alcohol use that in the opinion of the principle investigator,   would affect subject safety and/or compliance.   1. Contraindication to transfusion or history of prior reactions to   transfusion blood products.   1. Patients with known selective IgA deficiency. 2. Patients with mechanical ventilation and/or extracorporal   membrane oxygenation (ECMO) at time of initial inclusion into  the trial.   1. Participation in another trial with an investigational medicinal   product.   1. Treatment with SARS-CoV-2 convalescent plasma in the past. \| |  |
| Interventions | | 11a | | Patients are randomised to receive (experimental arm) or not receive (standard arm) convalescent plasma in two bags (238 - 337 ml plasma each) from different donors who recovered from COVID19 (day 1, day 2). |  |
|  |  | 11b | | A crossover of patients from the standard arm into the experimental arm is possible on day 10 if no improvement or worsening of the clinical situation occurs.  Cross-over into the experimental arm is intended for patients randomised into the standard arm if the following criteria is met:   - Starting from day 10 after randomisation - No improvement or worsening of clinical condition   For cross-over, all inclusion / exclusion criteria (except mechanical ventilation/ECMO) must again be met.  The actual possibility for cross-over into the experimental arm is based on discussion with the coordinating investigator on a case-by-case decision.  Definition of conditions leading to CP treatment discontinuation:   - **Adverse event with the transfusion of CP**: continuation of CP treatment after AE resolution to < grade 2 according to CTCAE - **Severe allergic reactions or anaphylactic shock / serious adverse event upon CP transfusion:** transfusion must be stopped immediately and no further CP treatment is applied. A second CP administration is skipped.   A patient must be withdrawn from the trial treatment or/and all trial-related procedures for the following reasons:   1. At any time at their own request withdrawal of patient’s consent to continue therapy. The patient is at any time free to discontinue treatment, without prejudice to further treatment. 2. Changes in medical status of the patient such that the investigator believes that patient safety is compromised or that it would be in the best interest of the patient to stop treatment 3. Pregnancy   A patient may be withdrawn from the trial treatment or/and all trial-related procedures for the following reasons:   1. Non-compliance by the patient with protocol requirements 2. Patient is lost to follow-up. If a patient does not return for scheduled visits, every effort should be made to re-establish contact. In any circumstance, every effort should be made to document patient outcome if possible |  |
|  |  | 11c | | Not applicable. |  |
|  |  | 11d | | The addition of what is considered standard of care is allowed. No medication must be discontinued during the trial. |  |
| Outcomes | | 12 | | **Primary endpoint and primary estimand**  The main purpose of the study is to assess the time from randomisation until an improvement within 84 days defined as two points on a seven point ordinal scale or live discharge from the hospital in high-risk patients (group 1 to group 4) with SARS-CoV-2 infection requiring hospital admission by infusion of plasma from subjects after convalescence of a SARS-CoV-2 infection or standard of care.  In the Addendum to the ICH E9 guideline (final version), the estimands framework is recommended as clear and transparent definition of “what is to be estimated” (International Council for Harmonization 2019). An estimand is defined through the treatment, the targeted population, the variable, a specification of how to handle intercurrent events (postrandomisation events) and a population-level summary. In the following, the primary estimand corresponding to the primary objective is described.  The primary estimand corresponding the primary objective is defined as follows:  **Treatment:** Infusion of frozen CP (on two days of intervention from two different donors) vs. standard of care.  **Population**: The targeted population is defined through the in- and exclusion criteria.  **Variable**: Time from randomisation to clinical improvement within 84 days by two points on a seven-point ordinal scale or live discharge from the hospital.  **Post-randomisation events**: live discharge from the hospital is incorporated into the variable definition (composite strategy), death from any cause within 84 days after randomisation without previous improvement is taken into account by censoring deceased patients at day 84 (see protocol sections 10.3 and 10.5.4 for rationale); treatment switch will be ignored (treatment policy strategy, see protocol sections 10.3 and 10.5.4 for rationale); event-free patients at the end of the follow-up period are censored and drop-outs are censored at the last observation (hypothetical strategy).  **Summary measure**: Hypothesis testing is conducted using a log-rank test. The summary measure for effect quantification is the hazard ratio for the endpoint “clinical improvement by two points or live discharge” between the two treatment arms (which is estimated using Cox regression).  **Secondary Endpoints**  Overall survival, defined as the time from randomisation until death from any cause.  28-day, 56-day and 84-day overall survival rates.  SARS-CoV-2 viral clearance and load as well as antibody titres.  Requirement mechanical ventilation at any time during hospital stay (yes/no).  Time until discharge from randomisation.  Viral load, changes in antibody titers and cytokine profiles are analysed in an exploratory manner using paired non-parametric tests (before – after treatment).    **Safety Endpoints**  This includes all AEs, their severity, SAEs, the relation of AEs to the study treatment, dose modifications for toxicity and discontinuation of study treatment during the trial phase. Toxic effects are graded according to the National Cancer Institute Common Toxicity Criteria (CTCAE) version 5.0. |  |
| Participant timeline | | 13 | | The duration of the trial for each patient is expected to be about 3 months, including two days of intervention (infusion of frozen CP), followed by a follow-up of 3 months. Furthermore, viral load is measured daily in nasopharagyngeal swabs at day 1, 2, 3, 5, 7, 10, 14, 28 or until hospital discharge within 84 days after randomisation (See figure 1).  Patients randomised into the standard arm of the study have the possibility to cross over into the experimental arm of the study starting from day 10 in case of not improving or worsening clinical condition.  All visits, time points and study assessments are summarised in the Trial Schedule (see Protocol Table 1).  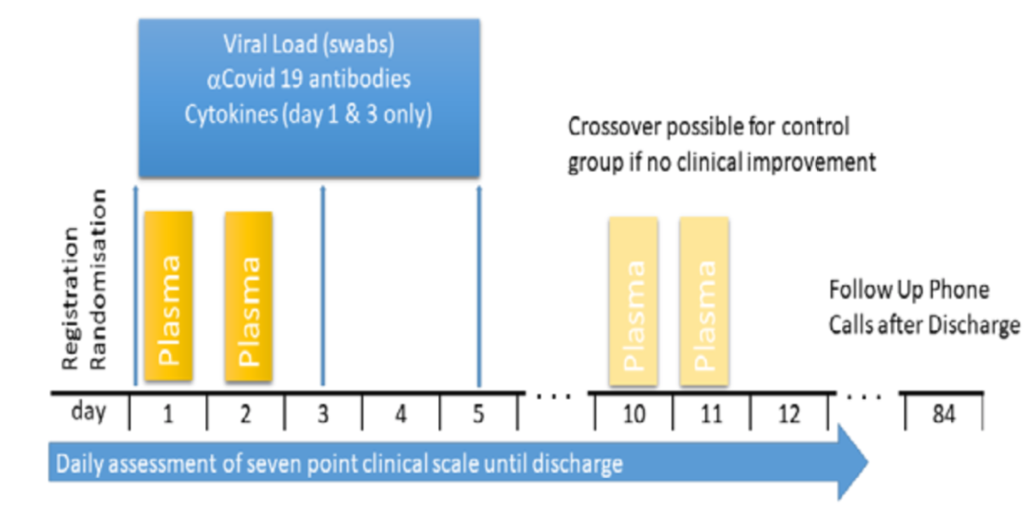 |  |
|  | |  | | Figure 1: Participant Timeline | |
| Sample size | | 14 | | To detect the assumed difference between treatment groups using a log-rank test comparing the cumulative improvement curves for the primary endpoint using a significance level of 5% (two-sided) with a power of 80%, a total number of 174 patients is required for the entire trial (87 patients per group) when additionally considering a dropout rate of 5%, meaning that n=164 patients who do not prematurely drop out of the study are required to be enrolled. The required number of events which was calculated using the formula by Schoenfeld, amounts to 142. It is expected that adjusting for the covariate “patient group” in the analysis will lead to an increase in power. Sample size calculation was done conservatively assuming an equally long follow-up period of 38 days for every patient. Sample size calculation was done using RPACT v 2.0.6.  For the sample size calculation, it was assumed that there would be a consistent median time to improvement and overall survival rate across all 4 patient groups. Since this might not necessarily be the case, and it could be likely that patients in group 1 and 2 might have a shorter median time to improvement, we conducted a simulation study to explore the robustness of our model under varying assumptions for the median event times. For our simulation study, we assumed exponentially distributed event times, with a median time to improvement of 16 days across all patient groups in the control group (med_C_^1^= med_C_^2^ = med_C_^3^ = med_C_^4^=16_)_, while we assumed differing median times to improvement in the patient groups 1 & 2 compared to patient groups 3 & 4 (med_E_^1^= med_E_^2^ , med_E_^3^ = med_E_^4^_)_ in the experimental group. We considered a sample size of n=164 evaluable patients for the whole trial, with 42 patients belonging to groups 1 and 3 each, and 40 patients belonging to groups 2 and 4 each corresponding to patient group membership probability of about 25% for all four groups. Also, we assumed a 38-day mortality rate of 20% in the control arm, while we assumed that the corresponding mortality rate in the experimental arm amounted to 13% (corresponding to a HR for overall survival of 1.6) across all four patient strata assuming exponentially distributed survival times. We simulated 10,000 trials per scenario corresponding to a maximum standard error of sqrt(0.5*0.5/10000)=0.005 for the simulated power.  We used a stratified log-rank test to assess whether the cumulative improvement curves in the two treatment groups (experimental/control) would be equal at a two-sided significance level of α=0.05 stratifying for the factor “patient group” for analysis. The analysis of the simulated datasets was done using the PROC LIFETEST procedure in SAS v9.4. The results are shown in Table 3 (see Protocol pg. 40). |  |
| Recruitment | | 15 | | 10-15 trial cites will include patients. |  |
| **Methods: Assignment of interventions (for controlled trials)** | | | | |  |
| Allocation: | |  | |  |  |
| Sequence generation | | 16a | | A computer-generated randomisation list was created. Participants are assigned random numbers based on consecutive enrolment. |  |
| Allocation concealment mechanism | | 16b | | A subject is considered enrolled when he or she has signed the Informed consent form. The patient receives a screening number at the clinical site (number of site plus number of patient in ascending order, e. g. 001-001 for the first enrolled patient at site 01) via registration in the eCRF system (www.xxx.). The screening number is used to identify the subject throughout the clinical study and must be used on all study documentation related to the subject.  Clinical sites must complete the baseline case report forms for all enrolled subjects, even if the subject is not randomised or treated in this study.  Upon confirmation of eligibility (patients must meet all inclusion criteria and must not meet exclusion criteria), the clinical site must contact a centralised internet randomisation system (https://randomizer.at/). Patients are randomised using block randomisation to one of the two arms, experimental arm or standard arm, in a 1:1 ratio considering a stratification according to the 4 risk groups (as described in the inclusion criteria).  All subjects randomised receive a unique randomisation number. All patients in the experimental group have to start CP treatment within 7 days of randomisation. Patients withdrawn from the trial retain their Patient ID and randomisation number.  The study is open-label. |  |
| Implementation | | 16c | | Generation of allocation sequence, enrolment of participants, and assignment of participants to interventions is performed in the NCT Trial Center, Heidelberg University Hospital, Germany. |  |
| Blinding (masking) | | 17a | | Not applicable |  |
|  | | 17b | | Not applicable |  |
| **Methods: Data collection, management, and analysis** | | | | |  |
| Data collection methods | | 18a | | All findings including clinical and laboratory data are documented by the investigator or an authorised member of the study team in the patient's medical record and in the electronic case report forms (eCRFs). The investigator at the clinical site is responsible for ensuring that all sections of the eCRFs are completed correctly and that entries can be verified against source data. The eCRFs have to be filled out according to the specified CRF Completion Guidelines. The correctness of entries in the eCRFs is confirmed by dated signature of the responsible local principal investigator or deputy principal investigator. |  |
|  | | 18b | | In all cases, the reason for withdrawal must be recorded in the eCRF and in the patient’s medical records. In case of withdrawal of a patient at his/ her own request, the reason should be asked for as extensively as possible and documented.  For patients with incomplete follow-up, time to last follow-up date is used as the censoring time in the analysis of time-to-event data. |  |
| Data management | | 19 | | Data entries undergo an automatic online check for plausibility and consistency. In case of implausibility, 'warnings' are produced. A responsible investigator is obliged either to correct the implausible data or to confirm its authenticity, and to give appropriate explanation. If not corrected, the data is flagged, enabling a convenient check of all questionable entries. A responsible monitor checks all flagged data and generates questions that are sent back to the responsible investigator. The investigator has to resolve all 'discrepancies'.  Further checks for plausibility, consistency, and completeness of data are performed after completion of the study. Queries are generated on the basis of these checks, combined with a visual control by a responsible monitor/data manager.  All missing data or inconsistencies are reported back to the sites and clarified by the responsible investigator. If no further corrections are to be made in the trial database it is declared closed and used for statistical analysis.  All data management activities are done according to the current Standard Operating Procedures (SOPs) of the IMBI. |  |
| Statistical methods | | 20a | | **Primary endpoints**  The primary analysis will assess the null hypothesis “the cumulative improvement curves for the primary endpoint in the experimental and control arm are equal”, i.e. H_0_: S_E_ = S_C_ against the alternative hypothesis H_1_: S_E_ ≠ S_C_ at a two-sided significance level of α=5%. This will be achieved by using the log-rank test stratified for the factor “patient group” as used in the randomisation procedure. The event “death from any cause” will be handled by censoring those patients at day 84 (in analogy to the approach of Cao et al, NEJM 2020). Using this approach ensures that deceased patients are considered as “not improved” over the whole observation period of 84 days. The hazard ratio for treatment group will be determined using a Cox regression model stratifying for the factor “patient group” together with a 95% confidence interval. The cumulative improvement curves are calculated using the Kaplan-Meier method together with 95% log-log-type confidence bands and will be calculated separately for both treatment groups. In addition, cumulative improvement curves will also be calculated separately for each patient group per treatment group.  The fact that some patients from the control group might switch to the experimental group at day 10 will be ignored in the primary analysis.  **Secondary endpoints**  Overall survival is analysed by using a Cox regression model adjusting for treatment and patient group, determining hazard ratios with 95% confidence intervals and (descriptive) p-values. Survival estimates are calculated using the Kaplan-Meier method together with 95% log-log-type confidence bands, and the 28-day survival rate will be given for both treatment groups together with 95% confidence intervals. Time until discharge will be assessed similarly to the primary endpoint, providing Kaplan-Meier estimates for the cumulative discharge rate for both treatment groups and conducting a log-rank test stratified for the factor “patient group”, and censoring patients who died from any cause at day 84 in analogy to the analysis of the primary endpoint.  The secondary endpoint “requirement of mechanical ventilation (yes/no)” is analysed by means of a logistic regression model adjusting for the factors treatment and patient group. Also, absolute and relative frequencies will be given for this endpoint, together with 95% confidence intervals.  The secondary endpoints SARS-CoV-2 viral load and antibody titres will be assessed over time by means of linear mixed models adjusting for treatment group, patient group, and baseline value as fixed factors, as well as time as repeated factor using an unstructured covariance matrix, calculating least square means estimates and 95% confidence intervals. |  |
|  | | 20b | | A supplementary analysis of the primary endpoint will involve the inverse probability censoring weighting (IPCW) approach to estimate a treatment effect in the hypothetical scenario that patients from the control group had not switched to the experimental group. This type of model requires to estimate the probability for a patient to switch to the experimental group based on (time-dependent) covariates, which in our case will be the baseline factor “patient group”, together with the time-varying factors “seven point ordinal scale measuring clinical improvement” and “viral load” over time. It should be noted that this model implicitly assumes that all relevant confounders predicting whether a patient switches from control to treatment have been included into the model, an assumption which is hardly verifiable, especially for such a recently emerged disease about which we currently only have limited understanding, thus being the reason why we did not choose this as the strategy for the primary analysis.  As another supplementary analysis intending to estimate the treatment effect in the hypothetical scenario which assumes that control patients had not switched to the treatment group, a rank preserving structural failure time (RPSFT) model will be fitted. |  |
|  | | 20c | | The Full Analysis Population (FAP) includes all randomised patients with treatment groups assigned in accordance with the randomisation, regardless of the treatment actually received. Patients who were randomised but did not subsequently receive treatment are included in the Full analysis population. The analysis of data using the Full analysis population therefore follows the Intention-to-Treat principles (ITT).  In the per protocol population, patients with important protocol deviations are excluded. Definition of important protocol deviations are given in the statistical analysis plan (SAP).  All enrolled patients who received treatment are subjected to the safety analysis. Patients will be evaluated with regard to the treatment actually received, meaning that patients who cross over from the control to the experimental arm will be evaluated in the experimental arm from day 10 onwards. Details of the safety analysis are specified in the statistical analysis plan (SAP).  **Handling with missing data**  For patients with incomplete follow-up, time to last follow-up date is used as the censoring time in the analysis of time-to-event data. Missing data of continuous outcomes over time will be handled via the multi-level approach making the implicit assumption that data are missing at random, thus not requiring any direct imputation of missing continuous data. The robustness of this assumption will be explored in sensitivity analyses by means of pattern mixture models assuming that data are not missing at random. Otherwise, no imputation of missing data will be conducted. |  |
| **Methods: Monitoring** | | | | |  |
| Data monitoring | | 21a | | The DMC is composed of at least three independent experts, assessing the progress and safety data. The mission of the DMC is to ensure the ethical conduct of the trial and to protect the safety interests of patients in this trial.  The DMC meetings are planned after treatment of 5, 10 and 25 patients in the experimental arm of the study and according to the DMC charter. Based on its review, the DMC provides the sponsor with recommendations.  Further details including DSUR reviews and DMC members is specified in the DMC charter. |  |
|  | | 21b | | The trial can be prematurely closed or suspended by the Sponsor after consulting the Coordinating Investigator. The Ethics Committee (EC) and the Competent Regulatory Authorities must then be informed. Furthermore, the Ethics Committee(s) and Competent Regulatory Authorities themselves may decide to stop or suspend the trial.  Should the trial be closed prematurely, all trial material (completed, partially completed, and blank CRFs, investigational medicinal product and other material) must be returned to the Sponsor in Heidelberg or treated according Sponsor notice.  All involved investigators have to be informed immediately about a cessation/suspension of the trial. The decision is binding to all trial centres and investigators. |  |
| Harms | | 22 | | All AEs reported by the patient or detected by the investigator are collected during the trial. AEs must also be documented in the patient’s medical records. Whenever possible, the investigator records the main diagnosis instead of the signs and symptoms normally included in the diagnoses.  In this trial, **all AEs that occur after signature of the informed consent** are documented on the pages provided in the CRF. AEs must be followed up from first plasma administration up to 28 days after last plasma administration or until all drug-related toxicities have been resolved, whichever is later, or until the investigator assesses AEs as “chronic” or “stable”. Each AE must be reported, indicating the worst CTC (Version 5.0) grade. If an event stops and later restarts, all occurrences must be reported. A specific procedure for definition and reporting of SAEs is described in Protocol Section 9.3. |  |
| Auditing | | 23 | | The investigators agree to allow the auditors/inspectors/monitors to have direct access to the trial records for review, being understood that these personnel is bound by professional secrecy, and as such will not disclose any personal identity or personal medical information. The investigator will make every effort to help with the performance of the audits and inspections, giving access to all necessary facilities, data, and documents. |  |
| Ethics and dissemination | | | | |  |
| Research ethics approval | | 24 | | The investigators submit the required documents to the responsible Ethics Committee (EC) of the Medical Faculty of Heidelberg University, Germany, and obtains the opinion of the Committee in writing. Participants will not be included until unconditional approval of EC has been received. |  |
| Protocol amendments | | 25 | | If the trial protocol has to be changed substantially after approval, a written amendment is required that must be signed by the same persons as mentioned in the trial protocol. Any protocol amendment will only be implemented after approval has been granted by the EC and the competent authorities. Any substantial protocol amendment affecting the benefit-to-risk ratio must be approved by the responsible EC must be notified to the local regulatory authority (Regierungspräsidium Karlsruhe). These procedures must be completed before any modifications can come into operation, except when they are necessary to eliminate immediate hazards for the trial participants. Participants will be informed about relevant changes in the trial and will be asked to re-consent in writing. |  |
| Consent or assent | | 26a | | Before being admitted to the clinical trial, the participant must consent to participate after being fully informed by the investigator or a designated member of the investigating team about the nature, importance, risks and individual consequences of the clinical trial and their right to terminate the participation at any time. |  |
|  | | 26b | | Not applicable |  |
| Confidentiality | | 27 | | The data obtained in the course of the trial is treated pursuant to the applicable Data Protection Law (EU General Data Protection Regulation – GDPR ‑ 2016/679), the Federal Data Protection Act (Bundesdatenschutzgesetz, BDSG), the State Data Protection Act of Baden-Württemberg (Landesdatenschutzgesetz, LDSG BW) as well as § 40 (2a) AMG.  During the clinical trial, patients are identified solely by means of an individual identification code (Patient ID). Storage of trial findings on a computer are done in accordance with local data protection law and are handled in strictest confidence. For protection of these data, organisational procedures are implemented to prevent distribution of data to unauthorised persons. The appropriate regulations of local data legislation are fulfilled in its entirety.  The patient consents in writing to relieve the investigator from his/her professional discretion in so far as to allow inspection of original data for monitoring purposes by health authorities and authorised persons (inspectors, monitors, auditors). Authorised persons (clinical monitors, auditors, inspectors) may inspect the patient-related data collected during the trial, ensuring the data protection law.  The investigator maintains a patient identification list (Patient IDs with the corresponding patient names) to enable records to be identified.  Patients who did not consent to circulate their pseudonymised data are not included into the trial. |  |
| Declaration of interests | | 28 | | There are no financial and other competing interests for the principal investigator or deputy investigators. |  |
| Access to data | | 29 | | After the trial has been completed and published, it is planned to make trial data available for re- and meta-analyses. An appropriate repository is defined at the end of the trial. |  |
| Ancillary and post-trial care | | 30 | | The sponsor has to subscribe to an insurance policy covering, in its terms and provisions, its legal liability for injuries caused to participating persons and arising out of this research performed strictly in accordance with the scientific protocol as well as with applicable law and professional standards. |  |
| Dissemination policy | | 31a | | All information concerning the trial is confidential before publication.  Trial results will be published in medical journals. |  |
|  | | 31b | | Authorship eligibility is based on the following criteria (ICMJE):   - Substantial contributions to the conception or design of the work;   or the acquisition, analysis, or interpretation of data for the work; AND   - Drafting the work or revising it critically for important intellectual content; AND - Final approval of the version to be published; AND - Agreement to be accountable for all aspects of the work in ensuring that questions related to the accuracy or integrity of any part of the work are appropriately investigated and resolved.   There is no intended use of professional writers. |  |
|  | | 31c | | Not applicable |  |
| Appendices | |  | |  |  |
| Informed consent materials | | 32 | | Not applicable, only in German |  |
| Biological specimens | | 33 | | Not applicable |  |

*It is strongly recommended that this checklist be read in conjunction with the SPIRIT 2013 Explanation & Elaboration for important clarification on the items. Amendments to the protocol should be tracked and dated. The SPIRIT checklist is copyrighted by the SPIRIT Group under the Creative Commons “[Attribution-NonCommercial-NoDerivs 3.0 Unported](http://www.creativecommons.org/licenses/by-nc-nd/3.0/)” license.

References

1 Yu, J., Ouyang, W., Chua, M. L. K. & Xie, C. SARS-CoV-2 Transmission in Patients With Cancer at a Tertiary Care Hospital in Wuhan, China. *JAMA Oncol*, doi:10.1001/jamaoncol.2020.0980 (2020).

2 Liang, W. *et al.* Cancer patients in SARS-CoV-2 infection: a nationwide analysis in China. *Lancet Oncol* **21**, 335-337, doi:10.1016/s1470-2045(20)30096-6 (2020).

3 Wu, Z. & McGoogan, J. M. Characteristics of and Important Lessons From the Coronavirus Disease 2019 (COVID-19) Outbreak in China: Summary of a Report of 72314 Cases From the Chinese Center for Disease Control and Prevention. *Jama*, doi:10.1001/jama.2020.2648 (2020).

4 UpToDate.com. [https://www.uptodate.com/contents/coronavirus-disease-2019-covid-19#](https://www.uptodate.com/contents/coronavirus-disease-2019-covid-19). (2020).

5 Rosenbaum, L. Facing Covid-19 in Italy - Ethics, Logistics, and Therapeutics on the Epidemic's Front Line. *N Engl J Med*, doi:10.1056/NEJMp2005492 (2020).

6 Hung, I. F. *et al.* Convalescent plasma treatment reduced mortality in patients with severe pandemic influenza A (H1N1) 2009 virus infection. *Clin Infect Dis* **52**, 447-456, doi:10.1093/cid/ciq106 (2011).

7 Shen, C. *et al.* Treatment of 5 Critically Ill Patients With COVID-19 With Convalescent Plasma. *Jama*, doi:10.1001/jama.2020.4783 (2020).

8 Cheng, Y. *et al.* Use of convalescent plasma therapy in SARS patients in Hong Kong. *Eur J Clin Microbiol Infect Dis* **24**, 44-46, doi:10.1007/s10096-004-1271-9 (2005).

9 Casadevall, A. & Pirofski, L. A. The convalescent sera option for containing COVID-19. *J Clin Invest*, doi:10.1172/jci138003 (2020).

10 Soo, Y. O. *et al.* Retrospective comparison of convalescent plasma with continuing high-dose methylprednisolone treatment in SARS patients. *Clin Microbiol Infect* **10**, 676-678, doi:10.1111/j.1469-0691.2004.00956.x (2004).

11 Luke, T.C. *et al.* Meta-analysis: convalescent blood products for Spanish influenza pneumonia: a future H5N1 treatment? *Ann Intern Med.* **145**, 599-609 (2006).

12 Hegerova, L. et al. Use of convalescent plasma in hospitalized patients with COVID-19: case series. *Blood.* **136**, 159-162 (2020).

13 Zeng, Q.L. *et al*. Effect of convalescent plasma therapy on viral shedding and survival in patients with coronavirus disease 2019. *J Infect Di*s **222**, 38-43 (2020).

14 Joyner M.J. *et al*. Early safety indicators of COVID-19 convalescent plasma in 5000 patients. *J Clin Invest,* doi :10.1172/JCI140200 (2020).
