# Supplementary material for: A Randomized Open label Phase-II Clinical Trial with or without Infusion of Plasma from Subjects after Convalescence of SARS-CoV-2 Infection in High-Risk Patients with Confirmed Severe SARS-CoV-2 Disease (RECOVER): A structured summary of a study protocol for a randomised controlled trial
Source: Trials. 2020 Oct 6;21:828. doi: 10.1186/s13063-020-04735-y (PMC7538058; doi:10.1186/s13063-020-04735-y)
Supplement: Supplementary file 3 — Additional file 3. [file 13063_2020_4735_MOESM3_ESM.pdf]

CRF

Centre ID	
Patient registration	
Patient ID	
Date of written informed consent	____ ______ ________

Baseline Visit

Day 1 -10

Day 1

Day 2

Day 3

Day 4

Day 5

Day 6

Day 7

Day 8

Day 9

Day 10

Day 1 -10 -- Cross-over Assessment

Day 1 - Cross-over Assessment

Day 2 - Cross-over Assessment

Day 3 - Cross-over Assessment

Day 4 - Cross-over Assessment

Day 5 - Cross-over Assessment

Day 6 - Cross-over Assessment

Day 7 - Cross-over Assessment

Day 8 - Cross-over Assessment

Day 9 - Cross-over Assessment

Day 10 - Cross-over Assessment

Day 11 - Cross-over Assessment

Day 12 - Cross-over Assessment

Day 13 - Cross-over assessment

Day 14 - Cross-over assessment

Day 15 - Cross-over assessment

Day 11 -28

Day 11

Day 12

Day 13

Day 14

Day 15

Day 16

Day 17

Day 18

Day 19

Day 20

Day 21

Day 22

Day 23

Day 24

Day 25

Day 26

Day 27

Day 28

Follow-Up period

Day 35

Day 42

Day 49

Day 56

Day 70

Day 84

Global Pages

Principal Investigator's Signature

Meaning: I confirm the completeness and correctness of all documented data.

Signed By:

Signature Date:

Baseline Visit

Centre ID	
Patient ID	
Visit	Baseline

- Eligibility
- Demography
- Medical history
- Concomitant medication
- Smoking history
- Signs/Symptoms
- Vital signs
- Physical examination
- Seven point ordinal scale
- SOFA-Score
- Oxygen saturation
- Echocardiogram
- ECG
- Hematology
- Blood chemistry and coagulation
- Urinalysis
- Other Virology
- Pregnancy test
- SARS-CoV-2 viral clearance and load as well as antibody titres
- Enrollment/Randomization

Investigator's Signature

Meaning: I confirm the completeness and correctness of all documented data.

Signed By:

Signature Date:

Eligibility

Centre ID	
Patient ID	
Visit	

Inclusion criteria

All inclusion criteria fulfilled	◯yes ◯no
1. PCR confirmed SARS-CoV-2 infection in a respiratory sample	◯yes ◯no
2. Oxygen saturation (SaO2) of 94% or less while breathing ambient air or a ratio of the partial pressure of oxygen (PaO2) to the fraction of inspired oxygen (FiO2) of less than 300 mm Hg	◯yes ◯no
3. High risk due to either	◯yes ◯no
pre-existing or concurrent hematological malignancy and/or active cancer therapy (incl. chemotherapy, radiotherapy, surgery) within the last 24 months or less (group 1)	◯yes
chronic immunosupression not meeting the criteria of group 1 (group 2)	◯yes
Age ≥ 50 - 75 years meeting neither the criteria of group 1 nor group 2 (group 3) and at least one of these criteria: Lymphopenia < 0.8 x G/l and/or D-dimer > 1µg/mL	◯yes
Age ≥ 75 years meeting neither the criteria of group 1 nor group 2 (group 4)	◯yes
4. Blood hemoglobin concentration ≥ 10 g/dl	◯yes ◯no
5. Provision of written informed consent	◯yes ◯no
6. Patient is able to understand and comply with the protocol for the duration of the study, including treatment and scheduled visits and examinations	◯yes ◯no
7. Male or female patient aged ≥ 18 years	◯yes ◯no
8. Postmenopausal or evidence of non-childbearing status. For women of childbearing potential: negative urine or serum pregnancy test within 14 days prior to study treatment	◯yes ◯no

Exclusion criteria

None of the exclusion criteria is fulfilled	◯yes ◯no
1. Dementia, psychiatric or cognitive illness or recreational drug/alcohol use that in the opinion of the principal investigator, would affect subject safety and/or compliance	◯yes ◯no
2. Contraindication to transfusion or history of prior reactions to transfusion blood products	◯yes ◯no
3. Patients with known selective IgA deficiency	◯yes ◯no
4. Patients with mechanical ventilation and/or extracoporal membrane oxygenation (ECMO) at time of initial inclusion into the trial.	◯yes ◯no
5. Participation in another trial with an investigational medicinal product	◯yes ◯no
6. Treatment with SARS-CoV-2 convalescent plasma in	◯yes ◯no

	the past	◯yes ◯no

Demography

Centre ID	
Patient ID	
Visit	
Sex	◯male ◯female
Year of birth	____________________
Childbearing potential	◯yes ◯no
Please specify reason	◯Permanent sterilization methods (hysterectomy bilateral salpingectomy or bilateral oophorectomy)<br>◯Naturally postmenopausal for at least 24 consecutive months
Ethnic origin	◯Asian<br>◯Black<br>◯Caucasian/White<br>◯Hispanic<br>◯Other
If other: Please specify	____________________

Medical history

Centre ID	
Patient ID	
Visit	

Anamnesis

Chronic lung disease	▼
if other: Please specify	
Cardiovascular disease	▼
if other: Please specify	
Chronic liver disease	▼
if other: Please specify	
Rheumatic / immunologic disease	▼
if other: Please specify	
Organ transplant	▼
Diabetes	▼
Chronic neurological or psychiatric disease?	▼
Cancer	◯No ◯Active ◯In remission
Cancer entity	
Date of first diagnosis	
Chemotherapy	◯yes ◯no
Start of Chemotherapy	
Radiotherapy	◯yes ◯no
Start of Radiotherapy	
Surgery	◯yes ◯no
Date of surgery	
Chronic kidney disease	▼
vaccination	▼
if Other: Please specify	
Was there oxygen or ventilation therapy before this study?	◯yes ◯no ◯not known
Travel	◯yes ◯no
Location	
Start of travel	
Stop of travel	
Gastric ulcer	◯yes ◯no ◯not known
DNR-Status	◯yes ◯no ◯not known

Date of first symptoms ____ ______ ________

Type of first symptoms

Olfactory or taste disorder	◯yes ◯no ◯not known
Abdominal pain	◯yes ◯no ◯not known
Confusion	◯yes ◯no ◯not known
Diarrhoea	◯yes ◯no ◯not known
Vomitting	◯yes ◯no ◯not known
Cough	◯yes ◯no ◯not known
Nausea	◯yes ◯no ◯not known
Fever	◯yes ◯no ◯not known
Headache	◯yes ◯no ◯not known
Dyspnea	◯yes ◯no ◯not known
Has the patient knowingly been in contact with a person who is likely or proven to have COVID-19 in the 14 days prior to the onset of their symptoms?	◯yes ◯no ◯not known

Medical imaging

Was a medical imaging realised?	◯yes ◯no ◯not known
Medical imaging of the lung	◯CT<br>◯X-ray<br>◯Ultrasound (US)
Result of imaging	◯Unspecific result<br>◯COVID-typical result<br>◯Normal result

Any prior therapy regarding COVID-19 done? ◯yes ◯no ◯not known

If 'yes' then add details of prior therapy regarding COVID-19

Previous dialysis / Hemofiltration	◯yes ◯no
Start date of dialysis	____ ______ ________
Is the dialysis at the start of study ongoing?	◯yes ◯no
Stop date of dialysis	____ ______ ________
Previous apheresis	◯yes ◯no ◯not known
Start date of apheresis	____ ______ ________
Is the apheresis at the start of study ongoing?	◯yes ◯no
Stop date of apheresis	____ ______ ________
Prone position	◯yes ◯no
Start date of prone position	____ ______ ________
Is the prone position at the start of study ongoing?	◯yes ◯no

Stop date of prone position	____ ______ ________
Previous ECMO therapy	◯yes ◯no
Start date of ECMO	____ ______ ________
Stop date of ECMO	____ ______ ________
Prior type of ventilation	◯no<br>◯Nasal high-flow oxygen therapy<br>◯Noninvasive mechanical ventilation<br>◯Invasive ventilation<br>◯Tracheotomy<br>◯Ambient air<br>◯Standard oxygen sources
Start date of ventilation	____ ______ ________
Is this type of ventilation at the start of study ongoing?	◯yes ◯no
Stop date of ventilation	____ ______ ________
Lies the patient in the intensive care unit?	◯yes ◯no
Any prior medication regarding COVID-19	◯yes ◯no ◯not known
Link to previous therapy	see table below

	Therapy number	Therapy	Total Daily Dose	Unit	Onset date of medication	Still ongoing	Stop date of therapy

Set Link / Create Entry ...

Family history

Cancer	◯yes ◯no ◯not known
Cardiovascular disease	◯yes ◯no ◯not known
Diabetes	◯yes ◯no ◯not known
Rheumatological disease	◯yes ◯no ◯not known

Additional medical history? ◯yes ◯no

If 'yes' then add details of medical history

Add Medical History...

	MH number	System category	Description/Condition (incl. all currently present concomitant illnesses)	Date of onset / diagnosis / surgery	Still ongoing	Date resolved

Medical History

Medical history number	
System category	▼
Description/Condition (incl. all currently present concomitant illnesses)	
Date of onset / diagnosis / surgery	
Still ongoing	◯yes ◯no
Date resolved	

Concomitant medication

Centre ID	
Patient ID	
Visit	
Any concomitant medication	○yes ○no
If yes, please document all concomitant medications in the following section:	
Link to Concomitant medication	

	Seq. no.	Drug Name	Indicatio n	Start date	Ongoing at end of study	Stop date

Set Link / Create Entry ...

Smoking history

Centre ID	
Patient ID	
Visit	
Does the patient smoke?	◯Yes ◯Non-smoker ◯Ex-smoker
Smoking duration	____________________ years
Smoking amount	____________________ packs per day

Signs/Symptoms

Centre ID	
Patient ID	
Visit	
Cough	○yes ○no
Fever	○yes ○no
Myalagia	○yes ○no
Fatigue	○yes ○no
Diarrhoea	○yes ○no
Vomitting	○yes ○no
Headache	○yes ○no
Abdominal pain	○yes ○no
Nausea	○yes ○no
Olfactory or taste disorder	○yes ○no
Dyspnea	○yes ○no
Confusion	○yes ○no

Vital signs

Centre ID	
Patient ID	
Visit	
Date of vital signs	____ ______ ________
WHO performance status	◯ECOG 0 = Fully active, able to carry on all pre-disease performance without restriction<br>◯ECOG 1 = Restricted in physically strenuous activity but ambulatory and able to carry out work of a light or sedentary nature, e.g., light house work, office work<br>◯ECOG 2 = Ambulatory and capable of all selfcare but unable to carry out any work activities; up and about more than 50% of waking hours<br>◯ECOG 3 = Capable of only limited self-care, confined to bed or chair more than 50% of waking hours<br>◯ECOG 4 = Completely disabled. Cannot carry on any self-care. Totally confined to bed or chair.
Body temperature	____________________ °C
Type of measurement	◯oral ◯tympanic
Systolic blood pressure	____________________ mmHg
Diastolic blood pressure	____________________ mmHg
Pulse rate	____________________ beats/min
Respiratory rate	____________________ breaths/min
Height	____________________ cm
Weight	____________________ kg

Physical examination

Centre ID	
Patient ID	
Visit	
Date of physical examination	____ ______ ________

Physical examination

Physical Examination	not done	Status	Please specify	Clinically significant
Inspection (General appearance)	◯not done	◯normal ◯abnormal	________	◯yes ◯no
Lung	◯not done	◯normal ◯abnormal	________	◯yes ◯no
Cardiac auscultation	◯not done	◯normal ◯abnormal	________	◯yes ◯no
Cardiac percussion	◯not done	◯normal ◯abnormal	________	◯yes ◯no
Abdominal auscultation	◯not done	◯normal ◯abnormal	________	◯yes ◯no
Abdominal percussion	◯not done	◯normal ◯abnormal	________	◯yes ◯no
Abdominal palpation	◯not done	◯normal ◯abnormal	________	◯yes ◯no
Palpation of lymph nodes sites	◯not done	◯normal ◯abnormal	________	◯yes ◯no
Neurological examination	◯not done	◯normal ◯abnormal	________	◯yes ◯no
Peripheral edema	◯not done	◯normal ◯abnormal	________	◯yes ◯no

Other physical examination? ◯yes ◯no

If 'Other physical examination?' answer with 'yes' then add

Add Other Physical examination...

	Number	Physical examination	Status	Please specify	Clinically significant

Other Physical examination

Number	
Body system	____________________
Status	◯normal ◯abnormal
if abnormal: Please specify	____________________
if abnormal: Clinically significant	◯yes ◯no

Seven point ordinal scale

Centre ID	
Patient ID	
Visit	
Time of record	____ ____ ____
Ordinal Scale for Clinical Improvement	◯1 = not hospitalized with resumption of normal activities<br>◯2 = not hospitalized, but unable to resume normal sctivities<br>◯3 = hospitalized,not requiring supplemental oxygen<br>◯4 = hospitalized, requiring supplemental oxygen<br>◯5 = hospitalized, requiring nasal high-flow oxygen therapy or, noninvasive mechanical ventilation<br>◯6 = hospitalized, requiring ECMO, invasive mechanical ventilation, or both<br>◯7 = death

SOFA-Score

Centre ID	
Patient ID	
Visit	
Respiratory system (PaO2/FiO2 (mmHg))	> 400<br>< 400<br>< 300<br>< 200 with respiratoy support<br>< 100 with respiratoy support
Nervous system (Glasgow Coma Scale)	15<br>13-14<br>10-12<br>6-9<br><6
Cardiovascular system (Mean arterial pressure (MAP) or administration of vasopressor required)	MAP > 70 mmHg<br>MAP < 70 mmHg<br>Dopamine 5 µg/kg/min or dobutamine any dose<br>Dopamine > 5 µg/kg/min or epinephrine 0.1 µg/kg/min or norepinephrine 0.1 µg/kg/min<br>Dopamine > 15 µg/kg/min OR epinephrine > 0.1 µg/kg/min OR norepinephrine > 0.1 µg/kg/min
Liver (Bilirubin (mg/dl) [µmol/l])	< 1.2 [< 20]<br>1.2 - 1.9 [20 - 32]<br>2.0 - 5.9 [33 - 101]<br>6.0 - 11.9 [102 - 204]<br>> 12.0 [> 204]
Coagulation (Platelets x103/ml)	> 150<br>100-150<br>50-99<br>20-49<br>< 20
Kidneys (Creatinine (mg/dl) [µmol/L]; urine output)	< 1.2 [< 110]<br>1.2 - 1.9 [110 - 170]<br>2.0 - 3.4 [171 - 299]<br>3. - 4.9 [300 - 440]<br>> 5.0 [> 440]

Oxygen saturation

Centre ID	
Patient ID	
Visit	
ECMO	◯yes ◯no
Type of ventilation	◯no<br>◯Nasal high-flow oxygen therapy<br>◯Noninvasive mechanical ventilation<br>◯Invasive ventilation<br>◯Tracheotomy<br>◯Ambient air<br>◯Standard oxygen sources
Amount of oxygen	____________________ liter/min
Oxygen saturation (SaO2)	____________________ %
Amount of supplemental oxygen that is required to keep SaO2 above 94%	____________________ liter/min
Fraction of Inspired Oxygen (FiO2)	____________________ %
Partial pressure of oxygen (PaO2)	____________________ mmHg
Partial pressure of carbon dioxid (PaCO2)	____________________ mmHg

Echocardiogram

Centre ID	
Patient ID	
Visit	
Date of examination	____ ______ ________
Left ventricular ejection fraction (LVEF)	◯Normal ◯Abnormal
LVEF result	____________________

ECG

Centre ID	
Patient ID	
Visit	
Date of examination	____ ______ ________
Result of ECG	◯normal ◯abnormal
Clinically significant	◯yes ◯no
Description	_______________
Heart rate	____________________ beats/min
Rhythm	◯Normal sinus ◯Other
Please specify	_______________

Hematology

Centre ID	
Patient ID	
Visit	
Date of sample taken	____ ______ ________

Hematology

<small>Please use the same unit for parameters <i>Leukocytes</i> to <i>Thrombocytes</i>.</small>
<small>Please use a point '.' as decimal separator.</small>

Parameter	not done	Result	Unit
Hemoglobin	◯yes		▾
RBC	◯yes		▾
WBC	◯yes		▾
Lymphocytes	◯yes		▾
Neutrophils	◯yes		▾
Thrombocytes	◯yes		▾

Blood chemistry and coagulation

Centre ID	
Patient ID	
Visit	
Date of sample taken	____ _____ _______

Clinical chemistry
<small>Please use a point '.' as decimal separator.</small>

Parameter	not done	Result	Unit
BUN	yes		
Creatinine	yes		
Albumin	yes		
AST/SGOT	yes		
ALT/SGPT	yes		
Total bilirubin	yes		
GGT	yes		
AP	yes		
LDH	yes		
Sodium	yes		
Potassium	yes		
Magnesium	yes		
Calcium	yes		
Uric acid	yes		
Troponin	yes		
CK	yes		U/l
CK-MB	yes		U/l
PTT	yes		sec
ATIII	yes		%
D-Dimer	yes		
Fibrinogen	yes		
Ferritin	yes		
Transferrin	yes		
Transferrin Saturation	yes		%
CRP	yes		
Total protein	yes		g/l
IL6	yes		pg/ml
Procalcitonin	yes		ng/ml
Total IgG	yes		
IgA	yes		
IgM	yes		
Lactate	yes		
INR	yes		

Urinalysis

Centre ID	
Patient ID	
Visit	
Date of urine sample	____ ______ ________

Urinanalysis

<small>Please use a point '.' as decimal separator.</small>

Parameter	not done	Result	Value	Unit
pH-value	◯yes			
Glucose	◯yes			mg/dl
Protein	◯yes			mg/dl

Other Virology

Centre ID	
Patient ID	
Visit	
Date of blood sample	____ ______ ________
Anti-HAV	◯Negative ◯Positive
Anti-HBV	◯Negative ◯Positive
Anti-HCV IgG	◯Negative ◯Positive
HIV	◯Negative ◯Positive
if positive: Virus load	____________________ U/ml

Pregnancy test

Centre ID	
Patient ID	
Visit	
Date sample collected	____ ______ ________
Test medium	◯Blood ◯Urine
hCG	◯Negative ◯Positive

SARS-CoV-2 viral clearance and load as well as antibody titres

Centre ID	
Patient ID	
Visit	
Sample collection on Date of visit?	◯yes ◯no
Date of sample collection	____ ______ ________
Type of smear	◯Nasopharyngeal<br>◯Oropharyngeal<br>◯Sputum<br>◯Other
If other: Please specify	____________________
Result of smear	◯Negative ◯Positive ◯Invalide
PCR test type	◯TibMolbiol<br>◯Seegene<br>◯Abbott<br>◯Altona<br>◯Other
If other: Please specify	____________________
CT-value	____________________
Serodiagnostic by ELISA (OD Ratio)	____________________
Neutralize antibody titre (1:....)	____________________ ▾
Immunofluorescence	◯Positive<br>◯Borderline positive<br>◯Negative<br>◯Not performed

Enrollment/Randomization

Centre ID	
Patient ID	
Visit	
Is the patient randomized in the study?	◯yes ◯no
Date of randomization	____ ______ ________
Randomization number	____________________
Randomization result	◯Experimental arm ◯Standard arm

Day 1

Centre ID	
Patient ID	
Visit	Day 1
Date of Visit	____ ______ ________

- Vital signs
- Seven point ordinal scale
- Sequential Organ Failure Assessment (SOFA) Score
- Oxygen saturation (SaO2)
- Hematology
- Blood chemistry and coagulation
- SARS-CoV-2 viral clearance and load as well as antibody titres
- Procurement of Samples for Biobanking
- Administration of convalescent plasma
- Concomitant medication
- Concomitant COVID-19 therapy
- Adverse events

Investigator's Signature

Meaning:	I confirm the completeness and correctness of all documented data.
Signed By:	
Signature Date:	

Vital signs

Centre ID	
Patient ID	
Visit	
WHO performance status	◯ECOG 0 = Fully active, able to carry on all pre-disease performance without restriction<br>◯ECOG 1 = Restricted in physically strenuous activity but ambulatory and able to carry out work of a light or sedentary nature, e.g., light house work, office work<br>◯ECOG 2 = Ambulatory and capable of all selfcare but unable to carry out any work activities; up and about more than 50% of waking hours<br>◯ECOG 3 = Capable of only limited self-care, confined to bed or chair more than 50% of waking hours<br>◯ECOG 4 = Completely disabled. Cannot carry on any self-care. Totally confined to bed or chair.
Body temperature	____________________ °C
Type of measurement	◯oral ◯tympanic
Systolic blood pressure	____________________ mmHg
Diastolic blood pressure	____________________ mmHg
Pulse rate	____________________ beats/min
respiratory rate	____________________ breaths/min

Seven point ordinal scale

Centre ID	
Patient ID	
Visit	
Time of record	____ ____ ____
Ordinal Scale for Clinical Improvement	◯1 = not hospitalized with resumption of normal activities<br>◯2 = not hospitalized, but unable to resume normal sctivities<br>◯3 = hospitalized,not requiring supplemental oxygen<br>◯4 = hospitalized, requiring supplemental oxygen<br>◯5 = hospitalized, requiring nasal high-flow oxygen therapy or, noninvasive mechanical ventilation<br>◯6 = hospitalized, requiring ECMO, invasive mechanical ventilation, or both<br>◯7 = death

Sequential Organ Failure Assessment (SOFA) Score

Centre ID	
Patient ID	
Visit	
Respiratory system (PaO2/FiO2 (mmHg))	◯> 400<br>◯< 400<br>◯< 300<br>◯< 200 with respiratoy support<br>◯< 100 with respiratoy support
Nervous system (Glasgow Coma Scale)	◯15<br>◯13-14<br>◯10-12<br>◯6-9<br>◯<6
Cardiovascular system (Mean arterial pressure (MAP) or administration of vasopressor required)	◯MAP > 70 mmHg<br>◯MAP < 70 mmHg<br>◯Dopamine 5 µg/kg/min or dobutamine any dose<br>◯Dopamine > 5 µg/kg/min or epinephrine 0.1 µg/kg/min or norepinephrine 0.1 µg/kg/min<br>◯Dopamine > 15 µg/kg/min OR epinephrine > 0.1 µg/kg/min OR norepinephrine > 0.1 µg/kg/min
Liver (Bilirubin (mg/dl) [µmol/l])	◯< 1.2 [< 20]<br>◯1.2 - 1.9 [20 - 32]<br>◯2.0 - 5.9 [33 - 101]<br>◯6.0 - 11.9 [102 - 204]<br>◯> 12.0 [> 204]
Coagulation (Platelets x103/ml)	◯> 150<br>◯100-150<br>◯50-99<br>◯20-49<br>◯< 20
Kidneys (Creatinine (mg/dl) [µmol/L]; urine output)	◯< 1.2 [< 110]<br>◯1.2 - 1.9 [110 - 170]<br>◯2.0 - 3.4 [171 - 299]<br>◯3. - 4.9 [300 - 440]<br>◯> 5.0 [> 440]

Oxygen saturation (SaO2)

Centre ID	
Patient ID	
Visit	
ECMO	◯yes ◯no
Type of ventilation	◯no<br>◯Nasal high-flow oxygen therapy<br>◯Noninvasive mechanical ventilation<br>◯Invasive ventilation<br>◯Tracheotomy<br>◯Ambient air<br>◯Standard oxygen sources
Amount of oxygen	____________________ liter/min
Oxygen saturation (SaO2)	____________________ %
Amount of supplemental oxygen that is required to keep SaO2 above 94%	____________________ liter/min
Fraction of Inspired Oxygen (FiO2)	____________________ %
Partial pressure of oxygen (PaO2)	____________________ mmHg
Partial pressure of carbon dioxid (PaCO2)	____________________ mmHg

Hematology

Centre ID	
Patient ID	
Visit	
Date of sample taken	____ _____ _______

Hematology

Parameter	not done	Result	Unit
Hemoglobin	yes		
RBC	yes		
WBC	yes		
Thrombocytes	yes		

Blood chemistry and coagulation

Centre ID	
Patient ID	
Visit	
Date of sample taken	____ ______ ________

Clinical chemistry
<small>Please use a point '.' as decimal separator.</small>

Parameter	not done	Result	Unit
BUN	yes		
Creatinine	yes		
Albumin	yes		
AST/SGOT	yes		
ALT/SGPT	yes		
Total bilirubin	yes		
GGT	yes		
AP	yes		
LDH	yes		
Sodium	yes		
Potassium	yes		
Magnesium	yes		
Calcium	yes		
Uric acid	yes		
Troponin	yes		
CK	yes		U/l
CK-MB	yes		U/l
PTT	yes		sec
ATIII	yes		%
D-Dimer	yes		
Fibrinogen	yes		
Ferritin	yes		
Transferrin	yes		
Transferrin Saturation	yes		%
CRP	yes		
Total protein	yes		g/l
IL6	yes		pg/ml
Procalcitonin	yes		ng/ml
Total IgG	yes		
IgA	yes		
IgM	yes		
Lactate	yes		
INR	yes		

SARS-CoV-2 viral clearance and load as well as antibody titres

Centre ID	
Patient ID	
Visit	
Sample collection on Date of visit?	◯yes ◯no
Date of sample collection	____ ______ ________
Time of sample collection	____ ____ ____
Type of smear	◯Nasopharyngeal<br>◯Oropharyngeal<br>◯Sputum<br>◯Other
If other: Please specify	____________________
Result of smear	◯Negative ◯Positive ◯Invalide
PCR test type	◯TibMolbiol<br>◯Seegene<br>◯Abbott<br>◯Altona<br>◯Other
If other: Please specify	____________________
CT-value	____________________
Serodiagnostic by ELISA (OD Ratio)	____________________
Neutralize antibody titre (1:....)	____________________ ▾
Immunofluorescence	◯Positive<br>◯Borderline positive<br>◯Negative<br>◯Not performed

Procurement of Samples for Biobanking

Centre ID	
Patient ID	
Visit	
1 x 7,5 ml serum taken?	◯yes ◯no
Sample collection on Date of visit?	◯yes ◯no
Date of collection	____ ______ ________
Time of collection	____ ____ ____
Material	◯complete ◯less material
if no: Reason	____________________
2 x 7,5 ml Lithium-heparin taken?	◯yes ◯no
Sample collection on Date of visit?	◯yes ◯no
Date of collection	____ ______ ________
Time of collection	____ ____ ____
Material	◯complete ◯less material
if no: Reason	____________________

Administration of convalescent plasma

Centre ID	
Patient ID	
Visit	
Batch number of convalascent plasma	________
Time of infusion start	____ ____ ____
Time of infusion stop	____ ____ ____
Amount of infusion	____________________ ml
Transfusion reactions	◯No<br>◯Transfusion associated circulatory overload (TACO)<br>◯Citrate intoxication<br>◯Allergic reaction<br>◯Transfusion transmitted infection<br>◯Transfusion associated lung injury (TRALI)<br>◯Other
if other: Please specify	_______________
Treatment discontinuation	◯yes ◯no
Reason for treatment discontinution	◯Adverse event (other than allergic reaction) with the transfusion of CP<br>◯Serious Adverse Event upon CP transfusion<br>◯Allergic reaction

Continuation of CP treatment after AE resolution to smaller than grade 2 according to CTCAE

■ Date of continuation	____ ______ ________
■ Time of infusion start	____ ____ ____
■ Time of infusion stop	____ ____ ____
■ Amount of infusion	____________________ ml
■ Transfusion reactions	◯No<br>◯Transfusion associated circulatory overload (TACO)<br>◯Citrate intoxication<br>◯Allergic reaction<br>◯Transfusion transmitted infection<br>◯Transfusion associated lung injury (TRALI)<br>◯Other
■ if other: Please specify	_______________

Concomitant medication

Centre ID	
Patient ID	
Visit	
Any new concomitant medication?	◯yes ◯no
If yes, please document all changes in Concomitant medications in the following section:	
Link to Concomitant medication	

	Seq. no.	Drug Name	Indicatio n	Start date	Ongoing at end of study	Stop date

Set Link / Create Entry ...

Concomitant COVID-19 therapy

Centre ID	
Patient ID	
Visit	
Any new Concomitant COVID therapy?	◯yes ◯no

If yes, please document all changes in Concomitant COVID-19 therapy in the following section:

Link to Concomitant COVID-19 therapy

	Therapy number	Therapy	Total Daily Dose	Unit	Onset date of medication	Still ongoing	Stop date of therapy

Set Link / Create Entry ...

Adverse events

Centre ID	
Patient ID	
Visit	
Any new Adverse events?	◯yes ◯no

If yes, please document all new Adverse Events in the following section:

Link to Adverse Events

	AE number	SOC of AE (according CTCAE V5.0)	AE term (according CTCAE V5.0)	Start date	Ongoing at the end of study	Stop date	Is the AE serious?

Set Link / Create Entry ...

Day 2

Centre ID	
Patient ID	
Visit	Day 2
Date of Visit	____ ______ ________

- Vital signs
- Seven point ordinal scale
- Sequential Organ Failure Assessment (SOFA) Score
- Oxygen saturation (SaO2)
- Hematology
- Blood chemistry and coagulation
- SARS-CoV-2 viral clearance and load as well as antibody titres
- Administration of convalescent plasma
- Concomitant medication
- Concomitant COVID-19 therapy
- Adverse events

Investigator's Signature

Meaning:	I confirm the completeness and correctness of all documented data.
Signed By:	
Signature Date:	

Vital signs

Centre ID	
Patient ID	
Visit	
WHO performance status	◯ECOG 0 = Fully active, able to carry on all pre-disease performance without restriction<br>◯ECOG 1 = Restricted in physically strenuous activity but ambulatory and able to carry out work of a light or sedentary nature, e.g., light house work, office work<br>◯ECOG 2 = Ambulatory and capable of all selfcare but unable to carry out any work activities; up and about more than 50% of waking hours<br>◯ECOG 3 = Capable of only limited self-care, confined to bed or chair more than 50% of waking hours<br>◯ECOG 4 = Completely disabled. Cannot carry on any self-care. Totally confined to bed or chair.
Body temperature	____________________ °C
Type of measurement	◯oral ◯tympanic
Systolic blood pressure	____________________ mmHg
Diastolic blood pressure	____________________ mmHg
Pulse rate	____________________ beats/min
respiratory rate	____________________ breaths/min

Seven point ordinal scale

Centre ID	
Patient ID	
Visit	
Time of record	____ ____ ____
Ordinal Scale for Clinical Improvement	◯1 = not hospitalized with resumption of normal activities<br>◯2 = not hospitalized, but unable to resume normal sctivities<br>◯3 = hospitalized,not requiring supplemental oxygen<br>◯4 = hospitalized, requiring supplemental oxygen<br>◯5 = hospitalized, requiring nasal high-flow oxygen therapy or, noninvasive mechanical ventilation<br>◯6 = hospitalized, requiring ECMO, invasive mechanical ventilation, or both<br>◯7 = death

Sequential Organ Failure Assessment (SOFA) Score

Centre ID	
Patient ID	
Visit	
Respiratory system (PaO2/FiO2 (mmHg))	◯> 400<br>◯< 400<br>◯< 300<br>◯< 200 with respiratoy support<br>◯< 100 with respiratoy support
Nervous system (Glasgow Coma Scale)	◯15<br>◯13-14<br>◯10-12<br>◯6-9<br>◯<6
Cardiovascular system (Mean arterial pressure (MAP) or administration of vasopressor required)	◯MAP > 70 mmHg<br>◯MAP < 70 mmHg<br>◯Dopamine 5 µg/kg/min or dobutamine any dose<br>◯Dopamine > 5 µg/kg/min or epinephrine 0.1 µg/kg/min or norepinephrine 0.1 µg/kg/min<br>◯Dopamine > 15 µg/kg/min OR epinephrine > 0.1 µg/kg/min OR norepinephrine > 0.1 µg/kg/min
Liver (Bilirubin (mg/dl) [µmol/l])	◯< 1.2 [< 20]<br>◯1.2 - 1.9 [20 - 32]<br>◯2.0 - 5.9 [33 - 101]<br>◯6.0 - 11.9 [102 - 204]<br>◯> 12.0 [> 204]
Coagulation (Platelets x103/ml)	◯> 150<br>◯100-150<br>◯50-99<br>◯20-49<br>◯< 20
Kidneys (Creatinine (mg/dl) [µmol/L]; urine output)	◯< 1.2 [< 110]<br>◯1.2 - 1.9 [110 - 170]<br>◯2.0 - 3.4 [171 - 299]<br>◯3. - 4.9 [300 - 440]<br>◯> 5.0 [> 440]

Oxygen saturation (SaO2)

Centre ID	
Patient ID	
Visit	
ECMO	◯yes ◯no
Type of ventilation	◯no<br>◯Nasal high-flow oxygen therapy<br>◯Noninvasive mechanical ventilation<br>◯Invasive ventilation<br>◯Tracheotomy<br>◯Ambient air<br>◯Standard oxygen sources
Amount of oxygen	____________________ liter/min
Oxygen saturation (SaO2)	____________________ %
Amount of supplemental oxygen that is required to keep SaO2 above 94%	____________________ liter/min
Fraction of Inspired Oxygen (FiO2)	____________________ %
Partial pressure of oxygen (PaO2)	____________________ mmHg
Partial pressure of carbon dioxid (PaCO2)	____________________ mmHg

Hematology

Centre ID	
Patient ID	
Visit	
Date of sample taken	____ _____ ______

Hematology

Parameter	not done	Result	Unit
Hemoglobin	yes		
RBC	yes		
WBC	yes		
Thrombocytes	yes		

Blood chemistry and coagulation

Centre ID	
Patient ID	
Visit	
Date of sample taken	____ _____ _______

Clinical chemistry
<small>Please use a point '.' as decimal separator.</small>

Parameter	not done	Result	Unit
BUN	yes		
Creatinine	yes		
Albumin	yes		
AST/SGOT	yes		
ALT/SGPT	yes		
Total bilirubin	yes		
GGT	yes		
AP	yes		
LDH	yes		
Sodium	yes		
Potassium	yes		
Magnesium	yes		
Calcium	yes		
Uric acid	yes		
Troponin	yes		
CK	yes		U/l
CK-MB	yes		U/l
PTT	yes		sec
ATIII	yes		%
D-Dimer	yes		
Fibrinogen	yes		
Ferritin	yes		
Transferrin	yes		
Transferrin Saturation	yes		%
CRP	yes		
Total protein	yes		g/l
IL6	yes		pg/ml
Procalcitonin	yes		ng/ml
Total IgG	yes		
IgA	yes		
IgM	yes		
Lactate	yes		
INR	yes		

SARS-CoV-2 viral clearance and load as well as antibody titres

Centre ID	
Patient ID	
Visit	
Sample collection on Date of visit?	◯yes ◯no
Date of sample collection	____ ______ ________
Type of smear	◯Nasopharyngeal<br>◯Oropharyngeal<br>◯Sputum<br>◯Other
If other: Please specify	____________________
Result of smear	◯Negative ◯Positive ◯Invalide
PCR test type	◯TibMolbiol<br>◯Seegene<br>◯Abbott<br>◯Altona<br>◯Other
If other: Please specify	____________________
CT-value	____________________
Serodiagnostic by ELISA (OD Ratio)	____________________
Neutralize antibody titre (1:....)	____________________ ▾
Immunofluorescence	◯Positive<br>◯Borderline positive<br>◯Negative<br>◯Not performed

Administration of convalescent plasma

Centre ID	
Patient ID	
Visit	
Batch number of convalascent plasma	________
Time of infusion start	____ ____ ____
Time of infusion stop	____ ____ ____
Amount of infusion	____________________ ml
Transfusion reactions	◯No<br>◯Transfusion associated circulatory overload (TACO)<br>◯Citrate intoxication<br>◯Allergic reaction<br>◯Transfusion transmitted infection<br>◯Transfusion associated lung injury (TRALI)<br>◯Other
if other: Please specify	_______________
Treatment discontinuation	◯yes ◯no
Reason for treatment discontinution	◯Adverse event (other than allergic reaction) with the transfusion of CP<br>◯Serious Adverse Event upon CP transfusion<br>◯Allergic reaction
Continuation of CP treatment after AE resolution to smaller than grade 2 according to CTCAE	
■ Date of continuation	____ ______ ________
■ Time of infusion start	____ ____ ____
■ Time of infusion stop	____ ____ ____
■ Amount of infusion	____________________ ml
■ Transfusion reactions	◯No<br>◯Transfusion associated circulatory overload (TACO)<br>◯Citrate intoxication<br>◯Allergic reaction<br>◯Transfusion transmitted infection<br>◯Transfusion associated lung injury (TRALI)<br>◯Other
■ if other: Please specify	_______________

Concomitant medication

Centre ID	
Patient ID	
Visit	
Any new or changed concomitant medication since last visit	◯yes ◯no
If yes, please document all changes in Concomitant medications in the following section:	
Link to Concomitant medication	

	Seq. no.	Drug Name	Indicatio n	Start date	Ongoing at end of study	Stop date

Set Link / Create Entry ...

Concomitant COVID-19 therapy

Centre ID	
Patient ID	
Visit	
Any new Concomitant COVID-19 therapy since last visit?	○yes ○no
If yes, please document all changes in Concomitant COVID-19 therapy in the following section:	
Link to Concomitant COVID-19 therapy	see table below

	Therapy number	Therapy	Total Daily Dose	Unit	Onset date of medication	Still ongoing	Stop date of therapy

Set Link / Create Entry ...

Adverse events

Centre ID	
Patient ID	
Visit	
Any new Adverse events since last visit?	◯yes ◯no

If yes, please document all new Adverse Events in the following section:

Link to Adverse Events

	AE number	SOC of AE (according CTCAE V5.0)	AE term (according CTCAE V5.0)	Start date	Ongoing at the end of study	Stop date	Is the AE serious?

Set Link / Create Entry ...

Day 3

Centre ID	
Patient ID	
Visit	Day 3
Date of Visit	____ ______ ________
Is the patient hospitalized?	◯yes ◯no

- Vital signs
- Seven point ordinal scale
- Sequential Organ Failure Assessment (SOFA) Score
- Oxygen saturation (SaO2)
- Hematology
- Blood chemistry and coagulation
- SARS-CoV-2 viral clearance and load as well as antibody titres
- Concomitant medication
- Concomitant COVID-19 therapy
- Adverse events

Investigator's Signature

Meaning:	I confirm the completeness and correctness of all documented data.
Signed By:	
Signature Date:	

Vital signs

Centre ID	
Patient ID	
Visit	
WHO performance status	◯ECOG 0 = Fully active, able to carry on all pre-disease performance without restriction<br>◯ECOG 1 = Restricted in physically strenuous activity but ambulatory and able to carry out work of a light or sedentary nature, e.g., light house work, office work<br>◯ECOG 2 = Ambulatory and capable of all selfcare but unable to carry out any work activities; up and about more than 50% of waking hours<br>◯ECOG 3 = Capable of only limited self-care, confined to bed or chair more than 50% of waking hours<br>◯ECOG 4 = Completely disabled. Cannot carry on any self-care. Totally confined to bed or chair.
Body temperature	____________________ °C
Type of measurement	◯oral ◯tympanic
Systolic blood pressure	____________________ mmHg
Diastolic blood pressure	____________________ mmHg
Pulse rate	____________________ beats/min
respiratory rate	____________________ breaths/min

Seven point ordinal scale

Centre ID	
Patient ID	
Visit	
Time of record	____ ____ ____
Ordinal Scale for Clinical Improvement	◯1 = not hospitalized with resumption of normal activities<br>◯2 = not hospitalized, but unable to resume normal sctivities<br>◯3 = hospitalized,not requiring supplemental oxygen<br>◯4 = hospitalized, requiring supplemental oxygen<br>◯5 = hospitalized, requiring nasal high-flow oxygen therapy or, noninvasive mechanical ventilation<br>◯6 = hospitalized, requiring ECMO, invasive mechanical ventilation, or both<br>◯7 = death

Sequential Organ Failure Assessment (SOFA) Score

Centre ID	
Patient ID	
Visit	
Respiratory system (PaO2/FiO2 (mmHg))	◯> 400<br>◯< 400<br>◯< 300<br>◯< 200 with respiratoy support<br>◯< 100 with respiratoy support
Nervous system (Glasgow Coma Scale)	◯15<br>◯13-14<br>◯10-12<br>◯6-9<br>◯<6
Cardiovascular system (Mean arterial pressure (MAP) or administration of vasopressor required)	◯MAP > 70 mmHg<br>◯MAP < 70 mmHg<br>◯Dopamine 5 µg/kg/min or dobutamine any dose<br>◯Dopamine > 5 µg/kg/min or epinephrine 0.1 µg/kg/min or norepinephrine 0.1 µg/kg/min<br>◯Dopamine > 15 µg/kg/min OR epinephrine > 0.1 µg/kg/min OR norepinephrine > 0.1 µg/kg/min
Liver (Bilirubin (mg/dl) [µmol/l])	◯< 1.2 [< 20]<br>◯1.2 - 1.9 [20 - 32]<br>◯2.0 - 5.9 [33 - 101]<br>◯6.0 - 11.9 [102 - 204]<br>◯> 12.0 [> 204]
Coagulation (Platelets x103/ml)	◯> 150<br>◯100-150<br>◯50-99<br>◯20-49<br>◯< 20
Kidneys (Creatinine (mg/dl) [µmol/L]; urine output)	◯< 1.2 [< 110]<br>◯1.2 - 1.9 [110 - 170]<br>◯2.0 - 3.4 [171 - 299]<br>◯3. - 4.9 [300 - 440]<br>◯> 5.0 [> 440]

Oxygen saturation (SaO2)

Centre ID	
Patient ID	
Visit	
ECMO	◯yes ◯no
Type of ventilation	◯no<br>◯Nasal high-flow oxygen therapy<br>◯Noninvasive mechanical ventilation<br>◯Invasive ventilation<br>◯Tracheotomy<br>◯Ambient air<br>◯Standard oxygen sources
Amount of oxygen	____________________ liter/min
Oxygen saturation (SaO2)	____________________ %
Amount of supplemental oxygen that is required to keep SaO2 above 94%	____________________ liter/min
Fraction of Inspired Oxygen (FiO2)	____________________ %
Partial pressure of oxygen (PaO2)	____________________ mmHg
Partial pressure of carbon dioxid (PaCO2)	____________________ mmHg

Hematology

Centre ID	
Patient ID	
Visit	
Date of sample taken	____ _____ ______

Hematology

Parameter	not done	Result	Unit
Hemoglobin	◯yes		▾
RBC	◯yes		▾
WBC	◯yes		▾
Thrombocytes	◯yes		▾

Blood chemistry and coagulation

Centre ID	
Patient ID	
Visit	
Date of sample taken	____ ______ ________

Clinical chemistry
<small>Please use a point '.' as decimal separator.</small>

Parameter	not done	Result	Unit
BUN	◯yes		
Creatinine	◯yes		
Albumin	◯yes		
AST/SGOT	◯yes		
ALT/SGPT	◯yes		
Total bilirubin	◯yes		
GGT	◯yes		
AP	◯yes		
LDH	◯yes		
Sodium	◯yes		
Potassium	◯yes		
Magnesium	◯yes		
Calcium	◯yes		
Uric acid	◯yes		
Troponin	◯yes		
CK	◯yes		U/l
CK-MB	◯yes		U/l
PTT	◯yes		sec
ATIII	◯yes		%
D-Dimer	◯yes		
Fibrinogen	◯yes		
Ferritin	◯yes		
Transferrin	◯yes		
Transferrin Saturation	◯yes		%
CRP	◯yes		
Total protein	◯yes		g/l
IL6	◯yes		pg/ml
Procalcitonin	◯yes		ng/ml
Total IgG	◯yes		
IgA	◯yes		
IgM	◯yes		
Lactate	◯yes		
INR	◯yes		

SARS-CoV-2 viral clearance and load as well as antibody titres

Centre ID	
Patient ID	
Visit	
Sample collection on Date of visit?	◯yes ◯no
Date of sample collection	____ ______ ________
Type of smear	◯Nasopharyngeal<br>◯Oropharyngeal<br>◯Sputum<br>◯Other
If other: Please specify	____________________
Result of smear	◯Negative ◯Positive ◯Invalide
PCR test type	◯TibMolbiol<br>◯Seegene<br>◯Abbott<br>◯Altona<br>◯Other
If other: Please specify	____________________
CT-value	____________________
Serodiagnostic by ELISA (OD Ratio)	____________________
Neutralize antibody titre (1:....)	____________________ ▾
Immunofluorescence	◯Positive<br>◯Borderline positive<br>◯Negative<br>◯Not performed

Concomitant medication

Centre ID	
Patient ID	
Visit	
Any new or changed concomitant medication since last visit	○yes ○no
If yes, please document all changes in Concomitant medications in the following section:	
Link to Concomitant medication	

	Seq. no.	Drug Name	Indicatio n	Start date	Ongoing at end of study	Stop date

Set Link / Create Entry ...

Concomitant COVID-19 therapy

Centre ID	
Patient ID	
Visit	
Any new Concomitant COVID-19 therapy since last visit?	○yes ○no
If yes, please document all changes in Concomitant COVID-19 therapy in the following section:	
Link to Concomitant COVID-19 therapy	(see table below)

	Therapy number	Therapy	Total Daily Dose	Unit	Onset date of medication	Still ongoing	Stop date of therapy

Set Link / Create Entry ...

Adverse events

Centre ID	
Patient ID	
Visit	
Any new Adverse events since last visit?	◯yes ◯no

If yes, please document all new Adverse Events in the following section:

Link to Adverse Events

	AE number	SOC of AE (according CTCAE V5.0)	AE term (according CTCAE V5.0)	Start date	Ongoing at the end of study	Stop date	Is the AE serious?

Set Link / Create Entry ...

Day 4

Centre ID	
Patient ID	
Visit	Day 4
Date of Visit	____ ______ ________
Hospitalization?	◯yes ◯no

- Vital signs
- Seven point ordinal scale
- Sequential Organ Failure Assessment (SOFA) Score
- Oxygen saturation (SaO2)
- Hematology
- Blood chemistry and coagulation
- Concomitant medication
- Concomitant COVID-19 therapy
- Adverse events

Investigator's Signature

Meaning:	I confirm the completeness and correctness of all documented data.
Signed By:	
Signature Date:	

Vital signs

Centre ID	
Patient ID	
Visit	
WHO performance status	◯ECOG 0 = Fully active, able to carry on all pre-disease performance without restriction<br>◯ECOG 1 = Restricted in physically strenuous activity but ambulatory and able to carry out work of a light or sedentary nature, e.g., light house work, office work<br>◯ECOG 2 = Ambulatory and capable of all selfcare but unable to carry out any work activities; up and about more than 50% of waking hours<br>◯ECOG 3 = Capable of only limited self-care, confined to bed or chair more than 50% of waking hours<br>◯ECOG 4 = Completely disabled. Cannot carry on any self-care. Totally confined to bed or chair.
Body temperature	____________________ °C
Type of measurement	◯oral ◯tympanic
Systolic blood pressure	____________________ mmHg
Diastolic blood pressure	____________________ mmHg
Pulse rate	____________________ beats/min
respiratory rate	____________________ breaths/min

Seven point ordinal scale

Centre ID	
Patient ID	
Visit	
Time of record	____ ____ ____
Ordinal Scale for Clinical Improvement	◯1 = not hospitalized with resumption of normal activities<br>◯2 = not hospitalized, but unable to resume normal sctivities<br>◯3 = hospitalized,not requiring supplemental oxygen<br>◯4 = hospitalized, requiring supplemental oxygen<br>◯5 = hospitalized, requiring nasal high-flow oxygen therapy or, noninvasive mechanical ventilation<br>◯6 = hospitalized, requiring ECMO, invasive mechanical ventilation, or both<br>◯7 = death

Sequential Organ Failure Assessment (SOFA) Score

Centre ID	
Patient ID	
Visit	
Respiratory system (PaO2/FiO2 (mmHg))	◯> 400<br>◯< 400<br>◯< 300<br>◯< 200 with respiratoy support<br>◯< 100 with respiratoy support
Nervous system (Glasgow Coma Scale)	◯15<br>◯13-14<br>◯10-12<br>◯6-9<br>◯<6
Cardiovascular system (Mean arterial pressure (MAP) or administration of vasopressor required)	◯MAP > 70 mmHg<br>◯MAP < 70 mmHg<br>◯Dopamine 5 µg/kg/min or dobutamine any dose<br>◯Dopamine > 5 µg/kg/min or epinephrine 0.1 µg/kg/min or norepinephrine 0.1 µg/kg/min<br>◯Dopamine > 15 µg/kg/min OR epinephrine > 0.1 µg/kg/min OR norepinephrine > 0.1 µg/kg/min
Liver (Bilirubin (mg/dl) [µmol/l])	◯< 1.2 [< 20]<br>◯1.2 - 1.9 [20 - 32]<br>◯2.0 - 5.9 [33 - 101]<br>◯6.0 - 11.9 [102 - 204]<br>◯> 12.0 [> 204]
Coagulation (Platelets x103/ml)	◯> 150<br>◯100-150<br>◯50-99<br>◯20-49<br>◯< 20
Kidneys (Creatinine (mg/dl) [µmol/L]; urine output)	◯< 1.2 [< 110]<br>◯1.2 - 1.9 [110 - 170]<br>◯2.0 - 3.4 [171 - 299]<br>◯3. - 4.9 [300 - 440]<br>◯> 5.0 [> 440]

Oxygen saturation (SaO2)

Centre ID	
Patient ID	
Visit	
ECMO	◯yes ◯no
Type of ventilation	◯no<br>◯Nasal high-flow oxygen therapy<br>◯Noninvasive mechanical ventilation<br>◯Invasive ventilation<br>◯Tracheotomy<br>◯Ambient air<br>◯Standard oxygen sources
Amount of oxygen	____________________ liter/min
Oxygen saturation (SaO2)	____________________ %
Amount of supplemental oxygen that is required to keep SaO2 above 94%	____________________ liter/min
Fraction of Inspired Oxygen (FiO2)	____________________ %
Partial pressure of oxygen (PaO2)	____________________ mmHg
Partial pressure of carbon dioxid (PaCO2)	____________________ mmHg

Hematology

Centre ID	
Patient ID	
Visit	
Date of sample taken	____ ______ ________

Hematology

Parameter	not done	Result	Unit
Hemoglobin	yes		
RBC	yes		
WBC	yes		
Thrombocytes	yes		

Blood chemistry and coagulation

Centre ID	
Patient ID	
Visit	
Date of sample taken	____ ______ ________

Clinical chemistry
<small>Please use a point '.' as decimal separator.</small>

Parameter	not done	Result	Unit
BUN	yes		
Creatinine	yes		
Albumin	yes		
AST/SGOT	yes		
ALT/SGPT	yes		
Total bilirubin	yes		
GGT	yes		
AP	yes		
LDH	yes		
Sodium	yes		
Potassium	yes		
Magnesium	yes		
Calcium	yes		
Uric acid	yes		
Troponin	yes		
CK	yes		U/l
CK-MB	yes		U/l
PTT	yes		sec
ATIII	yes		%
D-Dimer	yes		
Fibrinogen	yes		
Ferritin	yes		
Transferrin	yes		
Transferrin Saturation	yes		%
CRP	yes		
Total protein	yes		g/l
IL6	yes		pg/ml
Procalcitonin	yes		ng/ml
Total IgG	yes		
IgA	yes		
IgM	yes		
Lactate	yes		
INR	yes		

Concomitant medication

Centre ID	
Patient ID	
Visit	
Any new or changed concomitant medication since last visit	◯yes ◯no

If yes, please document all changes in Concomitant medications in the following section:

Link to Concomitant medication

	Seq. no.	Drug Name	Indicatio n	Start date	Ongoing at end of study	Stop date

Set Link / Create Entry ...

Concomitant COVID-19 therapy

Centre ID	
Patient ID	
Visit	
Any new Concomitant COVID-19 therapy since last visit?	◯yes ◯no
If yes, please document all changes in Concomitant COVID-19 therapy in the following section:	
Link to Concomitant COVID-19 therapy	see table below

	Therapy number	Therapy	Total Daily Dose	Unit	Onset date of medication	Still ongoing	Stop date of therapy

Set Link / Create Entry ...

Adverse events

Centre ID	
Patient ID	
Visit	
Any new Adverse events since last visit?	◯yes ◯no
If yes, please document all new Adverse Events in the following section:	
Link to Adverse Events	(see table below)

	AE number	SOC of AE (according CTCAE V5.0)	AE term (according CTCAE V5.0)	Start date	Ongoing at the end of study	Stop date	Is the AE serious?

Set Link / Create Entry ...

Day 5

Centre ID	
Patient ID	
Visit	Day 5
Date of Visit	____ ______ ________
Hospitalization?	◯yes ◯no

- Vital signs
- Seven point ordinal scale
- Sequential Organ Failure Assessment (SOFA) Score
- Oxygen saturation (SaO2)
- Hematology
- Blood chemistry and coagulation
- SARS-CoV-2 viral clearance and load as well as antibody titres
- Concomitant medication
- Concomitant COVID-19 therapy
- Adverse events

Investigator's Signature

Meaning:	I confirm the completeness and correctness of all documented data.
Signed By:	
Signature Date:	

Vital signs

Centre ID	
Patient ID	
Visit	
WHO performance status	◯ECOG 0 = Fully active, able to carry on all pre-disease performance without restriction<br>◯ECOG 1 = Restricted in physically strenuous activity but ambulatory and able to carry out work of a light or sedentary nature, e.g., light house work, office work<br>◯ECOG 2 = Ambulatory and capable of all selfcare but unable to carry out any work activities; up and about more than 50% of waking hours<br>◯ECOG 3 = Capable of only limited self-care, confined to bed or chair more than 50% of waking hours<br>◯ECOG 4 = Completely disabled. Cannot carry on any self-care. Totally confined to bed or chair.
Body temperature	____________________ °C
Type of measurement	◯oral ◯tympanic
Systolic blood pressure	____________________ mmHg
Diastolic blood pressure	____________________ mmHg
Pulse rate	____________________ beats/min
respiratory rate	____________________ breaths/min

Seven point ordinal scale

Centre ID	
Patient ID	
Visit	
Time of record	____ ____ ____
Ordinal Scale for Clinical Improvement	◯1 = not hospitalized with resumption of normal activities<br>◯2 = not hospitalized, but unable to resume normal sctivities<br>◯3 = hospitalized,not requiring supplemental oxygen<br>◯4 = hospitalized, requiring supplemental oxygen<br>◯5 = hospitalized, requiring nasal high-flow oxygen therapy or, noninvasive mechanical ventilation<br>◯6 = hospitalized, requiring ECMO, invasive mechanical ventilation, or both<br>◯7 = death

Sequential Organ Failure Assessment (SOFA) Score

Centre ID	
Patient ID	
Visit	
Respiratory system (PaO2/FiO2 (mmHg))	◯> 400<br>◯< 400<br>◯< 300<br>◯< 200 with respiratoy support<br>◯< 100 with respiratoy support
Nervous system (Glasgow Coma Scale)	◯15<br>◯13-14<br>◯10-12<br>◯6-9<br>◯<6
Cardiovascular system (Mean arterial pressure (MAP) or administration of vasopressor required)	◯MAP > 70 mmHg<br>◯MAP < 70 mmHg<br>◯Dopamine 5 µg/kg/min or dobutamine any dose<br>◯Dopamine > 5 µg/kg/min or epinephrine 0.1 µg/kg/min or norepinephrine 0.1 µg/kg/min<br>◯Dopamine > 15 µg/kg/min OR epinephrine > 0.1 µg/kg/min OR norepinephrine > 0.1 µg/kg/min
Liver (Bilirubin (mg/dl) [µmol/l])	◯< 1.2 [< 20]<br>◯1.2 - 1.9 [20 - 32]<br>◯2.0 - 5.9 [33 - 101]<br>◯6.0 - 11.9 [102 - 204]<br>◯> 12.0 [> 204]
Coagulation (Platelets x103/ml)	◯> 150<br>◯100-150<br>◯50-99<br>◯20-49<br>◯< 20
Kidneys (Creatinine (mg/dl) [µmol/L]; urine output)	◯< 1.2 [< 110]<br>◯1.2 - 1.9 [110 - 170]<br>◯2.0 - 3.4 [171 - 299]<br>◯3. - 4.9 [300 - 440]<br>◯> 5.0 [> 440]

Oxygen saturation (SaO2)

Centre ID	
Patient ID	
Visit	
ECMO	◯yes ◯no
Type of ventilation	◯no<br>◯Nasal high-flow oxygen therapy<br>◯Noninvasive mechanical ventilation<br>◯Invasive ventilation<br>◯Tracheotomy<br>◯Ambient air<br>◯Standard oxygen sources
Amount of oxygen	____________________ liter/min
Oxygen saturation (SaO2)	____________________ %
Amount of supplemental oxygen that is required to keep SaO2 above 94%	____________________ liter/min
Fraction of Inspired Oxygen (FiO2)	____________________ %
Partial pressure of oxygen (PaO2)	____________________ mmHg
Partial pressure of carbon dioxid (PaCO2)	____________________ mmHg

Hematology

Centre ID	
Patient ID	
Visit	
Date of sample taken	____ ______ ________

Hematology

Parameter	not done	Result	Unit
Hemoglobin	◯yes		▼
RBC	◯yes		▼
WBC	◯yes		▼
Thrombocytes	◯yes		▼

Blood chemistry and coagulation

Centre ID	
Patient ID	
Visit	
Date of sample taken	____ ______ ________

Clinical chemistry
<small>Please use a point '.' as decimal separator.</small>

Parameter	not done	Result	Unit
BUN	yes		
Creatinine	yes		
Albumin	yes		
AST/SGOT	yes		
ALT/SGPT	yes		
Total bilirubin	yes		
GGT	yes		
AP	yes		
LDH	yes		
Sodium	yes		
Potassium	yes		
Magnesium	yes		
Calcium	yes		
Uric acid	yes		
Troponin	yes		
CK	yes		U/l
CK-MB	yes		U/l
PTT	yes		sec
ATIII	yes		%
D-Dimer	yes		
Fibrinogen	yes		
Ferritin	yes		
Transferrin	yes		
Transferrin Saturation	yes		%
CRP	yes		
Total protein	yes		g/l
IL6	yes		pg/ml
Procalcitonin	yes		ng/ml
Total IgG	yes		
IgA	yes		
IgM	yes		
Lactate	yes		
INR	yes		

SARS-CoV-2 viral clearance and load as well as antibody titres

Centre ID	
Patient ID	
Visit	
Sample collection on Date of visit?	◯yes ◯no
Date of sample collection	____ ______ ________
Type of smear	◯Nasopharyngeal<br>◯Oropharyngeal<br>◯Sputum<br>◯Other
If other: Please specify	____________________
Result of smear	◯Negative ◯Positive ◯Invalide
PCR test type	◯TibMolbiol<br>◯Seegene<br>◯Abbott<br>◯Altona<br>◯Other
If other: Please specify	____________________
CT-value	____________________
Serodiagnostic by ELISA (OD Ratio)	____________________
Neutralize antibody titre (1:....)	____________________ ▼
Immunofluorescence	◯Positive<br>◯Borderline positive<br>◯Negative<br>◯Not performed

Concomitant medication

Centre ID	
Patient ID	
Visit	
Any new or changed concomitant medication since last visit	◯yes ◯no
If yes, please document all changes in Concomitant medications in the following section:	
Link to Concomitant medication	

	Seq. no.	Drug Name	Indicatio n	Start date	Ongoing at end of study	Stop date

Set Link / Create Entry ...

Concomitant COVID-19 therapy

Centre ID

Patient ID

Visit

Any new Concomitant COVID-19 therapy since last visit? ◯yes ◯no

If yes, please document all changes in Concomitant COVID-19 therapy in the following section:

Link to Concomitant COVID-19 therapy

	Therapy number	Therapy	Total Daily Dose	Unit	Onset date of medication	Still ongoing	Stop date of therapy

Set Link / Create Entry ...

Adverse events

Centre ID	
Patient ID	
Visit	
Any new Adverse events since last visit?	○yes ○no
If yes, please document all new Adverse Events in the following section:	
Link to Adverse Events	

	AE number	SOC of AE (according CTCAE V5.0)	AE term (according CTCAE V5.0)	Start date	Ongoing at the end of study	Stop date	Is the AE serious?

Set Link / Create Entry ...

Day 6

Centre ID	
Patient ID	
Visit	Day 6
Date of Visit	____ ______ ________
Hospitalization?	◯yes ◯no

- Vital signs
- Seven point ordinal scale
- Sequential Organ Failure Assessment (SOFA) Score
- Oxygen saturation (SaO2)
- Hematology
- Blood chemistry and coagulation
- Concomitant medication
- Concomitant COVID-19 therapy
- Adverse events

Investigator's Signature

Meaning:	I confirm the completeness and correctness of all documented data.
Signed By:	
Signature Date:	

Vital signs

Centre ID	
Patient ID	
Visit	
WHO performance status	◯ECOG 0 = Fully active, able to carry on all pre-disease performance without restriction<br>◯ECOG 1 = Restricted in physically strenuous activity but ambulatory and able to carry out work of a light or sedentary nature, e.g., light house work, office work<br>◯ECOG 2 = Ambulatory and capable of all selfcare but unable to carry out any work activities; up and about more than 50% of waking hours<br>◯ECOG 3 = Capable of only limited self-care, confined to bed or chair more than 50% of waking hours<br>◯ECOG 4 = Completely disabled. Cannot carry on any self-care. Totally confined to bed or chair.
Body temperature	____________________ °C
Type of measurement	◯oral ◯tympanic
Systolic blood pressure	____________________ mmHg
Diastolic blood pressure	____________________ mmHg
Pulse rate	____________________ beats/min
respiratory rate	____________________ breaths/min

Seven point ordinal scale

Centre ID	
Patient ID	
Visit	
Time of record	____ ____ ____
Ordinal Scale for Clinical Improvement	◯1 = not hospitalized with resumption of normal activities<br>◯2 = not hospitalized, but unable to resume normal sctivities<br>◯3 = hospitalized,not requiring supplemental oxygen<br>◯4 = hospitalized, requiring supplemental oxygen<br>◯5 = hospitalized, requiring nasal high-flow oxygen therapy or, noninvasive mechanical ventilation<br>◯6 = hospitalized, requiring ECMO, invasive mechanical ventilation, or both<br>◯7 = death

Sequential Organ Failure Assessment (SOFA) Score

Centre ID	
Patient ID	
Visit	
Respiratory system (PaO2/FiO2 (mmHg))	> 400<br>< 400<br>< 300<br>< 200 with respiratoy support<br>< 100 with respiratoy support
Nervous system (Glasgow Coma Scale)	15<br>13-14<br>10-12<br>6-9<br><6
Cardiovascular system (Mean arterial pressure (MAP) or administration of vasopressor required)	MAP > 70 mmHg<br>MAP < 70 mmHg<br>Dopamine 5 µg/kg/min or dobutamine any dose<br>Dopamine > 5 µg/kg/min or epinephrine 0.1 µg/kg/min or norepinephrine 0.1 µg/kg/min<br>Dopamine > 15 µg/kg/min OR epinephrine > 0.1 µg/kg/min OR norepinephrine > 0.1 µg/kg/min
Liver (Bilirubin (mg/dl) [µmol/l])	< 1.2 [< 20]<br>1.2 - 1.9 [20 - 32]<br>2.0 - 5.9 [33 - 101]<br>6.0 - 11.9 [102 - 204]<br>> 12.0 [> 204]
Coagulation (Platelets x103/ml)	> 150<br>100-150<br>50-99<br>20-49<br>< 20
Kidneys (Creatinine (mg/dl) [µmol/L]; urine output)	< 1.2 [< 110]<br>1.2 - 1.9 [110 - 170]<br>2.0 - 3.4 [171 - 299]<br>3. - 4.9 [300 - 440]<br>> 5.0 [> 440]

Oxygen saturation (SaO2)

Centre ID	
Patient ID	
Visit	
ECMO	◯yes ◯no
Type of ventilation	◯no<br>◯Nasal high-flow oxygen therapy<br>◯Noninvasive mechanical ventilation<br>◯Invasive ventilation<br>◯Tracheotomy<br>◯Ambient air<br>◯Standard oxygen sources
Amount of oxygen	____________________ liter/min
Oxygen saturation (SaO2)	____________________ %
Amount of supplemental oxygen that is required to keep SaO2 above 94%	____________________ liter/min
Fraction of Inspired Oxygen (FiO2)	____________________ %
Partial pressure of oxygen (PaO2)	____________________ mmHg
Partial pressure of carbon dioxid (PaCO2)	____________________ mmHg

Hematology

Centre ID	
Patient ID	
Visit	
Date of sample taken	____ ______ ________

Hematology

Parameter	not done	Result	Unit
Hemoglobin	◯yes		
RBC	◯yes		
WBC	◯yes		
Thrombocytes	◯yes		

Blood chemistry and coagulation

Centre ID	
Patient ID	
Visit	
Date of sample taken	____ _____ _______

Clinical chemistry
<small>Please use a point '.' as decimal separator.</small>

Parameter	not done	Result	Unit
BUN	yes		
Creatinine	yes		
Albumin	yes		
AST/SGOT	yes		
ALT/SGPT	yes		
Total bilirubin	yes		
GGT	yes		
AP	yes		
LDH	yes		
Sodium	yes		
Potassium	yes		
Magnesium	yes		
Calcium	yes		
Uric acid	yes		
Troponin	yes		
CK	yes		U/l
CK-MB	yes		U/l
PTT	yes		sec
ATIII	yes		%
D-Dimer	yes		
Fibrinogen	yes		
Ferritin	yes		
Transferrin	yes		
Transferrin Saturation	yes		%
CRP	yes		
Total protein	yes		g/l
IL6	yes		pg/ml
Procalcitonin	yes		ng/ml
Total IgG	yes		
IgA	yes		
IgM	yes		
Lactate	yes		
INR	yes		

Concomitant medication

Centre ID	
Patient ID	
Visit	
Any new or changed concomitant medication since last visit	◯yes ◯no
If yes, please document all changes in Concomitant medications in the following section:	
Link to Concomitant medication	

	Seq. no.	Drug Name	Indicatio n	Start date	Ongoing at end of study	Stop date

Set Link / Create Entry ...

Concomitant COVID-19 therapy

Centre ID	
Patient ID	
Visit	
Any new Concomitant COVID-19 therapy since last visit?	◯yes ◯no
If yes, please document all changes in Concomitant COVID-19 therapy in the following section:	
Link to Concomitant COVID-19 therapy	

	Therapy number	Therapy	Total Daily Dose	Unit	Onset date of medication	Still ongoing	Stop date of therapy

Set Link / Create Entry ...

Adverse events

Centre ID	
Patient ID	
Visit	
Any new Adverse events since last visit?	◯yes ◯no
If yes, please document all new Adverse Events in the following section:	
Link to Adverse Events	(see table below)

	AE number	SOC of AE (according CTCAE V5.0)	AE term (according CTCAE V5.0)	Start date	Ongoing at the end of study	Stop date	Is the AE serious?

Set Link / Create Entry ...

Day 7

Centre ID	
Patient ID	
Visit	Day 7
Date of Visit	____ ______ ________
Hospitalization?	◯yes ◯no

- Vital signs
- Seven point ordinal scale
- Sequential Organ Failure Assessment (SOFA) Score
- Oxygen saturation (SaO2)
- Hematology
- Blood chemistry and coagulation
- SARS-CoV-2 viral clearance and load as well as antibody titres
- Concomitant medication
- Concomitant COVID-19 therapy
- Adverse events

Investigator's Signature

Meaning:	I confirm the completeness and correctness of all documented data.
Signed By:	
Signature Date:	

Vital signs

Centre ID	
Patient ID	
Visit	
WHO performance status	◯ECOG 0 = Fully active, able to carry on all pre-disease performance without restriction<br>◯ECOG 1 = Restricted in physically strenuous activity but ambulatory and able to carry out work of a light or sedentary nature, e.g., light house work, office work<br>◯ECOG 2 = Ambulatory and capable of all selfcare but unable to carry out any work activities; up and about more than 50% of waking hours<br>◯ECOG 3 = Capable of only limited self-care, confined to bed or chair more than 50% of waking hours<br>◯ECOG 4 = Completely disabled. Cannot carry on any self-care. Totally confined to bed or chair.
Body temperature	____________________ °C
Type of measurement	◯oral ◯tympanic
Systolic blood pressure	____________________ mmHg
Diastolic blood pressure	____________________ mmHg
Pulse rate	____________________ beats/min
respiratory rate	____________________ breaths/min

Seven point ordinal scale

Centre ID	
Patient ID	
Visit	
Time of record	____ ____ ____
Ordinal Scale for Clinical Improvement	◯1 = not hospitalized with resumption of normal activities<br>◯2 = not hospitalized, but unable to resume normal sctivities<br>◯3 = hospitalized,not requiring supplemental oxygen<br>◯4 = hospitalized, requiring supplemental oxygen<br>◯5 = hospitalized, requiring nasal high-flow oxygen therapy or, noninvasive mechanical ventilation<br>◯6 = hospitalized, requiring ECMO, invasive mechanical ventilation, or both<br>◯7 = death

Sequential Organ Failure Assessment (SOFA) Score

Centre ID	
Patient ID	
Visit	
Respiratory system (PaO2/FiO2 (mmHg))	◯> 400<br>◯< 400<br>◯< 300<br>◯< 200 with respiratoy support<br>◯< 100 with respiratoy support
Nervous system (Glasgow Coma Scale)	◯15<br>◯13-14<br>◯10-12<br>◯6-9<br>◯<6
Cardiovascular system (Mean arterial pressure (MAP) or administration of vasopressor required)	◯MAP > 70 mmHg<br>◯MAP < 70 mmHg<br>◯Dopamine 5 µg/kg/min or dobutamine any dose<br>◯Dopamine > 5 µg/kg/min or epinephrine 0.1 µg/kg/min or norepinephrine 0.1 µg/kg/min<br>◯Dopamine > 15 µg/kg/min OR epinephrine > 0.1 µg/kg/min OR norepinephrine > 0.1 µg/kg/min
Liver (Bilirubin (mg/dl) [µmol/l])	◯< 1.2 [< 20]<br>◯1.2 - 1.9 [20 - 32]<br>◯2.0 - 5.9 [33 - 101]<br>◯6.0 - 11.9 [102 - 204]<br>◯> 12.0 [> 204]
Coagulation (Platelets x103/ml)	◯> 150<br>◯100-150<br>◯50-99<br>◯20-49<br>◯< 20
Kidneys (Creatinine (mg/dl) [µmol/L]; urine output)	◯< 1.2 [< 110]<br>◯1.2 - 1.9 [110 - 170]<br>◯2.0 - 3.4 [171 - 299]<br>◯3. - 4.9 [300 - 440]<br>◯> 5.0 [> 440]

Oxygen saturation (SaO2)

Centre ID	
Patient ID	
Visit	
ECMO	◯yes ◯no
Type of ventilation	◯no<br>◯Nasal high-flow oxygen therapy<br>◯Noninvasive mechanical ventilation<br>◯Invasive ventilation<br>◯Tracheotomy<br>◯Ambient air<br>◯Standard oxygen sources
Amount of oxygen	____________________ liter/min
Oxygen saturation (SaO2)	____________________ %
Amount of supplemental oxygen that is required to keep SaO2 above 94%	____________________ liter/min
Fraction of Inspired Oxygen (FiO2)	____________________ %
Partial pressure of oxygen (PaO2)	____________________ mmHg
Partial pressure of carbon dioxid (PaCO2)	____________________ mmHg

Hematology

Centre ID	
Patient ID	
Visit	
Date of sample taken	____ ______ ________

Hematology

Parameter	not done	Result	Unit
Hemoglobin	◯yes		▾
RBC	◯yes		▾
WBC	◯yes		▾
Thrombocytes	◯yes		▾

Blood chemistry and coagulation

Centre ID	
Patient ID	
Visit	
Date of sample taken	____ ______ ________

Clinical chemistry
<small>Please use a point '.' as decimal separator.</small>

Parameter	not done	Result	Unit
BUN	yes		
Creatinine	yes		
Albumin	yes		
AST/SGOT	yes		
ALT/SGPT	yes		
Total bilirubin	yes		
GGT	yes		
AP	yes		
LDH	yes		
Sodium	yes		
Potassium	yes		
Magnesium	yes		
Calcium	yes		
Uric acid	yes		
Troponin	yes		
CK	yes		U/l
CK-MB	yes		U/l
PTT	yes		sec
ATIII	yes		%
D-Dimer	yes		
Fibrinogen	yes		
Ferritin	yes		
Transferrin	yes		
Transferrin Saturation	yes		%
CRP	yes		
Total protein	yes		g/l
IL6	yes		pg/ml
Procalcitonin	yes		ng/ml
Total IgG	yes		
IgA	yes		
IgM	yes		
Lactate	yes		
INR	yes		

SARS-CoV-2 viral clearance and load as well as antibody titres

Centre ID	
Patient ID	
Visit	
Sample collection on Date of visit?	◯yes ◯no
Date of sample collection	____ ______ ________
Type of smear	◯Nasopharyngeal<br>◯Oropharyngeal<br>◯Sputum<br>◯Other
If other: Please specify	____________________
Result of smear	◯Negative ◯Positive ◯Invalide
PCR test type	◯TibMolbiol<br>◯Seegene<br>◯Abbott<br>◯Altona<br>◯Other
If other: Please specify	____________________
CT-value	____________________
Serodiagnostic by ELISA (OD Ratio)	____________________
Neutralize antibody titre (1:....)	____________________ ▼
Immunofluorescence	◯Positive<br>◯Borderline positive<br>◯Negative<br>◯Not performed

Concomitant medication

Centre ID	
Patient ID	
Visit	
Any new or changed concomitant medication since last visit	◯yes ◯no
If yes, please document all changes in Concomitant medications in the following section:	
Link to Concomitant medication	

	Seq. no.	Drug Name	Indication	Start date	Ongoing at end of study	Stop date

Set Link / Create Entry ...

Concomitant COVID-19 therapy

Centre ID

Patient ID

Visit

Any new Concomitant COVID-19 therapy since last visit? ◯yes ◯no

If yes, please document all changes in Concomitant COVID-19 therapy in the following section:

Link to Concomitant COVID-19 therapy

	Therapy number	Therapy	Total Daily Dose	Unit	Onset date of medication	Still ongoing	Stop date of therapy

Set Link / Create Entry ...

Adverse events

Centre ID	
Patient ID	
Visit	
Any new Adverse events since last visit?	◯yes ◯no
If yes, please document all new Adverse Events in the following section:	
Link to Adverse Events	

	AE number	SOC of AE (according CTCAE V5.0)	AE term (according CTCAE V5.0)	Start date	Ongoing at the end of study	Stop date	Is the AE serious?

Set Link / Create Entry ...

Day 8

Centre ID	
Patient ID	
Visit	Day 8
Date of Visit	____ ______ ________
Hospitalization?	◯yes ◯no

- Vital signs
- Seven point ordinal scale
- Sequential Organ Failure Assessment (SOFA) Score
- Oxygen saturation (SaO2)
- Hematology
- Blood chemistry and coagulation
- Concomitant medication
- Concomitant COVID-19 therapy
- Adverse events

Investigator's Signature

Meaning:	I confirm the completeness and correctness of all documented data.
Signed By:	
Signature Date:	

Vital signs

Centre ID	
Patient ID	
Visit	
WHO performance status	◯ECOG 0 = Fully active, able to carry on all pre-disease performance without restriction<br>◯ECOG 1 = Restricted in physically strenuous activity but ambulatory and able to carry out work of a light or sedentary nature, e.g., light house work, office work<br>◯ECOG 2 = Ambulatory and capable of all selfcare but unable to carry out any work activities; up and about more than 50% of waking hours<br>◯ECOG 3 = Capable of only limited self-care, confined to bed or chair more than 50% of waking hours<br>◯ECOG 4 = Completely disabled. Cannot carry on any self-care. Totally confined to bed or chair.
Body temperature	____________________ °C
Type of measurement	◯oral ◯tympanic
Systolic blood pressure	____________________ mmHg
Diastolic blood pressure	____________________ mmHg
Pulse rate	____________________ beats/min
respiratory rate	____________________ breaths/min

Seven point ordinal scale

Centre ID	
Patient ID	
Visit	
Time of record	____ ____ ____
Ordinal Scale for Clinical Improvement	◯1 = not hospitalized with resumption of normal activities<br>◯2 = not hospitalized, but unable to resume normal sctivities<br>◯3 = hospitalized,not requiring supplemental oxygen<br>◯4 = hospitalized, requiring supplemental oxygen<br>◯5 = hospitalized, requiring nasal high-flow oxygen therapy or, noninvasive mechanical ventilation<br>◯6 = hospitalized, requiring ECMO, invasive mechanical ventilation, or both<br>◯7 = death

Sequential Organ Failure Assessment (SOFA) Score

Centre ID	
Patient ID	
Visit	
Respiratory system (PaO2/FiO2 (mmHg))	◯> 400<br>◯< 400<br>◯< 300<br>◯< 200 with respiratoy support<br>◯< 100 with respiratoy support
Nervous system (Glasgow Coma Scale)	◯15<br>◯13-14<br>◯10-12<br>◯6-9<br>◯<6
Cardiovascular system (Mean arterial pressure (MAP) or administration of vasopressor required)	◯MAP > 70 mmHg<br>◯MAP < 70 mmHg<br>◯Dopamine 5 µg/kg/min or dobutamine any dose<br>◯Dopamine > 5 µg/kg/min or epinephrine 0.1 µg/kg/min or norepinephrine 0.1 µg/kg/min<br>◯Dopamine > 15 µg/kg/min OR epinephrine > 0.1 µg/kg/min OR norepinephrine > 0.1 µg/kg/min
Liver (Bilirubin (mg/dl) [µmol/l])	◯< 1.2 [< 20]<br>◯1.2 - 1.9 [20 - 32]<br>◯2.0 - 5.9 [33 - 101]<br>◯6.0 - 11.9 [102 - 204]<br>◯> 12.0 [> 204]
Coagulation (Platelets x103/ml)	◯> 150<br>◯100-150<br>◯50-99<br>◯20-49<br>◯< 20
Kidneys (Creatinine (mg/dl) [µmol/L]; urine output)	◯< 1.2 [< 110]<br>◯1.2 - 1.9 [110 - 170]<br>◯2.0 - 3.4 [171 - 299]<br>◯3. - 4.9 [300 - 440]<br>◯> 5.0 [> 440]

Oxygen saturation (SaO2)

Centre ID	
Patient ID	
Visit	
ECMO	◯yes ◯no
Type of ventilation	◯no<br>◯Nasal high-flow oxygen therapy<br>◯Noninvasive mechanical ventilation<br>◯Invasive ventilation<br>◯Tracheotomy<br>◯Ambient air<br>◯Standard oxygen sources
Amount of oxygen	____________________ liter/min
Oxygen saturation (SaO2)	____________________ %
Amount of supplemental oxygen that is required to keep SaO2 above 94%	____________________ liter/min
Fraction of Inspired Oxygen (FiO2)	____________________ %
Partial pressure of oxygen (PaO2)	____________________ mmHg
Partial pressure of carbon dioxid (PaCO2)	____________________ mmHg

Hematology

Centre ID	
Patient ID	
Visit	
Date of sample taken	____ ______ ________

Hematology

Parameter	not done	Result	Unit
Hemoglobin	◯yes		
RBC	◯yes		
WBC	◯yes		
Thrombocytes	◯yes		

Blood chemistry and coagulation

Centre ID	
Patient ID	
Visit	
Date of sample taken	____ ______ ________

Clinical chemistry
<small>Please use a point '.' as decimal separator.</small>

Parameter	not done	Result	Unit
BUN	yes		
Creatinine	yes		
Albumin	yes		
AST/SGOT	yes		
ALT/SGPT	yes		
Total bilirubin	yes		
GGT	yes		
AP	yes		
LDH	yes		
Sodium	yes		
Potassium	yes		
Magnesium	yes		
Calcium	yes		
Uric acid	yes		
Troponin	yes		
CK	yes		U/l
CK-MB	yes		U/l
PTT	yes		sec
ATIII	yes		%
D-Dimer	yes		
Fibrinogen	yes		
Ferritin	yes		
Transferrin	yes		
Transferrin Saturation	yes		%
CRP	yes		
Total protein	yes		g/l
IL6	yes		pg/ml
Procalcitonin	yes		ng/ml
Total IgG	yes		
IgA	yes		
IgM	yes		
Lactate	yes		
INR	yes		

Concomitant medication

Centre ID	
Patient ID	
Visit	
Any new or changed concomitant medication since last visit	◯yes ◯no
If yes, please document all changes in Concomitant medications in the following section:	
Link to Concomitant medication	

	Seq. no.	Drug Name	Indicatio n	Start date	Ongoing at end of study	Stop date

Set Link / Create Entry ...

Concomitant COVID-19 therapy

Centre ID	
Patient ID	
Visit	
Any new Concomitant COVID-19 therapy since last visit?	◯yes ◯no

If yes, please document all changes in Concomitant COVID-19 therapy in the following section:

Link to Concomitant COVID-19 therapy

	Therapy number	Therapy	Total Daily Dose	Unit	Onset date of medication	Still ongoing	Stop date of therapy

Set Link / Create Entry ...

Adverse events

Centre ID	
Patient ID	
Visit	
Any new Adverse events since last visit?	◯yes ◯no
If yes, please document all new Adverse Events in the following section:	
Link to Adverse Events	(see table below)

	AE number	SOC of AE (according CTCAE V5.0)	AE term (according CTCAE V5.0)	Start date	Ongoing at the end of study	Stop date	Is the AE serious?

Set Link / Create Entry ...

Day 9

Centre ID	
Patient ID	
Visit	Day 9
Date of Visit	____ ______ ________
Hospitalization?	◯yes ◯no

- Vital signs
- Seven point ordinal scale
- Sequential Organ Failure Assessment (SOFA) Score
- Oxygen saturation (SaO2)
- Hematology
- Blood chemistry and coagulation
- Concomitant medication
- Concomitant COVID-19 therapy
- Adverse events

Investigator's Signature

Meaning:	I confirm the completeness and correctness of all documented data.
Signed By:	
Signature Date:	

Vital signs

Centre ID	
Patient ID	
Visit	
WHO performance status	◯ECOG 0 = Fully active, able to carry on all pre-disease performance without restriction<br>◯ECOG 1 = Restricted in physically strenuous activity but ambulatory and able to carry out work of a light or sedentary nature, e.g., light house work, office work<br>◯ECOG 2 = Ambulatory and capable of all selfcare but unable to carry out any work activities; up and about more than 50% of waking hours<br>◯ECOG 3 = Capable of only limited self-care, confined to bed or chair more than 50% of waking hours<br>◯ECOG 4 = Completely disabled. Cannot carry on any self-care. Totally confined to bed or chair.
Body temperature	____________________ °C
Type of measurement	◯oral ◯tympanic
Systolic blood pressure	____________________ mmHg
Diastolic blood pressure	____________________ mmHg
Pulse rate	____________________ beats/min
respiratory rate	____________________ breaths/min

Seven point ordinal scale

Centre ID	
Patient ID	
Visit	
Time of record	____ ____ ____
Ordinal Scale for Clinical Improvement	◯1 = not hospitalized with resumption of normal activities<br>◯2 = not hospitalized, but unable to resume normal sctivities<br>◯3 = hospitalized,not requiring supplemental oxygen<br>◯4 = hospitalized, requiring supplemental oxygen<br>◯5 = hospitalized, requiring nasal high-flow oxygen therapy or, noninvasive mechanical ventilation<br>◯6 = hospitalized, requiring ECMO, invasive mechanical ventilation, or both<br>◯7 = death

Sequential Organ Failure Assessment (SOFA) Score

Centre ID	
Patient ID	
Visit	
Respiratory system (PaO2/FiO2 (mmHg))	> 400<br>< 400<br>< 300<br>< 200 with respiratoy support<br>< 100 with respiratoy support
Nervous system (Glasgow Coma Scale)	15<br>13-14<br>10-12<br>6-9<br><6
Cardiovascular system (Mean arterial pressure (MAP) or administration of vasopressor required)	MAP > 70 mmHg<br>MAP < 70 mmHg<br>Dopamine 5 µg/kg/min or dobutamine any dose<br>Dopamine > 5 µg/kg/min or epinephrine 0.1 µg/kg/min or norepinephrine 0.1 µg/kg/min<br>Dopamine > 15 µg/kg/min OR epinephrine > 0.1 µg/kg/min OR norepinephrine > 0.1 µg/kg/min
Liver (Bilirubin (mg/dl) [µmol/l])	< 1.2 [< 20]<br>1.2 - 1.9 [20 - 32]<br>2.0 - 5.9 [33 - 101]<br>6.0 - 11.9 [102 - 204]<br>> 12.0 [> 204]
Coagulation (Platelets x103/ml)	> 150<br>100-150<br>50-99<br>20-49<br>< 20
Kidneys (Creatinine (mg/dl) [µmol/L]; urine output)	< 1.2 [< 110]<br>1.2 - 1.9 [110 - 170]<br>2.0 - 3.4 [171 - 299]<br>3. - 4.9 [300 - 440]<br>> 5.0 [> 440]

Oxygen saturation (SaO2)

Centre ID	
Patient ID	
Visit	
ECMO	◯yes ◯no
Type of ventilation	◯no<br>◯Nasal high-flow oxygen therapy<br>◯Noninvasive mechanical ventilation<br>◯Invasive ventilation<br>◯Tracheotomy<br>◯Ambient air<br>◯Standard oxygen sources
Amount of oxygen	____________________ liter/min
Oxygen saturation (SaO2)	____________________ %
Amount of supplemental oxygen that is required to keep SaO2 above 94%	____________________ liter/min
Fraction of Inspired Oxygen (FiO2)	____________________ %
Partial pressure of oxygen (PaO2)	____________________ mmHg
Partial pressure of carbon dioxid (PaCO2)	____________________ mmHg

Hematology

Centre ID	
Patient ID	
Visit	
Date of sample taken	____ ______ ________

Hematology

Parameter	not done	Result	Unit
Hemoglobin	yes		
RBC	yes		
WBC	yes		
Thrombocytes	yes		

Blood chemistry and coagulation

Centre ID	
Patient ID	
Visit	
Date of sample taken	____ ______ ________

Clinical chemistry
<small>Please use a point '.' as decimal separator.</small>

Parameter	not done	Result	Unit
BUN	yes		
Creatinine	yes		
Albumin	yes		
AST/SGOT	yes		
ALT/SGPT	yes		
Total bilirubin	yes		
GGT	yes		
AP	yes		
LDH	yes		
Sodium	yes		
Potassium	yes		
Magnesium	yes		
Calcium	yes		
Uric acid	yes		
Troponin	yes		
CK	yes		U/l
CK-MB	yes		U/l
PTT	yes		sec
ATIII	yes		%
D-Dimer	yes		
Fibrinogen	yes		
Ferritin	yes		
Transferrin	yes		
Transferrin Saturation	yes		%
CRP	yes		
Total protein	yes		g/l
IL6	yes		pg/ml
Procalcitonin	yes		ng/ml
Total IgG	yes		
IgA	yes		
IgM	yes		
Lactate	yes		
INR	yes		

Concomitant medication

Centre ID	
Patient ID	
Visit	
Any new or changed concomitant medication since last visit	◯yes ◯no

If yes, please document all changes in Concomitant medications in the following section:

Link to Concomitant medication

	Seq. no.	Drug Name	Indication	Start date	Ongoing at end of study	Stop date

Set Link / Create Entry ...

Concomitant COVID-19 therapy

Centre ID	
Patient ID	
Visit	
Any new Concomitant COVID-19 therapy since last visit?	◯yes ◯no

If yes, please document all changes in Concomitant COVID-19 therapy in the following section:

Link to Concomitant COVID-19 therapy

	Therapy number	Therapy	Total Daily Dose	Unit	Onset date of medication	Still ongoing	Stop date of therapy

Set Link / Create Entry ...

Adverse events

Centre ID	
Patient ID	
Visit	
Any new Adverse events since last visit?	◯yes ◯no
If yes, please document all new Adverse Events in the following section:	
Link to Adverse Events	see table below

	AE number	SOC of AE (according CTCAE V5.0)	AE term (according CTCAE V5.0)	Start date	Ongoing at the end of study	Stop date	Is the AE serious?

Set Link / Create Entry ...

Day 10

Centre ID	
Patient ID	
Visit	Day 10
Date of Visit	____ ______ ________
Hospitalization?	◯yes ◯no

- Vital signs
- Seven point ordinal scale
- Sequential Organ Failure Assessment (SOFA) Score
- Oxygen saturation (SaO2)
- Hematology
- Blood chemistry and coagulation
- SARS-CoV-2 viral clearance and load as well as antibody titres
- Concomitant medication
- Concomitant COVID-19 therapy
- Adverse events

Investigator's Signature

Meaning:	I confirm the completeness and correctness of all documented data.
Signed By:	
Signature Date:	

Vital signs

Centre ID	
Patient ID	
Visit	
WHO performance status	◯ECOG 0 = Fully active, able to carry on all pre-disease performance without restriction<br>◯ECOG 1 = Restricted in physically strenuous activity but ambulatory and able to carry out work of a light or sedentary nature, e.g., light house work, office work<br>◯ECOG 2 = Ambulatory and capable of all selfcare but unable to carry out any work activities; up and about more than 50% of waking hours<br>◯ECOG 3 = Capable of only limited self-care, confined to bed or chair more than 50% of waking hours<br>◯ECOG 4 = Completely disabled. Cannot carry on any self-care. Totally confined to bed or chair.
Body temperature	____________________ °C
Type of measurement	◯oral ◯tympanic
Systolic blood pressure	____________________ mmHg
Diastolic blood pressure	____________________ mmHg
Pulse rate	____________________ beats/min
respiratory rate	____________________ breaths/min

Seven point ordinal scale

Centre ID	
Patient ID	
Visit	
Time of record	____ ____ ____
Ordinal Scale for Clinical Improvement	◯1 = not hospitalized with resumption of normal activities<br>◯2 = not hospitalized, but unable to resume normal sctivities<br>◯3 = hospitalized,not requiring supplemental oxygen<br>◯4 = hospitalized, requiring supplemental oxygen<br>◯5 = hospitalized, requiring nasal high-flow oxygen therapy or, noninvasive mechanical ventilation<br>◯6 = hospitalized, requiring ECMO, invasive mechanical ventilation, or both<br>◯7 = death

Sequential Organ Failure Assessment (SOFA) Score

Centre ID	
Patient ID	
Visit	
Respiratory system (PaO2/FiO2 (mmHg))	◯> 400<br>◯< 400<br>◯< 300<br>◯< 200 with respiratoy support<br>◯< 100 with respiratoy support
Nervous system (Glasgow Coma Scale)	◯15<br>◯13-14<br>◯10-12<br>◯6-9<br>◯<6
Cardiovascular system (Mean arterial pressure (MAP) or administration of vasopressor required)	◯MAP > 70 mmHg<br>◯MAP < 70 mmHg<br>◯Dopamine 5 µg/kg/min or dobutamine any dose<br>◯Dopamine > 5 µg/kg/min or epinephrine 0.1 µg/kg/min or norepinephrine 0.1 µg/kg/min<br>◯Dopamine > 15 µg/kg/min OR epinephrine > 0.1 µg/kg/min OR norepinephrine > 0.1 µg/kg/min
Liver (Bilirubin (mg/dl) [µmol/l])	◯< 1.2 [< 20]<br>◯1.2 - 1.9 [20 - 32]<br>◯2.0 - 5.9 [33 - 101]<br>◯6.0 - 11.9 [102 - 204]<br>◯> 12.0 [> 204]
Coagulation (Platelets x103/ml)	◯> 150<br>◯100-150<br>◯50-99<br>◯20-49<br>◯< 20
Kidneys (Creatinine (mg/dl) [µmol/L]; urine output)	◯< 1.2 [< 110]<br>◯1.2 - 1.9 [110 - 170]<br>◯2.0 - 3.4 [171 - 299]<br>◯3. - 4.9 [300 - 440]<br>◯> 5.0 [> 440]

Oxygen saturation (SaO2)

Centre ID	
Patient ID	
Visit	
ECMO	◯yes ◯no
Type of ventilation	◯no<br>◯Nasal high-flow oxygen therapy<br>◯Noninvasive mechanical ventilation<br>◯Invasive ventilation<br>◯Tracheotomy<br>◯Ambient air<br>◯Standard oxygen sources
Amount of oxygen	____________________ liter/min
Oxygen saturation (SaO2)	____________________ %
Amount of supplemental oxygen that is required to keep SaO2 above 94%	____________________ liter/min
Fraction of Inspired Oxygen (FiO2)	____________________ %
Partial pressure of oxygen (PaO2)	____________________ mmHg
Partial pressure of carbon dioxid (PaCO2)	____________________ mmHg

Hematology

Centre ID	
Patient ID	
Visit	
Date of sample taken	____ _____ ______

Hematology

Parameter	not done	Result	Unit
Hemoglobin	◯yes		
RBC	◯yes		
WBC	◯yes		
Thrombocytes	◯yes		

Blood chemistry and coagulation

Centre ID	
Patient ID	
Visit	
Date of sample taken	___ ____ ______

Clinical chemistry
<small>Please use a point '.' as decimal separator.</small>

Parameter	not done	Result	Unit
BUN	yes		
Creatinine	yes		
Albumin	yes		
AST/SGOT	yes		
ALT/SGPT	yes		
Total bilirubin	yes		
GGT	yes		
AP	yes		
LDH	yes		
Sodium	yes		
Potassium	yes		
Magnesium	yes		
Calcium	yes		
Uric acid	yes		
Troponin	yes		
CK	yes		U/l
CK-MB	yes		U/l
PTT	yes		sec
ATIII	yes		%
D-Dimer	yes		
Fibrinogen	yes		
Ferritin	yes		
Transferrin	yes		
Transferrin Saturation	yes		%
CRP	yes		
Total protein	yes		g/l
IL6	yes		pg/ml
Procalcitonin	yes		ng/ml
Total IgG	yes		
IgA	yes		
IgM	yes		
Lactate	yes		
INR	yes		

SARS-CoV-2 viral clearance and load as well as antibody titres

Centre ID	
Patient ID	
Visit	
Sample collection on Date of visit?	◯yes ◯no
Date of sample collection	____ ______ ________
Type of smear	◯Nasopharyngeal<br>◯Oropharyngeal<br>◯Sputum<br>◯Other
If other: Please specify	____________________
Result of smear	◯Negative ◯Positive ◯Invalide
PCR test type	◯TibMolbiol<br>◯Seegene<br>◯Abbott<br>◯Altona<br>◯Other
If other: Please specify	____________________
CT-value	____________________
Serodiagnostic by ELISA (OD Ratio)	____________________
Neutralize antibody titre (1:....)	____________________ ▾
Immunofluorescence	◯Positive<br>◯Borderline positive<br>◯Negative<br>◯Not performed

Concomitant medication

Centre ID	
Patient ID	
Visit	
Any new or changed concomitant medication since last visit	◯yes ◯no

If yes, please document all changes in Concomitant medications in the following section:

Link to Concomitant medication

	Seq. no.	Drug Name	Indicatio n	Start date	Ongoing at end of study	Stop date

Set Link / Create Entry ...

Concomitant COVID-19 therapy

Centre ID	
Patient ID	
Visit	
Any new Concomitant COVID-19 therapy since last visit?	◯yes ◯no
If yes, please document all changes in Concomitant COVID-19 therapy in the following section:	
Link to Concomitant COVID-19 therapy	

	Therapy number	Therapy	Total Daily Dose	Unit	Onset date of medication	Still ongoing	Stop date of therapy

Set Link / Create Entry ...

Adverse events

Centre ID	
Patient ID	
Visit	
Any new Adverse events since last visit?	◯yes ◯no
If yes, please document all new Adverse Events in the following section:	
Link to Adverse Events	see table below

	AE number	SOC of AE (according CTCAE V5.0)	AE term (according CTCAE V5.0)	Start date	Ongoing at the end of study	Stop date	Is the AE serious?

Set Link / Create Entry ...

Day 1 - Cross-over Assessment

Centre ID	
Patient ID	
Visit	Day 1 - Cross-over Assessment
Date of Visit	____ ______ ________

- Vital signs
- Seven point ordinal scale
- Sequential Organ Failure Assessment (SOFA) Score
- Oxygen saturation (SaO2)
- Hematology
- Blood chemistry and coagulation
- SARS-CoV-2 viral clearance and load as well as antibody titres
- Procurement of Samples for Biobanking
- Administration of convalescent plasma
- Concomitant medication
- Concomitant COVID-19 therapy
- Adverse events

Investigator's Signature

Meaning:	I confirm the completeness and correctness of all documented data.
Signed By:	
Signature Date:	

Vital signs

Centre ID	
Patient ID	
Visit	
WHO performance status	◯ECOG 0 = Fully active, able to carry on all pre-disease performance without restriction<br>◯ECOG 1 = Restricted in physically strenuous activity but ambulatory and able to carry out work of a light or sedentary nature, e.g., light house work, office work<br>◯ECOG 2 = Ambulatory and capable of all selfcare but unable to carry out any work activities; up and about more than 50% of waking hours<br>◯ECOG 3 = Capable of only limited self-care, confined to bed or chair more than 50% of waking hours<br>◯ECOG 4 = Completely disabled. Cannot carry on any self-care. Totally confined to bed or chair.
Body temperature	____________________ °C
Type of measurement	◯oral ◯tympanic
Systolic blood pressure	____________________ mmHg
Diastolic blood pressure	____________________ mmHg
Pulse rate	____________________ beats/min
respiratory rate	____________________ breaths/min

Seven point ordinal scale

Centre ID	
Patient ID	
Visit	
Time of record	____ ____ ____
Ordinal Scale for Clinical Improvement	◯1 = not hospitalized with resumption of normal activities<br>◯2 = not hospitalized, but unable to resume normal sctivities<br>◯3 = hospitalized,not requiring supplemental oxygen<br>◯4 = hospitalized, requiring supplemental oxygen<br>◯5 = hospitalized, requiring nasal high-flow oxygen therapy or, noninvasive mechanical ventilation<br>◯6 = hospitalized, requiring ECMO, invasive mechanical ventilation, or both<br>◯7 = death

Sequential Organ Failure Assessment (SOFA) Score

Centre ID	
Patient ID	
Visit	
Respiratory system (PaO2/FiO2 (mmHg))	◯> 400<br>◯< 400<br>◯< 300<br>◯< 200 with respiratoy support<br>◯< 100 with respiratoy support
Nervous system (Glasgow Coma Scale)	◯15<br>◯13-14<br>◯10-12<br>◯6-9<br>◯<6
Cardiovascular system (Mean arterial pressure (MAP) or administration of vasopressor required)	◯MAP > 70 mmHg<br>◯MAP < 70 mmHg<br>◯Dopamine 5 µg/kg/min or dobutamine any dose<br>◯Dopamine > 5 µg/kg/min or epinephrine 0.1 µg/kg/min or norepinephrine 0.1 µg/kg/min<br>◯Dopamine > 15 µg/kg/min OR epinephrine > 0.1 µg/kg/min OR norepinephrine > 0.1 µg/kg/min
Liver (Bilirubin (mg/dl) [µmol/l])	◯< 1.2 [< 20]<br>◯1.2 - 1.9 [20 - 32]<br>◯2.0 - 5.9 [33 - 101]<br>◯6.0 - 11.9 [102 - 204]<br>◯> 12.0 [> 204]
Coagulation (Platelets x103/ml)	◯> 150<br>◯100-150<br>◯50-99<br>◯20-49<br>◯< 20
Kidneys (Creatinine (mg/dl) [µmol/L]; urine output)	◯< 1.2 [< 110]<br>◯1.2 - 1.9 [110 - 170]<br>◯2.0 - 3.4 [171 - 299]<br>◯3. - 4.9 [300 - 440]<br>◯> 5.0 [> 440]

Oxygen saturation (SaO2)

Centre ID	
Patient ID	
Visit	
ECMO	◯yes ◯no
Type of ventilation	◯no<br>◯Nasal high-flow oxygen therapy<br>◯Noninvasive mechanical ventilation<br>◯Invasive ventilation<br>◯Tracheotomy<br>◯Ambient air<br>◯Standard oxygen sources
Amount of oxygen	____________________ liter/min
Oxygen saturation (SaO2)	____________________ %
Amount of supplemental oxygen that is required to keep SaO2 above 94%	____________________ liter/min
Fraction of Inspired Oxygen (FiO2)	____________________ %
Partial pressure of oxygen (PaO2)	____________________ mmHg
Partial pressure of carbon dioxid (PaCO2)	____________________ mmHg

Hematology

Centre ID	
Patient ID	
Visit	
Date of sample taken	____ ______ ________

Hematology

Parameter	not done	Result	Unit
Hemoglobin	◯yes		▾
RBC	◯yes		▾
WBC	◯yes		▾
Thrombocytes	◯yes		▾

Blood chemistry and coagulation

Centre ID	
Patient ID	
Visit	
Date of sample taken	___ ____ ______

Clinical chemistry
<small>Please use a point '.' as decimal separator.</small>

Parameter	not done	Result	Unit
BUN	yes		
Creatinine	yes		
Albumin	yes		
AST/SGOT	yes		
ALT/SGPT	yes		
Total bilirubin	yes		
GGT	yes		
AP	yes		
LDH	yes		
Sodium	yes		
Potassium	yes		
Magnesium	yes		
Calcium	yes		
Uric acid	yes		
Troponin	yes		
CK	yes		U/l
CK-MB	yes		U/l
PTT	yes		sec
ATIII	yes		%
D-Dimer	yes		
Fibrinogen	yes		
Ferritin	yes		
Transferrin	yes		
Transferrin Saturation	yes		%
CRP	yes		
Total protein	yes		g/l
IL6	yes		pg/ml
Procalcitonin	yes		ng/ml
Total IgG	yes		
IgA	yes		
IgM	yes		
Lactate	yes		
INR	yes		

SARS-CoV-2 viral clearance and load as well as antibody titres

Centre ID	
Patient ID	
Visit	
Sample collection on Date of visit?	◯yes ◯no
Date of sample collection	____ ______ ________
Type of smear	◯Nasopharyngeal<br>◯Oropharyngeal<br>◯Sputum<br>◯Other
If other: Please specify	____________________
Result of smear	◯Negative ◯Positive ◯Invalide
PCR test type	◯TibMolbiol<br>◯Seegene<br>◯Abbott<br>◯Altona<br>◯Other
If other: Please specify	____________________
CT-value	____________________
Serodiagnostic by ELISA (OD Ratio)	____________________
Neutralize antibody titre (1:....)	____________________ ▾
Immunofluorescence	◯Positive<br>◯Borderline positive<br>◯Negative<br>◯Not performed

Procurement of Samples for Biobanking

Centre ID	
Patient ID	
Visit	
1 x 7,5 ml serum taken?	◯yes ◯no
Sample collection on Date of visit?	◯yes ◯no
Date of collection	____ ______ ________
Time of collection	____ ____ ____
Material	◯complete ◯less material
if no: Reason	____________________
2 x 7,5 ml Lithium-heparin taken?	◯yes ◯no
Sample collection on Date of visit?	◯yes ◯no
Date of collection	____ ______ ________
Time of collection	____ ____ ____
Material	◯complete ◯less material
if no: Reason	____________________

Administration of convalescent plasma

Centre ID	
Patient ID	
Visit	
Batch number of convalascent plasma	________
Time of infusion start	____ ____ ____
Time of infusion stop	____ ____ ____
Amount of infusion	_____________________ ml
Transfusion reactions	◯No<br>◯Transfusion associated circulatory overload (TACO)<br>◯Citrate intoxication<br>◯Allergic reaction<br>◯Transfusion transmitted infection<br>◯Transfusion associated lung injury (TRALI)<br>◯Other
if other: Please specify	________________
Treatment discontinuation	◯yes ◯no
Reason for treatment discontinution	◯Adverse event (other than allergic reaction) with the transfusion of CP<br>◯Serious Adverse Event upon CP transfusion<br>◯Allergic reaction

Continuation of CP treatment after AE resolution to smaller than grade 2 according to CTCAE

■ Date of continuation	____ ______ ________
■ Time of infusion start	____ ____ ____
■ Time of infusion stop	____ ____ ____
■ Amount of infusion	_____________________ ml
■ Transfusion reactions	◯No<br>◯Transfusion associated circulatory overload (TACO)<br>◯Citrate intoxication<br>◯Allergic reaction<br>◯Transfusion transmitted infection<br>◯Transfusion associated lung injury (TRALI)<br>◯Other
■ if other: Please specify	________________

Concomitant medication

Centre ID	
Patient ID	
Visit	
Any new or changed concomitant medication since last visit	◯yes ◯no
If yes, please document all changes in Concomitant medications in the following section:	
Link to Concomitant medication	see table below

	Seq. no.	Drug Name	Indication	Start date	Ongoing at end of study	Stop date

Set Link / Create Entry ...

Concomitant COVID-19 therapy

Centre ID	
Patient ID	
Visit	
Any new Concomitant COVID-19 therapy since last visit?	◯yes ◯no
If yes, please document all changes in Concomitant COVID-19 therapy in the following section:	
Link to Concomitant COVID-19 therapy	

	Therapy number	Therapy	Total Daily Dose	Unit	Onset date of medication	Still ongoing	Stop date of therapy

Set Link / Create Entry ...

Adverse events

Centre ID	
Patient ID	
Visit	
Any new Adverse events since last visit?	○yes ○no
If yes, please document all new Adverse Events in the following section:	
Link to Adverse Events	

	AE number	SOC of AE (according CTCAE V5.0)	AE term (according CTCAE V5.0)	Start date	Ongoing at the end of study	Stop date	Is the AE serious?

Set Link / Create Entry ...

Day 2 - Cross-over Assessment

Centre ID	
Patient ID	
Visit	Day 2 - Cross-over Assessment
Date of Visit	____ ______ ________

- Vital signs
- Seven point ordinal scale
- Sequential Organ Failure Assessment (SOFA) Score
- Oxygen saturation (SaO2)
- Hematology
- Blood chemistry and coagulation
- SARS-CoV-2 viral clearance and load as well as antibody titres
- Administration of convalescent plasma
- Concomitant medication
- Concomitant COVID-19 therapy
- Adverse events

Investigator's Signature

Meaning:	I confirm the completeness and correctness of all documented data.
Signed By:	
Signature Date:	

Vital signs

Centre ID	
Patient ID	
Visit	
WHO performance status	◯ECOG 0 = Fully active, able to carry on all pre-disease performance without restriction<br>◯ECOG 1 = Restricted in physically strenuous activity but ambulatory and able to carry out work of a light or sedentary nature, e.g., light house work, office work<br>◯ECOG 2 = Ambulatory and capable of all selfcare but unable to carry out any work activities; up and about more than 50% of waking hours<br>◯ECOG 3 = Capable of only limited self-care, confined to bed or chair more than 50% of waking hours<br>◯ECOG 4 = Completely disabled. Cannot carry on any self-care. Totally confined to bed or chair.
Body temperature	____________________ °C
Type of measurement	◯oral ◯tympanic
Systolic blood pressure	____________________ mmHg
Diastolic blood pressure	____________________ mmHg
Pulse rate	____________________ beats/min
respiratory rate	____________________ breaths/min

Seven point ordinal scale

Centre ID	
Patient ID	
Visit	
Time of record	____ ____ ____
Ordinal Scale for Clinical Improvement	◯1 = not hospitalized with resumption of normal activities<br>◯2 = not hospitalized, but unable to resume normal sctivities<br>◯3 = hospitalized,not requiring supplemental oxygen<br>◯4 = hospitalized, requiring supplemental oxygen<br>◯5 = hospitalized, requiring nasal high-flow oxygen therapy or, noninvasive mechanical ventilation<br>◯6 = hospitalized, requiring ECMO, invasive mechanical ventilation, or both<br>◯7 = death

Sequential Organ Failure Assessment (SOFA) Score

Centre ID	
Patient ID	
Visit	
Respiratory system (PaO2/FiO2 (mmHg))	◯> 400<br>◯< 400<br>◯< 300<br>◯< 200 with respiratoy support<br>◯< 100 with respiratoy support
Nervous system (Glasgow Coma Scale)	◯15<br>◯13-14<br>◯10-12<br>◯6-9<br>◯<6
Cardiovascular system (Mean arterial pressure (MAP) or administration of vasopressor required)	◯MAP > 70 mmHg<br>◯MAP < 70 mmHg<br>◯Dopamine 5 µg/kg/min or dobutamine any dose<br>◯Dopamine > 5 µg/kg/min or epinephrine 0.1 µg/kg/min or norepinephrine 0.1 µg/kg/min<br>◯Dopamine > 15 µg/kg/min OR epinephrine > 0.1 µg/kg/min OR norepinephrine > 0.1 µg/kg/min
Liver (Bilirubin (mg/dl) [µmol/l])	◯< 1.2 [< 20]<br>◯1.2 - 1.9 [20 - 32]<br>◯2.0 - 5.9 [33 - 101]<br>◯6.0 - 11.9 [102 - 204]<br>◯> 12.0 [> 204]
Coagulation (Platelets x103/ml)	◯> 150<br>◯100-150<br>◯50-99<br>◯20-49<br>◯< 20
Kidneys (Creatinine (mg/dl) [µmol/L]; urine output)	◯< 1.2 [< 110]<br>◯1.2 - 1.9 [110 - 170]<br>◯2.0 - 3.4 [171 - 299]<br>◯3. - 4.9 [300 - 440]<br>◯> 5.0 [> 440]

Oxygen saturation (SaO2)

Centre ID	
Patient ID	
Visit	
ECMO	◯yes ◯no
Type of ventilation	◯no<br>◯Nasal high-flow oxygen therapy<br>◯Noninvasive mechanical ventilation<br>◯Invasive ventilation<br>◯Tracheotomy<br>◯Ambient air<br>◯Standard oxygen sources
Amount of oxygen	____________________ liter/min
Oxygen saturation (SaO2)	____________________ %
Amount of supplemental oxygen that is required to keep SaO2 above 94%	____________________ liter/min
Fraction of Inspired Oxygen (FiO2)	____________________ %
Partial pressure of oxygen (PaO2)	____________________ mmHg
Partial pressure of carbon dioxid (PaCO2)	____________________ mmHg

Hematology

Centre ID	
Patient ID	
Visit	
Date of sample taken	____ ______ ________

Hematology

Parameter	not done	Result	Unit
Hemoglobin	◯yes		▼
RBC	◯yes		▼
WBC	◯yes		▼
Thrombocytes	◯yes		▼

Blood chemistry and coagulation

Centre ID	
Patient ID	
Visit	
Date of sample taken	____ ______ ________

Clinical chemistry
<small>Please use a point '.' as decimal separator.</small>

Parameter	not done	Result	Unit
BUN	◯yes		
Creatinine	◯yes		
Albumin	◯yes		
AST/SGOT	◯yes		
ALT/SGPT	◯yes		
Total bilirubin	◯yes		
GGT	◯yes		
AP	◯yes		
LDH	◯yes		
Sodium	◯yes		
Potassium	◯yes		
Magnesium	◯yes		
Calcium	◯yes		
Uric acid	◯yes		
Troponin	◯yes		
CK	◯yes		U/l
CK-MB	◯yes		U/l
PTT	◯yes		sec
ATIII	◯yes		%
D-Dimer	◯yes		
Fibrinogen	◯yes		
Ferritin	◯yes		
Transferrin	◯yes		
Transferrin Saturation	◯yes		%
CRP	◯yes		
Total protein	◯yes		g/l
IL6	◯yes		pg/ml
Procalcitonin	◯yes		ng/ml
Total IgG	◯yes		
IgA	◯yes		
IgM	◯yes		
Lactate	◯yes		
INR	◯yes		

SARS-CoV-2 viral clearance and load as well as antibody titres

Centre ID	
Patient ID	
Visit	
Sample collection on Date of visit?	◯yes ◯no
Date of sample collection	____ ______ ________
Type of smear	◯Nasopharyngeal<br>◯Oropharyngeal<br>◯Sputum<br>◯Other
If other: Please specify	____________________
Result of smear	◯Negative ◯Positive ◯Invalide
PCR test type	◯TibMolbiol<br>◯Seegene<br>◯Abbott<br>◯Altona<br>◯Other
If other: Please specify	____________________
CT-value	____________________
Serodiagnostic by ELISA (OD Ratio)	____________________
Neutralize antibody titre (1:....)	____________________ ▼
Immunofluorescence	◯Positive<br>◯Borderline positive<br>◯Negative<br>◯Not performed

Administration of convalescent plasma

Centre ID	
Patient ID	
Visit	
Batch number of convalascent plasma	________
Time of infusion start	____ ____ ____
Time of infusion stop	____ ____ ____
Amount of infusion	____________________ ml
Transfusion reactions	◯No<br>◯Transfusion associated circulatory overload (TACO)<br>◯Citrate intoxication<br>◯Allergic reaction<br>◯Transfusion transmitted infection<br>◯Transfusion associated lung injury (TRALI)<br>◯Other
if other: Please specify	_______________
Treatment discontinuation	◯yes ◯no
Reason for treatment discontinution	◯Adverse event (other than allergic reaction) with the transfusion of CP<br>◯Serious Adverse Event upon CP transfusion<br>◯Allergic reaction
Continuation of CP treatment after AE resolution to smaller than grade 2 according to CTCAE	
■ Date of continuation	____ ______ ________
■ Time of infusion start	____ ____ ____
■ Time of infusion stop	____ ____ ____
■ Amount of infusion	____________________ ml
■ Transfusion reactions	◯No<br>◯Transfusion associated circulatory overload (TACO)<br>◯Citrate intoxication<br>◯Allergic reaction<br>◯Transfusion transmitted infection<br>◯Transfusion associated lung injury (TRALI)<br>◯Other
■ if other: Please specify	_______________

Concomitant medication

Centre ID	
Patient ID	
Visit	
Any new or changed concomitant medication since last visit	◯yes ◯no
If yes, please document all changes in Concomitant medications in the following section:	
Link to Concomitant medication	

	Seq. no.	Drug Name	Indicatio n	Start date	Ongoing at end of study	Stop date

Set Link / Create Entry ...

Concomitant COVID-19 therapy

Centre ID	
Patient ID	
Visit	
Any new Concomitant COVID-19 therapy since last visit?	○yes ○no
If yes, please document all changes in Concomitant COVID-19 therapy in the following section:	
Link to Concomitant COVID-19 therapy	

	Therapy number	Therapy	Total Daily Dose	Unit	Onset date of medication	Still ongoing	Stop date of therapy

Set Link / Create Entry ...

Adverse events

Centre ID	
Patient ID	
Visit	
Any new Adverse events since last visit?	◯yes ◯no
If yes, please document all new Adverse Events in the following section:	
Link to Adverse Events	(see table below)

	AE number	SOC of AE (according CTCAE V5.0)	AE term (according CTCAE V5.0)	Start date	Ongoing at the end of study	Stop date	Is the AE serious?

Set Link / Create Entry ...

Day 3 - Cross-over Assessment

Centre ID	
Patient ID	
Visit	Day 3 - Cross-over Assessment
Date of Visit	____ ______ ________
Hospitalization?	◯yes ◯no

- Vital signs
- Seven point ordinal scale
- Sequential Organ Failure Assessment (SOFA) Score
- Oxygen saturation (SaO2)
- Hematology
- Blood chemistry and coagulation
- SARS-CoV-2 viral clearance and load as well as antibody titres
- Concomitant medication
- Concomitant COVID-19 therapy
- Adverse events

Investigator's Signature

Meaning:	I confirm the completeness and correctness of all documented data.
Signed By:	
Signature Date:	

Vital signs

Centre ID	
Patient ID	
Visit	
WHO performance status	◯ECOG 0 = Fully active, able to carry on all pre-disease performance without restriction<br>◯ECOG 1 = Restricted in physically strenuous activity but ambulatory and able to carry out work of a light or sedentary nature, e.g., light house work, office work<br>◯ECOG 2 = Ambulatory and capable of all selfcare but unable to carry out any work activities; up and about more than 50% of waking hours<br>◯ECOG 3 = Capable of only limited self-care, confined to bed or chair more than 50% of waking hours<br>◯ECOG 4 = Completely disabled. Cannot carry on any self-care. Totally confined to bed or chair.
Body temperature	____________________ °C
Type of measurement	◯oral ◯tympanic
Systolic blood pressure	____________________ mmHg
Diastolic blood pressure	____________________ mmHg
Pulse rate	____________________ beats/min
respiratory rate	____________________ breaths/min

Seven point ordinal scale

Centre ID	
Patient ID	
Visit	
Time of record	____ ____ ____
Ordinal Scale for Clinical Improvement	◯1 = not hospitalized with resumption of normal activities<br>◯2 = not hospitalized, but unable to resume normal sctivities<br>◯3 = hospitalized,not requiring supplemental oxygen<br>◯4 = hospitalized, requiring supplemental oxygen<br>◯5 = hospitalized, requiring nasal high-flow oxygen therapy or, noninvasive mechanical ventilation<br>◯6 = hospitalized, requiring ECMO, invasive mechanical ventilation, or both<br>◯7 = death

Sequential Organ Failure Assessment (SOFA) Score

Centre ID	
Patient ID	
Visit	
Respiratory system (PaO2/FiO2 (mmHg))	◯> 400<br>◯< 400<br>◯< 300<br>◯< 200 with respiratoy support<br>◯< 100 with respiratoy support
Nervous system (Glasgow Coma Scale)	◯15<br>◯13-14<br>◯10-12<br>◯6-9<br>◯<6
Cardiovascular system (Mean arterial pressure (MAP) or administration of vasopressor required)	◯MAP > 70 mmHg<br>◯MAP < 70 mmHg<br>◯Dopamine 5 µg/kg/min or dobutamine any dose<br>◯Dopamine > 5 µg/kg/min or epinephrine 0.1 µg/kg/min or norepinephrine 0.1 µg/kg/min<br>◯Dopamine > 15 µg/kg/min OR epinephrine > 0.1 µg/kg/min OR norepinephrine > 0.1 µg/kg/min
Liver (Bilirubin (mg/dl) [µmol/l])	◯< 1.2 [< 20]<br>◯1.2 - 1.9 [20 - 32]<br>◯2.0 - 5.9 [33 - 101]<br>◯6.0 - 11.9 [102 - 204]<br>◯> 12.0 [> 204]
Coagulation (Platelets x103/ml)	◯> 150<br>◯100-150<br>◯50-99<br>◯20-49<br>◯< 20
Kidneys (Creatinine (mg/dl) [µmol/L]; urine output)	◯< 1.2 [< 110]<br>◯1.2 - 1.9 [110 - 170]<br>◯2.0 - 3.4 [171 - 299]<br>◯3. - 4.9 [300 - 440]<br>◯> 5.0 [> 440]

Oxygen saturation (SaO2)

Centre ID	
Patient ID	
Visit	
ECMO	◯yes ◯no
Type of ventilation	◯no<br>◯Nasal high-flow oxygen therapy<br>◯Noninvasive mechanical ventilation<br>◯Invasive ventilation<br>◯Tracheotomy<br>◯Ambient air<br>◯Standard oxygen sources
Amount of oxygen	____________________ liter/min
Oxygen saturation (SaO2)	____________________ %
Amount of supplemental oxygen that is required to keep SaO2 above 94%	____________________ liter/min
Fraction of Inspired Oxygen (FiO2)	____________________ %
Partial pressure of oxygen (PaO2)	____________________ mmHg
Partial pressure of carbon dioxid (PaCO2)	____________________ mmHg

Hematology

Centre ID	
Patient ID	
Visit	
Date of sample taken	____ _____ _______

Hematology

Parameter	not done	Result	Unit
Hemoglobin	◯yes		
RBC	◯yes		
WBC	◯yes		
Thrombocytes	◯yes		

Blood chemistry and coagulation

Centre ID	
Patient ID	
Visit	
Date of sample taken	____ ______ ________

Clinical chemistry
<small>Please use a point '.' as decimal separator.</small>

Parameter	not done	Result	Unit
BUN	yes		
Creatinine	yes		
Albumin	yes		
AST/SGOT	yes		
ALT/SGPT	yes		
Total bilirubin	yes		
GGT	yes		
AP	yes		
LDH	yes		
Sodium	yes		
Potassium	yes		
Magnesium	yes		
Calcium	yes		
Uric acid	yes		
Troponin	yes		
CK	yes		U/l
CK-MB	yes		U/l
PTT	yes		sec
ATIII	yes		%
D-Dimer	yes		
Fibrinogen	yes		
Ferritin	yes		
Transferrin	yes		
Transferrin Saturation	yes		%
CRP	yes		
Total protein	yes		g/l
IL6	yes		pg/ml
Procalcitonin	yes		ng/ml
Total IgG	yes		
IgA	yes		
IgM	yes		
Lactate	yes		
INR	yes		

SARS-CoV-2 viral clearance and load as well as antibody titres

Centre ID	
Patient ID	
Visit	
Sample collection on Date of visit?	◯yes ◯no
Date of sample collection	____ ______ ________
Type of smear	◯Nasopharyngeal<br>◯Oropharyngeal<br>◯Sputum<br>◯Other
If other: Please specify	____________________
Result of smear	◯Negative ◯Positive ◯Invalide
PCR test type	◯TibMolbiol<br>◯Seegene<br>◯Abbott<br>◯Altona<br>◯Other
If other: Please specify	____________________
CT-value	____________________
Serodiagnostic by ELISA (OD Ratio)	____________________
Neutralize antibody titre (1:....)	____________________ ▼
Immunofluorescence	◯Positive<br>◯Borderline positive<br>◯Negative<br>◯Not performed

Concomitant medication

Centre ID

Patient ID

Visit

Any new or changed concomitant medication since last visit ◯yes ◯no

If yes, please document all changes in Concomitant medications in the following section:

Link to Concomitant medication

	Seq. no.	Drug Name	Indicatio n	Start date	Ongoing at end of study	Stop date

Set Link / Create Entry ...

Concomitant COVID-19 therapy

Centre ID	
Patient ID	
Visit	
Any new Concomitant COVID-19 therapy since last visit?	◯yes ◯no
If yes, please document all changes in Concomitant COVID-19 therapy in the following section:	
Link to Concomitant COVID-19 therapy	see table below

	Therapy number	Therapy	Total Daily Dose	Unit	Onset date of medication	Still ongoing	Stop date of therapy

Set Link / Create Entry ...

Adverse events

Centre ID	
Patient ID	
Visit	
Any new Adverse events since last visit?	◯yes ◯no

If yes, please document all new Adverse Events in the following section:

Link to Adverse Events

	AE number	SOC of AE (according CTCAE V5.0)	AE term (according CTCAE V5.0)	Start date	Ongoing at the end of study	Stop date	Is the AE serious?

Set Link / Create Entry ...

Day 4 - Cross-over Assessment

Centre ID	
Patient ID	
Visit	Day 4 - Cross-over Assessment
Date of Visit	____ ______ ________
Hospitalization?	◯yes ◯no

- Vital signs
- Seven point ordinal scale
- Sequential Organ Failure Assessment (SOFA) Score
- Oxygen saturation (SaO2)
- Hematology
- Blood chemistry and coagulation
- Concomitant medication
- Concomitant COVID-19 therapy
- Adverse events

Investigator's Signature

Meaning:	I confirm the completeness and correctness of all documented data.
Signed By:	
Signature Date:	

Vital signs

Centre ID	
Patient ID	
Visit	
WHO performance status	◯ECOG 0 = Fully active, able to carry on all pre-disease performance without restriction<br>◯ECOG 1 = Restricted in physically strenuous activity but ambulatory and able to carry out work of a light or sedentary nature, e.g., light house work, office work<br>◯ECOG 2 = Ambulatory and capable of all selfcare but unable to carry out any work activities; up and about more than 50% of waking hours<br>◯ECOG 3 = Capable of only limited self-care, confined to bed or chair more than 50% of waking hours<br>◯ECOG 4 = Completely disabled. Cannot carry on any self-care. Totally confined to bed or chair.
Body temperature	____________________ °C
Type of measurement	◯oral ◯tympanic
Systolic blood pressure	____________________ mmHg
Diastolic blood pressure	____________________ mmHg
Pulse rate	____________________ beats/min
respiratory rate	____________________ breaths/min

Seven point ordinal scale

Centre ID	
Patient ID	
Visit	
Time of record	____ ____ ____
Ordinal Scale for Clinical Improvement	◯1 = not hospitalized with resumption of normal activities<br>◯2 = not hospitalized, but unable to resume normal sctivities<br>◯3 = hospitalized,not requiring supplemental oxygen<br>◯4 = hospitalized, requiring supplemental oxygen<br>◯5 = hospitalized, requiring nasal high-flow oxygen therapy or, noninvasive mechanical ventilation<br>◯6 = hospitalized, requiring ECMO, invasive mechanical ventilation, or both<br>◯7 = death

Sequential Organ Failure Assessment (SOFA) Score

Centre ID	
Patient ID	
Visit	
Respiratory system (PaO2/FiO2 (mmHg))	> 400<br>< 400<br>< 300<br>< 200 with respiratoy support<br>< 100 with respiratoy support
Nervous system (Glasgow Coma Scale)	15<br>13-14<br>10-12<br>6-9<br><6
Cardiovascular system (Mean arterial pressure (MAP) or administration of vasopressor required)	MAP > 70 mmHg<br>MAP < 70 mmHg<br>Dopamine 5 µg/kg/min or dobutamine any dose<br>Dopamine > 5 µg/kg/min or epinephrine 0.1 µg/kg/min or norepinephrine 0.1 µg/kg/min<br>Dopamine > 15 µg/kg/min OR epinephrine > 0.1 µg/kg/min OR norepinephrine > 0.1 µg/kg/min
Liver (Bilirubin (mg/dl) [µmol/l])	< 1.2 [< 20]<br>1.2 - 1.9 [20 - 32]<br>2.0 - 5.9 [33 - 101]<br>6.0 - 11.9 [102 - 204]<br>> 12.0 [> 204]
Coagulation (Platelets x103/ml)	> 150<br>100-150<br>50-99<br>20-49<br>< 20
Kidneys (Creatinine (mg/dl) [µmol/L]; urine output)	< 1.2 [< 110]<br>1.2 - 1.9 [110 - 170]<br>2.0 - 3.4 [171 - 299]<br>3. - 4.9 [300 - 440]<br>> 5.0 [> 440]

Oxygen saturation (SaO2)

Centre ID	
Patient ID	
Visit	
ECMO	◯yes ◯no
Type of ventilation	◯no<br>◯Nasal high-flow oxygen therapy<br>◯Noninvasive mechanical ventilation<br>◯Invasive ventilation<br>◯Tracheotomy<br>◯Ambient air<br>◯Standard oxygen sources
Amount of oxygen	____________________ liter/min
Oxygen saturation (SaO2)	____________________ %
Amount of supplemental oxygen that is required to keep SaO2 above 94%	____________________ liter/min
Fraction of Inspired Oxygen (FiO2)	____________________ %
Partial pressure of oxygen (PaO2)	____________________ mmHg
Partial pressure of carbon dioxid (PaCO2)	____________________ mmHg

Hematology

Centre ID	
Patient ID	
Visit	
Date of sample taken	____ ______ ________

Hematology

Parameter	not done	Result	Unit
Hemoglobin	◯yes		▾
RBC	◯yes		▾
WBC	◯yes		▾
Thrombocytes	◯yes		▾

Blood chemistry and coagulation

Centre ID	
Patient ID	
Visit	
Date of sample taken	____ _____ _______

Clinical chemistry
<small>Please use a point '.' as decimal separator.</small>

Parameter	not done	Result	Unit
BUN	yes		
Creatinine	yes		
Albumin	yes		
AST/SGOT	yes		
ALT/SGPT	yes		
Total bilirubin	yes		
GGT	yes		
AP	yes		
LDH	yes		
Sodium	yes		
Potassium	yes		
Magnesium	yes		
Calcium	yes		
Uric acid	yes		
Troponin	yes		
CK	yes		U/l
CK-MB	yes		U/l
PTT	yes		sec
ATIII	yes		%
D-Dimer	yes		
Fibrinogen	yes		
Ferritin	yes		
Transferrin	yes		
Transferrin Saturation	yes		%
CRP	yes		
Total protein	yes		g/l
IL6	yes		pg/ml
Procalcitonin	yes		ng/ml
Total IgG	yes		
IgA	yes		
IgM	yes		
Lactate	yes		
INR	yes		

Concomitant medication

Centre ID	
Patient ID	
Visit	
Any new or changed concomitant medication since last visit	◯yes ◯no
If yes, please document all changes in Concomitant medications in the following section:	
Link to Concomitant medication	

	Seq. no.	Drug Name	Indication	Start date	Ongoing at end of study	Stop date

Set Link / Create Entry ...

Concomitant COVID-19 therapy

Centre ID	
Patient ID	
Visit	
Any new Concomitant COVID-19 therapy since last visit?	◯yes ◯no

If yes, please document all changes in Concomitant COVID-19 therapy in the following section:

Link to Concomitant COVID-19 therapy

	Therapy number	Therapy	Total Daily Dose	Unit	Onset date of medication	Still ongoing	Stop date of therapy

Set Link / Create Entry ...

Adverse events

Centre ID	
Patient ID	
Visit	
Any new Adverse events since last visit?	◯yes ◯no
If yes, please document all new Adverse Events in the following section:	
Link to Adverse Events	

	AE number	SOC of AE (according CTCAE V5.0)	AE term (according CTCAE V5.0)	Start date	Ongoing at the end of study	Stop date	Is the AE serious?

Set Link / Create Entry ...

Day 5 - Cross-over Assessment

Centre ID	
Patient ID	
Visit	Day 5 - Cross-over Assessment
Date of Visit	____ ______ ________
Hospitalization?	◯yes ◯no

- Vital signs
- Seven point ordinal scale
- Sequential Organ Failure Assessment (SOFA) Score
- Oxygen saturation (SaO2)
- Hematology
- Blood chemistry and coagulation
- SARS-CoV-2 viral clearance and load as well as antibody titres
- Concomitant medication
- Concomitant COVID-19 therapy
- Adverse events

Investigator's Signature

Meaning:	I confirm the completeness and correctness of all documented data.
Signed By:	
Signature Date:	

Vital signs

Centre ID	
Patient ID	
Visit	
WHO performance status	◯ECOG 0 = Fully active, able to carry on all pre-disease performance without restriction<br>◯ECOG 1 = Restricted in physically strenuous activity but ambulatory and able to carry out work of a light or sedentary nature, e.g., light house work, office work<br>◯ECOG 2 = Ambulatory and capable of all selfcare but unable to carry out any work activities; up and about more than 50% of waking hours<br>◯ECOG 3 = Capable of only limited self-care, confined to bed or chair more than 50% of waking hours<br>◯ECOG 4 = Completely disabled. Cannot carry on any self-care. Totally confined to bed or chair.
Body temperature	____________________ °C
Type of measurement	◯oral ◯tympanic
Systolic blood pressure	____________________ mmHg
Diastolic blood pressure	____________________ mmHg
Pulse rate	____________________ beats/min
respiratory rate	____________________ breaths/min

Seven point ordinal scale

Centre ID	
Patient ID	
Visit	
Time of record	____ ____ ____
Ordinal Scale for Clinical Improvement	◯1 = not hospitalized with resumption of normal activities<br>◯2 = not hospitalized, but unable to resume normal sctivities<br>◯3 = hospitalized,not requiring supplemental oxygen<br>◯4 = hospitalized, requiring supplemental oxygen<br>◯5 = hospitalized, requiring nasal high-flow oxygen therapy or, noninvasive mechanical ventilation<br>◯6 = hospitalized, requiring ECMO, invasive mechanical ventilation, or both<br>◯7 = death

Sequential Organ Failure Assessment (SOFA) Score

Centre ID	
Patient ID	
Visit	
Respiratory system (PaO2/FiO2 (mmHg))	◯> 400<br>◯< 400<br>◯< 300<br>◯< 200 with respiratoy support<br>◯< 100 with respiratoy support
Nervous system (Glasgow Coma Scale)	◯15<br>◯13-14<br>◯10-12<br>◯6-9<br>◯<6
Cardiovascular system (Mean arterial pressure (MAP) or administration of vasopressor required)	◯MAP > 70 mmHg<br>◯MAP < 70 mmHg<br>◯Dopamine 5 µg/kg/min or dobutamine any dose<br>◯Dopamine > 5 µg/kg/min or epinephrine 0.1 µg/kg/min or norepinephrine 0.1 µg/kg/min<br>◯Dopamine > 15 µg/kg/min OR epinephrine > 0.1 µg/kg/min OR norepinephrine > 0.1 µg/kg/min
Liver (Bilirubin (mg/dl) [µmol/l])	◯< 1.2 [< 20]<br>◯1.2 - 1.9 [20 - 32]<br>◯2.0 - 5.9 [33 - 101]<br>◯6.0 - 11.9 [102 - 204]<br>◯> 12.0 [> 204]
Coagulation (Platelets x103/ml)	◯> 150<br>◯100-150<br>◯50-99<br>◯20-49<br>◯< 20
Kidneys (Creatinine (mg/dl) [µmol/L]; urine output)	◯< 1.2 [< 110]<br>◯1.2 - 1.9 [110 - 170]<br>◯2.0 - 3.4 [171 - 299]<br>◯3. - 4.9 [300 - 440]<br>◯> 5.0 [> 440]

Oxygen saturation (SaO2)

Centre ID	
Patient ID	
Visit	
ECMO	◯yes ◯no
Type of ventilation	◯no<br>◯Nasal high-flow oxygen therapy<br>◯Noninvasive mechanical ventilation<br>◯Invasive ventilation<br>◯Tracheotomy<br>◯Ambient air<br>◯Standard oxygen sources
Amount of oxygen	____________________ liter/min
Oxygen saturation (SaO2)	____________________ %
Amount of supplemental oxygen that is required to keep SaO2 above 94%	____________________ liter/min
Fraction of Inspired Oxygen (FiO2)	____________________ %
Partial pressure of oxygen (PaO2)	____________________ mmHg
Partial pressure of carbon dioxid (PaCO2)	____________________ mmHg

Hematology

Centre ID	
Patient ID	
Visit	
Date of sample taken	____ ______ ________

Hematology

Parameter	not done	Result	Unit
Hemoglobin	◯yes		
RBC	◯yes		
WBC	◯yes		
Thrombocytes	◯yes		

Blood chemistry and coagulation

Centre ID	
Patient ID	
Visit	
Date of sample taken	____ ______ ________

Clinical chemistry
<small>Please use a point '.' as decimal separator.</small>

Parameter	not done	Result	Unit
BUN	yes		
Creatinine	yes		
Albumin	yes		
AST/SGOT	yes		
ALT/SGPT	yes		
Total bilirubin	yes		
GGT	yes		
AP	yes		
LDH	yes		
Sodium	yes		
Potassium	yes		
Magnesium	yes		
Calcium	yes		
Uric acid	yes		
Troponin	yes		
CK	yes		U/l
CK-MB	yes		U/l
PTT	yes		sec
ATIII	yes		%
D-Dimer	yes		
Fibrinogen	yes		
Ferritin	yes		
Transferrin	yes		
Transferrin Saturation	yes		%
CRP	yes		
Total protein	yes		g/l
IL6	yes		pg/ml
Procalcitonin	yes		ng/ml
Total IgG	yes		
IgA	yes		
IgM	yes		
Lactate	yes		
INR	yes		

SARS-CoV-2 viral clearance and load as well as antibody titres

Centre ID	
Patient ID	
Visit	
Sample collection on Date of visit?	◯yes ◯no
Date of sample collection	____ ______ ________
Type of smear	◯Nasopharyngeal<br>◯Oropharyngeal<br>◯Sputum<br>◯Other
If other: Please specify	____________________
Result of smear	◯Negative ◯Positive ◯Invalide
PCR test type	◯TibMolbiol<br>◯Seegene<br>◯Abbott<br>◯Altona<br>◯Other
If other: Please specify	____________________
CT-value	____________________
Serodiagnostic by ELISA (OD Ratio)	____________________
Neutralize antibody titre (1:....)	____________________ ▼
Immunofluorescence	◯Positive<br>◯Borderline positive<br>◯Negative<br>◯Not performed

Concomitant medication

Centre ID	
Patient ID	
Visit	
Any new or changed concomitant medication since last visit	◯yes ◯no
If yes, please document all changes in Concomitant medications in the following section:	
Link to Concomitant medication	

	Seq. no.	Drug Name	Indicatio n	Start date	Ongoing at end of study	Stop date

Set Link / Create Entry ...

Concomitant COVID-19 therapy

Centre ID	
Patient ID	
Visit	
Any new Concomitant COVID-19 therapy since last visit?	◯yes ◯no

If yes, please document all changes in Concomitant COVID-19 therapy in the following section:

Link to Concomitant COVID-19 therapy

	Therapy number	Therapy	Total Daily Dose	Unit	Onset date of medication	Still ongoing	Stop date of therapy

Set Link / Create Entry ...

Adverse events

Centre ID	
Patient ID	
Visit	
Any new Adverse events since last visit?	◯yes ◯no
If yes, please document all new Adverse Events in the following section:	
Link to Adverse Events	(see table below)

	AE number	SOC of AE (according CTCAE V5.0)	AE term (according CTCAE V5.0)	Start date	Ongoing at the end of study	Stop date	Is the AE serious?

Set Link / Create Entry ...

Day 6 - Cross-over Assessment

Centre ID	
Patient ID	
Visit	Day 6 - Cross-over Assessment
Date of Visit	____ ______ ________
Hospitalization?	◯yes ◯no

- Vital signs
- Seven point ordinal scale
- Sequential Organ Failure Assessment (SOFA) Score
- Oxygen saturation (SaO2)
- Hematology
- Blood chemistry and coagulation
- Concomitant medication
- Concomitant COVID-19 therapy
- Adverse events

Investigator's Signature

Meaning:	I confirm the completeness and correctness of all documented data.
Signed By:	
Signature Date:	

Vital signs

Centre ID	
Patient ID	
Visit	
WHO performance status	◯ECOG 0 = Fully active, able to carry on all pre-disease performance without restriction<br>◯ECOG 1 = Restricted in physically strenuous activity but ambulatory and able to carry out work of a light or sedentary nature, e.g., light house work, office work<br>◯ECOG 2 = Ambulatory and capable of all selfcare but unable to carry out any work activities; up and about more than 50% of waking hours<br>◯ECOG 3 = Capable of only limited self-care, confined to bed or chair more than 50% of waking hours<br>◯ECOG 4 = Completely disabled. Cannot carry on any self-care. Totally confined to bed or chair.
Body temperature	____________________ °C
Type of measurement	◯oral ◯tympanic
Systolic blood pressure	____________________ mmHg
Diastolic blood pressure	____________________ mmHg
Pulse rate	____________________ beats/min
respiratory rate	____________________ breaths/min

Seven point ordinal scale

Centre ID	
Patient ID	
Visit	
Time of record	____ ____ ____
Ordinal Scale for Clinical Improvement	◯1 = not hospitalized with resumption of normal activities<br>◯2 = not hospitalized, but unable to resume normal sctivities<br>◯3 = hospitalized,not requiring supplemental oxygen<br>◯4 = hospitalized, requiring supplemental oxygen<br>◯5 = hospitalized, requiring nasal high-flow oxygen therapy or, noninvasive mechanical ventilation<br>◯6 = hospitalized, requiring ECMO, invasive mechanical ventilation, or both<br>◯7 = death

Sequential Organ Failure Assessment (SOFA) Score

Centre ID	
Patient ID	
Visit	
Respiratory system (PaO2/FiO2 (mmHg))	◯> 400<br>◯< 400<br>◯< 300<br>◯< 200 with respiratoy support<br>◯< 100 with respiratoy support
Nervous system (Glasgow Coma Scale)	◯15<br>◯13-14<br>◯10-12<br>◯6-9<br>◯<6
Cardiovascular system (Mean arterial pressure (MAP) or administration of vasopressor required)	◯MAP > 70 mmHg<br>◯MAP < 70 mmHg<br>◯Dopamine 5 µg/kg/min or dobutamine any dose<br>◯Dopamine > 5 µg/kg/min or epinephrine 0.1 µg/kg/min or norepinephrine 0.1 µg/kg/min<br>◯Dopamine > 15 µg/kg/min OR epinephrine > 0.1 µg/kg/min OR norepinephrine > 0.1 µg/kg/min
Liver (Bilirubin (mg/dl) [µmol/l])	◯< 1.2 [< 20]<br>◯1.2 - 1.9 [20 - 32]<br>◯2.0 - 5.9 [33 - 101]<br>◯6.0 - 11.9 [102 - 204]<br>◯> 12.0 [> 204]
Coagulation (Platelets x103/ml)	◯> 150<br>◯100-150<br>◯50-99<br>◯20-49<br>◯< 20
Kidneys (Creatinine (mg/dl) [µmol/L]; urine output)	◯< 1.2 [< 110]<br>◯1.2 - 1.9 [110 - 170]<br>◯2.0 - 3.4 [171 - 299]<br>◯3. - 4.9 [300 - 440]<br>◯> 5.0 [> 440]

Oxygen saturation (SaO2)

Centre ID	
Patient ID	
Visit	
ECMO	◯yes ◯no
Type of ventilation	◯no<br>◯Nasal high-flow oxygen therapy<br>◯Noninvasive mechanical ventilation<br>◯Invasive ventilation<br>◯Tracheotomy<br>◯Ambient air<br>◯Standard oxygen sources
Amount of oxygen	____________________ liter/min
Oxygen saturation (SaO2)	____________________ %
Amount of supplemental oxygen that is required to keep SaO2 above 94%	____________________ liter/min
Fraction of Inspired Oxygen (FiO2)	____________________ %
Partial pressure of oxygen (PaO2)	____________________ mmHg
Partial pressure of carbon dioxid (PaCO2)	____________________ mmHg

Hematology

Centre ID	
Patient ID	
Visit	
Date of sample taken	____ ______ ________

Hematology

Parameter	not done	Result	Unit
Hemoglobin	◯yes		▾
RBC	◯yes		▾
WBC	◯yes		▾
Thrombocytes	◯yes		▾

Blood chemistry and coagulation

Centre ID	
Patient ID	
Visit	
Date of sample taken	____ ______ ________

Clinical chemistry
<small>Please use a point '.' as decimal separator.</small>

Parameter	not done	Result	Unit
BUN	◯yes		
Creatinine	◯yes		
Albumin	◯yes		
AST/SGOT	◯yes		
ALT/SGPT	◯yes		
Total bilirubin	◯yes		
GGT	◯yes		
AP	◯yes		
LDH	◯yes		
Sodium	◯yes		
Potassium	◯yes		
Magnesium	◯yes		
Calcium	◯yes		
Uric acid	◯yes		
Troponin	◯yes		
CK	◯yes		U/l
CK-MB	◯yes		U/l
PTT	◯yes		sec
ATIII	◯yes		%
D-Dimer	◯yes		
Fibrinogen	◯yes		
Ferritin	◯yes		
Transferrin	◯yes		
Transferrin Saturation	◯yes		%
CRP	◯yes		
Total protein	◯yes		g/l
IL6	◯yes		pg/ml
Procalcitonin	◯yes		ng/ml
Total IgG	◯yes		
IgA	◯yes		
IgM	◯yes		
Lactate	◯yes		
INR	◯yes		

Concomitant medication

Centre ID	
Patient ID	
Visit	
Any new or changed concomitant medication since last visit	◯yes ◯no
If yes, please document all changes in Concomitant medications in the following section:	
Link to Concomitant medication	

	Seq. no.	Drug Name	Indicatio n	Start date	Ongoing at end of study	Stop date

Set Link / Create Entry ...

Concomitant COVID-19 therapy

Centre ID	
Patient ID	
Visit	
Any new Concomitant COVID-19 therapy since last visit?	◯yes ◯no
If yes, please document all changes in Concomitant COVID-19 therapy in the following section:	
Link to Concomitant COVID-19 therapy	

	Therapy number	Therapy	Total Daily Dose	Unit	Onset date of medication	Still ongoing	Stop date of therapy

Set Link / Create Entry ...

Adverse events

Centre ID	
Patient ID	
Visit	
Any new Adverse events since last visit?	◯yes ◯no
If yes, please document all new Adverse Events in the following section:	
Link to Adverse Events	see table below

	AE number	SOC of AE (according CTCAE V5.0)	AE term (according CTCAE V5.0)	Start date	Ongoing at the end of study	Stop date	Is the AE serious?

Set Link / Create Entry ...

Day 7 - Cross-over Assessment

Centre ID	
Patient ID	
Visit	Day 7 - Cross-over Assessment
Date of Visit	____ ______ ________
Hospitalization?	◯yes ◯no

- Vital signs
- Seven point ordinal scale
- Sequential Organ Failure Assessment (SOFA) Score
- Oxygen saturation (SaO2)
- Hematology
- Blood chemistry and coagulation
- SARS-CoV-2 viral clearance and load as well as antibody titres
- Concomitant medication
- Concomitant COVID-19 therapy
- Adverse events

Investigator's Signature

Meaning:	I confirm the completeness and correctness of all documented data.
Signed By:	
Signature Date:	

Vital signs

Centre ID	
Patient ID	
Visit	
WHO performance status	◯ECOG 0 = Fully active, able to carry on all pre-disease performance without restriction<br>◯ECOG 1 = Restricted in physically strenuous activity but ambulatory and able to carry out work of a light or sedentary nature, e.g., light house work, office work<br>◯ECOG 2 = Ambulatory and capable of all selfcare but unable to carry out any work activities; up and about more than 50% of waking hours<br>◯ECOG 3 = Capable of only limited self-care, confined to bed or chair more than 50% of waking hours<br>◯ECOG 4 = Completely disabled. Cannot carry on any self-care. Totally confined to bed or chair.
Body temperature	____________________ °C
Type of measurement	◯oral ◯tympanic
Systolic blood pressure	____________________ mmHg
Diastolic blood pressure	____________________ mmHg
Pulse rate	____________________ beats/min
respiratory rate	____________________ breaths/min

Seven point ordinal scale

Centre ID	
Patient ID	
Visit	
Time of record	____ ____ ____
Ordinal Scale for Clinical Improvement	◯1 = not hospitalized with resumption of normal activities<br>◯2 = not hospitalized, but unable to resume normal sctivities<br>◯3 = hospitalized,not requiring supplemental oxygen<br>◯4 = hospitalized, requiring supplemental oxygen<br>◯5 = hospitalized, requiring nasal high-flow oxygen therapy or, noninvasive mechanical ventilation<br>◯6 = hospitalized, requiring ECMO, invasive mechanical ventilation, or both<br>◯7 = death

Sequential Organ Failure Assessment (SOFA) Score

Centre ID	
Patient ID	
Visit	
Respiratory system (PaO2/FiO2 (mmHg))	> 400<br>< 400<br>< 300<br>< 200 with respiratoy support<br>< 100 with respiratoy support
Nervous system (Glasgow Coma Scale)	15<br>13-14<br>10-12<br>6-9<br><6
Cardiovascular system (Mean arterial pressure (MAP) or administration of vasopressor required)	MAP > 70 mmHg<br>MAP < 70 mmHg<br>Dopamine 5 µg/kg/min or dobutamine any dose<br>Dopamine > 5 µg/kg/min or epinephrine 0.1 µg/kg/min or norepinephrine 0.1 µg/kg/min<br>Dopamine > 15 µg/kg/min OR epinephrine > 0.1 µg/kg/min OR norepinephrine > 0.1 µg/kg/min
Liver (Bilirubin (mg/dl) [µmol/l])	< 1.2 [< 20]<br>1.2 - 1.9 [20 - 32]<br>2.0 - 5.9 [33 - 101]<br>6.0 - 11.9 [102 - 204]<br>> 12.0 [> 204]
Coagulation (Platelets x103/ml)	> 150<br>100-150<br>50-99<br>20-49<br>< 20
Kidneys (Creatinine (mg/dl) [µmol/L]; urine output)	< 1.2 [< 110]<br>1.2 - 1.9 [110 - 170]<br>2.0 - 3.4 [171 - 299]<br>3. - 4.9 [300 - 440]<br>> 5.0 [> 440]

Oxygen saturation (SaO2)

Centre ID	
Patient ID	
Visit	
ECMO	◯yes ◯no
Type of ventilation	◯no<br>◯Nasal high-flow oxygen therapy<br>◯Noninvasive mechanical ventilation<br>◯Invasive ventilation<br>◯Tracheotomy<br>◯Ambient air<br>◯Standard oxygen sources
Amount of oxygen	____________________ liter/min
Oxygen saturation (SaO2)	____________________ %
Amount of supplemental oxygen that is required to keep SaO2 above 94%	____________________ liter/min
Fraction of Inspired Oxygen (FiO2)	____________________ %
Partial pressure of oxygen (PaO2)	____________________ mmHg
Partial pressure of carbon dioxid (PaCO2)	____________________ mmHg

Hematology

Centre ID	
Patient ID	
Visit	
Date of sample taken	____ ______ ________

Hematology

Parameter	not done	Result	Unit
Hemoglobin	yes		
RBC	yes		
WBC	yes		
Thrombocytes	yes		

Blood chemistry and coagulation

Centre ID	
Patient ID	
Visit	
Date of sample taken	____ ______ ________

Clinical chemistry

<small>Please use a point '.' as decimal separator.</small>

Parameter	not done	Result	Unit
BUN	yes		
Creatinine	yes		
Albumin	yes		
AST/SGOT	yes		
ALT/SGPT	yes		
Total bilirubin	yes		
GGT	yes		
AP	yes		
LDH	yes		
Sodium	yes		
Potassium	yes		
Magnesium	yes		
Calcium	yes		
Uric acid	yes		
Troponin	yes		
CK	yes		U/l
CK-MB	yes		U/l
PTT	yes		sec
ATIII	yes		%
D-Dimer	yes		
Fibrinogen	yes		
Ferritin	yes		
Transferrin	yes		
Transferrin Saturation	yes		%
CRP	yes		
Total protein	yes		g/l
IL6	yes		pg/ml
Procalcitonin	yes		ng/ml
Total IgG	yes		
IgA	yes		
IgM	yes		
Lactate	yes		
INR	yes		

SARS-CoV-2 viral clearance and load as well as antibody titres

Centre ID	
Patient ID	
Visit	
Sample collection on Date of visit?	◯yes ◯no
Date of sample collection	____ ______ ________
Type of smear	◯Nasopharyngeal<br>◯Oropharyngeal<br>◯Sputum<br>◯Other
If other: Please specify	____________________
Result of smear	◯Negative ◯Positive ◯Invalide
PCR test type	◯TibMolbiol<br>◯Seegene<br>◯Abbott<br>◯Altona<br>◯Other
If other: Please specify	____________________
CT-value	____________________
Serodiagnostic by ELISA (OD Ratio)	____________________
Neutralize antibody titre (1:....)	____________________ ▾
Immunofluorescence	◯Positive<br>◯Borderline positive<br>◯Negative<br>◯Not performed

Concomitant medication

Centre ID	
Patient ID	
Visit	
Any new or changed concomitant medication since last visit	◯yes ◯no
If yes, please document all changes in Concomitant medications in the following section:	
Link to Concomitant medication	

	Seq. no.	Drug Name	Indicatio n	Start date	Ongoing at end of study	Stop date

Set Link / Create Entry ...

Concomitant COVID-19 therapy

Centre ID

Patient ID

Visit

Any new Concomitant COVID-19 therapy since last visit? ○yes ○no

If yes, please document all changes in Concomitant COVID-19 therapy in the following section:

Link to Concomitant COVID-19 therapy

	Therapy number	Therapy	Total Daily Dose	Unit	Onset date of medication	Still ongoing	Stop date of therapy

Set Link / Create Entry ...

Adverse events

Centre ID	
Patient ID	
Visit	
Any new Adverse events since last visit?	◯yes ◯no
If yes, please document all new Adverse Events in the following section:	
Link to Adverse Events	(see table below)

	AE number	SOC of AE (according CTCAE V5.0)	AE term (according CTCAE V5.0)	Start date	Ongoing at the end of study	Stop date	Is the AE serious?

Set Link / Create Entry ...

Day 8 - Cross-over Assessment

Centre ID	
Patient ID	
Visit	Day 8- Cross-over Assessment
Date of Visit	____ ______ ________
Hospitalization?	◯yes ◯no

- Vital signs
- Seven point ordinal scale
- Sequential Organ Failure Assessment (SOFA) Score
- Oxygen saturation (SaO2)
- Hematology
- Blood chemistry and coagulation
- Concomitant medication
- Concomitant COVID-19 therapy
- Adverse events

Investigator's Signature

Meaning:	I confirm the completeness and correctness of all documented data.
Signed By:	
Signature Date:	

Vital signs

Centre ID	
Patient ID	
Visit	
WHO performance status	◯ECOG 0 = Fully active, able to carry on all pre-disease performance without restriction<br>◯ECOG 1 = Restricted in physically strenuous activity but ambulatory and able to carry out work of a light or sedentary nature, e.g., light house work, office work<br>◯ECOG 2 = Ambulatory and capable of all selfcare but unable to carry out any work activities; up and about more than 50% of waking hours<br>◯ECOG 3 = Capable of only limited self-care, confined to bed or chair more than 50% of waking hours<br>◯ECOG 4 = Completely disabled. Cannot carry on any self-care. Totally confined to bed or chair.
Body temperature	____________________ °C
Type of measurement	◯oral ◯tympanic
Systolic blood pressure	____________________ mmHg
Diastolic blood pressure	____________________ mmHg
Pulse rate	____________________ beats/min
respiratory rate	____________________ breaths/min

Seven point ordinal scale

Centre ID	
Patient ID	
Visit	
Time of record	____ ____ ____
Ordinal Scale for Clinical Improvement	◯1 = not hospitalized with resumption of normal activities<br>◯2 = not hospitalized, but unable to resume normal sctivities<br>◯3 = hospitalized,not requiring supplemental oxygen<br>◯4 = hospitalized, requiring supplemental oxygen<br>◯5 = hospitalized, requiring nasal high-flow oxygen therapy or, noninvasive mechanical ventilation<br>◯6 = hospitalized, requiring ECMO, invasive mechanical ventilation, or both<br>◯7 = death

Sequential Organ Failure Assessment (SOFA) Score

Centre ID	
Patient ID	
Visit	
Respiratory system (PaO2/FiO2 (mmHg))	◯> 400<br>◯< 400<br>◯< 300<br>◯< 200 with respiratoy support<br>◯< 100 with respiratoy support
Nervous system (Glasgow Coma Scale)	◯15<br>◯13-14<br>◯10-12<br>◯6-9<br>◯<6
Cardiovascular system (Mean arterial pressure (MAP) or administration of vasopressor required)	◯MAP > 70 mmHg<br>◯MAP < 70 mmHg<br>◯Dopamine 5 µg/kg/min or dobutamine any dose<br>◯Dopamine > 5 µg/kg/min or epinephrine 0.1 µg/kg/min or norepinephrine 0.1 µg/kg/min<br>◯Dopamine > 15 µg/kg/min OR epinephrine > 0.1 µg/kg/min OR norepinephrine > 0.1 µg/kg/min
Liver (Bilirubin (mg/dl) [µmol/l])	◯< 1.2 [< 20]<br>◯1.2 - 1.9 [20 - 32]<br>◯2.0 - 5.9 [33 - 101]<br>◯6.0 - 11.9 [102 - 204]<br>◯> 12.0 [> 204]
Coagulation (Platelets x103/ml)	◯> 150<br>◯100-150<br>◯50-99<br>◯20-49<br>◯< 20
Kidneys (Creatinine (mg/dl) [µmol/L]; urine output)	◯< 1.2 [< 110]<br>◯1.2 - 1.9 [110 - 170]<br>◯2.0 - 3.4 [171 - 299]<br>◯3. - 4.9 [300 - 440]<br>◯> 5.0 [> 440]

Oxygen saturation (SaO2)

Centre ID	
Patient ID	
Visit	
ECMO	◯yes ◯no
Type of ventilation	◯no<br>◯Nasal high-flow oxygen therapy<br>◯Noninvasive mechanical ventilation<br>◯Invasive ventilation<br>◯Tracheotomy<br>◯Ambient air<br>◯Standard oxygen sources
Amount of oxygen	____________________ liter/min
Oxygen saturation (SaO2)	____________________ %
Amount of supplemental oxygen that is required to keep SaO2 above 94%	____________________ liter/min
Fraction of Inspired Oxygen (FiO2)	____________________ %
Partial pressure of oxygen (PaO2)	____________________ mmHg
Partial pressure of carbon dioxid (PaCO2)	____________________ mmHg

Hematology

Centre ID	
Patient ID	
Visit	
Date of sample taken	____ ______ ________

Hematology

Parameter	not done	Result	Unit
Hemoglobin	◯yes		▼
RBC	◯yes		▼
WBC	◯yes		▼
Thrombocytes	◯yes		▼

Blood chemistry and coagulation

Centre ID	
Patient ID	
Visit	
Date of sample taken	____ ______ ________

Clinical chemistry
<small>Please use a point '.' as decimal separator.</small>

Parameter	not done	Result	Unit
BUN	◯yes		▾
Creatinine	◯yes		▾
Albumin	◯yes		▾
AST/SGOT	◯yes		▾
ALT/SGPT	◯yes		▾
Total bilirubin	◯yes		▾
GGT	◯yes		▾
AP	◯yes		▾
LDH	◯yes		▾
Sodium	◯yes		▾
Potassium	◯yes		▾
Magnesium	◯yes		▾
Calcium	◯yes		▾
Uric acid	◯yes		▾
Troponin	◯yes		▾
CK	◯yes		U/l
CK-MB	◯yes		U/l
PTT	◯yes		sec
ATIII	◯yes		%
D-Dimer	◯yes		▾
Fibrinogen	◯yes		▾
Ferritin	◯yes		▾
Transferrin	◯yes		▾
Transferrin Saturation	◯yes		%
CRP	◯yes		▾
Total protein	◯yes		g/l
IL6	◯yes		pg/ml
Procalcitonin	◯yes		ng/ml
Total IgG	◯yes		▾
IgA	◯yes		▾
IgM	◯yes		▾
Lactate	◯yes		▾
INR	◯yes		

Concomitant medication

Centre ID	
Patient ID	
Visit	
Any new or changed concomitant medication since last visit	◯yes ◯no

If yes, please document all changes in Concomitant medications in the following section:

Link to Concomitant medication

	Seq. no.	Drug Name	Indication	Start date	Ongoing at end of study	Stop date

Set Link / Create Entry ...

Concomitant COVID-19 therapy

Centre ID	
Patient ID	
Visit	
Any new Concomitant COVID-19 therapy since last visit?	◯yes ◯no
If yes, please document all changes in Concomitant COVID-19 therapy in the following section:	
Link to Concomitant COVID-19 therapy	see table below

	Therapy number	Therapy	Total Daily Dose	Unit	Onset date of medication	Still ongoing	Stop date of therapy

Set Link / Create Entry ...

Adverse events

Centre ID	
Patient ID	
Visit	
Any new Adverse events since last visit?	○yes ○no
If yes, please document all new Adverse Events in the following section:	
Link to Adverse Events	(see table below)

	AE number	SOC of AE (according CTCAE V5.0)	AE term (according CTCAE V5.0)	Start date	Ongoing at the end of study	Stop date	Is the AE serious?

Set Link / Create Entry ...

Day 9 - Cross-over Assessment

Centre ID	
Patient ID	
Visit	Day 9 - Cross-over Assessment
Date of Visit	____ ______ ________
Hospitalization?	◯yes ◯no

- Vital signs
- Seven point ordinal scale
- Sequential Organ Failure Assessment (SOFA) Score
- Oxygen saturation (SaO2)
- Hematology
- Blood chemistry and coagulation
- Concomitant medication
- Concomitant COVID-19 therapy
- Adverse events

Investigator's Signature

Meaning:	I confirm the completeness and correctness of all documented data.
Signed By:	
Signature Date:	

Vital signs

Centre ID	
Patient ID	
Visit	
WHO performance status	◯ECOG 0 = Fully active, able to carry on all pre-disease performance without restriction<br>◯ECOG 1 = Restricted in physically strenuous activity but ambulatory and able to carry out work of a light or sedentary nature, e.g., light house work, office work<br>◯ECOG 2 = Ambulatory and capable of all selfcare but unable to carry out any work activities; up and about more than 50% of waking hours<br>◯ECOG 3 = Capable of only limited self-care, confined to bed or chair more than 50% of waking hours<br>◯ECOG 4 = Completely disabled. Cannot carry on any self-care. Totally confined to bed or chair.
Body temperature	____________________ °C
Type of measurement	◯oral ◯tympanic
Systolic blood pressure	____________________ mmHg
Diastolic blood pressure	____________________ mmHg
Pulse rate	____________________ beats/min
respiratory rate	____________________ breaths/min

Seven point ordinal scale

Centre ID	
Patient ID	
Visit	
Time of record	____ ____ ____
Ordinal Scale for Clinical Improvement	◯1 = not hospitalized with resumption of normal activities<br>◯2 = not hospitalized, but unable to resume normal sctivities<br>◯3 = hospitalized,not requiring supplemental oxygen<br>◯4 = hospitalized, requiring supplemental oxygen<br>◯5 = hospitalized, requiring nasal high-flow oxygen therapy or, noninvasive mechanical ventilation<br>◯6 = hospitalized, requiring ECMO, invasive mechanical ventilation, or both<br>◯7 = death

Sequential Organ Failure Assessment (SOFA) Score

Centre ID	
Patient ID	
Visit	
Respiratory system (PaO2/FiO2 (mmHg))	> 400<br>< 400<br>< 300<br>< 200 with respiratoy support<br>< 100 with respiratoy support
Nervous system (Glasgow Coma Scale)	15<br>13-14<br>10-12<br>6-9<br><6
Cardiovascular system (Mean arterial pressure (MAP) or administration of vasopressor required)	MAP > 70 mmHg<br>MAP < 70 mmHg<br>Dopamine 5 µg/kg/min or dobutamine any dose<br>Dopamine > 5 µg/kg/min or epinephrine 0.1 µg/kg/min or norepinephrine 0.1 µg/kg/min<br>Dopamine > 15 µg/kg/min OR epinephrine > 0.1 µg/kg/min OR norepinephrine > 0.1 µg/kg/min
Liver (Bilirubin (mg/dl) [µmol/l])	< 1.2 [< 20]<br>1.2 - 1.9 [20 - 32]<br>2.0 - 5.9 [33 - 101]<br>6.0 - 11.9 [102 - 204]<br>> 12.0 [> 204]
Coagulation (Platelets x103/ml)	> 150<br>100-150<br>50-99<br>20-49<br>< 20
Kidneys (Creatinine (mg/dl) [µmol/L]; urine output)	< 1.2 [< 110]<br>1.2 - 1.9 [110 - 170]<br>2.0 - 3.4 [171 - 299]<br>3. - 4.9 [300 - 440]<br>> 5.0 [> 440]

Oxygen saturation (SaO2)

Centre ID	
Patient ID	
Visit	
ECMO	◯yes ◯no
Type of ventilation	◯no<br>◯Nasal high-flow oxygen therapy<br>◯Noninvasive mechanical ventilation<br>◯Invasive ventilation<br>◯Tracheotomy<br>◯Ambient air<br>◯Standard oxygen sources
Amount of oxygen	____________________ liter/min
Oxygen saturation (SaO2)	____________________ %
Amount of supplemental oxygen that is required to keep SaO2 above 94%	____________________ liter/min
Fraction of Inspired Oxygen (FiO2)	____________________ %
Partial pressure of oxygen (PaO2)	____________________ mmHg
Partial pressure of carbon dioxid (PaCO2)	____________________ mmHg

Hematology

Centre ID	
Patient ID	
Visit	
Date of sample taken	____ ______ ________

Hematology

Parameter	not done	Result	Unit
Hemoglobin	◯yes		
RBC	◯yes		
WBC	◯yes		
Thrombocytes	◯yes		

Blood chemistry and coagulation

Centre ID	
Patient ID	
Visit	
Date of sample taken	____ ______ ________

Clinical chemistry
<small>Please use a point '.' as decimal separator.</small>

Parameter	not done	Result	Unit
BUN	yes		
Creatinine	yes		
Albumin	yes		
AST/SGOT	yes		
ALT/SGPT	yes		
Total bilirubin	yes		
GGT	yes		
AP	yes		
LDH	yes		
Sodium	yes		
Potassium	yes		
Magnesium	yes		
Calcium	yes		
Uric acid	yes		
Troponin	yes		
CK	yes		U/l
CK-MB	yes		U/l
PTT	yes		sec
ATIII	yes		%
D-Dimer	yes		
Fibrinogen	yes		
Ferritin	yes		
Transferrin	yes		
Transferrin Saturation	yes		%
CRP	yes		
Total protein	yes		g/l
IL6	yes		pg/ml
Procalcitonin	yes		ng/ml
Total IgG	yes		
IgA	yes		
IgM	yes		
Lactate	yes		
INR	yes		

Concomitant medication

Centre ID	
Patient ID	
Visit	
Any new or changed concomitant medication since last visit	◯yes ◯no
If yes, please document all changes in Concomitant medications in the following section:	
Link to Concomitant medication	

	Seq. no.	Drug Name	Indication	Start date	Ongoing at end of study	Stop date

Set Link / Create Entry ...

Concomitant COVID-19 therapy

Centre ID	
Patient ID	
Visit	
Any new Concomitant COVID-19 therapy since last visit?	◯yes ◯no
If yes, please document all changes in Concomitant COVID-19 therapy in the following section:	
Link to Concomitant COVID-19 therapy	see table below

	Therapy number	Therapy	Total Daily Dose	Unit	Onset date of medication	Still ongoing	Stop date of therapy

Set Link / Create Entry ...

Adverse events

Centre ID	
Patient ID	
Visit	
Any new Adverse events since last visit?	◯yes ◯no

If yes, please document all new Adverse Events in the following section:

Link to Adverse Events

	AE number	SOC of AE (according CTCAE V5.0)	AE term (according CTCAE V5.0)	Start date	Ongoing at the end of study	Stop date	Is the AE serious?

Set Link / Create Entry ...

Day 10 - Cross-over Assessment

Centre ID	
Patient ID	
Visit	Day 10 - Cross-over Assessment
Date of Visit	____ ______ ________
Hospitalization?	◯yes ◯no

- Vital signs
- Seven point ordinal scale
- Sequential Organ Failure Assessment (SOFA) Score
- Oxygen saturation (SaO2)
- Hematology
- Blood chemistry and coagulation
- SARS-CoV-2 viral clearance and load as well as antibody titres
- Concomitant medication
- Concomitant COVID-19 therapy
- Adverse events

Investigator's Signature

Meaning:	I confirm the completeness and correctness of all documented data.
Signed By:	
Signature Date:	

Vital signs

Centre ID	
Patient ID	
Visit	
WHO performance status	◯ECOG 0 = Fully active, able to carry on all pre-disease performance without restriction<br>◯ECOG 1 = Restricted in physically strenuous activity but ambulatory and able to carry out work of a light or sedentary nature, e.g., light house work, office work<br>◯ECOG 2 = Ambulatory and capable of all selfcare but unable to carry out any work activities; up and about more than 50% of waking hours<br>◯ECOG 3 = Capable of only limited self-care, confined to bed or chair more than 50% of waking hours<br>◯ECOG 4 = Completely disabled. Cannot carry on any self-care. Totally confined to bed or chair.
Body temperature	____________________ °C
Type of measurement	◯oral ◯tympanic
Systolic blood pressure	____________________ mmHg
Diastolic blood pressure	____________________ mmHg
Pulse rate	____________________ beats/min
respiratory rate	____________________ breaths/min

Seven point ordinal scale

Centre ID	
Patient ID	
Visit	
Time of record	____ ____ ____
Ordinal Scale for Clinical Improvement	◯1 = not hospitalized with resumption of normal activities<br>◯2 = not hospitalized, but unable to resume normal sctivities<br>◯3 = hospitalized,not requiring supplemental oxygen<br>◯4 = hospitalized, requiring supplemental oxygen<br>◯5 = hospitalized, requiring nasal high-flow oxygen therapy or, noninvasive mechanical ventilation<br>◯6 = hospitalized, requiring ECMO, invasive mechanical ventilation, or both<br>◯7 = death

Sequential Organ Failure Assessment (SOFA) Score

Centre ID	
Patient ID	
Visit	
Respiratory system (PaO2/FiO2 (mmHg))	◯> 400<br>◯< 400<br>◯< 300<br>◯< 200 with respiratoy support<br>◯< 100 with respiratoy support
Nervous system (Glasgow Coma Scale)	◯15<br>◯13-14<br>◯10-12<br>◯6-9<br>◯<6
Cardiovascular system (Mean arterial pressure (MAP) or administration of vasopressor required)	◯MAP > 70 mmHg<br>◯MAP < 70 mmHg<br>◯Dopamine 5 µg/kg/min or dobutamine any dose<br>◯Dopamine > 5 µg/kg/min or epinephrine 0.1 µg/kg/min or norepinephrine 0.1 µg/kg/min<br>◯Dopamine > 15 µg/kg/min OR epinephrine > 0.1 µg/kg/min OR norepinephrine > 0.1 µg/kg/min
Liver (Bilirubin (mg/dl) [µmol/l])	◯< 1.2 [< 20]<br>◯1.2 - 1.9 [20 - 32]<br>◯2.0 - 5.9 [33 - 101]<br>◯6.0 - 11.9 [102 - 204]<br>◯> 12.0 [> 204]
Coagulation (Platelets x103/ml)	◯> 150<br>◯100-150<br>◯50-99<br>◯20-49<br>◯< 20
Kidneys (Creatinine (mg/dl) [µmol/L]; urine output)	◯< 1.2 [< 110]<br>◯1.2 - 1.9 [110 - 170]<br>◯2.0 - 3.4 [171 - 299]<br>◯3. - 4.9 [300 - 440]<br>◯> 5.0 [> 440]

Oxygen saturation (SaO2)

Centre ID	
Patient ID	
Visit	
ECMO	◯yes ◯no
Type of ventilation	◯no<br>◯Nasal high-flow oxygen therapy<br>◯Noninvasive mechanical ventilation<br>◯Invasive ventilation<br>◯Tracheotomy<br>◯Ambient air<br>◯Standard oxygen sources
Amount of oxygen	____________________ liter/min
Oxygen saturation (SaO2)	____________________ %
Amount of supplemental oxygen that is required to keep SaO2 above 94%	____________________ liter/min
Fraction of Inspired Oxygen (FiO2)	____________________ %
Partial pressure of oxygen (PaO2)	____________________ mmHg
Partial pressure of carbon dioxid (PaCO2)	____________________ mmHg

Hematology

Centre ID	
Patient ID	
Visit	
Date of sample taken	____ ______ ________

Hematology

Parameter	not done	Result	Unit
Hemoglobin	yes		
RBC	yes		
WBC	yes		
Thrombocytes	yes		

Blood chemistry and coagulation

Centre ID	
Patient ID	
Visit	
Date of sample taken	____ ______ ________

Clinical chemistry
<small>Please use a point '.' as decimal separator.</small>

Parameter	not done	Result	Unit
BUN	yes		
Creatinine	yes		
Albumin	yes		
AST/SGOT	yes		
ALT/SGPT	yes		
Total bilirubin	yes		
GGT	yes		
AP	yes		
LDH	yes		
Sodium	yes		
Potassium	yes		
Magnesium	yes		
Calcium	yes		
Uric acid	yes		
Troponin	yes		
CK	yes		U/l
CK-MB	yes		U/l
PTT	yes		sec
ATIII	yes		%
D-Dimer	yes		
Fibrinogen	yes		
Ferritin	yes		
Transferrin	yes		
Transferrin Saturation	yes		%
CRP	yes		
Total protein	yes		g/l
IL6	yes		pg/ml
Procalcitonin	yes		ng/ml
Total IgG	yes		
IgA	yes		
IgM	yes		
Lactate	yes		
INR	yes		

SARS-CoV-2 viral clearance and load as well as antibody titres

Centre ID	
Patient ID	
Visit	
Sample collection on Date of visit?	◯yes ◯no
Date of sample collection	____ ______ ________
Type of smear	◯Nasopharyngeal<br>◯Oropharyngeal<br>◯Sputum<br>◯Other
If other: Please specify	____________________
Result of smear	◯Negative ◯Positive ◯Invalide
PCR test type	◯TibMolbiol<br>◯Seegene<br>◯Abbott<br>◯Altona<br>◯Other
If other: Please specify	____________________
CT-value	____________________
Serodiagnostic by ELISA (OD Ratio)	____________________
Neutralize antibody titre (1:....)	____________________ ▾
Immunofluorescence	◯Positive<br>◯Borderline positive<br>◯Negative<br>◯Not performed

Concomitant medication

Centre ID	
Patient ID	
Visit	
Any new or changed concomitant medication since last visit	◯yes ◯no

If yes, please document all changes in Concomitant medications in the following section:

Link to Concomitant medication

	Seq. no.	Drug Name	Indication	Start date	Ongoing at end of study	Stop date

Set Link / Create Entry ...

Concomitant COVID-19 therapy

Centre ID

Patient ID

Visit

Any new Concomitant COVID-19 therapy since last visit? ◯yes ◯no

If yes, please document all changes in Concomitant COVID-19 therapy in the following section:

Link to Concomitant COVID-19 therapy

	Therapy number	Therapy	Total Daily Dose	Unit	Onset date of medication	Still ongoing	Stop date of therapy

Set Link / Create Entry ...

Adverse events

Centre ID	
Patient ID	
Visit	
Any new Adverse events since last visit?	◯yes ◯no
If yes, please document all new Adverse Events in the following section:	
Link to Adverse Events	see table below

	AE number	SOC of AE (according CTCAE V5.0)	AE term (according CTCAE V5.0)	Start date	Ongoing at the end of study	Stop date	Is the AE serious?

Set Link / Create Entry ...

Day 11 - Cross-over Assessment

Centre ID	
Patient ID	
Visit	Day 11 - Cross-over Assessment
Date of Visit	____ ______ ________
Hospitalization?	◯yes ◯no

- Vital signs
- Seven point ordinal scale
- Sequential Organ Failure Assessment (SOFA) Score
- Oxygen saturation (SaO2)
- Concomitant medication
- Concomitant COVID-19 therapy
- Adverse events

Investigator's Signature

Meaning:	I confirm the completeness and correctness of all documented data.
Signed By:	
Signature Date:	

Vital signs

Centre ID	
Patient ID	
Visit	
WHO performance status	◯ECOG 0 = Fully active, able to carry on all pre-disease performance without restriction<br>◯ECOG 1 = Restricted in physically strenuous activity but ambulatory and able to carry out work of a light or sedentary nature, e.g., light house work, office work<br>◯ECOG 2 = Ambulatory and capable of all selfcare but unable to carry out any work activities; up and about more than 50% of waking hours<br>◯ECOG 3 = Capable of only limited self-care, confined to bed or chair more than 50% of waking hours<br>◯ECOG 4 = Completely disabled. Cannot carry on any self-care. Totally confined to bed or chair.
Body temperature	____________________ °C
Type of measurement	◯oral ◯tympanic
Systolic blood pressure	____________________ mmHg
Diastolic blood pressure	____________________ mmHg
Pulse rate	____________________ beats/min
respiratory rate	____________________ breaths/min

Seven point ordinal scale

Centre ID	
Patient ID	
Visit	
Time of record	____ ____ ____
Ordinal Scale for Clinical Improvement	◯1 = not hospitalized with resumption of normal activities<br>◯2 = not hospitalized, but unable to resume normal sctivities<br>◯3 = hospitalized,not requiring supplemental oxygen<br>◯4 = hospitalized, requiring supplemental oxygen<br>◯5 = hospitalized, requiring nasal high-flow oxygen therapy or, noninvasive mechanical ventilation<br>◯6 = hospitalized, requiring ECMO, invasive mechanical ventilation, or both<br>◯7 = death

Sequential Organ Failure Assessment (SOFA) Score

Centre ID	
Patient ID	
Visit	
Respiratory system (PaO2/FiO2 (mmHg))	◯> 400<br>◯< 400<br>◯< 300<br>◯< 200 with respiratoy support<br>◯< 100 with respiratoy support
Nervous system (Glasgow Coma Scale)	◯15<br>◯13-14<br>◯10-12<br>◯6-9<br>◯<6
Cardiovascular system (Mean arterial pressure (MAP) or administration of vasopressor required)	◯MAP > 70 mmHg<br>◯MAP < 70 mmHg<br>◯Dopamine 5 µg/kg/min or dobutamine any dose<br>◯Dopamine > 5 µg/kg/min or epinephrine 0.1 µg/kg/min or norepinephrine 0.1 µg/kg/min<br>◯Dopamine > 15 µg/kg/min OR epinephrine > 0.1 µg/kg/min OR norepinephrine > 0.1 µg/kg/min
Liver (Bilirubin (mg/dl) [µmol/l])	◯< 1.2 [< 20]<br>◯1.2 - 1.9 [20 - 32]<br>◯2.0 - 5.9 [33 - 101]<br>◯6.0 - 11.9 [102 - 204]<br>◯> 12.0 [> 204]
Coagulation (Platelets x103/ml)	◯> 150<br>◯100-150<br>◯50-99<br>◯20-49<br>◯< 20
Kidneys (Creatinine (mg/dl) [µmol/L]; urine output)	◯< 1.2 [< 110]<br>◯1.2 - 1.9 [110 - 170]<br>◯2.0 - 3.4 [171 - 299]<br>◯3. - 4.9 [300 - 440]<br>◯> 5.0 [> 440]

Oxygen saturation (SaO2)

Centre ID	
Patient ID	
Visit	
ECMO	◯yes ◯no
Type of ventilation	◯no<br>◯Nasal high-flow oxygen therapy<br>◯Noninvasive mechanical ventilation<br>◯Invasive ventilation<br>◯Tracheotomy<br>◯Ambient air<br>◯Standard oxygen sources
Amount of oxygen	____________________ liter/min
Oxygen saturation (SaO2)	____________________ %
Amount of supplemental oxygen that is required to keep SaO2 above 94%	____________________ liter/min
Fraction of Inspired Oxygen (FiO2)	____________________ %
Partial pressure of oxygen (PaO2)	____________________ mmHg
Partial pressure of carbon dioxid (PaCO2)	____________________ mmHg

Concomitant medication

Centre ID	
Patient ID	
Visit	
Any new or changed concomitant medication since last visit	◯yes ◯no
If yes, please document all changes in Concomitant medications in the following section:	
Link to Concomitant medication	

	Seq. no.	Drug Name	Indication	Start date	Ongoing at end of study	Stop date

Set Link / Create Entry ...

Concomitant COVID-19 therapy

Centre ID	
Patient ID	
Visit	
Any new Concomitant COVID-19 therapy since last visit?	◯yes ◯no
If yes, please document all changes in Concomitant COVID-19 therapy in the following section:	
Link to Concomitant COVID-19 therapy	

	Therapy number	Therapy	Total Daily Dose	Unit	Onset date of medication	Still ongoing	Stop date of therapy

Set Link / Create Entry ...

Adverse events

Centre ID	
Patient ID	
Visit	
Any new Adverse events since last visit?	◯yes ◯no

If yes, please document all new Adverse Events in the following section:

Link to Adverse Events

	AE number	SOC of AE (according CTCAE V5.0)	AE term (according CTCAE V5.0)	Start date	Ongoing at the end of study	Stop date	Is the AE serious?

Set Link / Create Entry ...

Day 12 - Cross-over Assessment

Centre ID	
Patient ID	
Visit	Day 12 - Cross-over Assessment
Date of Visit	____ ______ ________
Hospitalization?	◯yes ◯no

- Vital signs
- Seven point ordinal scale
- Sequential Organ Failure Assessment (SOFA) Score
- Oxygen saturation (SaO2)
- Concomitant medication
- Concomitant COVID-19 therapy
- Adverse events

Investigator's Signature

Meaning:	I confirm the completeness and correctness of all documented data.
Signed By:	
Signature Date:	

Vital signs

Centre ID	
Patient ID	
Visit	
WHO performance status	◯ECOG 0 = Fully active, able to carry on all pre-disease performance without restriction<br>◯ECOG 1 = Restricted in physically strenuous activity but ambulatory and able to carry out work of a light or sedentary nature, e.g., light house work, office work<br>◯ECOG 2 = Ambulatory and capable of all selfcare but unable to carry out any work activities; up and about more than 50% of waking hours<br>◯ECOG 3 = Capable of only limited self-care, confined to bed or chair more than 50% of waking hours<br>◯ECOG 4 = Completely disabled. Cannot carry on any self-care. Totally confined to bed or chair.
Body temperature	____________________ °C
Type of measurement	◯oral ◯tympanic
Systolic blood pressure	____________________ mmHg
Diastolic blood pressure	____________________ mmHg
Pulse rate	____________________ beats/min
respiratory rate	____________________ breaths/min

Seven point ordinal scale

Centre ID	
Patient ID	
Visit	
Time of record	____ ____ ____
Ordinal Scale for Clinical Improvement	◯1 = not hospitalized with resumption of normal activities<br>◯2 = not hospitalized, but unable to resume normal sctivities<br>◯3 = hospitalized,not requiring supplemental oxygen<br>◯4 = hospitalized, requiring supplemental oxygen<br>◯5 = hospitalized, requiring nasal high-flow oxygen therapy or, noninvasive mechanical ventilation<br>◯6 = hospitalized, requiring ECMO, invasive mechanical ventilation, or both<br>◯7 = death

Sequential Organ Failure Assessment (SOFA) Score

Centre ID	
Patient ID	
Visit	
Respiratory system (PaO2/FiO2 (mmHg))	> 400<br>< 400<br>< 300<br>< 200 with respiratoy support<br>< 100 with respiratoy support
Nervous system (Glasgow Coma Scale)	15<br>13-14<br>10-12<br>6-9<br><6
Cardiovascular system (Mean arterial pressure (MAP) or administration of vasopressor required)	MAP > 70 mmHg<br>MAP < 70 mmHg<br>Dopamine 5 µg/kg/min or dobutamine any dose<br>Dopamine > 5 µg/kg/min or epinephrine 0.1 µg/kg/min or norepinephrine 0.1 µg/kg/min<br>Dopamine > 15 µg/kg/min OR epinephrine > 0.1 µg/kg/min OR norepinephrine > 0.1 µg/kg/min
Liver (Bilirubin (mg/dl) [µmol/l])	< 1.2 [< 20]<br>1.2 - 1.9 [20 - 32]<br>2.0 - 5.9 [33 - 101]<br>6.0 - 11.9 [102 - 204]<br>> 12.0 [> 204]
Coagulation (Platelets x103/ml)	> 150<br>100-150<br>50-99<br>20-49<br>< 20
Kidneys (Creatinine (mg/dl) [µmol/L]; urine output)	< 1.2 [< 110]<br>1.2 - 1.9 [110 - 170]<br>2.0 - 3.4 [171 - 299]<br>3. - 4.9 [300 - 440]<br>> 5.0 [> 440]

Oxygen saturation (SaO2)

Centre ID	
Patient ID	
Visit	
ECMO	◯yes ◯no
Type of ventilation	◯no<br>◯Nasal high-flow oxygen therapy<br>◯Noninvasive mechanical ventilation<br>◯Invasive ventilation<br>◯Tracheotomy<br>◯Ambient air<br>◯Standard oxygen sources
Amount of oxygen	____________________ liter/min
Oxygen saturation (SaO2)	____________________ %
Amount of supplemental oxygen that is required to keep SaO2 above 94%	____________________ liter/min
Fraction of Inspired Oxygen (FiO2)	____________________ %
Partial pressure of oxygen (PaO2)	____________________ mmHg
Partial pressure of carbon dioxid (PaCO2)	____________________ mmHg

Concomitant medication

Centre ID	
Patient ID	
Visit	
Any new or changed concomitant medication since last visit	◯yes ◯no
If yes, please document all changes in Concomitant medications in the following section:	
Link to Concomitant medication	(see table below)

	Seq. no.	Drug Name	Indicatio n	Start date	Ongoing at end of study	Stop date

Set Link / Create Entry ...

Concomitant COVID-19 therapy

Centre ID	
Patient ID	
Visit	
Any new Concomitant COVID-19 therapy since last visit?	○yes ○no
If yes, please document all changes in Concomitant COVID-19 therapy in the following section:	
Link to Concomitant COVID-19 therapy	

	Therapy number	Therapy	Total Daily Dose	Unit	Onset date of medication	Still ongoing	Stop date of therapy

Set Link / Create Entry ...

Adverse events

Centre ID	
Patient ID	
Visit	
Any new Adverse events since last visit?	◯yes ◯no
If yes, please document all new Adverse Events in the following section:	
Link to Adverse Events	

	AE number	SOC of AE (according CTCAE V5.0)	AE term (according CTCAE V5.0)	Start date	Ongoing at the end of study	Stop date	Is the AE serious?

Set Link / Create Entry ...

Day 13 - Cross-over assessment

Centre ID	
Patient ID	
Visit	Day 13 - Cross-over Assessment
Date of Visit	____ ______ ________
Hospitalization?	◯yes ◯no

- Vital signs
- Seven point ordinal scale
- Sequential Organ Failure Assessment (SOFA) Score
- Oxygen saturation (SaO2)
- Concomitant medication
- Concomitant COVID-19 therapy
- Adverse events

Investigator's Signature

Meaning:	I confirm the completeness and correctness of all documented data.
Signed By:	
Signature Date:	

Vital signs

Centre ID	
Patient ID	
Visit	
WHO performance status	◯ECOG 0 = Fully active, able to carry on all pre-disease performance without restriction<br>◯ECOG 1 = Restricted in physically strenuous activity but ambulatory and able to carry out work of a light or sedentary nature, e.g., light house work, office work<br>◯ECOG 2 = Ambulatory and capable of all selfcare but unable to carry out any work activities; up and about more than 50% of waking hours<br>◯ECOG 3 = Capable of only limited self-care, confined to bed or chair more than 50% of waking hours<br>◯ECOG 4 = Completely disabled. Cannot carry on any self-care. Totally confined to bed or chair.
Body temperature	____________________ °C
Type of measurement	◯oral ◯tympanic
Systolic blood pressure	____________________ mmHg
Diastolic blood pressure	____________________ mmHg
Pulse rate	____________________ beats/min
respiratory rate	____________________ breaths/min

Seven point ordinal scale

Centre ID	
Patient ID	
Visit	
Time of record	____ ____ ____
Ordinal Scale for Clinical Improvement	◯1 = not hospitalized with resumption of normal activities<br>◯2 = not hospitalized, but unable to resume normal sctivities<br>◯3 = hospitalized,not requiring supplemental oxygen<br>◯4 = hospitalized, requiring supplemental oxygen<br>◯5 = hospitalized, requiring nasal high-flow oxygen therapy or, noninvasive mechanical ventilation<br>◯6 = hospitalized, requiring ECMO, invasive mechanical ventilation, or both<br>◯7 = death

Sequential Organ Failure Assessment (SOFA) Score

Centre ID	
Patient ID	
Visit	
Respiratory system (PaO2/FiO2 (mmHg))	◯> 400<br>◯< 400<br>◯< 300<br>◯< 200 with respiratoy support<br>◯< 100 with respiratoy support
Nervous system (Glasgow Coma Scale)	◯15<br>◯13-14<br>◯10-12<br>◯6-9<br>◯<6
Cardiovascular system (Mean arterial pressure (MAP) or administration of vasopressor required)	◯MAP > 70 mmHg<br>◯MAP < 70 mmHg<br>◯Dopamine 5 µg/kg/min or dobutamine any dose<br>◯Dopamine > 5 µg/kg/min or epinephrine 0.1 µg/kg/min or norepinephrine 0.1 µg/kg/min<br>◯Dopamine > 15 µg/kg/min OR epinephrine > 0.1 µg/kg/min OR norepinephrine > 0.1 µg/kg/min
Liver (Bilirubin (mg/dl) [µmol/l])	◯< 1.2 [< 20]<br>◯1.2 - 1.9 [20 - 32]<br>◯2.0 - 5.9 [33 - 101]<br>◯6.0 - 11.9 [102 - 204]<br>◯> 12.0 [> 204]
Coagulation (Platelets x103/ml)	◯> 150<br>◯100-150<br>◯50-99<br>◯20-49<br>◯< 20
Kidneys (Creatinine (mg/dl) [µmol/L]; urine output)	◯< 1.2 [< 110]<br>◯1.2 - 1.9 [110 - 170]<br>◯2.0 - 3.4 [171 - 299]<br>◯3. - 4.9 [300 - 440]<br>◯> 5.0 [> 440]

Oxygen saturation (SaO2)

Centre ID	
Patient ID	
Visit	
ECMO	◯yes ◯no
Type of ventilation	◯no<br>◯Nasal high-flow oxygen therapy<br>◯Noninvasive mechanical ventilation<br>◯Invasive ventilation<br>◯Tracheotomy<br>◯Ambient air<br>◯Standard oxygen sources
Amount of oxygen	____________________ liter/min
Oxygen saturation (SaO2)	____________________ %
Amount of supplemental oxygen that is required to keep SaO2 above 94%	____________________ liter/min
Fraction of Inspired Oxygen (FiO2)	____________________ %
Partial pressure of oxygen (PaO2)	____________________ mmHg
Partial pressure of carbon dioxid (PaCO2)	____________________ mmHg

Concomitant medication

Centre ID	
Patient ID	
Visit	
Any new or changed concomitant medication since last visit	◯yes ◯no
If yes, please document all changes in Concomitant medications in the following section:	
Link to Concomitant medication	

	Seq. no.	Drug Name	Indicatio n	Start date	Ongoing at end of study	Stop date

Set Link / Create Entry ...

Concomitant COVID-19 therapy

Centre ID	
Patient ID	
Visit	
Any new Concomitant COVID-19 therapy since last visit?	○yes ○no
If yes, please document all changes in Concomitant COVID-19 therapy in the following section:	
Link to Concomitant COVID-19 therapy	see table below

	Therapy number	Therapy	Total Daily Dose	Unit	Onset date of medication	Still ongoing	Stop date of therapy

Set Link / Create Entry ...

Adverse events

Centre ID	
Patient ID	
Visit	
Any new Adverse events since last visit?	◯yes ◯no
If yes, please document all new Adverse Events in the following section:	
Link to Adverse Events	

	AE number	SOC of AE (according CTCAE V5.0)	AE term (according CTCAE V5.0)	Start date	Ongoing at the end of study	Stop date	Is the AE serious?

Set Link / Create Entry ...

Day 14 - Cross-over assessment

Centre ID	
Patient ID	
Visit	Day 14 - Cross-over Assessment
Date of Visit	____ ______ ________
Hospitalization?	◯yes ◯no

- Vital signs
- Seven point ordinal scale
- Sequential Organ Failure Assessment (SOFA) Score
- Oxygen saturation (SaO2)
- Hematology
- Blood chemistry and coagulation
- SARS-CoV-2 viral clearance and load as well as antibody titres
- Procurement of Samples for Biobanking
- Concomitant medication
- Concomitant COVID-19 therapy
- Adverse events

Investigator's Signature

Meaning:	I confirm the completeness and correctness of all documented data.
Signed By:	
Signature Date:	

Vital signs

Centre ID	
Patient ID	
Visit	
WHO performance status	◯ECOG 0 = Fully active, able to carry on all pre-disease performance without restriction<br>◯ECOG 1 = Restricted in physically strenuous activity but ambulatory and able to carry out work of a light or sedentary nature, e.g., light house work, office work<br>◯ECOG 2 = Ambulatory and capable of all selfcare but unable to carry out any work activities; up and about more than 50% of waking hours<br>◯ECOG 3 = Capable of only limited self-care, confined to bed or chair more than 50% of waking hours<br>◯ECOG 4 = Completely disabled. Cannot carry on any self-care. Totally confined to bed or chair.
Body temperature	____________________ °C
Type of measurement	◯oral ◯tympanic
Systolic blood pressure	____________________ mmHg
Diastolic blood pressure	____________________ mmHg
Pulse rate	____________________ beats/min
respiratory rate	____________________ breaths/min

Seven point ordinal scale

Centre ID	
Patient ID	
Visit	
Time of record	____ ____ ____
Ordinal Scale for Clinical Improvement	◯1 = not hospitalized with resumption of normal activities<br>◯2 = not hospitalized, but unable to resume normal sctivities<br>◯3 = hospitalized,not requiring supplemental oxygen<br>◯4 = hospitalized, requiring supplemental oxygen<br>◯5 = hospitalized, requiring nasal high-flow oxygen therapy or, noninvasive mechanical ventilation<br>◯6 = hospitalized, requiring ECMO, invasive mechanical ventilation, or both<br>◯7 = death

Sequential Organ Failure Assessment (SOFA) Score

Centre ID	
Patient ID	
Visit	
Respiratory system (PaO2/FiO2 (mmHg))	> 400<br>< 400<br>< 300<br>< 200 with respiratoy support<br>< 100 with respiratoy support
Nervous system (Glasgow Coma Scale)	15<br>13-14<br>10-12<br>6-9<br><6
Cardiovascular system (Mean arterial pressure (MAP) or administration of vasopressor required)	MAP > 70 mmHg<br>MAP < 70 mmHg<br>Dopamine 5 µg/kg/min or dobutamine any dose<br>Dopamine > 5 µg/kg/min or epinephrine 0.1 µg/kg/min or norepinephrine 0.1 µg/kg/min<br>Dopamine > 15 µg/kg/min OR epinephrine > 0.1 µg/kg/min OR norepinephrine > 0.1 µg/kg/min
Liver (Bilirubin (mg/dl) [µmol/l])	< 1.2 [< 20]<br>1.2 - 1.9 [20 - 32]<br>2.0 - 5.9 [33 - 101]<br>6.0 - 11.9 [102 - 204]<br>> 12.0 [> 204]
Coagulation (Platelets x103/ml)	> 150<br>100-150<br>50-99<br>20-49<br>< 20
Kidneys (Creatinine (mg/dl) [µmol/L]; urine output)	< 1.2 [< 110]<br>1.2 - 1.9 [110 - 170]<br>2.0 - 3.4 [171 - 299]<br>3. - 4.9 [300 - 440]<br>> 5.0 [> 440]

Oxygen saturation (SaO2)

Centre ID	
Patient ID	
Visit	
ECMO	◯yes ◯no
Type of ventilation	◯no<br>◯Nasal high-flow oxygen therapy<br>◯Noninvasive mechanical ventilation<br>◯Invasive ventilation<br>◯Tracheotomy<br>◯Ambient air<br>◯Standard oxygen sources
Amount of oxygen	____________________ liter/min
Oxygen saturation (SaO2)	____________________ %
Amount of supplemental oxygen that is required to keep SaO2 above 94%	____________________ liter/min
Fraction of Inspired Oxygen (FiO2)	____________________ %
Partial pressure of oxygen (PaO2)	____________________ mmHg
Partial pressure of carbon dioxid (PaCO2)	____________________ mmHg

Hematology

Centre ID	
Patient ID	
Visit	
Date of sample taken	____ _____ ______

Hematology

Parameter	not done	Result	Unit
Hemoglobin	◯yes		▼
RBC	◯yes		▼
WBC	◯yes		▼
Thrombocytes	◯yes		▼

Blood chemistry and coagulation

Centre ID	
Patient ID	
Visit	
Date of sample taken	____ ______ ________

Clinical chemistry
<small>Please use a point '.' as decimal separator.</small>

Parameter	not done	Result	Unit
BUN	yes		
Creatinine	yes		
Albumin	yes		
AST/SGOT	yes		
ALT/SGPT	yes		
Total bilirubin	yes		
GGT	yes		
AP	yes		
LDH	yes		
Sodium	yes		
Potassium	yes		
Magnesium	yes		
Calcium	yes		
Uric acid	yes		
Troponin	yes		
CK	yes		U/l
CK-MB	yes		U/l
PTT	yes		sec
ATIII	yes		%
D-Dimer	yes		
Fibrinogen	yes		
Ferritin	yes		
Transferrin	yes		
Transferrin Saturation	yes		%
CRP	yes		
Total protein	yes		g/l
IL6	yes		pg/ml
Procalcitonin	yes		ng/ml
Total IgG	yes		
IgA	yes		
IgM	yes		
Lactate	yes		
INR	yes		

SARS-CoV-2 viral clearance and load as well as antibody titres

Centre ID	
Patient ID	
Visit	
Sample collection on Date of visit?	◯yes ◯no
Date of sample collection	____ ______ ________
Type of smear	◯Nasopharyngeal<br>◯Oropharyngeal<br>◯Sputum<br>◯Other
If other: Please specify	____________________
Result of smear	◯Negative ◯Positive ◯Invalide
PCR test type	◯TibMolbiol<br>◯Seegene<br>◯Abbott<br>◯Altona<br>◯Other
If other: Please specify	____________________
CT-value	____________________
Serodiagnostic by ELISA (OD Ratio)	____________________
Neutralize antibody titre (1:....)	____________________ ▾
Immunofluorescence	◯Positive<br>◯Borderline positive<br>◯Negative<br>◯Not performed

Procurement of Samples for Biobanking

Centre ID	
Patient ID	
Visit	
1 x 7,5 ml serum taken?	◯yes ◯no
Sample collection on Date of visit?	◯yes ◯no
Date of collection	____ ______ ________
Time of collection	____ ____ ____
Material	◯complete ◯less material
if no: Reason	____________________
2 x 7,5 ml Lithium-heparin taken?	◯yes ◯no
Sample collection on Date of visit?	◯yes ◯no
Date of collection	____ ______ ________
Time of collection	____ ____ ____
Material	◯complete ◯less material
if no: Reason	____________________

Concomitant medication

Centre ID	
Patient ID	
Visit	
Any new or changed concomitant medication since last visit	○yes ○no
If yes, please document all changes in Concomitant medications in the following section:	
Link to Concomitant medication	

	Seq. no.	Drug Name	Indicatio n	Start date	Ongoing at end of study	Stop date

Set Link / Create Entry ...

Concomitant COVID-19 therapy

Centre ID	
Patient ID	
Visit	
Any new Concomitant COVID-19 therapy since last visit?	◯yes ◯no
If yes, please document all changes in Concomitant COVID-19 therapy in the following section:	
Link to Concomitant COVID-19 therapy	see table below

	Therapy number	Therapy	Total Daily Dose	Unit	Onset date of medication	Still ongoing	Stop date of therapy

Set Link / Create Entry ...

Adverse events

Centre ID	
Patient ID	
Visit	
Any new Adverse events since last visit?	◯yes ◯no
If yes, please document all new Adverse Events in the following section:	
Link to Adverse Events	

	AE number	SOC of AE (according CTCAE V5.0)	AE term (according CTCAE V5.0)	Start date	Ongoing at the end of study	Stop date	Is the AE serious?

Set Link / Create Entry ...

Day 15 - Cross-over assessment

Centre ID	
Patient ID	
Visit	Day 15 - Cross-over Assessment
Date of Visit	____ ______ ________
Hospitalization?	◯yes ◯no

- Vital signs
- Seven point ordinal scale
- Sequential Organ Failure Assessment (SOFA) Score
- Oxygen saturation (SaO2)
- Concomitant medication
- Concomitant COVID-19 therapy
- Adverse events

Investigator's Signature

Meaning:	I confirm the completeness and correctness of all documented data.
Signed By:	
Signature Date:	

Vital signs

Centre ID	
Patient ID	
Visit	
WHO performance status	◯ECOG 0 = Fully active, able to carry on all pre-disease performance without restriction<br>◯ECOG 1 = Restricted in physically strenuous activity but ambulatory and able to carry out work of a light or sedentary nature, e.g., light house work, office work<br>◯ECOG 2 = Ambulatory and capable of all selfcare but unable to carry out any work activities; up and about more than 50% of waking hours<br>◯ECOG 3 = Capable of only limited self-care, confined to bed or chair more than 50% of waking hours<br>◯ECOG 4 = Completely disabled. Cannot carry on any self-care. Totally confined to bed or chair.
Body temperature	____________________ °C
Type of measurement	◯oral ◯tympanic
Systolic blood pressure	____________________ mmHg
Diastolic blood pressure	____________________ mmHg
Pulse rate	____________________ beats/min
respiratory rate	____________________ breaths/min

Seven point ordinal scale

Centre ID	
Patient ID	
Visit	
Time of record	____ ____ ____
Ordinal Scale for Clinical Improvement	◯1 = not hospitalized with resumption of normal activities<br>◯2 = not hospitalized, but unable to resume normal sctivities<br>◯3 = hospitalized,not requiring supplemental oxygen<br>◯4 = hospitalized, requiring supplemental oxygen<br>◯5 = hospitalized, requiring nasal high-flow oxygen therapy or, noninvasive mechanical ventilation<br>◯6 = hospitalized, requiring ECMO, invasive mechanical ventilation, or both<br>◯7 = death

Sequential Organ Failure Assessment (SOFA) Score

Centre ID	
Patient ID	
Visit	
Respiratory system (PaO2/FiO2 (mmHg))	> 400<br>< 400<br>< 300<br>< 200 with respiratoy support<br>< 100 with respiratoy support
Nervous system (Glasgow Coma Scale)	15<br>13-14<br>10-12<br>6-9<br><6
Cardiovascular system (Mean arterial pressure (MAP) or administration of vasopressor required)	MAP > 70 mmHg<br>MAP < 70 mmHg<br>Dopamine 5 µg/kg/min or dobutamine any dose<br>Dopamine > 5 µg/kg/min or epinephrine 0.1 µg/kg/min or norepinephrine 0.1 µg/kg/min<br>Dopamine > 15 µg/kg/min OR epinephrine > 0.1 µg/kg/min OR norepinephrine > 0.1 µg/kg/min
Liver (Bilirubin (mg/dl) [µmol/l])	< 1.2 [< 20]<br>1.2 - 1.9 [20 - 32]<br>2.0 - 5.9 [33 - 101]<br>6.0 - 11.9 [102 - 204]<br>> 12.0 [> 204]
Coagulation (Platelets x103/ml)	> 150<br>100-150<br>50-99<br>20-49<br>< 20
Kidneys (Creatinine (mg/dl) [µmol/L]; urine output)	< 1.2 [< 110]<br>1.2 - 1.9 [110 - 170]<br>2.0 - 3.4 [171 - 299]<br>3. - 4.9 [300 - 440]<br>> 5.0 [> 440]

Oxygen saturation (SaO2)

Centre ID	
Patient ID	
Visit	
ECMO	◯yes ◯no
Type of ventilation	◯no<br>◯Nasal high-flow oxygen therapy<br>◯Noninvasive mechanical ventilation<br>◯Invasive ventilation<br>◯Tracheotomy<br>◯Ambient air<br>◯Standard oxygen sources
Amount of oxygen	____________________ liter/min
Oxygen saturation (SaO2)	____________________ %
Amount of supplemental oxygen that is required to keep SaO2 above 94%	____________________ liter/min
Fraction of Inspired Oxygen (FiO2)	____________________ %
Partial pressure of oxygen (PaO2)	____________________ mmHg
Partial pressure of carbon dioxid (PaCO2)	____________________ mmHg

Concomitant medication

Centre ID	
Patient ID	
Visit	
Any new or changed concomitant medication since last visit	○yes ○no
If yes, please document all changes in Concomitant medications in the following section:	
Link to Concomitant medication	(see table below)

	Seq. no.	Drug Name	Indication	Start date	Ongoing at end of study	Stop date

Set Link / Create Entry ...

Concomitant COVID-19 therapy

Centre ID	
Patient ID	
Visit	
Any new Concomitant COVID-19 therapy since last visit?	○yes ○no
If yes, please document all changes in Concomitant COVID-19 therapy in the following section:	
Link to Concomitant COVID-19 therapy	

	Therapy number	Therapy	Total Daily Dose	Unit	Onset date of medication	Still ongoing	Stop date of therapy

Set Link / Create Entry ...

Adverse events

Centre ID	
Patient ID	
Visit	
Any new Adverse events since last visit?	◯yes ◯no
If yes, please document all new Adverse Events in the following section:	
Link to Adverse Events	(see table below)

	AE number	SOC of AE (according CTCAE V5.0)	AE term (according CTCAE V5.0)	Start date	Ongoing at the end of study	Stop date	Is the AE serious?

Set Link / Create Entry ...

Day 11

Centre ID	
Patient ID	
Visit	Day 11
Date of Visit	____ ______ ________
Hospitalization?	◯yes ◯no

- Vital signs
- Seven point ordinal scale
- Sequential Organ Failure Assessment (SOFA) Score
- Oxygen saturation (SaO2)
- Concomitant medication
- Concomitant COVID-19 therapy
- Adverse events

Investigator's Signature

Meaning:	I confirm the completeness and correctness of all documented data.
Signed By:	
Signature Date:	

Vital signs

Centre ID	
Patient ID	
Visit	
WHO performance status	◯ECOG 0 = Fully active, able to carry on all pre-disease performance without restriction<br>◯ECOG 1 = Restricted in physically strenuous activity but ambulatory and able to carry out work of a light or sedentary nature, e.g., light house work, office work<br>◯ECOG 2 = Ambulatory and capable of all selfcare but unable to carry out any work activities; up and about more than 50% of waking hours<br>◯ECOG 3 = Capable of only limited self-care, confined to bed or chair more than 50% of waking hours<br>◯ECOG 4 = Completely disabled. Cannot carry on any self-care. Totally confined to bed or chair.
Body temperature	____________________ °C
Type of measurement	◯oral ◯tympanic
Systolic blood pressure	____________________ mmHg
Diastolic blood pressure	____________________ mmHg
Pulse rate	____________________ beats/min
respiratory rate	____________________ breaths/min

Seven point ordinal scale

Centre ID	
Patient ID	
Visit	
Time of record	____ ____ ____
Ordinal Scale for Clinical Improvement	◯1 = not hospitalized with resumption of normal activities<br>◯2 = not hospitalized, but unable to resume normal sctivities<br>◯3 = hospitalized,not requiring supplemental oxygen<br>◯4 = hospitalized, requiring supplemental oxygen<br>◯5 = hospitalized, requiring nasal high-flow oxygen therapy or, noninvasive mechanical ventilation<br>◯6 = hospitalized, requiring ECMO, invasive mechanical ventilation, or both<br>◯7 = death

Sequential Organ Failure Assessment (SOFA) Score

Centre ID	
Patient ID	
Visit	
Respiratory system (PaO2/FiO2 (mmHg))	> 400<br>< 400<br>< 300<br>< 200 with respiratoy support<br>< 100 with respiratoy support
Nervous system (Glasgow Coma Scale)	15<br>13-14<br>10-12<br>6-9<br><6
Cardiovascular system (Mean arterial pressure (MAP) or administration of vasopressor required)	MAP > 70 mmHg<br>MAP < 70 mmHg<br>Dopamine 5 µg/kg/min or dobutamine any dose<br>Dopamine > 5 µg/kg/min or epinephrine 0.1 µg/kg/min or norepinephrine 0.1 µg/kg/min<br>Dopamine > 15 µg/kg/min OR epinephrine > 0.1 µg/kg/min OR norepinephrine > 0.1 µg/kg/min
Liver (Bilirubin (mg/dl) [µmol/l])	< 1.2 [< 20]<br>1.2 - 1.9 [20 - 32]<br>2.0 - 5.9 [33 - 101]<br>6.0 - 11.9 [102 - 204]<br>> 12.0 [> 204]
Coagulation (Platelets x103/ml)	> 150<br>100-150<br>50-99<br>20-49<br>< 20
Kidneys (Creatinine (mg/dl) [µmol/L]; urine output)	< 1.2 [< 110]<br>1.2 - 1.9 [110 - 170]<br>2.0 - 3.4 [171 - 299]<br>3. - 4.9 [300 - 440]<br>> 5.0 [> 440]

Oxygen saturation (SaO2)

Centre ID	
Patient ID	
Visit	
ECMO	◯yes ◯no
Type of ventilation	◯no<br>◯Nasal high-flow oxygen therapy<br>◯Noninvasive mechanical ventilation<br>◯Invasive ventilation<br>◯Tracheotomy<br>◯Ambient air<br>◯Standard oxygen sources
Amount of oxygen	____________________ liter/min
Oxygen saturation (SaO2)	____________________ %
Amount of supplemental oxygen that is required to keep SaO2 above 94%	____________________ liter/min
Fraction of Inspired Oxygen (FiO2)	____________________ %
Partial pressure of oxygen (PaO2)	____________________ mmHg
Partial pressure of carbon dioxid (PaCO2)	____________________ mmHg

Concomitant medication

Centre ID	
Patient ID	
Visit	
Any new or changed concomitant medication since last visit	◯yes ◯no
If yes, please document all changes in Concomitant medications in the following section:	
Link to Concomitant medication	(see table below)

	Seq. no.	Drug Name	Indicatio n	Start date	Ongoing at end of study	Stop date

Set Link / Create Entry ...

Concomitant COVID-19 therapy

Centre ID	
Patient ID	
Visit	
Any new Concomitant COVID-19 therapy since last visit?	◯yes ◯no

If yes, please document all changes in Concomitant COVID-19 therapy in the following section:

Link to Concomitant COVID-19 therapy

	Therapy number	Therapy	Total Daily Dose	Unit	Onset date of medication	Still ongoing	Stop date of therapy

Set Link / Create Entry ...

Adverse events

Centre ID	
Patient ID	
Visit	
Any new Adverse events since last visit?	◯yes ◯no

If yes, please document all new Adverse Events in the following section:

Link to Adverse Events

	AE number	SOC of AE (according CTCAE V5.0)	AE term (according CTCAE V5.0)	Start date	Ongoing at the end of study	Stop date	Is the AE serious?

Set Link / Create Entry ...

Day 12

Centre ID	
Patient ID	
Visit	Day 12
Date of Visit	____ ______ ________
Hospitalization?	◯yes ◯no

- Vital signs
- Seven point ordinal scale
- Sequential Organ Failure Assessment (SOFA) Score
- Oxygen saturation (SaO2)
- Concomitant medication
- Concomitant COVID-19 therapy
- Adverse events

Investigator's Signature

Meaning:	I confirm the completeness and correctness of all documented data.
Signed By:	
Signature Date:	

Vital signs

Centre ID	
Patient ID	
Visit	
WHO performance status	◯ECOG 0 = Fully active, able to carry on all pre-disease performance without restriction<br>◯ECOG 1 = Restricted in physically strenuous activity but ambulatory and able to carry out work of a light or sedentary nature, e.g., light house work, office work<br>◯ECOG 2 = Ambulatory and capable of all selfcare but unable to carry out any work activities; up and about more than 50% of waking hours<br>◯ECOG 3 = Capable of only limited self-care, confined to bed or chair more than 50% of waking hours<br>◯ECOG 4 = Completely disabled. Cannot carry on any self-care. Totally confined to bed or chair.
Body temperature	____________________ °C
Type of measurement	◯oral ◯tympanic
Systolic blood pressure	____________________ mmHg
Diastolic blood pressure	____________________ mmHg
Pulse rate	____________________ beats/min
respiratory rate	____________________ breaths/min

Seven point ordinal scale

Centre ID	
Patient ID	
Visit	
Time of record	____ ____ ____
Ordinal Scale for Clinical Improvement	◯1 = not hospitalized with resumption of normal activities<br>◯2 = not hospitalized, but unable to resume normal sctivities<br>◯3 = hospitalized,not requiring supplemental oxygen<br>◯4 = hospitalized, requiring supplemental oxygen<br>◯5 = hospitalized, requiring nasal high-flow oxygen therapy or, noninvasive mechanical ventilation<br>◯6 = hospitalized, requiring ECMO, invasive mechanical ventilation, or both<br>◯7 = death

Sequential Organ Failure Assessment (SOFA) Score

Centre ID	
Patient ID	
Visit	
Respiratory system (PaO2/FiO2 (mmHg))	◯> 400<br>◯< 400<br>◯< 300<br>◯< 200 with respiratoy support<br>◯< 100 with respiratoy support
Nervous system (Glasgow Coma Scale)	◯15<br>◯13-14<br>◯10-12<br>◯6-9<br>◯<6
Cardiovascular system (Mean arterial pressure (MAP) or administration of vasopressor required)	◯MAP > 70 mmHg<br>◯MAP < 70 mmHg<br>◯Dopamine 5 µg/kg/min or dobutamine any dose<br>◯Dopamine > 5 µg/kg/min or epinephrine 0.1 µg/kg/min or norepinephrine 0.1 µg/kg/min<br>◯Dopamine > 15 µg/kg/min OR epinephrine > 0.1 µg/kg/min OR norepinephrine > 0.1 µg/kg/min
Liver (Bilirubin (mg/dl) [µmol/l])	◯< 1.2 [< 20]<br>◯1.2 - 1.9 [20 - 32]<br>◯2.0 - 5.9 [33 - 101]<br>◯6.0 - 11.9 [102 - 204]<br>◯> 12.0 [> 204]
Coagulation (Platelets x103/ml)	◯> 150<br>◯100-150<br>◯50-99<br>◯20-49<br>◯< 20
Kidneys (Creatinine (mg/dl) [µmol/L]; urine output)	◯< 1.2 [< 110]<br>◯1.2 - 1.9 [110 - 170]<br>◯2.0 - 3.4 [171 - 299]<br>◯3. - 4.9 [300 - 440]<br>◯> 5.0 [> 440]

Oxygen saturation (SaO2)

Centre ID	
Patient ID	
Visit	
ECMO	◯yes ◯no
Type of ventilation	◯no<br>◯Nasal high-flow oxygen therapy<br>◯Noninvasive mechanical ventilation<br>◯Invasive ventilation<br>◯Tracheotomy<br>◯Ambient air<br>◯Standard oxygen sources
Amount of oxygen	____________________ liter/min
Oxygen saturation (SaO2)	____________________ %
Amount of supplemental oxygen that is required to keep SaO2 above 94%	____________________ liter/min
Fraction of Inspired Oxygen (FiO2)	____________________ %
Partial pressure of oxygen (PaO2)	____________________ mmHg
Partial pressure of carbon dioxid (PaCO2)	____________________ mmHg

Concomitant medication

Centre ID	
Patient ID	
Visit	
Any new or changed concomitant medication since last visit	◯yes ◯no
If yes, please document all changes in Concomitant medications in the following section:	
Link to Concomitant medication	

	Seq. no.	Drug Name	Indicatio n	Start date	Ongoing at end of study	Stop date

Set Link / Create Entry ...

Concomitant COVID-19 therapy

Centre ID	
Patient ID	
Visit	
Any new Concomitant COVID-19 therapy since last visit?	◯yes ◯no
If yes, please document all changes in Concomitant COVID-19 therapy in the following section:	
Link to Concomitant COVID-19 therapy	

	Therapy number	Therapy	Total Daily Dose	Unit	Onset date of medication	Still ongoing	Stop date of therapy

Set Link / Create Entry ...

Adverse events

Centre ID	
Patient ID	
Visit	
Any new Adverse events since last visit?	◯yes ◯no

If yes, please document all new Adverse Events in the following section:

Link to Adverse Events

	AE number	SOC of AE (according CTCAE V5.0)	AE term (according CTCAE V5.0)	Start date	Ongoing at the end of study	Stop date	Is the AE serious?

Set Link / Create Entry ...

Day 13

Centre ID	
Patient ID	
Visit	Day 13
Date of Visit	____ ______ ________
Hospitalization?	◯yes ◯no

- Vital signs
- Seven point ordinal scale
- Sequential Organ Failure Assessment (SOFA) Score
- Oxygen saturation (SaO2)
- Concomitant medication
- Concomitant COVID-19 therapy
- Adverse events

Investigator's Signature

Meaning:	I confirm the completeness and correctness of all documented data.
Signed By:	
Signature Date:	

Vital signs

Centre ID	
Patient ID	
Visit	
WHO performance status	◯ECOG 0 = Fully active, able to carry on all pre-disease performance without restriction<br>◯ECOG 1 = Restricted in physically strenuous activity but ambulatory and able to carry out work of a light or sedentary nature, e.g., light house work, office work<br>◯ECOG 2 = Ambulatory and capable of all selfcare but unable to carry out any work activities; up and about more than 50% of waking hours<br>◯ECOG 3 = Capable of only limited self-care, confined to bed or chair more than 50% of waking hours<br>◯ECOG 4 = Completely disabled. Cannot carry on any self-care. Totally confined to bed or chair.
Body temperature	____________________ °C
Type of measurement	◯oral ◯tympanic
Systolic blood pressure	____________________ mmHg
Diastolic blood pressure	____________________ mmHg
Pulse rate	____________________ beats/min
respiratory rate	____________________ breaths/min

Seven point ordinal scale

Centre ID	
Patient ID	
Visit	
Time of record	____ ____ ____
Ordinal Scale for Clinical Improvement	◯1 = not hospitalized with resumption of normal activities<br>◯2 = not hospitalized, but unable to resume normal sctivities<br>◯3 = hospitalized,not requiring supplemental oxygen<br>◯4 = hospitalized, requiring supplemental oxygen<br>◯5 = hospitalized, requiring nasal high-flow oxygen therapy or, noninvasive mechanical ventilation<br>◯6 = hospitalized, requiring ECMO, invasive mechanical ventilation, or both<br>◯7 = death

Sequential Organ Failure Assessment (SOFA) Score

Centre ID	
Patient ID	
Visit	
Respiratory system (PaO2/FiO2 (mmHg))	◯> 400<br>◯< 400<br>◯< 300<br>◯< 200 with respiratoy support<br>◯< 100 with respiratoy support
Nervous system (Glasgow Coma Scale)	◯15<br>◯13-14<br>◯10-12<br>◯6-9<br>◯<6
Cardiovascular system (Mean arterial pressure (MAP) or administration of vasopressor required)	◯MAP > 70 mmHg<br>◯MAP < 70 mmHg<br>◯Dopamine 5 µg/kg/min or dobutamine any dose<br>◯Dopamine > 5 µg/kg/min or epinephrine 0.1 µg/kg/min or norepinephrine 0.1 µg/kg/min<br>◯Dopamine > 15 µg/kg/min OR epinephrine > 0.1 µg/kg/min OR norepinephrine > 0.1 µg/kg/min
Liver (Bilirubin (mg/dl) [µmol/l])	◯< 1.2 [< 20]<br>◯1.2 - 1.9 [20 - 32]<br>◯2.0 - 5.9 [33 - 101]<br>◯6.0 - 11.9 [102 - 204]<br>◯> 12.0 [> 204]
Coagulation (Platelets x103/ml)	◯> 150<br>◯100-150<br>◯50-99<br>◯20-49<br>◯< 20
Kidneys (Creatinine (mg/dl) [µmol/L]; urine output)	◯< 1.2 [< 110]<br>◯1.2 - 1.9 [110 - 170]<br>◯2.0 - 3.4 [171 - 299]<br>◯3. - 4.9 [300 - 440]<br>◯> 5.0 [> 440]

Oxygen saturation (SaO2)

Centre ID	
Patient ID	
Visit	
ECMO	◯yes ◯no
Type of ventilation	◯no<br>◯Nasal high-flow oxygen therapy<br>◯Noninvasive mechanical ventilation<br>◯Invasive ventilation<br>◯Tracheotomy<br>◯Ambient air<br>◯Standard oxygen sources
Amount of oxygen	____________________ liter/min
Oxygen saturation (SaO2)	____________________ %
Amount of supplemental oxygen that is required to keep SaO2 above 94%	____________________ liter/min
Fraction of Inspired Oxygen (FiO2)	____________________ %
Partial pressure of oxygen (PaO2)	____________________ mmHg
Partial pressure of carbon dioxid (PaCO2)	____________________ mmHg

Concomitant medication

Centre ID	
Patient ID	
Visit	
Any new or changed concomitant medication since last visit	○yes ○no
If yes, please document all changes in Concomitant medications in the following section:	
Link to Concomitant medication	

	Seq. no.	Drug Name	Indication	Start date	Ongoing at end of study	Stop date

Set Link / Create Entry ...

Concomitant COVID-19 therapy

Centre ID	
Patient ID	
Visit	
Any new Concomitant COVID-19 therapy since last visit?	○yes ○no

If yes, please document all changes in Concomitant COVID-19 therapy in the following section:

Link to Concomitant COVID-19 therapy

	Therapy number	Therapy	Total Daily Dose	Unit	Onset date of medication	Still ongoing	Stop date of therapy

Set Link / Create Entry ...

Adverse events

Centre ID	
Patient ID	
Visit	
Any new Adverse events since last visit?	◯yes ◯no

If yes, please document all new Adverse Events in the following section:

Link to Adverse Events

	AE number	SOC of AE (according CTCAE V5.0)	AE term (according CTCAE V5.0)	Start date	Ongoing at the end of study	Stop date	Is the AE serious?

Set Link / Create Entry ...

Day 14

Centre ID	
Patient ID	
Visit	Day 14
Date of Visit	____ ______ ________
Hospitalization?	◯yes ◯no

- Vital signs
- Seven point ordinal scale
- Sequential Organ Failure Assessment (SOFA) Score
- Oxygen saturation (SaO2)
- Hematology
- Blood chemistry and coagulation
- SARS-CoV-2 viral clearance and load as well as antibody titres
- Procurement of Samples for Biobanking
- Concomitant medication
- Concomitant COVID-19 therapy
- Adverse events

Investigator's Signature

Meaning:	I confirm the completeness and correctness of all documented data.
Signed By:	
Signature Date:	

Vital signs

Centre ID	
Patient ID	
Visit	
WHO performance status	◯ECOG 0 = Fully active, able to carry on all pre-disease performance without restriction<br>◯ECOG 1 = Restricted in physically strenuous activity but ambulatory and able to carry out work of a light or sedentary nature, e.g., light house work, office work<br>◯ECOG 2 = Ambulatory and capable of all selfcare but unable to carry out any work activities; up and about more than 50% of waking hours<br>◯ECOG 3 = Capable of only limited self-care, confined to bed or chair more than 50% of waking hours<br>◯ECOG 4 = Completely disabled. Cannot carry on any self-care. Totally confined to bed or chair.
Body temperature	____________________ °C
Type of measurement	◯oral ◯tympanic
Systolic blood pressure	____________________ mmHg
Diastolic blood pressure	____________________ mmHg
Pulse rate	____________________ beats/min
respiratory rate	____________________ breaths/min

Seven point ordinal scale

Centre ID	
Patient ID	
Visit	
Time of record	____ ____ ____
Ordinal Scale for Clinical Improvement	◯1 = not hospitalized with resumption of normal activities<br>◯2 = not hospitalized, but unable to resume normal sctivities<br>◯3 = hospitalized,not requiring supplemental oxygen<br>◯4 = hospitalized, requiring supplemental oxygen<br>◯5 = hospitalized, requiring nasal high-flow oxygen therapy or, noninvasive mechanical ventilation<br>◯6 = hospitalized, requiring ECMO, invasive mechanical ventilation, or both<br>◯7 = death

Sequential Organ Failure Assessment (SOFA) Score

Centre ID	
Patient ID	
Visit	
Respiratory system (PaO2/FiO2 (mmHg))	◯> 400<br>◯< 400<br>◯< 300<br>◯< 200 with respiratoy support<br>◯< 100 with respiratoy support
Nervous system (Glasgow Coma Scale)	◯15<br>◯13-14<br>◯10-12<br>◯6-9<br>◯<6
Cardiovascular system (Mean arterial pressure (MAP) or administration of vasopressor required)	◯MAP > 70 mmHg<br>◯MAP < 70 mmHg<br>◯Dopamine 5 µg/kg/min or dobutamine any dose<br>◯Dopamine > 5 µg/kg/min or epinephrine 0.1 µg/kg/min or norepinephrine 0.1 µg/kg/min<br>◯Dopamine > 15 µg/kg/min OR epinephrine > 0.1 µg/kg/min OR norepinephrine > 0.1 µg/kg/min
Liver (Bilirubin (mg/dl) [µmol/l])	◯< 1.2 [< 20]<br>◯1.2 - 1.9 [20 - 32]<br>◯2.0 - 5.9 [33 - 101]<br>◯6.0 - 11.9 [102 - 204]<br>◯> 12.0 [> 204]
Coagulation (Platelets x103/ml)	◯> 150<br>◯100-150<br>◯50-99<br>◯20-49<br>◯< 20
Kidneys (Creatinine (mg/dl) [µmol/L]; urine output)	◯< 1.2 [< 110]<br>◯1.2 - 1.9 [110 - 170]<br>◯2.0 - 3.4 [171 - 299]<br>◯3. - 4.9 [300 - 440]<br>◯> 5.0 [> 440]

Oxygen saturation (SaO2)

Centre ID	
Patient ID	
Visit	
ECMO	◯yes ◯no
Type of ventilation	◯no<br>◯Nasal high-flow oxygen therapy<br>◯Noninvasive mechanical ventilation<br>◯Invasive ventilation<br>◯Tracheotomy<br>◯Ambient air<br>◯Standard oxygen sources
Amount of oxygen	____________________ liter/min
Oxygen saturation (SaO2)	____________________ %
Amount of supplemental oxygen that is required to keep SaO2 above 94%	____________________ liter/min
Fraction of Inspired Oxygen (FiO2)	____________________ %
Partial pressure of oxygen (PaO2)	____________________ mmHg
Partial pressure of carbon dioxid (PaCO2)	____________________ mmHg

Hematology

Centre ID	
Patient ID	
Visit	
Date of sample taken	____ ______ ________

Hematology

Parameter	not done	Result	Unit
Hemoglobin	◯yes		▼
RBC	◯yes		▼
WBC	◯yes		▼
Thrombocytes	◯yes		▼

Blood chemistry and coagulation

Centre ID	
Patient ID	
Visit	
Date of sample taken	____ _____ _______

Clinical chemistry
<small>Please use a point '.' as decimal separator.</small>

Parameter	not done	Result	Unit
BUN	yes		
Creatinine	yes		
Albumin	yes		
AST/SGOT	yes		
ALT/SGPT	yes		
Total bilirubin	yes		
GGT	yes		
AP	yes		
LDH	yes		
Sodium	yes		
Potassium	yes		
Magnesium	yes		
Calcium	yes		
Uric acid	yes		
Troponin	yes		
CK	yes		U/l
CK-MB	yes		U/l
PTT	yes		sec
ATIII	yes		%
D-Dimer	yes		
Fibrinogen	yes		
Ferritin	yes		
Transferrin	yes		
Transferrin Saturation	yes		%
CRP	yes		
Total protein	yes		g/l
IL6	yes		pg/ml
Procalcitonin	yes		ng/ml
Total IgG	yes		
IgA	yes		
IgM	yes		
Lactate	yes		
INR	yes		

SARS-CoV-2 viral clearance and load as well as antibody titres

Centre ID	
Patient ID	
Visit	
Sample collection on Date of visit?	◯yes ◯no
Date of sample collection	____ ______ ________
Type of smear	◯Nasopharyngeal<br>◯Oropharyngeal<br>◯Sputum<br>◯Other
If other: Please specify	____________________
Result of smear	◯Negative ◯Positive ◯Invalide
PCR test type	◯TibMolbiol<br>◯Seegene<br>◯Abbott<br>◯Altona<br>◯Other
If other: Please specify	____________________
CT-value	____________________
Serodiagnostic by ELISA (OD Ratio)	____________________
Neutralize antibody titre (1:....)	____________________ ▼
Immunofluorescence	◯Positive<br>◯Borderline positive<br>◯Negative<br>◯Not performed

Procurement of Samples for Biobanking

Centre ID	
Patient ID	
Visit	
1 x 7,5 ml serum taken?	◯yes ◯no
Sample collection on Date of visit?	◯yes ◯no
Date of collection	____ ______ ________
Time of collection	____ ____ ____
Material	◯complete ◯less material
if no: Reason	____________________
2 x 7,5 ml Lithium-heparin taken?	◯yes ◯no
Sample collection on Date of visit?	◯yes ◯no
Date of collection	____ ______ ________
Time of collection	____ ____ ____
Material	◯complete ◯less material
if no: Reason	____________________

Concomitant medication

Centre ID	
Patient ID	
Visit	
Any new or changed concomitant medication since last visit	◯yes ◯no
If yes, please document all changes in Concomitant medications in the following section:	
Link to Concomitant medication	

	Seq. no.	Drug Name	Indicatio n	Start date	Ongoing at end of study	Stop date

Set Link / Create Entry ...

Concomitant COVID-19 therapy

Centre ID	
Patient ID	
Visit	
Any new Concomitant COVID-19 therapy since last visit?	○yes ○no

If yes, please document all changes in Concomitant COVID-19 therapy in the following section:

Link to Concomitant COVID-19 therapy

	Therapy number	Therapy	Total Daily Dose	Unit	Onset date of medication	Still ongoing	Stop date of therapy

Set Link / Create Entry ...

Adverse events

Centre ID	
Patient ID	
Visit	
Any new Adverse events since last visit?	◯yes ◯no
If yes, please document all new Adverse Events in the following section:	
Link to Adverse Events	(see table below)

	AE number	SOC of AE (according CTCAE V5.0)	AE term (according CTCAE V5.0)	Start date	Ongoing at the end of study	Stop date	Is the AE serious?

Set Link / Create Entry ...

Day 15

Centre ID	
Patient ID	
Visit	Day 15
Date of Visit	____ ______ ________
Hospitalization?	◯yes ◯no

- Vital signs
- Seven point ordinal scale
- Sequential Organ Failure Assessment (SOFA) Score
- Oxygen saturation (SaO2)
- Concomitant medication
- Concomitant COVID-19 therapy
- Adverse events

Investigator's Signature

Meaning:	I confirm the completeness and correctness of all documented data.
Signed By:	
Signature Date:	

Vital signs

Centre ID	
Patient ID	
Visit	
WHO performance status	◯ECOG 0 = Fully active, able to carry on all pre-disease performance without restriction<br>◯ECOG 1 = Restricted in physically strenuous activity but ambulatory and able to carry out work of a light or sedentary nature, e.g., light house work, office work<br>◯ECOG 2 = Ambulatory and capable of all selfcare but unable to carry out any work activities; up and about more than 50% of waking hours<br>◯ECOG 3 = Capable of only limited self-care, confined to bed or chair more than 50% of waking hours<br>◯ECOG 4 = Completely disabled. Cannot carry on any self-care. Totally confined to bed or chair.
Body temperature	____________________ °C
Type of measurement	◯oral ◯tympanic
Systolic blood pressure	____________________ mmHg
Diastolic blood pressure	____________________ mmHg
Pulse rate	____________________ beats/min
respiratory rate	____________________ breaths/min

Seven point ordinal scale

Centre ID	
Patient ID	
Visit	
Time of record	____ ____ ____
Ordinal Scale for Clinical Improvement	◯1 = not hospitalized with resumption of normal activities<br>◯2 = not hospitalized, but unable to resume normal sctivities<br>◯3 = hospitalized,not requiring supplemental oxygen<br>◯4 = hospitalized, requiring supplemental oxygen<br>◯5 = hospitalized, requiring nasal high-flow oxygen therapy or, noninvasive mechanical ventilation<br>◯6 = hospitalized, requiring ECMO, invasive mechanical ventilation, or both<br>◯7 = death

Sequential Organ Failure Assessment (SOFA) Score

Centre ID	
Patient ID	
Visit	
Respiratory system (PaO2/FiO2 (mmHg))	> 400<br>< 400<br>< 300<br>< 200 with respiratoy support<br>< 100 with respiratoy support
Nervous system (Glasgow Coma Scale)	15<br>13-14<br>10-12<br>6-9<br><6
Cardiovascular system (Mean arterial pressure (MAP) or administration of vasopressor required)	MAP > 70 mmHg<br>MAP < 70 mmHg<br>Dopamine 5 µg/kg/min or dobutamine any dose<br>Dopamine > 5 µg/kg/min or epinephrine 0.1 µg/kg/min or norepinephrine 0.1 µg/kg/min<br>Dopamine > 15 µg/kg/min OR epinephrine > 0.1 µg/kg/min OR norepinephrine > 0.1 µg/kg/min
Liver (Bilirubin (mg/dl) [µmol/l])	< 1.2 [< 20]<br>1.2 - 1.9 [20 - 32]<br>2.0 - 5.9 [33 - 101]<br>6.0 - 11.9 [102 - 204]<br>> 12.0 [> 204]
Coagulation (Platelets x103/ml)	> 150<br>100-150<br>50-99<br>20-49<br>< 20
Kidneys (Creatinine (mg/dl) [µmol/L]; urine output)	< 1.2 [< 110]<br>1.2 - 1.9 [110 - 170]<br>2.0 - 3.4 [171 - 299]<br>3. - 4.9 [300 - 440]<br>> 5.0 [> 440]

Oxygen saturation (SaO2)

Centre ID	
Patient ID	
Visit	
ECMO	◯yes ◯no
Type of ventilation	◯no<br>◯Nasal high-flow oxygen therapy<br>◯Noninvasive mechanical ventilation<br>◯Invasive ventilation<br>◯Tracheotomy<br>◯Ambient air<br>◯Standard oxygen sources
Amount of oxygen	____________________ liter/min
Oxygen saturation (SaO2)	____________________ %
Amount of supplemental oxygen that is required to keep SaO2 above 94%	____________________ liter/min
Fraction of Inspired Oxygen (FiO2)	____________________ %
Partial pressure of oxygen (PaO2)	____________________ mmHg
Partial pressure of carbon dioxid (PaCO2)	____________________ mmHg

Concomitant medication

Centre ID		
Patient ID		
Visit		
Any new or changed concomitant medication since last visit		◯yes ◯no
If yes, please document all changes in Concomitant medications in the following section:		
Link to Concomitant medication		

	Seq. no.	Drug Name	Indication	Start date	Ongoing at end of study	Stop date

Set Link / Create Entry ...

Concomitant COVID-19 therapy

Centre ID	
Patient ID	
Visit	
Any new Concomitant COVID-19 therapy since last visit?	◯yes ◯no

If yes, please document all changes in Concomitant COVID-19 therapy in the following section:

Link to Concomitant COVID-19 therapy

	Therapy number	Therapy	Total Daily Dose	Unit	Onset date of medication	Still ongoing	Stop date of therapy

Set Link / Create Entry ...

Adverse events

Centre ID	
Patient ID	
Visit	
Any new Adverse events since last visit?	◯yes ◯no
If yes, please document all new Adverse Events in the following section:	
Link to Adverse Events	(see table below)

	AE number	SOC of AE (according CTCAE V5.0)	AE term (according CTCAE V5.0)	Start date	Ongoing at the end of study	Stop date	Is the AE serious?

Set Link / Create Entry ...

Day 16

Centre ID	
Patient ID	
Visit	Day 16
Date of Visit	____ ______ ________
Hospitalization?	◯yes ◯no

- Vital signs
- Seven point ordinal scale
- Sequential Organ Failure Assessment (SOFA) Score
- Oxygen saturation (SaO2)
- Concomitant medication
- Concomitant COVID-19 therapy
- Adverse events

Investigator's Signature

Meaning:	I confirm the completeness and correctness of all documented data.
Signed By:	
Signature Date:	

Vital signs

Centre ID	
Patient ID	
Visit	
WHO performance status	◯ECOG 0 = Fully active, able to carry on all pre-disease performance without restriction<br>◯ECOG 1 = Restricted in physically strenuous activity but ambulatory and able to carry out work of a light or sedentary nature, e.g., light house work, office work<br>◯ECOG 2 = Ambulatory and capable of all selfcare but unable to carry out any work activities; up and about more than 50% of waking hours<br>◯ECOG 3 = Capable of only limited self-care, confined to bed or chair more than 50% of waking hours<br>◯ECOG 4 = Completely disabled. Cannot carry on any self-care. Totally confined to bed or chair.
Body temperature	____________________ °C
Type of measurement	◯oral ◯tympanic
Systolic blood pressure	____________________ mmHg
Diastolic blood pressure	____________________ mmHg
Pulse rate	____________________ beats/min
respiratory rate	____________________ breaths/min

Seven point ordinal scale

Centre ID	
Patient ID	
Visit	
Time of record	____ ____ ____
Ordinal Scale for Clinical Improvement	◯1 = not hospitalized with resumption of normal activities<br>◯2 = not hospitalized, but unable to resume normal sctivities<br>◯3 = hospitalized,not requiring supplemental oxygen<br>◯4 = hospitalized, requiring supplemental oxygen<br>◯5 = hospitalized, requiring nasal high-flow oxygen therapy or, noninvasive mechanical ventilation<br>◯6 = hospitalized, requiring ECMO, invasive mechanical ventilation, or both<br>◯7 = death

Sequential Organ Failure Assessment (SOFA) Score

Centre ID	
Patient ID	
Visit	
Respiratory system (PaO2/FiO2 (mmHg))	◯> 400<br>◯< 400<br>◯< 300<br>◯< 200 with respiratoy support<br>◯< 100 with respiratoy support
Nervous system (Glasgow Coma Scale)	◯15<br>◯13-14<br>◯10-12<br>◯6-9<br>◯<6
Cardiovascular system (Mean arterial pressure (MAP) or administration of vasopressor required)	◯MAP > 70 mmHg<br>◯MAP < 70 mmHg<br>◯Dopamine 5 µg/kg/min or dobutamine any dose<br>◯Dopamine > 5 µg/kg/min or epinephrine 0.1 µg/kg/min or norepinephrine 0.1 µg/kg/min<br>◯Dopamine > 15 µg/kg/min OR epinephrine > 0.1 µg/kg/min OR norepinephrine > 0.1 µg/kg/min
Liver (Bilirubin (mg/dl) [µmol/l])	◯< 1.2 [< 20]<br>◯1.2 - 1.9 [20 - 32]<br>◯2.0 - 5.9 [33 - 101]<br>◯6.0 - 11.9 [102 - 204]<br>◯> 12.0 [> 204]
Coagulation (Platelets x103/ml)	◯> 150<br>◯100-150<br>◯50-99<br>◯20-49<br>◯< 20
Kidneys (Creatinine (mg/dl) [µmol/L]; urine output)	◯< 1.2 [< 110]<br>◯1.2 - 1.9 [110 - 170]<br>◯2.0 - 3.4 [171 - 299]<br>◯3. - 4.9 [300 - 440]<br>◯> 5.0 [> 440]

Oxygen saturation (SaO2)

Centre ID	
Patient ID	
Visit	
ECMO	◯yes ◯no
Type of ventilation	◯no<br>◯Nasal high-flow oxygen therapy<br>◯Noninvasive mechanical ventilation<br>◯Invasive ventilation<br>◯Tracheotomy<br>◯Ambient air<br>◯Standard oxygen sources
Amount of oxygen	____________________ liter/min
Oxygen saturation (SaO2)	____________________ %
Amount of supplemental oxygen that is required to keep SaO2 above 94%	____________________ liter/min
Fraction of Inspired Oxygen (FiO2)	____________________ %
Partial pressure of oxygen (PaO2)	____________________ mmHg
Partial pressure of carbon dioxid (PaCO2)	____________________ mmHg

Concomitant medication

Centre ID	
Patient ID	
Visit	
Any new or changed concomitant medication since last visit	◯yes ◯no

If yes, please document all changes in Concomitant medications in the following section:

Link to Concomitant medication

	Seq. no.	Drug Name	Indicatio n	Start date	Ongoing at end of study	Stop date

Set Link / Create Entry ...

Concomitant COVID-19 therapy

Centre ID	
Patient ID	
Visit	
Any new Concomitant COVID-19 therapy since last visit?	◯yes ◯no

If yes, please document all changes in Concomitant COVID-19 therapy in the following section:

Link to Concomitant COVID-19 therapy

	Therapy number	Therapy	Total Daily Dose	Unit	Onset date of medication	Still ongoing	Stop date of therapy

Set Link / Create Entry ...

Adverse events

Centre ID	
Patient ID	
Visit	
Any new Adverse events since last visit?	◯yes ◯no

If yes, please document all new Adverse Events in the following section:

Link to Adverse Events

	AE number	SOC of AE (according CTCAE V5.0)	AE term (according CTCAE V5.0)	Start date	Ongoing at the end of study	Stop date	Is the AE serious?

Set Link / Create Entry ...

Day 17

Centre ID	
Patient ID	
Visit	Day 17
Date of Visit	____ ______ ________
Hospitalization?	◯yes ◯no

- Vital signs
- Seven point ordinal scale
- Sequential Organ Failure Assessment (SOFA) Score
- Oxygen saturation (SaO2)
- Hematology
- Blood chemistry and coagulation
- Concomitant medication
- Concomitant COVID-19 therapy
- Adverse events

Investigator's Signature

Meaning:	I confirm the completeness and correctness of all documented data.
Signed By:	
Signature Date:	

Vital signs

Centre ID	
Patient ID	
Visit	
WHO performance status	◯ECOG 0 = Fully active, able to carry on all pre-disease performance without restriction<br>◯ECOG 1 = Restricted in physically strenuous activity but ambulatory and able to carry out work of a light or sedentary nature, e.g., light house work, office work<br>◯ECOG 2 = Ambulatory and capable of all selfcare but unable to carry out any work activities; up and about more than 50% of waking hours<br>◯ECOG 3 = Capable of only limited self-care, confined to bed or chair more than 50% of waking hours<br>◯ECOG 4 = Completely disabled. Cannot carry on any self-care. Totally confined to bed or chair.
Body temperature	____________________ °C
Type of measurement	◯oral ◯tympanic
Systolic blood pressure	____________________ mmHg
Diastolic blood pressure	____________________ mmHg
Pulse rate	____________________ beats/min
respiratory rate	____________________ breaths/min

Seven point ordinal scale

Centre ID	
Patient ID	
Visit	
Time of record	____ ____ ____
Ordinal Scale for Clinical Improvement	◯1 = not hospitalized with resumption of normal activities<br>◯2 = not hospitalized, but unable to resume normal sctivities<br>◯3 = hospitalized,not requiring supplemental oxygen<br>◯4 = hospitalized, requiring supplemental oxygen<br>◯5 = hospitalized, requiring nasal high-flow oxygen therapy or, noninvasive mechanical ventilation<br>◯6 = hospitalized, requiring ECMO, invasive mechanical ventilation, or both<br>◯7 = death

Sequential Organ Failure Assessment (SOFA) Score

Centre ID	
Patient ID	
Visit	
Respiratory system (PaO2/FiO2 (mmHg))	> 400<br>< 400<br>< 300<br>< 200 with respiratoy support<br>< 100 with respiratoy support
Nervous system (Glasgow Coma Scale)	15<br>13-14<br>10-12<br>6-9<br><6
Cardiovascular system (Mean arterial pressure (MAP) or administration of vasopressor required)	MAP > 70 mmHg<br>MAP < 70 mmHg<br>Dopamine 5 µg/kg/min or dobutamine any dose<br>Dopamine > 5 µg/kg/min or epinephrine 0.1 µg/kg/min or norepinephrine 0.1 µg/kg/min<br>Dopamine > 15 µg/kg/min OR epinephrine > 0.1 µg/kg/min OR norepinephrine > 0.1 µg/kg/min
Liver (Bilirubin (mg/dl) [µmol/l])	< 1.2 [< 20]<br>1.2 - 1.9 [20 - 32]<br>2.0 - 5.9 [33 - 101]<br>6.0 - 11.9 [102 - 204]<br>> 12.0 [> 204]
Coagulation (Platelets x103/ml)	> 150<br>100-150<br>50-99<br>20-49<br>< 20
Kidneys (Creatinine (mg/dl) [µmol/L]; urine output)	< 1.2 [< 110]<br>1.2 - 1.9 [110 - 170]<br>2.0 - 3.4 [171 - 299]<br>3. - 4.9 [300 - 440]<br>> 5.0 [> 440]

Oxygen saturation (SaO2)

Centre ID	
Patient ID	
Visit	
ECMO	◯yes ◯no
Type of ventilation	◯no<br>◯Nasal high-flow oxygen therapy<br>◯Noninvasive mechanical ventilation<br>◯Invasive ventilation<br>◯Tracheotomy<br>◯Ambient air<br>◯Standard oxygen sources
Amount of oxygen	____________________ liter/min
Oxygen saturation (SaO2)	____________________ %
Amount of supplemental oxygen that is required to keep SaO2 above 94%	____________________ liter/min
Fraction of Inspired Oxygen (FiO2)	____________________ %
Partial pressure of oxygen (PaO2)	____________________ mmHg
Partial pressure of carbon dioxid (PaCO2)	____________________ mmHg

Hematology

Centre ID	
Patient ID	
Visit	
Date of sample taken	____ _____ ______

Hematology

Parameter	not done	Result	Unit
Hemoglobin	yes		
RBC	yes		
WBC	yes		
Thrombocytes	yes		

Blood chemistry and coagulation

Centre ID	
Patient ID	
Visit	
Date of sample taken	____ _____ _______

Clinical chemistry
<small>Please use a point '.' as decimal separator.</small>

Parameter	not done	Result	Unit
BUN	yes		
Creatinine	yes		
Albumin	yes		
AST/SGOT	yes		
ALT/SGPT	yes		
Total bilirubin	yes		
GGT	yes		
AP	yes		
LDH	yes		
Sodium	yes		
Potassium	yes		
Magnesium	yes		
Calcium	yes		
Uric acid	yes		
Troponin	yes		
CK	yes		U/l
CK-MB	yes		U/l
PTT	yes		sec
ATIII	yes		%
D-Dimer	yes		
Fibrinogen	yes		
Ferritin	yes		
Transferrin	yes		
Transferrin Saturation	yes		%
CRP	yes		
Total protein	yes		g/l
IL6	yes		pg/ml
Procalcitonin	yes		ng/ml
Total IgG	yes		
IgA	yes		
IgM	yes		
Lactate	yes		
INR	yes		

Concomitant medication

Centre ID	
Patient ID	
Visit	
Any new or changed concomitant medication since last visit	◯yes ◯no
If yes, please document all changes in Concomitant medications in the following section:	
Link to Concomitant medication	

	Seq. no.	Drug Name	Indicatio n	Start date	Ongoing at end of study	Stop date

Set Link / Create Entry ...

Concomitant COVID-19 therapy

Centre ID		
Patient ID		
Visit		
Any new Concomitant COVID-19 therapy since last visit?	○yes ○no	

If yes, please document all changes in Concomitant COVID-19 therapy in the following section:

Link to Concomitant COVID-19 therapy

	Therapy number	Therapy	Total Daily Dose	Unit	Onset date of medication	Still ongoing	Stop date of therapy

Set Link / Create Entry ...

Adverse events

Centre ID	
Patient ID	
Visit	
Any new Adverse events since last visit?	◯yes ◯no
If yes, please document all new Adverse Events in the following section:	
Link to Adverse Events	(see table below)

	AE number	SOC of AE (according CTCAE V5.0)	AE term (according CTCAE V5.0)	Start date	Ongoing at the end of study	Stop date	Is the AE serious?

Set Link / Create Entry ...

Day 18

Centre ID	
Patient ID	
Visit	Day 18
Date of Visit	____ ______ ________
Hospitalization?	◯yes ◯no

- Vital signs
- Seven point ordinal scale
- Sequential Organ Failure Assessment (SOFA) Score
- Oxygen saturation (SaO2)
- Concomitant medication
- Concomitant COVID-19 therapy
- Adverse events

Investigator's Signature

Meaning:	I confirm the completeness and correctness of all documented data.
Signed By:	
Signature Date:	

Vital signs

Centre ID	
Patient ID	
Visit	
WHO performance status	◯ECOG 0 = Fully active, able to carry on all pre-disease performance without restriction<br>◯ECOG 1 = Restricted in physically strenuous activity but ambulatory and able to carry out work of a light or sedentary nature, e.g., light house work, office work<br>◯ECOG 2 = Ambulatory and capable of all selfcare but unable to carry out any work activities; up and about more than 50% of waking hours<br>◯ECOG 3 = Capable of only limited self-care, confined to bed or chair more than 50% of waking hours<br>◯ECOG 4 = Completely disabled. Cannot carry on any self-care. Totally confined to bed or chair.
Body temperature	____________________ °C
Type of measurement	◯oral ◯tympanic
Systolic blood pressure	____________________ mmHg
Diastolic blood pressure	____________________ mmHg
Pulse rate	____________________ beats/min
respiratory rate	____________________ breaths/min

Seven point ordinal scale

Centre ID	
Patient ID	
Visit	
Time of record	____ ____ ____
Ordinal Scale for Clinical Improvement	◯1 = not hospitalized with resumption of normal activities<br>◯2 = not hospitalized, but unable to resume normal sctivities<br>◯3 = hospitalized,not requiring supplemental oxygen<br>◯4 = hospitalized, requiring supplemental oxygen<br>◯5 = hospitalized, requiring nasal high-flow oxygen therapy or, noninvasive mechanical ventilation<br>◯6 = hospitalized, requiring ECMO, invasive mechanical ventilation, or both<br>◯7 = death

Sequential Organ Failure Assessment (SOFA) Score

Centre ID	
Patient ID	
Visit	
Respiratory system (PaO2/FiO2 (mmHg))	◯> 400<br>◯< 400<br>◯< 300<br>◯< 200 with respiratoy support<br>◯< 100 with respiratoy support
Nervous system (Glasgow Coma Scale)	◯15<br>◯13-14<br>◯10-12<br>◯6-9<br>◯<6
Cardiovascular system (Mean arterial pressure (MAP) or administration of vasopressor required)	◯MAP > 70 mmHg<br>◯MAP < 70 mmHg<br>◯Dopamine 5 µg/kg/min or dobutamine any dose<br>◯Dopamine > 5 µg/kg/min or epinephrine 0.1 µg/kg/min or norepinephrine 0.1 µg/kg/min<br>◯Dopamine > 15 µg/kg/min OR epinephrine > 0.1 µg/kg/min OR norepinephrine > 0.1 µg/kg/min
Liver (Bilirubin (mg/dl) [µmol/l])	◯< 1.2 [< 20]<br>◯1.2 - 1.9 [20 - 32]<br>◯2.0 - 5.9 [33 - 101]<br>◯6.0 - 11.9 [102 - 204]<br>◯> 12.0 [> 204]
Coagulation (Platelets x103/ml)	◯> 150<br>◯100-150<br>◯50-99<br>◯20-49<br>◯< 20
Kidneys (Creatinine (mg/dl) [µmol/L]; urine output)	◯< 1.2 [< 110]<br>◯1.2 - 1.9 [110 - 170]<br>◯2.0 - 3.4 [171 - 299]<br>◯3. - 4.9 [300 - 440]<br>◯> 5.0 [> 440]

Oxygen saturation (SaO2)

Centre ID	
Patient ID	
Visit	
ECMO	◯yes ◯no
Type of ventilation	◯no<br>◯Nasal high-flow oxygen therapy<br>◯Noninvasive mechanical ventilation<br>◯Invasive ventilation<br>◯Tracheotomy<br>◯Ambient air<br>◯Standard oxygen sources
Amount of oxygen	____________________ liter/min
Oxygen saturation (SaO2)	____________________ %
Amount of supplemental oxygen that is required to keep SaO2 above 94%	____________________ liter/min
Fraction of Inspired Oxygen (FiO2)	____________________ %
Partial pressure of oxygen (PaO2)	____________________ mmHg
Partial pressure of carbon dioxid (PaCO2)	____________________ mmHg

Concomitant medication

Centre ID	
Patient ID	
Visit	
Any new or changed concomitant medication since last visit	◯yes ◯no

If yes, please document all changes in Concomitant medications in the following section:

Link to Concomitant medication

	Seq. no.	Drug Name	Indication	Start date	Ongoing at end of study	Stop date

Set Link / Create Entry ...

Concomitant COVID-19 therapy

Centre ID	
Patient ID	
Visit	
Any new Concomitant COVID-19 therapy since last visit?	○yes ○no
If yes, please document all changes in Concomitant COVID-19 therapy in the following section:	
Link to Concomitant COVID-19 therapy	

	Therapy number	Therapy	Total Daily Dose	Unit	Onset date of medication	Still ongoing	Stop date of therapy

Set Link / Create Entry ...

Adverse events

Centre ID	
Patient ID	
Visit	
Any new Adverse events since last visit?	◯yes ◯no
If yes, please document all new Adverse Events in the following section:	
Link to Adverse Events	see table below

	AE number	SOC of AE (according CTCAE V5.0)	AE term (according CTCAE V5.0)	Start date	Ongoing at the end of study	Stop date	Is the AE serious?

Set Link / Create Entry ...

Day 19

Centre ID	
Patient ID	
Visit	Day 19
Date of Visit	____ ______ ________
Hospitalization?	◯yes ◯no

- Vital signs
- Seven point ordinal scale
- Sequential Organ Failure Assessment (SOFA) Score
- Oxygen saturation (SaO2)
- Concomitant medication
- Concomitant COVID-19 therapy
- Adverse events

Investigator's Signature

Meaning:	I confirm the completeness and correctness of all documented data.
Signed By:	
Signature Date:	

Vital signs

Centre ID	
Patient ID	
Visit	
WHO performance status	◯ECOG 0 = Fully active, able to carry on all pre-disease performance without restriction<br>◯ECOG 1 = Restricted in physically strenuous activity but ambulatory and able to carry out work of a light or sedentary nature, e.g., light house work, office work<br>◯ECOG 2 = Ambulatory and capable of all selfcare but unable to carry out any work activities; up and about more than 50% of waking hours<br>◯ECOG 3 = Capable of only limited self-care, confined to bed or chair more than 50% of waking hours<br>◯ECOG 4 = Completely disabled. Cannot carry on any self-care. Totally confined to bed or chair.
Body temperature	____________________ °C
Type of measurement	◯oral ◯tympanic
Systolic blood pressure	____________________ mmHg
Diastolic blood pressure	____________________ mmHg
Pulse rate	____________________ beats/min
respiratory rate	____________________ breaths/min

Seven point ordinal scale

Centre ID	
Patient ID	
Visit	
Time of record	____ ____ ____
Ordinal Scale for Clinical Improvement	◯1 = not hospitalized with resumption of normal activities<br>◯2 = not hospitalized, but unable to resume normal sctivities<br>◯3 = hospitalized,not requiring supplemental oxygen<br>◯4 = hospitalized, requiring supplemental oxygen<br>◯5 = hospitalized, requiring nasal high-flow oxygen therapy or, noninvasive mechanical ventilation<br>◯6 = hospitalized, requiring ECMO, invasive mechanical ventilation, or both<br>◯7 = death

Sequential Organ Failure Assessment (SOFA) Score

Centre ID	
Patient ID	
Visit	
Respiratory system (PaO2/FiO2 (mmHg))	◯> 400<br>◯< 400<br>◯< 300<br>◯< 200 with respiratoy support<br>◯< 100 with respiratoy support
Nervous system (Glasgow Coma Scale)	◯15<br>◯13-14<br>◯10-12<br>◯6-9<br>◯<6
Cardiovascular system (Mean arterial pressure (MAP) or administration of vasopressor required)	◯MAP > 70 mmHg<br>◯MAP < 70 mmHg<br>◯Dopamine 5 µg/kg/min or dobutamine any dose<br>◯Dopamine > 5 µg/kg/min or epinephrine 0.1 µg/kg/min or norepinephrine 0.1 µg/kg/min<br>◯Dopamine > 15 µg/kg/min OR epinephrine > 0.1 µg/kg/min OR norepinephrine > 0.1 µg/kg/min
Liver (Bilirubin (mg/dl) [µmol/l])	◯< 1.2 [< 20]<br>◯1.2 - 1.9 [20 - 32]<br>◯2.0 - 5.9 [33 - 101]<br>◯6.0 - 11.9 [102 - 204]<br>◯> 12.0 [> 204]
Coagulation (Platelets x103/ml)	◯> 150<br>◯100-150<br>◯50-99<br>◯20-49<br>◯< 20
Kidneys (Creatinine (mg/dl) [µmol/L]; urine output)	◯< 1.2 [< 110]<br>◯1.2 - 1.9 [110 - 170]<br>◯2.0 - 3.4 [171 - 299]<br>◯3. - 4.9 [300 - 440]<br>◯> 5.0 [> 440]

Oxygen saturation (SaO2)

Centre ID	
Patient ID	
Visit	
ECMO	◯yes ◯no
Type of ventilation	◯no<br>◯Nasal high-flow oxygen therapy<br>◯Noninvasive mechanical ventilation<br>◯Invasive ventilation<br>◯Tracheotomy<br>◯Ambient air<br>◯Standard oxygen sources
Amount of oxygen	____________________ liter/min
Oxygen saturation (SaO2)	____________________ %
Amount of supplemental oxygen that is required to keep SaO2 above 94%	____________________ liter/min
Fraction of Inspired Oxygen (FiO2)	____________________ %
Partial pressure of oxygen (PaO2)	____________________ mmHg
Partial pressure of carbon dioxid (PaCO2)	____________________ mmHg

Concomitant medication

Centre ID	
Patient ID	
Visit	
Any new or changed concomitant medication since last visit	○yes ○no
If yes, please document all changes in Concomitant medications in the following section:	
Link to Concomitant medication	

	Seq. no.	Drug Name	Indication	Start date	Ongoing at end of study	Stop date

Set Link / Create Entry ...

Concomitant COVID-19 therapy

Centre ID

Patient ID

Visit

Any new Concomitant COVID-19 therapy since last visit? ○yes ○no

If yes, please document all changes in Concomitant COVID-19 therapy in the following section:

Link to Concomitant COVID-19 therapy

	Therapy number	Therapy	Total Daily Dose	Unit	Onset date of medication	Still ongoing	Stop date of therapy

Set Link / Create Entry ...

Adverse events

Centre ID	
Patient ID	
Visit	
Any new Adverse events since last visit?	◯yes ◯no
If yes, please document all new Adverse Events in the following section:	
Link to Adverse Events	

	AE number	SOC of AE (according CTCAE V5.0)	AE term (according CTCAE V5.0)	Start date	Ongoing at the end of study	Stop date	Is the AE serious?

Set Link / Create Entry ...

Day 20

Centre ID	
Patient ID	
Visit	Day 20
Date of Visit	____ ______ ________
Hospitalization?	◯yes ◯no

- Vital signs
- Seven point ordinal scale
- Sequential Organ Failure Assessment (SOFA) Score
- Oxygen saturation (SaO2)
- Concomitant medication
- Concomitant COVID-19 therapy
- Adverse events

Investigator's Signature

Meaning:	I confirm the completeness and correctness of all documented data.
Signed By:	
Signature Date:	

Vital signs

Centre ID	
Patient ID	
Visit	
WHO performance status	◯ECOG 0 = Fully active, able to carry on all pre-disease performance without restriction<br>◯ECOG 1 = Restricted in physically strenuous activity but ambulatory and able to carry out work of a light or sedentary nature, e.g., light house work, office work<br>◯ECOG 2 = Ambulatory and capable of all selfcare but unable to carry out any work activities; up and about more than 50% of waking hours<br>◯ECOG 3 = Capable of only limited self-care, confined to bed or chair more than 50% of waking hours<br>◯ECOG 4 = Completely disabled. Cannot carry on any self-care. Totally confined to bed or chair.
Body temperature	____________________ °C
Type of measurement	◯oral ◯tympanic
Systolic blood pressure	____________________ mmHg
Diastolic blood pressure	____________________ mmHg
Pulse rate	____________________ beats/min
respiratory rate	____________________ breaths/min

Seven point ordinal scale

Centre ID	
Patient ID	
Visit	
Time of record	____ ____ ____
Ordinal Scale for Clinical Improvement	◯1 = not hospitalized with resumption of normal activities<br>◯2 = not hospitalized, but unable to resume normal sctivities<br>◯3 = hospitalized,not requiring supplemental oxygen<br>◯4 = hospitalized, requiring supplemental oxygen<br>◯5 = hospitalized, requiring nasal high-flow oxygen therapy or, noninvasive mechanical ventilation<br>◯6 = hospitalized, requiring ECMO, invasive mechanical ventilation, or both<br>◯7 = death

Sequential Organ Failure Assessment (SOFA) Score

Centre ID	
Patient ID	
Visit	
Respiratory system (PaO2/FiO2 (mmHg))	◯> 400<br>◯< 400<br>◯< 300<br>◯< 200 with respiratoy support<br>◯< 100 with respiratoy support
Nervous system (Glasgow Coma Scale)	◯15<br>◯13-14<br>◯10-12<br>◯6-9<br>◯<6
Cardiovascular system (Mean arterial pressure (MAP) or administration of vasopressor required)	◯MAP > 70 mmHg<br>◯MAP < 70 mmHg<br>◯Dopamine 5 µg/kg/min or dobutamine any dose<br>◯Dopamine > 5 µg/kg/min or epinephrine 0.1 µg/kg/min or norepinephrine 0.1 µg/kg/min<br>◯Dopamine > 15 µg/kg/min OR epinephrine > 0.1 µg/kg/min OR norepinephrine > 0.1 µg/kg/min
Liver (Bilirubin (mg/dl) [µmol/l])	◯< 1.2 [< 20]<br>◯1.2 - 1.9 [20 - 32]<br>◯2.0 - 5.9 [33 - 101]<br>◯6.0 - 11.9 [102 - 204]<br>◯> 12.0 [> 204]
Coagulation (Platelets x103/ml)	◯> 150<br>◯100-150<br>◯50-99<br>◯20-49<br>◯< 20
Kidneys (Creatinine (mg/dl) [µmol/L]; urine output)	◯< 1.2 [< 110]<br>◯1.2 - 1.9 [110 - 170]<br>◯2.0 - 3.4 [171 - 299]<br>◯3. - 4.9 [300 - 440]<br>◯> 5.0 [> 440]

Oxygen saturation (SaO2)

Centre ID	
Patient ID	
Visit	
ECMO	◯yes ◯no
Type of ventilation	◯no<br>◯Nasal high-flow oxygen therapy<br>◯Noninvasive mechanical ventilation<br>◯Invasive ventilation<br>◯Tracheotomy<br>◯Ambient air<br>◯Standard oxygen sources
Amount of oxygen	____________________ liter/min
Oxygen saturation (SaO2)	____________________ %
Amount of supplemental oxygen that is required to keep SaO2 above 94%	____________________ liter/min
Fraction of Inspired Oxygen (FiO2)	____________________ %
Partial pressure of oxygen (PaO2)	____________________ mmHg
Partial pressure of carbon dioxid (PaCO2)	____________________ mmHg

Concomitant medication

Centre ID	
Patient ID	
Visit	
Any new or changed concomitant medication since last visit	○yes ○no
If yes, please document all changes in Concomitant medications in the following section:	
Link to Concomitant medication	

	Seq. no.	Drug Name	Indication	Start date	Ongoing at end of study	Stop date

Set Link / Create Entry ...

Concomitant COVID-19 therapy

Centre ID	
Patient ID	
Visit	
Any new Concomitant COVID-19 therapy since last visit?	○yes ○no

If yes, please document all changes in Concomitant COVID-19 therapy in the following section:

Link to Concomitant COVID-19 therapy

	Therapy number	Therapy	Total Daily Dose	Unit	Onset date of medication	Still ongoing	Stop date of therapy

Set Link / Create Entry ...

Adverse events

Centre ID	
Patient ID	
Visit	
Any new Adverse events since last visit?	◯yes ◯no

If yes, please document all new Adverse Events in the following section:

Link to Adverse Events

	AE number	SOC of AE (according CTCAE V5.0)	AE term (according CTCAE V5.0)	Start date	Ongoing at the end of study	Stop date	Is the AE serious?

Set Link / Create Entry ...

Day 21

Centre ID	
Patient ID	
Visit	Day 21
Date of Visit	____ ______ ________
Hospitalization?	◯yes ◯no

- Vital signs
- Seven point ordinal scale
- Sequential Organ Failure Assessment (SOFA) Score
- Oxygen saturation (SaO2)
- Hematology
- Blood chemistry and coagulation
- Concomitant medication
- Concomitant COVID-19 therapy
- Adverse events

Investigator's Signature

Meaning:	I confirm the completeness and correctness of all documented data.
Signed By:	
Signature Date:	

Vital signs

Centre ID	
Patient ID	
Visit	
WHO performance status	◯ECOG 0 = Fully active, able to carry on all pre-disease performance without restriction<br>◯ECOG 1 = Restricted in physically strenuous activity but ambulatory and able to carry out work of a light or sedentary nature, e.g., light house work, office work<br>◯ECOG 2 = Ambulatory and capable of all selfcare but unable to carry out any work activities; up and about more than 50% of waking hours<br>◯ECOG 3 = Capable of only limited self-care, confined to bed or chair more than 50% of waking hours<br>◯ECOG 4 = Completely disabled. Cannot carry on any self-care. Totally confined to bed or chair.
Body temperature	____________________ °C
Type of measurement	◯oral ◯tympanic
Systolic blood pressure	____________________ mmHg
Diastolic blood pressure	____________________ mmHg
Pulse rate	____________________ beats/min
respiratory rate	____________________ breaths/min

Seven point ordinal scale

Centre ID	
Patient ID	
Visit	
Time of record	____ ____ ____
Ordinal Scale for Clinical Improvement	◯1 = not hospitalized with resumption of normal activities<br>◯2 = not hospitalized, but unable to resume normal sctivities<br>◯3 = hospitalized,not requiring supplemental oxygen<br>◯4 = hospitalized, requiring supplemental oxygen<br>◯5 = hospitalized, requiring nasal high-flow oxygen therapy or, noninvasive mechanical ventilation<br>◯6 = hospitalized, requiring ECMO, invasive mechanical ventilation, or both<br>◯7 = death

Sequential Organ Failure Assessment (SOFA) Score

Centre ID	
Patient ID	
Visit	
Respiratory system (PaO2/FiO2 (mmHg))	◯> 400<br>◯< 400<br>◯< 300<br>◯< 200 with respiratoy support<br>◯< 100 with respiratoy support
Nervous system (Glasgow Coma Scale)	◯15<br>◯13-14<br>◯10-12<br>◯6-9<br>◯<6
Cardiovascular system (Mean arterial pressure (MAP) or administration of vasopressor required)	◯MAP > 70 mmHg<br>◯MAP < 70 mmHg<br>◯Dopamine 5 µg/kg/min or dobutamine any dose<br>◯Dopamine > 5 µg/kg/min or epinephrine 0.1 µg/kg/min or norepinephrine 0.1 µg/kg/min<br>◯Dopamine > 15 µg/kg/min OR epinephrine > 0.1 µg/kg/min OR norepinephrine > 0.1 µg/kg/min
Liver (Bilirubin (mg/dl) [µmol/l])	◯< 1.2 [< 20]<br>◯1.2 - 1.9 [20 - 32]<br>◯2.0 - 5.9 [33 - 101]<br>◯6.0 - 11.9 [102 - 204]<br>◯> 12.0 [> 204]
Coagulation (Platelets x103/ml)	◯> 150<br>◯100-150<br>◯50-99<br>◯20-49<br>◯< 20
Kidneys (Creatinine (mg/dl) [µmol/L]; urine output)	◯< 1.2 [< 110]<br>◯1.2 - 1.9 [110 - 170]<br>◯2.0 - 3.4 [171 - 299]<br>◯3. - 4.9 [300 - 440]<br>◯> 5.0 [> 440]

Oxygen saturation (SaO2)

Centre ID	
Patient ID	
Visit	
ECMO	◯yes ◯no
Type of ventilation	◯no<br>◯Nasal high-flow oxygen therapy<br>◯Noninvasive mechanical ventilation<br>◯Invasive ventilation<br>◯Tracheotomy<br>◯Ambient air<br>◯Standard oxygen sources
Amount of oxygen	____________________ liter/min
Oxygen saturation (SaO2)	____________________ %
Amount of supplemental oxygen that is required to keep SaO2 above 94%	____________________ liter/min
Fraction of Inspired Oxygen (FiO2)	____________________ %
Partial pressure of oxygen (PaO2)	____________________ mmHg
Partial pressure of carbon dioxid (PaCO2)	____________________ mmHg

Hematology

Centre ID	
Patient ID	
Visit	
Date of sample taken	____ ______ ________

Hematology

Parameter	not done	Result	Unit
Hemoglobin	◯yes		
RBC	◯yes		
WBC	◯yes		
Thrombocytes	◯yes		

Blood chemistry and coagulation

Centre ID	
Patient ID	
Visit	
Date of sample taken	____ ______ ________

Clinical chemistry
<small>Please use a point '.' as decimal separator.</small>

Parameter	not done	Result	Unit
BUN	yes		
Creatinine	yes		
Albumin	yes		
AST/SGOT	yes		
ALT/SGPT	yes		
Total bilirubin	yes		
GGT	yes		
AP	yes		
LDH	yes		
Sodium	yes		
Potassium	yes		
Magnesium	yes		
Calcium	yes		
Uric acid	yes		
Troponin	yes		
CK	yes		U/l
CK-MB	yes		U/l
PTT	yes		sec
ATIII	yes		%
D-Dimer	yes		
Fibrinogen	yes		
Ferritin	yes		
Transferrin	yes		
Transferrin Saturation	yes		%
CRP	yes		
Total protein	yes		g/l
IL6	yes		pg/ml
Procalcitonin	yes		ng/ml
Total IgG	yes		
IgA	yes		
IgM	yes		
Lactate	yes		
INR	yes		

Concomitant medication

Centre ID	
Patient ID	
Visit	
Any new or changed concomitant medication since last visit	◯yes ◯no
If yes, please document all changes in Concomitant medications in the following section:	
Link to Concomitant medication	

	Seq. no.	Drug Name	Indicatio n	Start date	Ongoing at end of study	Stop date

Set Link / Create Entry ...

Concomitant COVID-19 therapy

Centre ID	
Patient ID	
Visit	
Any new Concomitant COVID-19 therapy since last visit?	○yes ○no
If yes, please document all changes in Concomitant COVID-19 therapy in the following section:	
Link to Concomitant COVID-19 therapy	

	Therapy number	Therapy	Total Daily Dose	Unit	Onset date of medication	Still ongoing	Stop date of therapy

Set Link / Create Entry ...

Adverse events

Centre ID	
Patient ID	
Visit	
Any new Adverse events since last visit?	◯yes ◯no
If yes, please document all new Adverse Events in the following section:	
Link to Adverse Events	

	AE number	SOC of AE (according CTCAE V5.0)	AE term (according CTCAE V5.0)	Start date	Ongoing at the end of study	Stop date	Is the AE serious ?

Set Link / Create Entry ...

Day 22

Centre ID	
Patient ID	
Visit	Day 22
Date of Visit	____ ______ ________
Hospitalization?	◯yes ◯no

- Vital signs
- Seven point ordinal scale
- Sequential Organ Failure Assessment (SOFA) Score
- Oxygen saturation (SaO2)
- Concomitant medication
- Concomitant COVID-19 therapy
- Adverse events

Investigator's Signature

Meaning:	I confirm the completeness and correctness of all documented data.
Signed By:	
Signature Date:	

Vital signs

Centre ID	
Patient ID	
Visit	
WHO performance status	◯ECOG 0 = Fully active, able to carry on all pre-disease performance without restriction<br>◯ECOG 1 = Restricted in physically strenuous activity but ambulatory and able to carry out work of a light or sedentary nature, e.g., light house work, office work<br>◯ECOG 2 = Ambulatory and capable of all selfcare but unable to carry out any work activities; up and about more than 50% of waking hours<br>◯ECOG 3 = Capable of only limited self-care, confined to bed or chair more than 50% of waking hours<br>◯ECOG 4 = Completely disabled. Cannot carry on any self-care. Totally confined to bed or chair.
Body temperature	____________________ °C
Type of measurement	◯oral ◯tympanic
Systolic blood pressure	____________________ mmHg
Diastolic blood pressure	____________________ mmHg
Pulse rate	____________________ beats/min
respiratory rate	____________________ breaths/min

Seven point ordinal scale

Centre ID	
Patient ID	
Visit	
Time of record	____ ____ ____
Ordinal Scale for Clinical Improvement	◯1 = not hospitalized with resumption of normal activities<br>◯2 = not hospitalized, but unable to resume normal sctivities<br>◯3 = hospitalized,not requiring supplemental oxygen<br>◯4 = hospitalized, requiring supplemental oxygen<br>◯5 = hospitalized, requiring nasal high-flow oxygen therapy or, noninvasive mechanical ventilation<br>◯6 = hospitalized, requiring ECMO, invasive mechanical ventilation, or both<br>◯7 = death

Sequential Organ Failure Assessment (SOFA) Score

Centre ID	
Patient ID	
Visit	
Respiratory system (PaO2/FiO2 (mmHg))	◯> 400<br>◯< 400<br>◯< 300<br>◯< 200 with respiratoy support<br>◯< 100 with respiratoy support
Nervous system (Glasgow Coma Scale)	◯15<br>◯13-14<br>◯10-12<br>◯6-9<br>◯<6
Cardiovascular system (Mean arterial pressure (MAP) or administration of vasopressor required)	◯MAP > 70 mmHg<br>◯MAP < 70 mmHg<br>◯Dopamine 5 µg/kg/min or dobutamine any dose<br>◯Dopamine > 5 µg/kg/min or epinephrine 0.1 µg/kg/min or norepinephrine 0.1 µg/kg/min<br>◯Dopamine > 15 µg/kg/min OR epinephrine > 0.1 µg/kg/min OR norepinephrine > 0.1 µg/kg/min
Liver (Bilirubin (mg/dl) [µmol/l])	◯< 1.2 [< 20]<br>◯1.2 - 1.9 [20 - 32]<br>◯2.0 - 5.9 [33 - 101]<br>◯6.0 - 11.9 [102 - 204]<br>◯> 12.0 [> 204]
Coagulation (Platelets x103/ml)	◯> 150<br>◯100-150<br>◯50-99<br>◯20-49<br>◯< 20
Kidneys (Creatinine (mg/dl) [µmol/L]; urine output)	◯< 1.2 [< 110]<br>◯1.2 - 1.9 [110 - 170]<br>◯2.0 - 3.4 [171 - 299]<br>◯3. - 4.9 [300 - 440]<br>◯> 5.0 [> 440]

Oxygen saturation (SaO2)

Centre ID	
Patient ID	
Visit	
ECMO	◯yes ◯no
Type of ventilation	◯no<br>◯Nasal high-flow oxygen therapy<br>◯Noninvasive mechanical ventilation<br>◯Invasive ventilation<br>◯Tracheotomy<br>◯Ambient air<br>◯Standard oxygen sources
Amount of oxygen	____________________ liter/min
Oxygen saturation (SaO2)	____________________ %
Amount of supplemental oxygen that is required to keep SaO2 above 94%	____________________ liter/min
Fraction of Inspired Oxygen (FiO2)	____________________ %
Partial pressure of oxygen (PaO2)	____________________ mmHg
Partial pressure of carbon dioxid (PaCO2)	____________________ mmHg

Concomitant medication

Centre ID	
Patient ID	
Visit	
Any new or changed concomitant medication since last visit	◯yes ◯no
If yes, please document all changes in Concomitant medications in the following section:	
Link to Concomitant medication	

	Seq. no.	Drug Name	Indicatio n	Start date	Ongoing at end of study	Stop date

Set Link / Create Entry ...

Concomitant COVID-19 therapy

Centre ID	
Patient ID	
Visit	
Any new Concomitant COVID-19 therapy since last visit?	◯yes ◯no
If yes, please document all changes in Concomitant COVID-19 therapy in the following section:	
Link to Concomitant COVID-19 therapy	

	Therapy number	Therapy	Total Daily Dose	Unit	Onset date of medication	Still ongoing	Stop date of therapy

Set Link / Create Entry ...

Adverse events

Centre ID	
Patient ID	
Visit	
Any new Adverse events since last visit?	◯yes ◯no
If yes, please document all new Adverse Events in the following section:	
Link to Adverse Events	

	AE number	SOC of AE (according CTCAE V5.0)	AE term (according CTCAE V5.0)	Start date	Ongoing at the end of study	Stop date	Is the AE serious?

Set Link / Create Entry ...

Day 23

Centre ID	
Patient ID	
Visit	Day 23
Date of Visit	____ ______ ________
Hospitalization?	◯yes ◯no

- Vital signs
- Seven point ordinal scale
- Sequential Organ Failure Assessment (SOFA) Score
- Oxygen saturation (SaO2)
- Concomitant medication
- Concomitant COVID-19 therapy
- Adverse events

Investigator's Signature

Meaning:	I confirm the completeness and correctness of all documented data.
Signed By:	
Signature Date:	

Vital signs

Centre ID	
Patient ID	
Visit	
WHO performance status	◯ECOG 0 = Fully active, able to carry on all pre-disease performance without restriction<br>◯ECOG 1 = Restricted in physically strenuous activity but ambulatory and able to carry out work of a light or sedentary nature, e.g., light house work, office work<br>◯ECOG 2 = Ambulatory and capable of all selfcare but unable to carry out any work activities; up and about more than 50% of waking hours<br>◯ECOG 3 = Capable of only limited self-care, confined to bed or chair more than 50% of waking hours<br>◯ECOG 4 = Completely disabled. Cannot carry on any self-care. Totally confined to bed or chair.
Body temperature	____________________ °C
Type of measurement	◯oral ◯tympanic
Systolic blood pressure	____________________ mmHg
Diastolic blood pressure	____________________ mmHg
Pulse rate	____________________ beats/min
respiratory rate	____________________ breaths/min

Seven point ordinal scale

Centre ID	
Patient ID	
Visit	
Time of record	____ ____ ____
Ordinal Scale for Clinical Improvement	◯1 = not hospitalized with resumption of normal activities<br>◯2 = not hospitalized, but unable to resume normal sctivities<br>◯3 = hospitalized,not requiring supplemental oxygen<br>◯4 = hospitalized, requiring supplemental oxygen<br>◯5 = hospitalized, requiring nasal high-flow oxygen therapy or, noninvasive mechanical ventilation<br>◯6 = hospitalized, requiring ECMO, invasive mechanical ventilation, or both<br>◯7 = death

Sequential Organ Failure Assessment (SOFA) Score

Centre ID	
Patient ID	
Visit	
Respiratory system (PaO2/FiO2 (mmHg))	◯> 400<br>◯< 400<br>◯< 300<br>◯< 200 with respiratoy support<br>◯< 100 with respiratoy support
Nervous system (Glasgow Coma Scale)	◯15<br>◯13-14<br>◯10-12<br>◯6-9<br>◯<6
Cardiovascular system (Mean arterial pressure (MAP) or administration of vasopressor required)	◯MAP > 70 mmHg<br>◯MAP < 70 mmHg<br>◯Dopamine 5 µg/kg/min or dobutamine any dose<br>◯Dopamine > 5 µg/kg/min or epinephrine 0.1 µg/kg/min or norepinephrine 0.1 µg/kg/min<br>◯Dopamine > 15 µg/kg/min OR epinephrine > 0.1 µg/kg/min OR norepinephrine > 0.1 µg/kg/min
Liver (Bilirubin (mg/dl) [µmol/l])	◯< 1.2 [< 20]<br>◯1.2 - 1.9 [20 - 32]<br>◯2.0 - 5.9 [33 - 101]<br>◯6.0 - 11.9 [102 - 204]<br>◯> 12.0 [> 204]
Coagulation (Platelets x103/ml)	◯> 150<br>◯100-150<br>◯50-99<br>◯20-49<br>◯< 20
Kidneys (Creatinine (mg/dl) [µmol/L]; urine output)	◯< 1.2 [< 110]<br>◯1.2 - 1.9 [110 - 170]<br>◯2.0 - 3.4 [171 - 299]<br>◯3. - 4.9 [300 - 440]<br>◯> 5.0 [> 440]

Oxygen saturation (SaO2)

Centre ID	
Patient ID	
Visit	
ECMO	◯yes ◯no
Type of ventilation	◯no<br>◯Nasal high-flow oxygen therapy<br>◯Noninvasive mechanical ventilation<br>◯Invasive ventilation<br>◯Tracheotomy<br>◯Ambient air<br>◯Standard oxygen sources
Amount of oxygen	____________________ liter/min
Oxygen saturation (SaO2)	____________________ %
Amount of supplemental oxygen that is required to keep SaO2 above 94%	____________________ liter/min
Fraction of Inspired Oxygen (FiO2)	____________________ %
Partial pressure of oxygen (PaO2)	____________________ mmHg
Partial pressure of carbon dioxid (PaCO2)	____________________ mmHg

Concomitant medication

Centre ID	
Patient ID	
Visit	
Any new or changed concomitant medication since last visit	◯yes ◯no
If yes, please document all changes in Concomitant medications in the following section:	
Link to Concomitant medication	

	Seq. no.	Drug Name	Indication	Start date	Ongoing at end of study	Stop date

Set Link / Create Entry ...

Concomitant COVID-19 therapy

Centre ID	
Patient ID	
Visit	
Any new Concomitant COVID-19 therapy since last visit?	○yes ○no
If yes, please document all changes in Concomitant COVID-19 therapy in the following section:	
Link to Concomitant COVID-19 therapy	(see table below)

	Therapy number	Therapy	Total Daily Dose	Unit	Onset date of medication	Still ongoing	Stop date of therapy

Set Link / Create Entry ...

Adverse events

Centre ID	
Patient ID	
Visit	
Any new Adverse events since last visit?	◯yes ◯no
If yes, please document all new Adverse Events in the following section:	
Link to Adverse Events	

	AE number	SOC of AE (according CTCAE V5.0)	AE term (according CTCAE V5.0)	Start date	Ongoing at the end of study	Stop date	Is the AE serious?

Set Link / Create Entry ...

Day 24

Centre ID	
Patient ID	
Visit	Day 24
Date of Visit	____ ______ ________
Hospitalization?	◯yes ◯no

- Vital signs
- Seven point ordinal scale
- Sequential Organ Failure Assessment (SOFA) Score
- Oxygen saturation (SaO2)
- Hematology
- Blood chemistry and coagulation
- Concomitant medication
- Concomitant COVID-19 therapy
- Adverse events

Investigator's Signature

Meaning:	I confirm the completeness and correctness of all documented data.
Signed By:	
Signature Date:	

Vital signs

Centre ID	
Patient ID	
Visit	
WHO performance status	◯ECOG 0 = Fully active, able to carry on all pre-disease performance without restriction<br>◯ECOG 1 = Restricted in physically strenuous activity but ambulatory and able to carry out work of a light or sedentary nature, e.g., light house work, office work<br>◯ECOG 2 = Ambulatory and capable of all selfcare but unable to carry out any work activities; up and about more than 50% of waking hours<br>◯ECOG 3 = Capable of only limited self-care, confined to bed or chair more than 50% of waking hours<br>◯ECOG 4 = Completely disabled. Cannot carry on any self-care. Totally confined to bed or chair.
Body temperature	____________________ °C
Type of measurement	◯oral ◯tympanic
Systolic blood pressure	____________________ mmHg
Diastolic blood pressure	____________________ mmHg
Pulse rate	____________________ beats/min
respiratory rate	____________________ breaths/min

Seven point ordinal scale

Centre ID	
Patient ID	
Visit	
Time of record	____ ____ ____
Ordinal Scale for Clinical Improvement	◯1 = not hospitalized with resumption of normal activities<br>◯2 = not hospitalized, but unable to resume normal sctivities<br>◯3 = hospitalized,not requiring supplemental oxygen<br>◯4 = hospitalized, requiring supplemental oxygen<br>◯5 = hospitalized, requiring nasal high-flow oxygen therapy or, noninvasive mechanical ventilation<br>◯6 = hospitalized, requiring ECMO, invasive mechanical ventilation, or both<br>◯7 = death

Sequential Organ Failure Assessment (SOFA) Score

Centre ID	
Patient ID	
Visit	
Respiratory system (PaO2/FiO2 (mmHg))	◯> 400<br>◯< 400<br>◯< 300<br>◯< 200 with respiratoy support<br>◯< 100 with respiratoy support
Nervous system (Glasgow Coma Scale)	◯15<br>◯13-14<br>◯10-12<br>◯6-9<br>◯<6
Cardiovascular system (Mean arterial pressure (MAP) or administration of vasopressor required)	◯MAP > 70 mmHg<br>◯MAP < 70 mmHg<br>◯Dopamine 5 µg/kg/min or dobutamine any dose<br>◯Dopamine > 5 µg/kg/min or epinephrine 0.1 µg/kg/min or norepinephrine 0.1 µg/kg/min<br>◯Dopamine > 15 µg/kg/min OR epinephrine > 0.1 µg/kg/min OR norepinephrine > 0.1 µg/kg/min
Liver (Bilirubin (mg/dl) [µmol/l])	◯< 1.2 [< 20]<br>◯1.2 - 1.9 [20 - 32]<br>◯2.0 - 5.9 [33 - 101]<br>◯6.0 - 11.9 [102 - 204]<br>◯> 12.0 [> 204]
Coagulation (Platelets x103/ml)	◯> 150<br>◯100-150<br>◯50-99<br>◯20-49<br>◯< 20
Kidneys (Creatinine (mg/dl) [µmol/L]; urine output)	◯< 1.2 [< 110]<br>◯1.2 - 1.9 [110 - 170]<br>◯2.0 - 3.4 [171 - 299]<br>◯3. - 4.9 [300 - 440]<br>◯> 5.0 [> 440]

Oxygen saturation (SaO2)

Centre ID	
Patient ID	
Visit	
ECMO	◯yes ◯no
Type of ventilation	◯no<br>◯Nasal high-flow oxygen therapy<br>◯Noninvasive mechanical ventilation<br>◯Invasive ventilation<br>◯Tracheotomy<br>◯Ambient air<br>◯Standard oxygen sources
Amount of oxygen	____________________ liter/min
Oxygen saturation (SaO2)	____________________ %
Amount of supplemental oxygen that is required to keep SaO2 above 94%	____________________ liter/min
Fraction of Inspired Oxygen (FiO2)	____________________ %
Partial pressure of oxygen (PaO2)	____________________ mmHg
Partial pressure of carbon dioxid (PaCO2)	____________________ mmHg

Hematology

Centre ID	
Patient ID	
Visit	
Date of sample taken	____ _____ _______

Hematology

Parameter	not done	Result	Unit
Hemoglobin	◯yes		▼
RBC	◯yes		▼
WBC	◯yes		▼
Thrombocytes	◯yes		▼

Blood chemistry and coagulation

Centre ID	
Patient ID	
Visit	
Date of sample taken	___ ___ ___

Clinical chemistry
<small>Please use a point '.' as decimal separator.</small>

Parameter	not done	Result	Unit
BUN	yes		
Creatinine	yes		
Albumin	yes		
AST/SGOT	yes		
ALT/SGPT	yes		
Total bilirubin	yes		
GGT	yes		
AP	yes		
LDH	yes		
Sodium	yes		
Potassium	yes		
Magnesium	yes		
Calcium	yes		
Uric acid	yes		
Troponin	yes		
CK	yes		U/l
CK-MB	yes		U/l
PTT	yes		sec
ATIII	yes		%
D-Dimer	yes		
Fibrinogen	yes		
Ferritin	yes		
Transferrin	yes		
Transferrin Saturation	yes		%
CRP	yes		
Total protein	yes		g/l
IL6	yes		pg/ml
Procalcitonin	yes		ng/ml
Total IgG	yes		
IgA	yes		
IgM	yes		
Lactate	yes		
INR	yes		

Concomitant medication

Centre ID	
Patient ID	
Visit	
Any new or changed concomitant medication since last visit	◯yes ◯no
If yes, please document all changes in Concomitant medications in the following section:	
Link to Concomitant medication	

	Seq. no.	Drug Name	Indicatio n	Start date	Ongoing at end of study	Stop date

Set Link / Create Entry ...

Concomitant COVID-19 therapy

Centre ID	
Patient ID	
Visit	
Any new Concomitant COVID-19 therapy since last visit?	○yes ○no

If yes, please document all changes in Concomitant COVID-19 therapy in the following section:

Link to Concomitant COVID-19 therapy

	Therapy number	Therapy	Total Daily Dose	Unit	Onset date of medication	Still ongoing	Stop date of therapy

Set Link / Create Entry ...

Adverse events

Centre ID	
Patient ID	
Visit	
Any new Adverse events since last visit?	◯yes ◯no
If yes, please document all new Adverse Events in the following section:	
Link to Adverse Events	(see table below)

	AE number	SOC of AE (according CTCAE V5.0)	AE term (according CTCAE V5.0)	Start date	Ongoing at the end of study	Stop date	Is the AE serious?

Set Link / Create Entry ...

Day 25

Centre ID	
Patient ID	
Visit	Day 25
Date of Visit	____ ______ ________
Hospitalization?	◯yes ◯no

- Vital signs
- Seven point ordinal scale
- Sequential Organ Failure Assessment (SOFA) Score
- Oxygen saturation (SaO2)
- Concomitant medication
- Concomitant COVID-19 therapy
- Adverse events

Investigator's Signature

Meaning:	I confirm the completeness and correctness of all documented data.
Signed By:	
Signature Date:	

Vital signs

Centre ID	
Patient ID	
Visit	
WHO performance status	◯ECOG 0 = Fully active, able to carry on all pre-disease performance without restriction<br>◯ECOG 1 = Restricted in physically strenuous activity but ambulatory and able to carry out work of a light or sedentary nature, e.g., light house work, office work<br>◯ECOG 2 = Ambulatory and capable of all selfcare but unable to carry out any work activities; up and about more than 50% of waking hours<br>◯ECOG 3 = Capable of only limited self-care, confined to bed or chair more than 50% of waking hours<br>◯ECOG 4 = Completely disabled. Cannot carry on any self-care. Totally confined to bed or chair.
Body temperature	____________________ °C
Type of measurement	◯oral ◯tympanic
Systolic blood pressure	____________________ mmHg
Diastolic blood pressure	____________________ mmHg
Pulse rate	____________________ beats/min
respiratory rate	____________________ breaths/min

Seven point ordinal scale

Centre ID	
Patient ID	
Visit	
Time of record	____ ____ ____
Ordinal Scale for Clinical Improvement	◯1 = not hospitalized with resumption of normal activities<br>◯2 = not hospitalized, but unable to resume normal sctivities<br>◯3 = hospitalized,not requiring supplemental oxygen<br>◯4 = hospitalized, requiring supplemental oxygen<br>◯5 = hospitalized, requiring nasal high-flow oxygen therapy or, noninvasive mechanical ventilation<br>◯6 = hospitalized, requiring ECMO, invasive mechanical ventilation, or both<br>◯7 = death

Sequential Organ Failure Assessment (SOFA) Score

Centre ID	
Patient ID	
Visit	
Respiratory system (PaO2/FiO2 (mmHg))	> 400<br>< 400<br>< 300<br>< 200 with respiratoy support<br>< 100 with respiratoy support
Nervous system (Glasgow Coma Scale)	15<br>13-14<br>10-12<br>6-9<br><6
Cardiovascular system (Mean arterial pressure (MAP) or administration of vasopressor required)	MAP > 70 mmHg<br>MAP < 70 mmHg<br>Dopamine 5 µg/kg/min or dobutamine any dose<br>Dopamine > 5 µg/kg/min or epinephrine 0.1 µg/kg/min or norepinephrine 0.1 µg/kg/min<br>Dopamine > 15 µg/kg/min OR epinephrine > 0.1 µg/kg/min OR norepinephrine > 0.1 µg/kg/min
Liver (Bilirubin (mg/dl) [µmol/l])	< 1.2 [< 20]<br>1.2 - 1.9 [20 - 32]<br>2.0 - 5.9 [33 - 101]<br>6.0 - 11.9 [102 - 204]<br>> 12.0 [> 204]
Coagulation (Platelets x103/ml)	> 150<br>100-150<br>50-99<br>20-49<br>< 20
Kidneys (Creatinine (mg/dl) [µmol/L]; urine output)	< 1.2 [< 110]<br>1.2 - 1.9 [110 - 170]<br>2.0 - 3.4 [171 - 299]<br>3. - 4.9 [300 - 440]<br>> 5.0 [> 440]

Oxygen saturation (SaO2)

Centre ID	
Patient ID	
Visit	
ECMO	◯yes ◯no
Type of ventilation	◯no<br>◯Nasal high-flow oxygen therapy<br>◯Noninvasive mechanical ventilation<br>◯Invasive ventilation<br>◯Tracheotomy<br>◯Ambient air<br>◯Standard oxygen sources
Amount of oxygen	____________________ liter/min
Oxygen saturation (SaO2)	____________________ %
Amount of supplemental oxygen that is required to keep SaO2 above 94%	____________________ liter/min
Fraction of Inspired Oxygen (FiO2)	____________________ %
Partial pressure of oxygen (PaO2)	____________________ mmHg
Partial pressure of carbon dioxid (PaCO2)	____________________ mmHg

Concomitant medication

Centre ID	
Patient ID	
Visit	
Any new or changed concomitant medication since last visit	◯yes ◯no

If yes, please document all changes in Concomitant medications in the following section:

Link to Concomitant medication

	Seq. no.	Drug Name	Indication	Start date	Ongoing at end of study	Stop date

Set Link / Create Entry ...

Concomitant COVID-19 therapy

Centre ID	
Patient ID	
Visit	
Any new Concomitant COVID-19 therapy since last visit?	◯yes ◯no
If yes, please document all changes in Concomitant COVID-19 therapy in the following section:	
Link to Concomitant COVID-19 therapy	

	Therapy number	Therapy	Total Daily Dose	Unit	Onset date of medication	Still ongoing	Stop date of therapy

Set Link / Create Entry ...

Adverse events

Centre ID	
Patient ID	
Visit	
Any new Adverse events since last visit?	◯yes ◯no
If yes, please document all new Adverse Events in the following section:	
Link to Adverse Events	

	AE number	SOC of AE (according CTCAE V5.0)	AE term (according CTCAE V5.0)	Start date	Ongoing at the end of study	Stop date	Is the AE serious?

Set Link / Create Entry ...

Day 26

Centre ID	
Patient ID	
Visit	Day 26
Date of Visit	____ ______ ________
Hospitalization?	◯yes ◯no

Vital signs

Seven point ordinal scale

Sequential Organ Failure Assessment (SOFA) Score

Oxygen saturation (SaO2)

Concomitant medication

Concomitant COVID-19 therapy

Adverse events

Investigator's Signature

Meaning:	I confirm the completeness and correctness of all documented data.
Signed By:	
Signature Date:	

Vital signs

Centre ID	
Patient ID	
Visit	
WHO performance status	◯ECOG 0 = Fully active, able to carry on all pre-disease performance without restriction<br>◯ECOG 1 = Restricted in physically strenuous activity but ambulatory and able to carry out work of a light or sedentary nature, e.g., light house work, office work<br>◯ECOG 2 = Ambulatory and capable of all selfcare but unable to carry out any work activities; up and about more than 50% of waking hours<br>◯ECOG 3 = Capable of only limited self-care, confined to bed or chair more than 50% of waking hours<br>◯ECOG 4 = Completely disabled. Cannot carry on any self-care. Totally confined to bed or chair.
Body temperature	____________________ °C
Type of measurement	◯oral ◯tympanic
Systolic blood pressure	____________________ mmHg
Diastolic blood pressure	____________________ mmHg
Pulse rate	____________________ beats/min
respiratory rate	____________________ breaths/min

Seven point ordinal scale

Centre ID	
Patient ID	
Visit	
Time of record	____ ____ ____
Ordinal Scale for Clinical Improvement	◯1 = not hospitalized with resumption of normal activities<br>◯2 = not hospitalized, but unable to resume normal sctivities<br>◯3 = hospitalized,not requiring supplemental oxygen<br>◯4 = hospitalized, requiring supplemental oxygen<br>◯5 = hospitalized, requiring nasal high-flow oxygen therapy or, noninvasive mechanical ventilation<br>◯6 = hospitalized, requiring ECMO, invasive mechanical ventilation, or both<br>◯7 = death

Sequential Organ Failure Assessment (SOFA) Score

Centre ID	
Patient ID	
Visit	
Respiratory system (PaO2/FiO2 (mmHg))	◯> 400<br>◯< 400<br>◯< 300<br>◯< 200 with respiratoy support<br>◯< 100 with respiratoy support
Nervous system (Glasgow Coma Scale)	◯15<br>◯13-14<br>◯10-12<br>◯6-9<br>◯<6
Cardiovascular system (Mean arterial pressure (MAP) or administration of vasopressor required)	◯MAP > 70 mmHg<br>◯MAP < 70 mmHg<br>◯Dopamine 5 µg/kg/min or dobutamine any dose<br>◯Dopamine > 5 µg/kg/min or epinephrine 0.1 µg/kg/min or norepinephrine 0.1 µg/kg/min<br>◯Dopamine > 15 µg/kg/min OR epinephrine > 0.1 µg/kg/min OR norepinephrine > 0.1 µg/kg/min
Liver (Bilirubin (mg/dl) [µmol/l])	◯< 1.2 [< 20]<br>◯1.2 - 1.9 [20 - 32]<br>◯2.0 - 5.9 [33 - 101]<br>◯6.0 - 11.9 [102 - 204]<br>◯> 12.0 [> 204]
Coagulation (Platelets x103/ml)	◯> 150<br>◯100-150<br>◯50-99<br>◯20-49<br>◯< 20
Kidneys (Creatinine (mg/dl) [µmol/L]; urine output)	◯< 1.2 [< 110]<br>◯1.2 - 1.9 [110 - 170]<br>◯2.0 - 3.4 [171 - 299]<br>◯3. - 4.9 [300 - 440]<br>◯> 5.0 [> 440]

Oxygen saturation (SaO2)

Centre ID	
Patient ID	
Visit	
ECMO	◯yes ◯no
Type of ventilation	◯no<br>◯Nasal high-flow oxygen therapy<br>◯Noninvasive mechanical ventilation<br>◯Invasive ventilation<br>◯Tracheotomy<br>◯Ambient air<br>◯Standard oxygen sources
Amount of oxygen	____________________ liter/min
Oxygen saturation (SaO2)	____________________ %
Amount of supplemental oxygen that is required to keep SaO2 above 94%	____________________ liter/min
Fraction of Inspired Oxygen (FiO2)	____________________ %
Partial pressure of oxygen (PaO2)	____________________ mmHg
Partial pressure of carbon dioxid (PaCO2)	____________________ mmHg

Concomitant medication

Centre ID	
Patient ID	
Visit	
Any new or changed concomitant medication since last visit	◯yes ◯no
If yes, please document all changes in Concomitant medications in the following section:	
Link to Concomitant medication	

	Seq. no.	Drug Name	Indicatio n	Start date	Ongoing at end of study	Stop date

Set Link / Create Entry ...

Concomitant COVID-19 therapy

Centre ID	
Patient ID	
Visit	
Any new Concomitant COVID-19 therapy since last visit?	○yes ○no

If yes, please document all changes in Concomitant COVID-19 therapy in the following section:

Link to Concomitant COVID-19 therapy

	Therapy number	Therapy	Total Daily Dose	Unit	Onset date of medication	Still ongoing	Stop date of therapy

Set Link / Create Entry ...

Adverse events

Centre ID	
Patient ID	
Visit	
Any new Adverse events since last visit?	◯yes ◯no
If yes, please document all new Adverse Events in the following section:	
Link to Adverse Events	

	AE number	SOC of AE (according CTCAE V5.0)	AE term (according CTCAE V5.0)	Start date	Ongoing at the end of study	Stop date	Is the AE serious?

Set Link / Create Entry ...

Day 27

Centre ID	
Patient ID	
Visit	Day 27
Date of Visit	____ ______ ________
Hospitalization?	◯yes ◯no

- Vital signs
- Seven point ordinal scale
- Sequential Organ Failure Assessment (SOFA) Score
- Oxygen saturation (SaO2)
- Concomitant medication
- Concomitant COVID-19 therapy
- Adverse events

Investigator's Signature

Meaning:	I confirm the completeness and correctness of all documented data.
Signed By:	
Signature Date:	

Vital signs

Centre ID	
Patient ID	
Visit	
WHO performance status	◯ECOG 0 = Fully active, able to carry on all pre-disease performance without restriction<br>◯ECOG 1 = Restricted in physically strenuous activity but ambulatory and able to carry out work of a light or sedentary nature, e.g., light house work, office work<br>◯ECOG 2 = Ambulatory and capable of all selfcare but unable to carry out any work activities; up and about more than 50% of waking hours<br>◯ECOG 3 = Capable of only limited self-care, confined to bed or chair more than 50% of waking hours<br>◯ECOG 4 = Completely disabled. Cannot carry on any self-care. Totally confined to bed or chair.
Body temperature	____________________ °C
Type of measurement	◯oral ◯tympanic
Systolic blood pressure	____________________ mmHg
Diastolic blood pressure	____________________ mmHg
Pulse rate	____________________ beats/min
respiratory rate	____________________ breaths/min

Seven point ordinal scale

Centre ID	
Patient ID	
Visit	
Time of record	____ ____ ____
Ordinal Scale for Clinical Improvement	◯1 = not hospitalized with resumption of normal activities<br>◯2 = not hospitalized, but unable to resume normal sctivities<br>◯3 = hospitalized,not requiring supplemental oxygen<br>◯4 = hospitalized, requiring supplemental oxygen<br>◯5 = hospitalized, requiring nasal high-flow oxygen therapy or, noninvasive mechanical ventilation<br>◯6 = hospitalized, requiring ECMO, invasive mechanical ventilation, or both<br>◯7 = death

Sequential Organ Failure Assessment (SOFA) Score

Centre ID	
Patient ID	
Visit	
Respiratory system (PaO2/FiO2 (mmHg))	> 400<br>< 400<br>< 300<br>< 200 with respiratoy support<br>< 100 with respiratoy support
Nervous system (Glasgow Coma Scale)	15<br>13-14<br>10-12<br>6-9<br><6
Cardiovascular system (Mean arterial pressure (MAP) or administration of vasopressor required)	MAP > 70 mmHg<br>MAP < 70 mmHg<br>Dopamine 5 µg/kg/min or dobutamine any dose<br>Dopamine > 5 µg/kg/min or epinephrine 0.1 µg/kg/min or norepinephrine 0.1 µg/kg/min<br>Dopamine > 15 µg/kg/min OR epinephrine > 0.1 µg/kg/min OR norepinephrine > 0.1 µg/kg/min
Liver (Bilirubin (mg/dl) [µmol/l])	< 1.2 [< 20]<br>1.2 - 1.9 [20 - 32]<br>2.0 - 5.9 [33 - 101]<br>6.0 - 11.9 [102 - 204]<br>> 12.0 [> 204]
Coagulation (Platelets x103/ml)	> 150<br>100-150<br>50-99<br>20-49<br>< 20
Kidneys (Creatinine (mg/dl) [µmol/L]; urine output)	< 1.2 [< 110]<br>1.2 - 1.9 [110 - 170]<br>2.0 - 3.4 [171 - 299]<br>3. - 4.9 [300 - 440]<br>> 5.0 [> 440]

Oxygen saturation (SaO2)

Centre ID	
Patient ID	
Visit	
ECMO	◯yes ◯no
Type of ventilation	◯no<br>◯Nasal high-flow oxygen therapy<br>◯Noninvasive mechanical ventilation<br>◯Invasive ventilation<br>◯Tracheotomy<br>◯Ambient air<br>◯Standard oxygen sources
Amount of oxygen	____________________ liter/min
Oxygen saturation (SaO2)	____________________ %
Amount of supplemental oxygen that is required to keep SaO2 above 94%	____________________ liter/min
Fraction of Inspired Oxygen (FiO2)	____________________ %
Partial pressure of oxygen (PaO2)	____________________ mmHg
Partial pressure of carbon dioxid (PaCO2)	____________________ mmHg

Concomitant medication

Centre ID	
Patient ID	
Visit	
Any new or changed concomitant medication since last visit	◯yes ◯no
If yes, please document all changes in Concomitant medications in the following section:	
Link to Concomitant medication	

	Seq. no.	Drug Name	Indication	Start date	Ongoing at end of study	Stop date

Set Link / Create Entry ...

Concomitant COVID-19 therapy

Centre ID	
Patient ID	
Visit	
Any new Concomitant COVID-19 therapy since last visit?	◯yes ◯no
If yes, please document all changes in Concomitant COVID-19 therapy in the following section:	
Link to Concomitant COVID-19 therapy	

	Therapy number	Therapy	Total Daily Dose	Unit	Onset date of medication	Still ongoing	Stop date of therapy

Set Link / Create Entry ...

Adverse events

Centre ID	
Patient ID	
Visit	
Any new Adverse events since last visit?	◯yes ◯no
If yes, please document all new Adverse Events in the following section:	
Link to Adverse Events	(see table below)

	AE number	SOC of AE (according CTCAE V5.0)	AE term (according CTCAE V5.0)	Start date	Ongoing at the end of study	Stop date	Is the AE serious?

Set Link / Create Entry ...

Day 28

Centre ID	
Patient ID	
Visit	Day 28
Date of Visit	____ ______ ________
Hospitalization?	◯yes ◯no

- Vital signs
- Seven point ordinal scale
- Sequential Organ Failure Assessment (SOFA) Score
- Oxygen saturation (SaO2)
- Hematology
- Blood chemistry and coagulation
- SARS-CoV-2 viral clearance and load as well as antibody titres
- Procurement of Samples for Biobanking
- Concomitant medication
- Concomitant COVID-19 therapy
- Adverse events

Investigator's Signature

Meaning:	I confirm the completeness and correctness of all documented data.
Signed By:	
Signature Date:	

Vital signs

Centre ID	
Patient ID	
Visit	
WHO performance status	◯ECOG 0 = Fully active, able to carry on all pre-disease performance without restriction<br>◯ECOG 1 = Restricted in physically strenuous activity but ambulatory and able to carry out work of a light or sedentary nature, e.g., light house work, office work<br>◯ECOG 2 = Ambulatory and capable of all selfcare but unable to carry out any work activities; up and about more than 50% of waking hours<br>◯ECOG 3 = Capable of only limited self-care, confined to bed or chair more than 50% of waking hours<br>◯ECOG 4 = Completely disabled. Cannot carry on any self-care. Totally confined to bed or chair.
Body temperature	____________________ °C
Type of measurement	◯oral ◯tympanic
Systolic blood pressure	____________________ mmHg
Diastolic blood pressure	____________________ mmHg
Pulse rate	____________________ beats/min
respiratory rate	____________________ breaths/min

Seven point ordinal scale

Centre ID	
Patient ID	
Visit	
Time of record	____ ____ ____
Ordinal Scale for Clinical Improvement	◯1 = not hospitalized with resumption of normal activities<br>◯2 = not hospitalized, but unable to resume normal sctivities<br>◯3 = hospitalized,not requiring supplemental oxygen<br>◯4 = hospitalized, requiring supplemental oxygen<br>◯5 = hospitalized, requiring nasal high-flow oxygen therapy or, noninvasive mechanical ventilation<br>◯6 = hospitalized, requiring ECMO, invasive mechanical ventilation, or both<br>◯7 = death

Sequential Organ Failure Assessment (SOFA) Score

Centre ID	
Patient ID	
Visit	
Respiratory system (PaO2/FiO2 (mmHg))	◯> 400<br>◯< 400<br>◯< 300<br>◯< 200 with respiratoy support<br>◯< 100 with respiratoy support
Nervous system (Glasgow Coma Scale)	◯15<br>◯13-14<br>◯10-12<br>◯6-9<br>◯<6
Cardiovascular system (Mean arterial pressure (MAP) or administration of vasopressor required)	◯MAP > 70 mmHg<br>◯MAP < 70 mmHg<br>◯Dopamine 5 µg/kg/min or dobutamine any dose<br>◯Dopamine > 5 µg/kg/min or epinephrine 0.1 µg/kg/min or norepinephrine 0.1 µg/kg/min<br>◯Dopamine > 15 µg/kg/min OR epinephrine > 0.1 µg/kg/min OR norepinephrine > 0.1 µg/kg/min
Liver (Bilirubin (mg/dl) [µmol/l])	◯< 1.2 [< 20]<br>◯1.2 - 1.9 [20 - 32]<br>◯2.0 - 5.9 [33 - 101]<br>◯6.0 - 11.9 [102 - 204]<br>◯> 12.0 [> 204]
Coagulation (Platelets x103/ml)	◯> 150<br>◯100-150<br>◯50-99<br>◯20-49<br>◯< 20
Kidneys (Creatinine (mg/dl) [µmol/L]; urine output)	◯< 1.2 [< 110]<br>◯1.2 - 1.9 [110 - 170]<br>◯2.0 - 3.4 [171 - 299]<br>◯3. - 4.9 [300 - 440]<br>◯> 5.0 [> 440]

Oxygen saturation (SaO2)

Centre ID	
Patient ID	
Visit	
ECMO	◯yes ◯no
Type of ventilation	◯no<br>◯Nasal high-flow oxygen therapy<br>◯Noninvasive mechanical ventilation<br>◯Invasive ventilation<br>◯Tracheotomy<br>◯Ambient air<br>◯Standard oxygen sources
Amount of oxygen	____________________ liter/min
Oxygen saturation (SaO2)	____________________ %
Amount of supplemental oxygen that is required to keep SaO2 above 94%	____________________ liter/min
Fraction of Inspired Oxygen (FiO2)	____________________ %
Partial pressure of oxygen (PaO2)	____________________ mmHg
Partial pressure of carbon dioxid (PaCO2)	____________________ mmHg

Hematology

Centre ID	
Patient ID	
Visit	
Date of sample taken	____ ______ ________

Hematology

Parameter	not done	Result	Unit
Hemoglobin	◯yes		
RBC	◯yes		
WBC	◯yes		
Thrombocytes	◯yes		

Blood chemistry and coagulation

Centre ID	
Patient ID	
Visit	
Date of sample taken	____ ______ ________

Clinical chemistry
<small>Please use a point '.' as decimal separator.</small>

Parameter	not done	Result	Unit
BUN	◯yes		
Creatinine	◯yes		
Albumin	◯yes		
AST/SGOT	◯yes		
ALT/SGPT	◯yes		
Total bilirubin	◯yes		
GGT	◯yes		
AP	◯yes		
LDH	◯yes		
Sodium	◯yes		
Potassium	◯yes		
Magnesium	◯yes		
Calcium	◯yes		
Uric acid	◯yes		
Troponin	◯yes		
CK	◯yes		U/l
CK-MB	◯yes		U/l
PTT	◯yes		sec
ATIII	◯yes		%
D-Dimer	◯yes		
Fibrinogen	◯yes		
Ferritin	◯yes		
Transferrin	◯yes		
Transferrin Saturation	◯yes		%
CRP	◯yes		
Total protein	◯yes		g/l
IL6	◯yes		pg/ml
Procalcitonin	◯yes		ng/ml
Total IgG	◯yes		
IgA	◯yes		
IgM	◯yes		
Lactate	◯yes		
INR	◯yes		

SARS-CoV-2 viral clearance and load as well as antibody titres

Centre ID	
Patient ID	
Visit	
Sample collection on Date of visit?	◯yes ◯no
Date of sample collection	____ ______ ________
Type of smear	◯Nasopharyngeal<br>◯Oropharyngeal<br>◯Sputum<br>◯Other
If other: Please specify	____________________
Result of smear	◯Negative ◯Positive ◯Invalide
PCR test type	◯TibMolbiol<br>◯Seegene<br>◯Abbott<br>◯Altona<br>◯Other
If other: Please specify	____________________
CT-value	____________________
Serodiagnostic by ELISA (OD Ratio)	____________________
Neutralize antibody titre (1:....)	____________________ ▾
Immunofluorescence	◯Positive<br>◯Borderline positive<br>◯Negative<br>◯Not performed

Procurement of Samples for Biobanking

Centre ID	
Patient ID	
Visit	
1 x 7,5 ml serum taken?	◯yes ◯no
Sample collection on Date of visit?	◯yes ◯no
Date of collection	____ ______ ________
Time of collection	____ ____ ____
Material	◯complete ◯less material
if no: Reason	____________________
2 x 7,5 ml Lithium-heparin taken?	◯yes ◯no
Sample collection on Date of visit?	◯yes ◯no
Date of collection	____ ______ ________
Time of collection	____ ____ ____
Material	◯complete ◯less material
if no: Reason	____________________

Concomitant medication

Centre ID

Patient ID

Visit

Any new or changed concomitant medication since last visit ◯yes ◯no

If yes, please document all changes in Concomitant medications in the following section:

Link to Concomitant medication

	Seq. no.	Drug Name	Indication	Start date	Ongoing at end of study	Stop date

Set Link / Create Entry ...

Concomitant COVID-19 therapy

Centre ID	
Patient ID	
Visit	
Any new Concomitant COVID-19 therapy since last visit?	◯yes ◯no
If yes, please document all changes in Concomitant COVID-19 therapy in the following section:	
Link to Concomitant COVID-19 therapy	

	Therapy number	Therapy	Total Daily Dose	Unit	Onset date of medication	Still ongoing	Stop date of therapy

Set Link / Create Entry ...

Adverse events

Centre ID	
Patient ID	
Visit	
Any new Adverse events since last visit?	◯yes ◯no
If yes, please document all new Adverse Events in the following section:	
Link to Adverse Events	(see table below)

	AE number	SOC of AE (according CTCAE V5.0)	AE term (according CTCAE V5.0)	Start date	Ongoing at the end of study	Stop date	Is the AE serious?

Set Link / Create Entry ...

Day 35

Centre ID	
Patient ID	
Visit	Day 35
Date of Visit	____ ______ ________
Hospitalization?	◯yes ◯no

Vital signs

Seven point ordinal scale

Sequential Organ Failure Assessment (SOFA) Score

Oxygen saturation (SaO2)

Hematology

Blood chemistry and coagulation

Concomitant medication

Concomitant COVID-19 therapy

Adverse events

Investigator's Signature

Meaning:	I confirm the completeness and correctness of all documented data.
Signed By:	
Signature Date:	

Vital signs

Centre ID	
Patient ID	
Visit	
WHO performance status	◯ECOG 0 = Fully active, able to carry on all pre-disease performance without restriction<br>◯ECOG 1 = Restricted in physically strenuous activity but ambulatory and able to carry out work of a light or sedentary nature, e.g., light house work, office work<br>◯ECOG 2 = Ambulatory and capable of all selfcare but unable to carry out any work activities; up and about more than 50% of waking hours<br>◯ECOG 3 = Capable of only limited self-care, confined to bed or chair more than 50% of waking hours<br>◯ECOG 4 = Completely disabled. Cannot carry on any self-care. Totally confined to bed or chair.
Body temperature	____________________ °C
Type of measurement	◯oral ◯tympanic
Systolic blood pressure	____________________ mmHg
Diastolic blood pressure	____________________ mmHg
Pulse rate	____________________ beats/min
respiratory rate	____________________ breaths/min

Seven point ordinal scale

Centre ID	
Patient ID	
Visit	
Time of record	____ ____ ____
Ordinal Scale for Clinical Improvement	◯1 = not hospitalized with resumption of normal activities<br>◯2 = not hospitalized, but unable to resume normal sctivities<br>◯3 = hospitalized,not requiring supplemental oxygen<br>◯4 = hospitalized, requiring supplemental oxygen<br>◯5 = hospitalized, requiring nasal high-flow oxygen therapy or, noninvasive mechanical ventilation<br>◯6 = hospitalized, requiring ECMO, invasive mechanical ventilation, or both<br>◯7 = death

Sequential Organ Failure Assessment (SOFA) Score

Centre ID	
Patient ID	
Visit	
Respiratory system (PaO2/FiO2 (mmHg))	> 400<br>< 400<br>< 300<br>< 200 with respiratoy support<br>< 100 with respiratoy support
Nervous system (Glasgow Coma Scale)	15<br>13-14<br>10-12<br>6-9<br><6
Cardiovascular system (Mean arterial pressure (MAP) or administration of vasopressor required)	MAP > 70 mmHg<br>MAP < 70 mmHg<br>Dopamine 5 µg/kg/min or dobutamine any dose<br>Dopamine > 5 µg/kg/min or epinephrine 0.1 µg/kg/min or norepinephrine 0.1 µg/kg/min<br>Dopamine > 15 µg/kg/min OR epinephrine > 0.1 µg/kg/min OR norepinephrine > 0.1 µg/kg/min
Liver (Bilirubin (mg/dl) [µmol/l])	< 1.2 [< 20]<br>1.2 - 1.9 [20 - 32]<br>2.0 - 5.9 [33 - 101]<br>6.0 - 11.9 [102 - 204]<br>> 12.0 [> 204]
Coagulation (Platelets x103/ml)	> 150<br>100-150<br>50-99<br>20-49<br>< 20
Kidneys (Creatinine (mg/dl) [µmol/L]; urine output)	< 1.2 [< 110]<br>1.2 - 1.9 [110 - 170]<br>2.0 - 3.4 [171 - 299]<br>3. - 4.9 [300 - 440]<br>> 5.0 [> 440]

Oxygen saturation (SaO2)

Centre ID	
Patient ID	
Visit	
ECMO	◯yes ◯no
Type of ventilation	◯no<br>◯Nasal high-flow oxygen therapy<br>◯Noninvasive mechanical ventilation<br>◯Invasive ventilation<br>◯Tracheotomy<br>◯Ambient air<br>◯Standard oxygen sources
Amount of oxygen	____________________ liter/min
Oxygen saturation (SaO2)	____________________ %
Amount of supplemental oxygen that is required to keep SaO2 above 94%	____________________ liter/min
Fraction of Inspired Oxygen (FiO2)	____________________ %
Partial pressure of oxygen (PaO2)	____________________ mmHg
Partial pressure of carbon dioxid (PaCO2)	____________________ mmHg

Hematology

Centre ID	
Patient ID	
Visit	
Date of sample taken	____ ______ ________

Hematology

Parameter	not done	Result	Unit
Hemoglobin	◯yes		
RBC	◯yes		
WBC	◯yes		
Thrombocytes	◯yes		

Blood chemistry and coagulation

Centre ID	
Patient ID	
Visit	
Date of sample taken	____ _____ _______

Clinical chemistry

<small>Please use a point '.' as decimal separator.</small>

Parameter	not done	Result	Unit
BUN	yes		
Creatinine	yes		
Albumin	yes		
AST/SGOT	yes		
ALT/SGPT	yes		
Total bilirubin	yes		
GGT	yes		
AP	yes		
LDH	yes		
Sodium	yes		
Potassium	yes		
Magnesium	yes		
Calcium	yes		
Uric acid	yes		
Troponin	yes		
CK	yes		U/l
CK-MB	yes		U/l
PTT	yes		sec
ATIII	yes		%
D-Dimer	yes		
Fibrinogen	yes		
Ferritin	yes		
Transferrin	yes		
Transferrin Saturation	yes		%
CRP	yes		
Total protein	yes		g/l
IL6	yes		pg/ml
Procalcitonin	yes		ng/ml
Total IgG	yes		
IgA	yes		
IgM	yes		
Lactate	yes		
INR	yes		

Concomitant medication

Centre ID		
Patient ID		
Visit		
Any new or changed concomitant medication since last visit		◯yes ◯no
If yes, please document all changes in Concomitant medications in the following section:		
Link to Concomitant medication		

	Seq. no.	Drug Name	Indication	Start date	Ongoing at end of study	Stop date

Set Link / Create Entry ...

Concomitant COVID-19 therapy

Centre ID	
Patient ID	
Visit	
Any new Concomitant COVID-19 therapy since last visit?	◯yes ◯no
If yes, please document all changes in Concomitant COVID-19 therapy in the following section:	
Link to Concomitant COVID-19 therapy	

	Therapy number	Therapy	Total Daily Dose	Unit	Onset date of medication	Still ongoing	Stop date of therapy

Set Link / Create Entry ...

Adverse events

Centre ID	
Patient ID	
Visit	
Any new Adverse events since last visit?	◯yes ◯no
If yes, please document all new Adverse Events in the following section:	
Link to Adverse Events	

	AE number	SOC of AE (according CTCAE V5.0)	AE term (according CTCAE V5.0)	Start date	Ongoing at the end of study	Stop date	Is the AE serious?

Set Link / Create Entry ...

Day 42

Centre ID	
Patient ID	
Visit	Day 42
Date of Visit	____ ______ ________
Hospitalization?	◯yes ◯no

- Vital signs
- Seven point ordinal scale
- Sequential Organ Failure Assessment (SOFA) Score
- Oxygen saturation (SaO2)
- Hematology
- Blood chemistry and coagulation
- Concomitant medication
- Concomitant COVID-19 therapy
- Adverse events

Investigator's Signature

Meaning:	I confirm the completeness and correctness of all documented data.
Signed By:	
Signature Date:	

Vital signs

Centre ID	
Patient ID	
Visit	
WHO performance status	◯ECOG 0 = Fully active, able to carry on all pre-disease performance without restriction<br>◯ECOG 1 = Restricted in physically strenuous activity but ambulatory and able to carry out work of a light or sedentary nature, e.g., light house work, office work<br>◯ECOG 2 = Ambulatory and capable of all selfcare but unable to carry out any work activities; up and about more than 50% of waking hours<br>◯ECOG 3 = Capable of only limited self-care, confined to bed or chair more than 50% of waking hours<br>◯ECOG 4 = Completely disabled. Cannot carry on any self-care. Totally confined to bed or chair.
Body temperature	____________________ °C
Type of measurement	◯oral ◯tympanic
Systolic blood pressure	____________________ mmHg
Diastolic blood pressure	____________________ mmHg
Pulse rate	____________________ beats/min
respiratory rate	____________________ breaths/min

Seven point ordinal scale

Centre ID	
Patient ID	
Visit	
Time of record	____ ____ ____
Ordinal Scale for Clinical Improvement	◯1 = not hospitalized with resumption of normal activities<br>◯2 = not hospitalized, but unable to resume normal sctivities<br>◯3 = hospitalized,not requiring supplemental oxygen<br>◯4 = hospitalized, requiring supplemental oxygen<br>◯5 = hospitalized, requiring nasal high-flow oxygen therapy or, noninvasive mechanical ventilation<br>◯6 = hospitalized, requiring ECMO, invasive mechanical ventilation, or both<br>◯7 = death

Sequential Organ Failure Assessment (SOFA) Score

Centre ID	
Patient ID	
Visit	
Respiratory system (PaO2/FiO2 (mmHg))	> 400<br>< 400<br>< 300<br>< 200 with respiratoy support<br>< 100 with respiratoy support
Nervous system (Glasgow Coma Scale)	15<br>13-14<br>10-12<br>6-9<br><6
Cardiovascular system (Mean arterial pressure (MAP) or administration of vasopressor required)	MAP > 70 mmHg<br>MAP < 70 mmHg<br>Dopamine 5 µg/kg/min or dobutamine any dose<br>Dopamine > 5 µg/kg/min or epinephrine 0.1 µg/kg/min or norepinephrine 0.1 µg/kg/min<br>Dopamine > 15 µg/kg/min OR epinephrine > 0.1 µg/kg/min OR norepinephrine > 0.1 µg/kg/min
Liver (Bilirubin (mg/dl) [µmol/l])	< 1.2 [< 20]<br>1.2 - 1.9 [20 - 32]<br>2.0 - 5.9 [33 - 101]<br>6.0 - 11.9 [102 - 204]<br>> 12.0 [> 204]
Coagulation (Platelets x103/ml)	> 150<br>100-150<br>50-99<br>20-49<br>< 20
Kidneys (Creatinine (mg/dl) [µmol/L]; urine output)	< 1.2 [< 110]<br>1.2 - 1.9 [110 - 170]<br>2.0 - 3.4 [171 - 299]<br>3. - 4.9 [300 - 440]<br>> 5.0 [> 440]

Oxygen saturation (SaO2)

Centre ID	
Patient ID	
Visit	
ECMO	◯yes ◯no
Type of ventilation	◯no<br>◯Nasal high-flow oxygen therapy<br>◯Noninvasive mechanical ventilation<br>◯Invasive ventilation<br>◯Tracheotomy<br>◯Ambient air<br>◯Standard oxygen sources
Amount of oxygen	____________________ liter/min
Oxygen saturation (SaO2)	____________________ %
Amount of supplemental oxygen that is required to keep SaO2 above 94%	____________________ liter/min
Fraction of Inspired Oxygen (FiO2)	____________________ %
Partial pressure of oxygen (PaO2)	____________________ mmHg
Partial pressure of carbon dioxid (PaCO2)	____________________ mmHg

Hematology

Centre ID	
Patient ID	
Visit	
Date of sample taken	____ ______ ________

Hematology

Parameter	not done	Result	Unit
Hemoglobin	◯yes		
RBC	◯yes		
WBC	◯yes		
Thrombocytes	◯yes		

Blood chemistry and coagulation

Centre ID	
Patient ID	
Visit	
Date of sample taken	____ _____ ______

Clinical chemistry
<small>Please use a point '.' as decimal separator.</small>

Parameter	not done	Result	Unit
BUN	yes		
Creatinine	yes		
Albumin	yes		
AST/SGOT	yes		
ALT/SGPT	yes		
Total bilirubin	yes		
GGT	yes		
AP	yes		
LDH	yes		
Sodium	yes		
Potassium	yes		
Magnesium	yes		
Calcium	yes		
Uric acid	yes		
Troponin	yes		
CK	yes		U/l
CK-MB	yes		U/l
PTT	yes		sec
ATIII	yes		%
D-Dimer	yes		
Fibrinogen	yes		
Ferritin	yes		
Transferrin	yes		
Transferrin Saturation	yes		%
CRP	yes		
Total protein	yes		g/l
IL6	yes		pg/ml
Procalcitonin	yes		ng/ml
Total IgG	yes		
IgA	yes		
IgM	yes		
Lactate	yes		
INR	yes		

Concomitant medication

Centre ID	
Patient ID	
Visit	
Any new or changed concomitant medication since last visit	◯yes ◯no

If yes, please document all changes in Concomitant medications in the following section:

Link to Concomitant medication

	Seq. no.	Drug Name	Indicatio n	Start date	Ongoing at end of study	Stop date

Set Link / Create Entry ...

Concomitant COVID-19 therapy

Centre ID	
Patient ID	
Visit	
Any new Concomitant COVID-19 therapy since last visit?	◯yes ◯no
If yes, please document all changes in Concomitant COVID-19 therapy in the following section:	
Link to Concomitant COVID-19 therapy	see table below

	Therapy number	Therapy	Total Daily Dose	Unit	Onset date of medication	Still ongoing	Stop date of therapy

Set Link / Create Entry ...

Adverse events

Centre ID	
Patient ID	
Visit	
Any new Adverse events since last visit?	◯yes ◯no
If yes, please document all new Adverse Events in the following section:	
Link to Adverse Events	(see table below)

	AE number	SOC of AE (according CTCAE V5.0)	AE term (according CTCAE V5.0)	Start date	Ongoing at the end of study	Stop date	Is the AE serious?

Set Link / Create Entry ...

Day 49

Centre ID	
Patient ID	
Visit	Day 49
Date of Visit	____ ______ ________
Hospitalization?	◯yes ◯no

- Vital signs
- Seven point ordinal scale
- Sequential Organ Failure Assessment (SOFA) Score
- Oxygen saturation (SaO2)
- Hematology
- Blood chemistry and coagulation
- Concomitant medication
- Concomitant COVID-19 therapy
- Adverse events

Investigator's Signature

Meaning:	I confirm the completeness and correctness of all documented data.
Signed By:	
Signature Date:	

Vital signs

Centre ID	
Patient ID	
Visit	
WHO performance status	◯ECOG 0 = Fully active, able to carry on all pre-disease performance without restriction<br>◯ECOG 1 = Restricted in physically strenuous activity but ambulatory and able to carry out work of a light or sedentary nature, e.g., light house work, office work<br>◯ECOG 2 = Ambulatory and capable of all selfcare but unable to carry out any work activities; up and about more than 50% of waking hours<br>◯ECOG 3 = Capable of only limited self-care, confined to bed or chair more than 50% of waking hours<br>◯ECOG 4 = Completely disabled. Cannot carry on any self-care. Totally confined to bed or chair.
Body temperature	____________________ °C
Type of measurement	◯oral ◯tympanic
Systolic blood pressure	____________________ mmHg
Diastolic blood pressure	____________________ mmHg
Pulse rate	____________________ beats/min
respiratory rate	____________________ breaths/min

Seven point ordinal scale

Centre ID	
Patient ID	
Visit	
Time of record	____ ____ ____
Ordinal Scale for Clinical Improvement	◯1 = not hospitalized with resumption of normal activities<br>◯2 = not hospitalized, but unable to resume normal sctivities<br>◯3 = hospitalized,not requiring supplemental oxygen<br>◯4 = hospitalized, requiring supplemental oxygen<br>◯5 = hospitalized, requiring nasal high-flow oxygen therapy or, noninvasive mechanical ventilation<br>◯6 = hospitalized, requiring ECMO, invasive mechanical ventilation, or both<br>◯7 = death

Sequential Organ Failure Assessment (SOFA) Score

Centre ID	
Patient ID	
Visit	
Respiratory system (PaO2/FiO2 (mmHg))	◯> 400<br>◯< 400<br>◯< 300<br>◯< 200 with respiratoy support<br>◯< 100 with respiratoy support
Nervous system (Glasgow Coma Scale)	◯15<br>◯13-14<br>◯10-12<br>◯6-9<br>◯<6
Cardiovascular system (Mean arterial pressure (MAP) or administration of vasopressor required)	◯MAP > 70 mmHg<br>◯MAP < 70 mmHg<br>◯Dopamine 5 µg/kg/min or dobutamine any dose<br>◯Dopamine > 5 µg/kg/min or epinephrine 0.1 µg/kg/min or norepinephrine 0.1 µg/kg/min<br>◯Dopamine > 15 µg/kg/min OR epinephrine > 0.1 µg/kg/min OR norepinephrine > 0.1 µg/kg/min
Liver (Bilirubin (mg/dl) [µmol/l])	◯< 1.2 [< 20]<br>◯1.2 - 1.9 [20 - 32]<br>◯2.0 - 5.9 [33 - 101]<br>◯6.0 - 11.9 [102 - 204]<br>◯> 12.0 [> 204]
Coagulation (Platelets x103/ml)	◯> 150<br>◯100-150<br>◯50-99<br>◯20-49<br>◯< 20
Kidneys (Creatinine (mg/dl) [µmol/L]; urine output)	◯< 1.2 [< 110]<br>◯1.2 - 1.9 [110 - 170]<br>◯2.0 - 3.4 [171 - 299]<br>◯3. - 4.9 [300 - 440]<br>◯> 5.0 [> 440]

Oxygen saturation (SaO2)

Centre ID	
Patient ID	
Visit	
ECMO	◯yes ◯no
Type of ventilation	◯no<br>◯Nasal high-flow oxygen therapy<br>◯Noninvasive mechanical ventilation<br>◯Invasive ventilation<br>◯Tracheotomy<br>◯Ambient air<br>◯Standard oxygen sources
Amount of oxygen	____________________ liter/min
Oxygen saturation (SaO2)	____________________ %
Amount of supplemental oxygen that is required to keep SaO2 above 94%	____________________ liter/min
Fraction of Inspired Oxygen (FiO2)	____________________ %
Partial pressure of oxygen (PaO2)	____________________ mmHg
Partial pressure of carbon dioxid (PaCO2)	____________________ mmHg

Hematology

Centre ID	
Patient ID	
Visit	
Date of sample taken	____ _____ ______

Hematology

Parameter	not done	Result	Unit
Hemoglobin	◯yes		▼
RBC	◯yes		▼
WBC	◯yes		▼
Thrombocytes	◯yes		▼

Blood chemistry and coagulation

Centre ID	
Patient ID	
Visit	
Date of sample taken	____ ______ ________

Clinical chemistry
<small>Please use a point '.' as decimal separator.</small>

Parameter	not done	Result	Unit
BUN	◯yes		
Creatinine	◯yes		
Albumin	◯yes		
AST/SGOT	◯yes		
ALT/SGPT	◯yes		
Total bilirubin	◯yes		
GGT	◯yes		
AP	◯yes		
LDH	◯yes		
Sodium	◯yes		
Potassium	◯yes		
Magnesium	◯yes		
Calcium	◯yes		
Uric acid	◯yes		
Troponin	◯yes		
CK	◯yes		U/l
CK-MB	◯yes		U/l
PTT	◯yes		sec
ATIII	◯yes		%
D-Dimer	◯yes		
Fibrinogen	◯yes		
Ferritin	◯yes		
Transferrin	◯yes		
Transferrin Saturation	◯yes		%
CRP	◯yes		
Total protein	◯yes		g/l
IL6	◯yes		pg/ml
Procalcitonin	◯yes		ng/ml
Total IgG	◯yes		
IgA	◯yes		
IgM	◯yes		
Lactate	◯yes		
INR	◯yes		

Concomitant medication

Centre ID	
Patient ID	
Visit	
Any new or changed concomitant medication since last visit	◯yes ◯no
If yes, please document all changes in Concomitant medications in the following section:	
Link to Concomitant medication	

	Seq. no.	Drug Name	Indicatio n	Start date	Ongoing at end of study	Stop date

Set Link / Create Entry ...

Concomitant COVID-19 therapy

Centre ID	
Patient ID	
Visit	
Any new Concomitant COVID-19 therapy since last visit?	○yes ○no

If yes, please document all changes in Concomitant COVID-19 therapy in the following section:

Link to Concomitant COVID-19 therapy

	Therapy number	Therapy	Total Daily Dose	Unit	Onset date of medication	Still ongoing	Stop date of therapy

Set Link / Create Entry ...

Adverse events

Centre ID	
Patient ID	
Visit	
Any new Adverse events since last visit?	◯yes ◯no
If yes, please document all new Adverse Events in the following section:	
Link to Adverse Events	see table below

	AE number	SOC of AE (according CTCAE V5.0)	AE term (according CTCAE V5.0)	Start date	Ongoing at the end of study	Stop date	Is the AE serious?

Set Link / Create Entry ...

Day 56

Centre ID	
Patient ID	
Visit	Day 56
Date of Visit	____ ______ ________
Hospitalization?	◯yes ◯no

- Vital signs
- Seven point ordinal scale
- Sequential Organ Failure Assessment (SOFA) Score
- Oxygen saturation (SaO2)
- Hematology
- Blood chemistry and coagulation
- Concomitant medication
- Concomitant COVID-19 therapy
- Adverse events

Investigator's Signature

Meaning:	I confirm the completeness and correctness of all documented data.
Signed By:	
Signature Date:	

Vital signs

Centre ID	
Patient ID	
Visit	
WHO performance status	◯ECOG 0 = Fully active, able to carry on all pre-disease performance without restriction<br>◯ECOG 1 = Restricted in physically strenuous activity but ambulatory and able to carry out work of a light or sedentary nature, e.g., light house work, office work<br>◯ECOG 2 = Ambulatory and capable of all selfcare but unable to carry out any work activities; up and about more than 50% of waking hours<br>◯ECOG 3 = Capable of only limited self-care, confined to bed or chair more than 50% of waking hours<br>◯ECOG 4 = Completely disabled. Cannot carry on any self-care. Totally confined to bed or chair.
Body temperature	____________________ °C
Type of measurement	◯oral ◯tympanic
Systolic blood pressure	____________________ mmHg
Diastolic blood pressure	____________________ mmHg
Pulse rate	____________________ beats/min
respiratory rate	____________________ breaths/min

Seven point ordinal scale

Centre ID	
Patient ID	
Visit	
Time of record	____ ____ ____
Ordinal Scale for Clinical Improvement	◯1 = not hospitalized with resumption of normal activities<br>◯2 = not hospitalized, but unable to resume normal sctivities<br>◯3 = hospitalized,not requiring supplemental oxygen<br>◯4 = hospitalized, requiring supplemental oxygen<br>◯5 = hospitalized, requiring nasal high-flow oxygen therapy or, noninvasive mechanical ventilation<br>◯6 = hospitalized, requiring ECMO, invasive mechanical ventilation, or both<br>◯7 = death

Sequential Organ Failure Assessment (SOFA) Score

Centre ID	
Patient ID	
Visit	
Respiratory system (PaO2/FiO2 (mmHg))	◯> 400<br>◯< 400<br>◯< 300<br>◯< 200 with respiratoy support<br>◯< 100 with respiratoy support
Nervous system (Glasgow Coma Scale)	◯15<br>◯13-14<br>◯10-12<br>◯6-9<br>◯<6
Cardiovascular system (Mean arterial pressure (MAP) or administration of vasopressor required)	◯MAP > 70 mmHg<br>◯MAP < 70 mmHg<br>◯Dopamine 5 µg/kg/min or dobutamine any dose<br>◯Dopamine > 5 µg/kg/min or epinephrine 0.1 µg/kg/min or norepinephrine 0.1 µg/kg/min<br>◯Dopamine > 15 µg/kg/min OR epinephrine > 0.1 µg/kg/min OR norepinephrine > 0.1 µg/kg/min
Liver (Bilirubin (mg/dl) [µmol/l])	◯< 1.2 [< 20]<br>◯1.2 - 1.9 [20 - 32]<br>◯2.0 - 5.9 [33 - 101]<br>◯6.0 - 11.9 [102 - 204]<br>◯> 12.0 [> 204]
Coagulation (Platelets x103/ml)	◯> 150<br>◯100-150<br>◯50-99<br>◯20-49<br>◯< 20
Kidneys (Creatinine (mg/dl) [µmol/L]; urine output)	◯< 1.2 [< 110]<br>◯1.2 - 1.9 [110 - 170]<br>◯2.0 - 3.4 [171 - 299]<br>◯3. - 4.9 [300 - 440]<br>◯> 5.0 [> 440]

Oxygen saturation (SaO2)

Centre ID	
Patient ID	
Visit	
ECMO	◯yes ◯no
Type of ventilation	◯no<br>◯Nasal high-flow oxygen therapy<br>◯Noninvasive mechanical ventilation<br>◯Invasive ventilation<br>◯Tracheotomy<br>◯Ambient air<br>◯Standard oxygen sources
Amount of oxygen	____________________ liter/min
Oxygen saturation (SaO2)	____________________ %
Amount of supplemental oxygen that is required to keep SaO2 above 94%	____________________ liter/min
Fraction of Inspired Oxygen (FiO2)	____________________ %
Partial pressure of oxygen (PaO2)	____________________ mmHg
Partial pressure of carbon dioxid (PaCO2)	____________________ mmHg

Hematology

Centre ID	
Patient ID	
Visit	
Date of sample taken	____ _____ _______

Hematology

Parameter	not done	Result	Unit
Hemoglobin	○yes		
RBC	○yes		
WBC	○yes		
Thrombocytes	○yes		

Blood chemistry and coagulation

Centre ID	
Patient ID	
Visit	
Date of sample taken	____ ______ ________

Clinical chemistry
<small>Please use a point '.' as decimal separator.</small>

Parameter	not done	Result	Unit
BUN	yes		
Creatinine	yes		
Albumin	yes		
AST/SGOT	yes		
ALT/SGPT	yes		
Total bilirubin	yes		
GGT	yes		
AP	yes		
LDH	yes		
Sodium	yes		
Potassium	yes		
Magnesium	yes		
Calcium	yes		
Uric acid	yes		
Troponin	yes		
CK	yes		U/l
CK-MB	yes		U/l
PTT	yes		sec
ATIII	yes		%
D-Dimer	yes		
Fibrinogen	yes		
Ferritin	yes		
Transferrin	yes		
Transferrin Saturation	yes		%
CRP	yes		
Total protein	yes		g/l
IL6	yes		pg/ml
Procalcitonin	yes		ng/ml
Total IgG	yes		
IgA	yes		
IgM	yes		
Lactate	yes		
INR	yes		

Concomitant medication

Centre ID	
Patient ID	
Visit	
Any new or changed concomitant medication since last visit	◯yes ◯no
If yes, please document all changes in Concomitant medications in the following section:	
Link to Concomitant medication	

	Seq. no.	Drug Name	Indication	Start date	Ongoing at end of study	Stop date

Set Link / Create Entry ...

Concomitant COVID-19 therapy

Centre ID	
Patient ID	
Visit	
Any new Concomitant COVID-19 therapy since last visit?	◯yes ◯no

If yes, please document all changes in Concomitant COVID-19 therapy in the following section:

Link to Concomitant COVID-19 therapy

	Therapy number	Therapy	Total Daily Dose	Unit	Onset date of medication	Still ongoing	Stop date of therapy

Set Link / Create Entry ...

Adverse events

Centre ID	
Patient ID	
Visit	
Any new Adverse events since last visit?	◯yes ◯no
If yes, please document all new Adverse Events in the following section:	
Link to Adverse Events	(see table below)

	AE number	SOC of AE (according CTCAE V5.0)	AE term (according CTCAE V5.0)	Start date	Ongoing at the end of study	Stop date	Is the AE serious?

Set Link / Create Entry ...

Day 70

Centre ID	
Patient ID	
Visit	Day 70
Date of Visit	____ ______ ________
Hospitalization?	◯yes ◯no

- Vital signs
- Seven point ordinal scale
- Sequential Organ Failure Assessment (SOFA) Score
- Oxygen saturation (SaO2)
- Hematology
- Blood chemistry and coagulation
- Concomitant medication
- Concomitant COVID-19 therapy
- Adverse events

Investigator's Signature

Meaning:	I confirm the completeness and correctness of all documented data.
Signed By:	
Signature Date:	

Vital signs

Centre ID	
Patient ID	
Visit	
WHO performance status	◯ECOG 0 = Fully active, able to carry on all pre-disease performance without restriction<br>◯ECOG 1 = Restricted in physically strenuous activity but ambulatory and able to carry out work of a light or sedentary nature, e.g., light house work, office work<br>◯ECOG 2 = Ambulatory and capable of all selfcare but unable to carry out any work activities; up and about more than 50% of waking hours<br>◯ECOG 3 = Capable of only limited self-care, confined to bed or chair more than 50% of waking hours<br>◯ECOG 4 = Completely disabled. Cannot carry on any self-care. Totally confined to bed or chair.
Body temperature	____________________ °C
Type of measurement	◯oral ◯tympanic
Systolic blood pressure	____________________ mmHg
Diastolic blood pressure	____________________ mmHg
Pulse rate	____________________ beats/min
respiratory rate	____________________ breaths/min

Seven point ordinal scale

Centre ID	
Patient ID	
Visit	
Time of record	____ ____ ____
Ordinal Scale for Clinical Improvement	◯1 = not hospitalized with resumption of normal activities<br>◯2 = not hospitalized, but unable to resume normal sctivities<br>◯3 = hospitalized,not requiring supplemental oxygen<br>◯4 = hospitalized, requiring supplemental oxygen<br>◯5 = hospitalized, requiring nasal high-flow oxygen therapy or, noninvasive mechanical ventilation<br>◯6 = hospitalized, requiring ECMO, invasive mechanical ventilation, or both<br>◯7 = death

Sequential Organ Failure Assessment (SOFA) Score

Centre ID	
Patient ID	
Visit	
Respiratory system (PaO2/FiO2 (mmHg))	◯> 400<br>◯< 400<br>◯< 300<br>◯< 200 with respiratoy support<br>◯< 100 with respiratoy support
Nervous system (Glasgow Coma Scale)	◯15<br>◯13-14<br>◯10-12<br>◯6-9<br>◯<6
Cardiovascular system (Mean arterial pressure (MAP) or administration of vasopressor required)	◯MAP > 70 mmHg<br>◯MAP < 70 mmHg<br>◯Dopamine 5 µg/kg/min or dobutamine any dose<br>◯Dopamine > 5 µg/kg/min or epinephrine 0.1 µg/kg/min or norepinephrine 0.1 µg/kg/min<br>◯Dopamine > 15 µg/kg/min OR epinephrine > 0.1 µg/kg/min OR norepinephrine > 0.1 µg/kg/min
Liver (Bilirubin (mg/dl) [µmol/l])	◯< 1.2 [< 20]<br>◯1.2 - 1.9 [20 - 32]<br>◯2.0 - 5.9 [33 - 101]<br>◯6.0 - 11.9 [102 - 204]<br>◯> 12.0 [> 204]
Coagulation (Platelets x103/ml)	◯> 150<br>◯100-150<br>◯50-99<br>◯20-49<br>◯< 20
Kidneys (Creatinine (mg/dl) [µmol/L]; urine output)	◯< 1.2 [< 110]<br>◯1.2 - 1.9 [110 - 170]<br>◯2.0 - 3.4 [171 - 299]<br>◯3. - 4.9 [300 - 440]<br>◯> 5.0 [> 440]

Oxygen saturation (SaO2)

Centre ID	
Patient ID	
Visit	
ECMO	◯yes ◯no
Type of ventilation	◯no<br>◯Nasal high-flow oxygen therapy<br>◯Noninvasive mechanical ventilation<br>◯Invasive ventilation<br>◯Tracheotomy<br>◯Ambient air<br>◯Standard oxygen sources
Amount of oxygen	____________________ liter/min
Oxygen saturation (SaO2)	____________________ %
Amount of supplemental oxygen that is required to keep SaO2 above 94%	____________________ liter/min
Fraction of Inspired Oxygen (FiO2)	____________________ %
Partial pressure of oxygen (PaO2)	____________________ mmHg
Partial pressure of carbon dioxid (PaCO2)	____________________ mmHg

Hematology

Centre ID	
Patient ID	
Visit	
Date of sample taken	____ ______ ________

Hematology

Parameter	not done	Result	Unit
Hemoglobin	◯yes		
RBC	◯yes		
WBC	◯yes		
Thrombocytes	◯yes		

Blood chemistry and coagulation

Centre ID	
Patient ID	
Visit	
Date of sample taken	____ ______ ________

Clinical chemistry
<small>Please use a point '.' as decimal separator.</small>

Parameter	not done	Result	Unit
BUN	yes		
Creatinine	yes		
Albumin	yes		
AST/SGOT	yes		
ALT/SGPT	yes		
Total bilirubin	yes		
GGT	yes		
AP	yes		
LDH	yes		
Sodium	yes		
Potassium	yes		
Magnesium	yes		
Calcium	yes		
Uric acid	yes		
Troponin	yes		
CK	yes		U/l
CK-MB	yes		U/l
PTT	yes		sec
ATIII	yes		%
D-Dimer	yes		
Fibrinogen	yes		
Ferritin	yes		
Transferrin	yes		
Transferrin Saturation	yes		%
CRP	yes		
Total protein	yes		g/l
IL6	yes		pg/ml
Procalcitonin	yes		ng/ml
Total IgG	yes		
IgA	yes		
IgM	yes		
Lactate	yes		
INR	yes		

Concomitant medication

Centre ID	
Patient ID	
Visit	
Any new or changed concomitant medication since last visit	◯yes ◯no
If yes, please document all changes in Concomitant medications in the following section:	
Link to Concomitant medication	

	Seq. no.	Drug Name	Indicatio n	Start date	Ongoing at end of study	Stop date

Set Link / Create Entry ...

Concomitant COVID-19 therapy

Centre ID	
Patient ID	
Visit	
Any new Concomitant COVID-19 therapy since last visit?	◯yes ◯no

If yes, please document all changes in Concomitant COVID-19 therapy in the following section:

Link to Concomitant COVID-19 therapy

	Therapy number	Therapy	Total Daily Dose	Unit	Onset date of medication	Still ongoing	Stop date of therapy

Set Link / Create Entry ...

Adverse events

Centre ID	
Patient ID	
Visit	
Any new Adverse events since last visit?	◯yes ◯no
If yes, please document all new Adverse Events in the following section:	
Link to Adverse Events	

	AE number	SOC of AE (according CTCAE V5.0)	AE term (according CTCAE V5.0)	Start date	Ongoing at the end of study	Stop date	Is the AE serious?

Set Link / Create Entry ...

Day 84

Centre ID	
Patient ID	
Visit	Day 84
Date of Visit	____ ______ ________
Hospitalization?	◯yes ◯no

- Vital signs
- Seven point ordinal scale
- Sequential Organ Failure Assessment (SOFA) Score
- Oxygen saturation (SaO2)
- Hematology
- Blood chemistry and coagulation
- Concomitant medication
- Concomitant COVID-19 therapy
- Adverse events

Investigator's Signature

Meaning:	I confirm the completeness and correctness of all documented data.
Signed By:	
Signature Date:	

Vital signs

Centre ID	
Patient ID	
Visit	
WHO performance status	◯ECOG 0 = Fully active, able to carry on all pre-disease performance without restriction<br>◯ECOG 1 = Restricted in physically strenuous activity but ambulatory and able to carry out work of a light or sedentary nature, e.g., light house work, office work<br>◯ECOG 2 = Ambulatory and capable of all selfcare but unable to carry out any work activities; up and about more than 50% of waking hours<br>◯ECOG 3 = Capable of only limited self-care, confined to bed or chair more than 50% of waking hours<br>◯ECOG 4 = Completely disabled. Cannot carry on any self-care. Totally confined to bed or chair.
Body temperature	____________________ °C
Type of measurement	◯oral ◯tympanic
Systolic blood pressure	____________________ mmHg
Diastolic blood pressure	____________________ mmHg
Pulse rate	____________________ beats/min
respiratory rate	____________________ breaths/min

Seven point ordinal scale

Centre ID	
Patient ID	
Visit	
Time of record	____ ____ ____
Ordinal Scale for Clinical Improvement	◯1 = not hospitalized with resumption of normal activities<br>◯2 = not hospitalized, but unable to resume normal sctivities<br>◯3 = hospitalized,not requiring supplemental oxygen<br>◯4 = hospitalized, requiring supplemental oxygen<br>◯5 = hospitalized, requiring nasal high-flow oxygen therapy or, noninvasive mechanical ventilation<br>◯6 = hospitalized, requiring ECMO, invasive mechanical ventilation, or both<br>◯7 = death

Sequential Organ Failure Assessment (SOFA) Score

Centre ID	
Patient ID	
Visit	
Respiratory system (PaO2/FiO2 (mmHg))	◯ > 400<br>◯ < 400<br>◯ < 300<br>◯ < 200 with respiratoy support<br>◯ < 100 with respiratoy support
Nervous system (Glasgow Coma Scale)	◯ 15<br>◯ 13-14<br>◯ 10-12<br>◯ 6-9<br>◯ <6
Cardiovascular system (Mean arterial pressure (MAP) or administration of vasopressor required)	◯ MAP > 70 mmHg<br>◯ MAP < 70 mmHg<br>◯ Dopamine 5 µg/kg/min or dobutamine any dose<br>◯ Dopamine > 5 µg/kg/min or epinephrine 0.1 µg/kg/min or norepinephrine 0.1 µg/kg/min<br>◯ Dopamine > 15 µg/kg/min OR epinephrine > 0.1 µg/kg/min OR norepinephrine > 0.1 µg/kg/min
Liver (Bilirubin (mg/dl) [µmol/l])	◯ < 1.2 [< 20]<br>◯ 1.2 - 1.9 [20 - 32]<br>◯ 2.0 - 5.9 [33 - 101]<br>◯ 6.0 - 11.9 [102 - 204]<br>◯ > 12.0 [> 204]
Coagulation (Platelets x103/ml)	◯ > 150<br>◯ 100-150<br>◯ 50-99<br>◯ 20-49<br>◯ < 20
Kidneys (Creatinine (mg/dl) [µmol/L]; urine output)	◯ < 1.2 [< 110]<br>◯ 1.2 - 1.9 [110 - 170]<br>◯ 2.0 - 3.4 [171 - 299]<br>◯ 3. - 4.9 [300 - 440]<br>◯ > 5.0 [> 440]

Oxygen saturation (SaO2)

Centre ID	
Patient ID	
Visit	
ECMO	◯yes ◯no
Type of ventilation	◯no<br>◯Nasal high-flow oxygen therapy<br>◯Noninvasive mechanical ventilation<br>◯Invasive ventilation<br>◯Tracheotomy<br>◯Ambient air<br>◯Standard oxygen sources
Amount of oxygen	____________________ liter/min
Oxygen saturation (SaO2)	____________________ %
Amount of supplemental oxygen that is required to keep SaO2 above 94%	____________________ liter/min
Fraction of Inspired Oxygen (FiO2)	____________________ %
Partial pressure of oxygen (PaO2)	____________________ mmHg
Partial pressure of carbon dioxid (PaCO2)	____________________ mmHg

Hematology

Centre ID	
Patient ID	
Visit	
Date of sample taken	____ ______ ________

Hematology

Parameter	not done	Result	Unit
Hemoglobin	◯yes		
RBC	◯yes		
WBC	◯yes		
Thrombocytes	◯yes		

Blood chemistry and coagulation

Centre ID	
Patient ID	
Visit	
Date of sample taken	___ ___ ___

Clinical chemistry

<small>Please use a point '.' as decimal separator.</small>

Parameter	not done	Result	Unit
BUN	yes		
Creatinine	yes		
Albumin	yes		
AST/SGOT	yes		
ALT/SGPT	yes		
Total bilirubin	yes		
GGT	yes		
AP	yes		
LDH	yes		
Sodium	yes		
Potassium	yes		
Magnesium	yes		
Calcium	yes		
Uric acid	yes		
Troponin	yes		
CK	yes		U/l
CK-MB	yes		U/l
PTT	yes		sec
ATIII	yes		%
D-Dimer	yes		
Fibrinogen	yes		
Ferritin	yes		
Transferrin	yes		
Transferrin Saturation	yes		%
CRP	yes		
Total protein	yes		g/l
IL6	yes		pg/ml
Procalcitonin	yes		ng/ml
Total IgG	yes		
IgA	yes		
IgM	yes		
Lactate	yes		
INR	yes		

Concomitant medication

Centre ID	
Patient ID	
Visit	
Any new or changed concomitant medication since last visit	◯yes ◯no
If yes, please document all changes in Concomitant medications in the following section:	
Link to Concomitant medication	

	Seq. no.	Drug Name	Indicatio n	Start date	Ongoing at end of study	Stop date

Set Link / Create Entry ...

Concomitant COVID-19 therapy

Centre ID	
Patient ID	
Visit	
Any new Concomitant COVID-19 therapy since last visit?	○yes ○no
If yes, please document all changes in Concomitant COVID-19 therapy in the following section:	
Link to Concomitant COVID-19 therapy	(see table below)

	Therapy number	Therapy	Total Daily Dose	Unit	Onset date of medication	Still ongoing	Stop date of therapy

Set Link / Create Entry ...

Adverse events

Centre ID	
Patient ID	
Visit	
Any new Adverse events since last visit?	◯yes ◯no

If yes, please document all new Adverse Events in the following section:

Link to Adverse Events

	AE number	SOC of AE (according CTCAE V5.0)	AE term (according CTCAE V5.0)	Start date	Ongoing at the end of study	Stop date	Is the AE serious?

Set Link / Create Entry ...

Global Pages

Centre ID	
Patient ID	
Visit	Global Page

- Adverse Events
- Previous and concomitant medication
- Concomitant COVID-19 therapy
- Cross-over assessment
- Discharge of Hospitalization
- Rehospitalization
- Serodiagnostic
- Protocol violations
- Premature termination of Study

Investigator's Signature

Meaning:	I confirm the completeness and correctness of all documented data.
Signed By:	
Signature Date:	

Adverse Events

Centre ID	
Patient ID	
Visit	

Adverse Events

Add Adverse Events...

	AE number	SOC of AE (according CTCAE V5.0)	AE term (according CTCAE V5.0)	Start date	Ongoing at the end of study	Stop date	Is the AE serious?

Adverse Events

AE number	
SOC of AE (according CTCAE V5.0)	▾
AE term (according CTCAE V5.0)	▾
AE term (according CTCAE V5.0)	▾
AE term (according CTCAE V5.0)	◯Congenital, familial and genetic disorders - Other, specify
AE term (according CTCAE V5.0)	▾
AE term (according CTCAE V5.0)	▾
AE term (according CTCAE V5.0)	▾
AE term (according CTCAE V5.0)	▾
AE term (according CTCAE V5.0)	▾
AE term (according CTCAE V5.0)	▾
AE term (according CTCAE V5.0)	▾
AE term (according CTCAE V5.0)	▾
AE term (according CTCAE V5.0)	▾
AE term (according CTCAE V5.0)	▾
AE term (according CTCAE V5.0)	▾
AE term (according CTCAE V5.0)	▾
AE term (according CTCAE V5.0)	▾
AE term (according CTCAE V5.0)	▾
AE term (according CTCAE V5.0)	▾
AE term (according CTCAE V5.0)	▾
AE term (according CTCAE V5.0)	▾
AE term (according CTCAE V5.0)	▾
AE term (according CTCAE V5.0)	▾
AE term (according CTCAE V5.0)	▾
AE term (according CTCAE V5.0)	◯Social circumstances - Other, specify
AE term (according CTCAE V5.0)	◯Surgical and medical procedures - Other, specify
AE term (according CTCAE V5.0)	▾
Specification	____
Start date	___ ___ ___
Ongoing at end of study	◯yes ◯no
Stop date	___ ___ ___
Serious adverse event (SAE)?	◯yes ◯no
SAE number	____
Intensity CTCAE V5.0 grade	◯Grade 1 - mild
Intensity CTCAE V5.0 grade	◯Grade 2 - moderate

Intensity CTCAE V5.0 grade	◯Grade 3 - severe
Intensity CTCAE V5.0 grade	◯Grade 4 - life-threatening
Intensity CTCAE V5.0 grade	◯Grade 5 - death
Intensity CTCAE V5.0 grade	◯Grade 1 - mild<br>◯Grade 2 -moderate
Intensity CTCAE V5.0 grade	◯Grade 1 - mild<br>◯Grade 3 - severe
Intensity CTCAE V5.0 grade	◯Grade 2 - moderate<br>◯Grade 3 - severe
Intensity CTCAE V5.0 grade	◯Grade 3 - severe<br>◯Grade 4 - life-threatening
Intensity CTCAE V5.0 grade	◯Grade 4 - life-threatening<br>◯Grade 5 - death
Intensity CTCAE V5.0 grade	◯Grade 1 - mild<br>◯Grade 2 - moderate<br>◯Grade 3 - severe
Intensity CTCAE V5.0 grade	◯Grade 1 - mild<br>◯Grade 4 - life-threatening<br>◯Grade 5 - death
Intensity CTCAE V5.0 grade	◯Grade 2 - moderate<br>◯Grade 3 - severe<br>◯Grade 4 - life-threatening
Intensity CTCAE V5.0 grade	◯Grade 3 - severe<br>◯Grade 4 - life-threatening<br>◯Grade 5 - death
Intensity CTCAE V5.0 grade	◯Grade 1 - mild<br>◯Grade 2 - moderate<br>◯Grade 3 - severe<br>◯Grade 4 - life-threatening
Intensity CTCAE V5.0 grade	◯Grade 1 - mild<br>◯Grade 2 - moderate<br>◯Grade 3 - severe<br>◯Grade 5 - death
Intensity CTCAE V5.0 grade	◯Grade 1 - mild<br>◯Grade 3 - severe<br>◯Grade 4 - life-threatening<br>◯Grade 5 - death
Intensity CTCAE V5.0 grade	◯Grade 2 - moderate<br>◯Grade 3 - severe<br>◯Grade 4 - life-threatening<br>◯Grade 5 - death
Intensity CTCAE V5.0 grade	◯Grade 1 - mild<br>◯Grade 2 - moderate<br>◯Grade 3 - severe<br>◯Grade 4 - life-threatening<br>◯Grade 5 - death
Relationship to IMP	◯Related ◯Unrelated
Action taken to IMP	________ ▾
Outcome	________ ▾
Date of Death	____ ______ ________
Countermeasures taken	◯yes ◯no
Drug treatment	◯yes ◯no
Other treatments	◯yes ◯no
Specification or other treatments	____________

Previous and concomitant medication

Centre ID	
Patient ID	
Visit	

Previous and concomitant medication

Add Concomitant medication...

	Seq. no.	Drug Name	Indication	Start date	Ongoing at end of study	Stop date

Concomitant medication

Seq. no.	
Drug Name	________________
Indication	________________
Total Daily Dose	______________________
Unit	______________________ ▼
if other: please specify	________________
Frequency	______________________ ▼
if other: Please specify	________________
Start date	____ ______ ________
Ongoing at end of study	◯yes ◯no
if no: Stop date	____ ______ ________
Route of administration	______________________ ▼
if other: Please specify	________________

Concomitant COVID-19 therapy

Centre ID	
Patient ID	
Visit	

Prior and concomitant COVID-19 therapy

Add Therapy of COVID-19...

	Therapy number	Therapy	Total Daily Dose	Unit	Onset date of medication	Still ongoing	Stop date of therapy

Therapy of COVID-19

Therapy number	
Therapy	▼
Please specify therapy name	
Dosis known?	○yes ○no
Total Daily Dose	
Unit	○g ○mg ○ml ○µg ○IU ○other
if other: please specify	
Onset date of therapy	
Still ongoing	○yes ○no
Stop date of therapy	

Cross-over assessment

Centre ID	
Patient ID	
Visit	
Cross-over assessment?	◯yes ◯no
Date of Cross-over assessment	____ ______ ________
Reason of Cross-over	◯No improvement of clinical condition ◯Worsening of clinical condition

Eligibility

Signs/symptoms

Physical examination

ECG

Eligibility

Centre ID	
Patient ID	
Visit	

Inclusion criteria

All inclusion criteria fulfilled	◯yes ◯no
1. PCR confirmed SARS-CoV-2 infection in a respiratory sample	◯yes ◯no
2. Oxygen saturation (SaO2) of 94% or less while breathing ambient air or a ratio of the partial pressure of oxygen (PaO2) to the fraction of inspired oxygen (FiO2) of less than 300 mm Hg	◯yes ◯no
3. High risk due to either	◯yes ◯no
pre-existing or concurrent hematological malignancy and/or active cancer therapy (incl. chemotherapy, radiotherapy, surgery) within the last 24 months or less (group 1)	◯yes
chronic immunosupression not meeting the criteria of group 1 (group 2)	◯yes
Age ≥ 50 - 75 years meeting neither the criteria of group 1 nor group 2 (group 3) and at least one of these criteria: Lymphopenia < 0.8 x G/l and/or D-dimer > 1µg/mL	◯yes
Age ≥ 75 years meeting neither the criteria of group 1 nor group 2 (group 4)	◯yes
4. Blood hemoglobin concentration ≥ 10 g/dl	◯yes ◯no
5. Provision of written informed consent	◯yes ◯no
6. Patient is able to understand and comply with the protocol for the duration of the study, including treatment and scheduled visits and examinations	◯yes ◯no
7. Male or female patient aged ≥ 18 years	◯yes ◯no
8. Postmenopausal or evidence of non-childbearing status. For women of childbearing potential: negative urine or serum pregnancy test within 14 days prior to study treatment	◯yes ◯no

Exclusion criteria

None of the exclusion criteria is fulfilled	◯yes ◯no
1. Dementia, psychiatric or cognitive illness or recreational drug/alcohol use that in the opinion of the principal investigator, would affect subject safety and/or compliance	◯yes ◯no
2. Contraindication to transfusion or history of prior reactions to transfusion blood products	◯yes ◯no
3. Patients with known selective IgA deficiency	◯yes ◯no
4. Patients with mechanical ventilation and/or extracoporal membrane oxygenation (ECMO) at time of initial inclusion into the trial.	◯yes ◯no
5. Participation in another trial with an investigational medicinal product	◯yes ◯no
6. Treatment with SARS-CoV-2 convalescent plasma in	◯yes ◯no

	the past	◯yes ◯no

Signs/symptoms

Centre ID	
Patient ID	
Visit	
Cough	◯yes ◯no
Fever	◯yes ◯no
Myalagia	◯yes ◯no
Fatigue	◯yes ◯no
Diarrhoea	◯yes ◯no
Vomitting	◯yes ◯no
Headache	◯yes ◯no
Abdominal pain	◯yes ◯no
Nausea	◯yes ◯no
Olfactory or taste disorder	◯yes ◯no
Dyspnea	◯yes ◯no
Confusion	◯yes ◯no

Physical examination

Centre ID	
Patient ID	
Visit	
Date of physical examination	____ ______ ________

Physical examination

Physical Examination	not done	Status	Please specify	Clinically significant
Inspection (General appearance)	◯not done	◯normal ◯abnormal	________	◯yes ◯no
Lung	◯not done	◯normal ◯abnormal	________	◯yes ◯no
Cardiac auscultation	◯not done	◯normal ◯abnormal	________	◯yes ◯no
Cardiac percussion	◯not done	◯normal ◯abnormal	________	◯yes ◯no
Abdominal auscultation	◯not done	◯normal ◯abnormal	________	◯yes ◯no
Abdominal percussion	◯not done	◯normal ◯abnormal	________	◯yes ◯no
Abdominal palpation	◯not done	◯normal ◯abnormal	________	◯yes ◯no
Palpation of lymph nodes sites	◯not done	◯normal ◯abnormal	________	◯yes ◯no
Neurological examination	◯not done	◯normal ◯abnormal	________	◯yes ◯no
Peripheral edema	◯not done	◯normal ◯abnormal	________	◯yes ◯no

Other physical examination? ◯yes ◯no

If 'Other physical examination?' answer with 'yes' then add

Add Other Physical examination...

	Number	Physical examination	Status	Please specify	Clinically significant

Other Physical examination

Number	
Body system	____________________
Status	◯normal ◯abnormal
if abnormal: Please specify	____________________
if abnormal: Clinically significant	◯yes ◯no

ECG

Centre ID	
Patient ID	
Visit	
Date of examination	____ ______ ________
Result of ECG	◯normal ◯abnormal
Clinically significant	◯yes ◯no
Description	_______________
Heart rate	____________________ beats/min
Rhythm	◯Normal sinus ◯Other
Please specify	_______________

Discharge of Hospitalization

Centre ID	
Patient ID	
Visit	
Date of discharge	____ ______ ________

Rehospitalization

Centre ID	
Patient ID	
Visit	

Rehospitalization

Add Rehospitalization...

	Number	Reason	Please specify	Date of admission	Date of discharge

Rehospitalization

Number of rehospitalization	
Reason for rehospitalization	◯Infection/fever ◯Other
if other: Please specify	________________
Date of admission	____ ______ ________
Date of discharge	____ ______ ________

Serodiagnostic

Centre ID	
Patient ID	
Visit	

Serology

Add Serodiagnostic...

	Serology No.	Date of sample collection	Serodiagnostic by ELISA (OD Ratio)	Neutralize antibody titre (1:....)	Immunofluorescence

Serodiagnostic

Serology No.	
Date of sample collection	____ ______ ________
Serodiagnostic by ELISA (OD Ratio)	____________________
Neutralize antibody titre (1:....)	▼
Immunofluorescence	◯ Positive<br>◯ Borderline positive<br>◯ Negative<br>◯ Not performed

Protocol violations

Centre ID	
Patient ID	
Visit	

Protocol violations

Add Protocol violation...

	Protocol violation number	Date of protocol violation	Specification of protocol violation

Protocol violation

Protocol violation number	
Date of protocol violation	____ ______ ________
Specification of protocol violation	_______________

Premature termination of Study

Centre ID	
Patient ID	
Visit	
Date of premature termination	____ ______ ________
Main reason for premature termination	◯Withdrawal of informed consents<br>◯Death<br>◯Lost to follow up<br>◯Pregnancy<br>◯Other
if other: Please specify	____________________
Date of death	____ ______ ________
Cause of death	◯Due to COVID-19<br>◯Due to cancer progression<br>◯Other
if other cause of death: please specify	____________________
